# Enantiopure Pyridinium Bisretinoids of Ocular Lipofuscin with Hexahydrobenzofuran Structure: Total Synthesis and Structure-Dependent Aggregated Morphology

**DOI:** 10.1021/acs.joc.5c02843

**Published:** 2026-01-27

**Authors:** Brais Vidal, Rafael Rodríguez, Angeles Peña-Gallego, Rosana Álvarez, Claudio Martínez, Ángel R. de Lera

**Affiliations:** † CINBIO, Departamento de Química Orgánica, 16784 Universidade de Vigo, IBIV, As Lagoas-Marcosende, 36310 Vigo, Spain; ‡ Departamento de Química Física, Universidade de Vigo, As Lagoas-Marcosende, 36310 Vigo, Spain

## Abstract

Oxidized photoproducts of pyridinium bisretinoid A2E, including the mono- and bishexahydrobenzofurans, which have been isolated from lipofuscin in the retinal pigment epithelium (RPE) cells of human eyes, have been synthesized in enantiopure form using as key step a Horner-Wadsworth-Emmons (HWE) condensation reaction of pyridinecarbaldehydes and enantiopure cyclohexene oxide pentadienylphosphonates. The synthesis of the trienylcyclohexene oxide branch on the shorter arm (S) of the pyridine ring was followed by a diastereoselective rearrangement to the hexahydrobenzofurandienyl substituent under acidic conditions. In contrast, the construction of the polyenic long arm (L) of the pyridine ring by HWE condensation evolved to the formation of diastereomeric hexahydrobenzofurantrienyl substituents in an unselective rearrangement. The alternative and more straightforward bidirectional HWE condensation of the cyclohexene oxide pentadienylphosphonates with 4-formylpyridine-2-butenal afforded a more complex mixture of products, from which the bishexahydrobenzofurans together with the 11-*cis* double bond isomer and a rearrangement product in S were also characterized. DFT studies on model systems provided a mechanistic rationale for these transformations. The pyridinium bisretinoid hexahydrobenzofurans underwent aggregation upon nanoprecipitation using methanol/water solvent mixtures, and (5′*R*,8′*R*)-L-*trans*-hexahydrobenzofuran-A2E (**9a**) was shown by TEM to form spherical aggregates.

## Graphical abstract

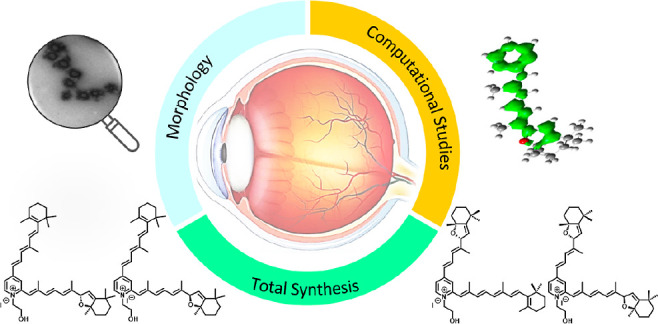

## Introduction

The absorption of light in rod and cone photoreceptors by visual pigments, which contain a protonated Schiff base derived from the condensation of 11-*cis*-retinal (**1**, Figure 1A) and a lysine group (Lys296) of the protein opsin, is the first event on the complex visual process in vertebrates.^1–3^ The visual cycle^4^ proceeds with the isomerization of the chromophore to the all-*trans* geometry.^5^ After hydrolysis, all-*trans*-retinal (**2**, Figure 1A) is converted back to **1** in the photoreceptor cells through reduction to all-*trans*-retinol (**3**, Figure 1A) by the action of NADPH-dependent retinol dehydrogenases (RDH8, RDH11, and RDH12), isomerization of the latter via the esters (**4**, Figure 1A) to 11-*cis*-retinol (**5**, Figure 1A) promoted by retinal pigment epithelium (RPE)-specific 65 kDa protein (RPE65) isomerohydrolase, and oxidation by retinol dehydrogenase.^1–3^

**Figure 1:**
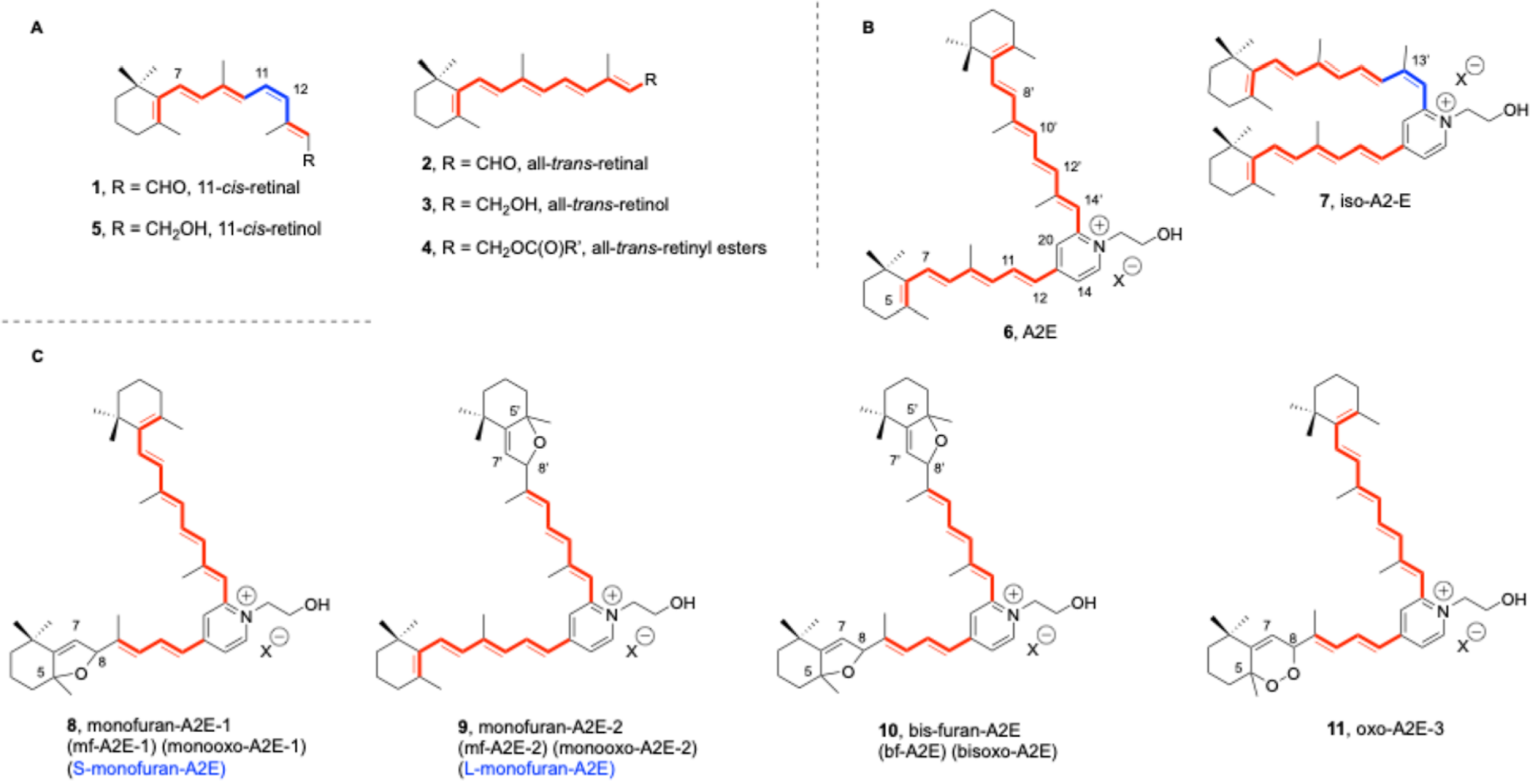
A**)**. Retinoids implicated in the visual cycle. (B**)**. Pyridinium bis-retinoids (**6,7**). (C**)**. Oxidized derivatives (**8–11**) of A2E (**6**).

An excess of all-*trans*-retinal (**2**) in human eyes due to anomalies of the retinoid metabolic routes^6^ perturbs lipid metabolism and produces a buildup of pro-inflammatory lipid-containing granules of fluorophores and protein debris called lipofuscin that accumulate in RPE phagolysosomes.^7^ Pyridinium ions substituted with cyclohexenyl-containing unsaturated fragments (tetraenes and pentaenes; the most common are shown in Figure 1B) derived from all-*trans*-retinal (**2**) are members of the structurally complex mixture of lipofuscin components.^8^ The most abundant of these fluorophores, namely A2PE, is generated through condensation of two molecules of all*-trans*-retinal (**2**) with dipalmitoyl-l-β-phosphatidylethanolamine.^9^ Further enzymatic hydrolysis of the bis-retinoid fluorophore precursors^8,10,11^ in the photoreceptor outer segment membrane generates as major product *N*-retinylidene-*N*-retinylethanolamine or A2E (**6**, Figure 1B).^7,8,12,13^ A ca. 4:1 photostationary equilibrium mixture of A2E (**6**) and its C13’ = C14′ *cis* isomer, namely *iso*-A2E (**7**, Figure 1B) has been isolated from human RPE,^14,15^ and shown to be present upon photochemical irradiation of eye extracts.^8,13,14^ A2E was proposed to also function as a sensitizer on the photochemical generation of singlet oxygen from triplet oxygen.^16,17^

Understanding the etiology of eye diseases and their effects on the visual cycle, as well as the genetic defects of the enzymes responsible for these processes, is crucial to suggest therapeutic options.^8,18–20^ In this regard, ABCR is a high molecular weight glycoprotein found in photoreceptor red outer segment disk membranes (foveal and peripheral cone) that functions as a photoreceptor-specific ATP-binding cassette transporter. Mutations in the gene encoding ABCR have been implicated in eye diseases such as Stargard’s macular dystrophy.^21,22^

Oxidized photoproducts of A2E (**6**)^11,23^ were first detected in the organic soluble portion of human retinal lipofuscin.^24,25^ As a model of the photooxidation occurring in the eyes, they were also detected in the organic extracts of bovine RPE cells derived from calf eyes fed with A2E (**6**) following irradiation of the samples under ambient conditions.^23,26,27^ They could also be generated when solutions of A2E-laden RPE in abcr^–/–^ mice were exposed to blue light.^26,28^

Although the small quantities of these compounds precluded further structural studies, larger amounts where obtained upon treating solutions of synthetic A2E (**6**)^29^ in MeOH with 2 equiv of *meta*-chloroperoxybenzoic acid (MCPBA) for 12 h at ambient temperature in the dark.^26^ ^1^H NMR and UV spectroscopic characterization of the main components of the reaction mixture confirmed the presence of the 5,8,5′,8′-bishexahydrobenzofuran **10** (Figure 1C) (first named 5,8,5′,8′-bis-furanoid oxide, or bis-furan-A2E or bf-A2E^25^ or bis-oxo-A2E^26^), the 5,8- and 5′,8′-hexahydrobenzofurans **8** and **9** (Figure 1C)^24^ at the shorter (S) or long (L) arms of A2E (also called monofuran-A2E, mf-A2E-1 and mf-A2E-2, respectively, or monooxo-A2E),^11,23,26^ and the bisoxygenated 5,8-monoperoxy-A2E **11** (first named oxo-A2E-1) (Figure 1C).^11,23^

The hexahydrobenzofurans were proposed to be generated upon rearrangement under acidic conditions of the corresponding cyclohexene oxides, which would be preferentially formed, as occurs with the bisoxygenated analogue **11** (Figure 1C), by oxidation at the most electron-rich endocyclic double bond (C5 = C6 and/or C5′ = C6′ as indicated in Figure 1C). Since lipofuscin accumulates within the RPE cells in lysosomes, which are more acidic than the surrounding cytoplasm,^30^ it was anticipated^25^ that these hexahydrobenzofurans would be generated from the rearrangement of the first formed cyclohexene oxides promoted by small amounts of acid.^31^

Despite the limited and incomplete spectroscopic characterization of these (bis)­hexahydrobenzofurans (Figure 1C),^25,26^ and their comparison with the racemates obtained from the oxidation of A2E (**6**) with MCPBA, no diastereo- and enantioselective synthesis of these compounds has been reported, and therefore we targeted them as an extension of our work on enantiopure hexahydrobenzofuran-containing polyenes.^32,33^

Given the stereochemical complexity of the target compounds with six/seven stereogenic double bonds and the presence of two stereocenters in the monohexahydrobenzofurans-A2E (**8**,**9**) and four stereocenters in the bishexahydrobenzofuran-A2E (**10**), a total of 4 and 16 stereoisomers sharing the all-*trans* geometry of the double bonds are possible for these compounds. Being the absolute and relative configurations of the stereocenters unknown due do the minute amounts isolated from human retinal lipofuscin,^24–26^ we selected a single enantiomer of the putative biogenetic precursors 5,6-cyclohexene oxides **12**–**14** (Scheme 1) and directed our efforts to explore the generation of the second stereocenter, as previously demonstrated for carotenoids containing that motif.^32–35^ Based on these precedents, we considered feasible that the 5,8-(bis)­hexahydrobenzofurans **8**, **9** and **10** would be generated by the acid-promoted rearrangement of the corresponding enantiopure A2E-related 5,6-cyclohexene oxides **12**–**14** and the latter by pyridine alkylation of precursors **15**–**17**. The longer and shorter conjugated arms of these bis-unsaturated pyridines would result from the stepwise condensation reaction^33^ of pyridinecarbaldehyde **20** with cyclohexene oxide dienylphosphonates **18** and **19** as complementary partners to reach **15** and **16**, or by the double condensation reaction of **21** with **18** to construct **17** (Scheme 1). Enantiopure **18** has been previously prepared^33^ using a sequence that included as key step an enantioselective Sharpless asymmetric epoxidation of a precursor allylic alcohol.^36^

**Scheme 1:**
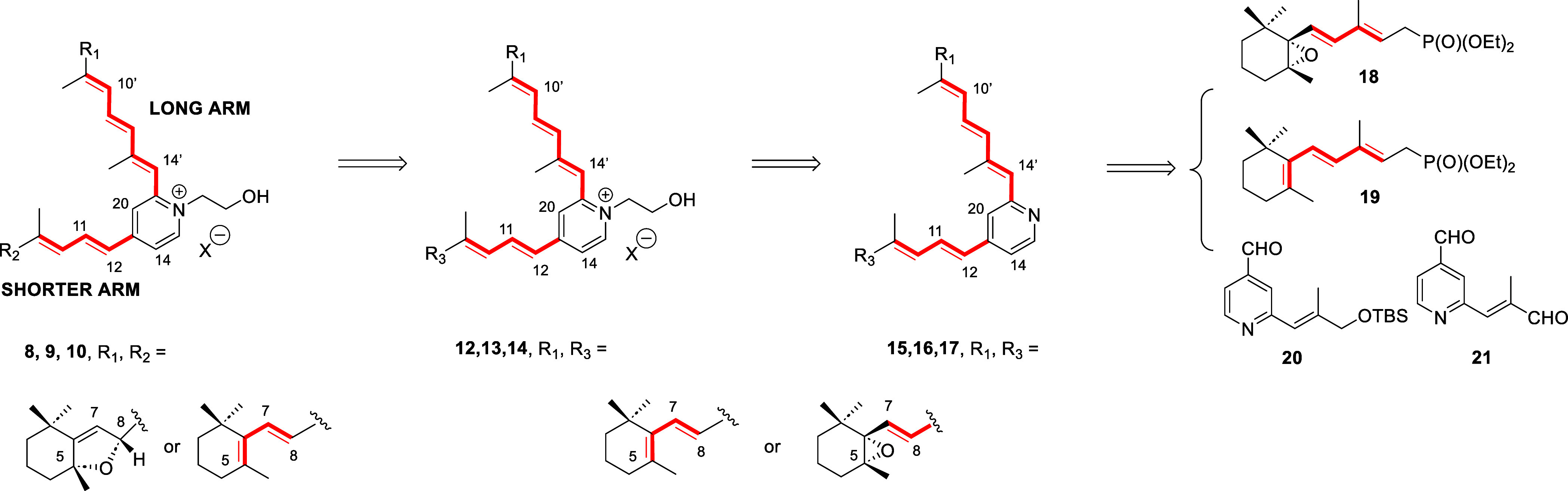
Retrosynthetic Analysis of the Stepwise and Bidirectional HWE Reaction for the Total Synthesis of Enantiopure A2E-Oxidized Derivatives Containing Hexahydrobenzofuran Rings (**8–10**)

## Results and Discussion

### Synthetic Studies

As target for methodological development, the mono-oxidized A2E in the shorter arm (namely, **8**, Figure 1C) was first selected. Based on our prior experience on hexahydrobenzofuran containing carotenoids,^33^ the Horner-Wadsworth-Emmons (HWE) condensation reaction^37–42^ was considered reliable to construct the polyene fragments on each arm of the disubstituted pyridine skeleton.

The required 2-alkenylisonicotinaldehyde **20** was generated in 96% yield by Suzuki–Miyaura cross-coupling reaction^43–45^ of commercial 2-bromoisonicotinaldehyde **25** with alkenylboronate **24** promoted by Pd­(OAc)_2_ and PPh_3_ with Na_2_CO_3_ as base in dioxane at 95 °C (Scheme 2). Alkenylboronate **24** was previously formed in 78% yield upon heating, at 80 °C in DMSO solution, protected (*E*)-3-iodo-2-methylpropenol **22** with bis­(pinacolato)­diboron **23** under catalysis of Pd­(dppf)­Cl_2_·CH_2_Cl_2_ in the presence of KOAc.^46^

**Scheme 2:**
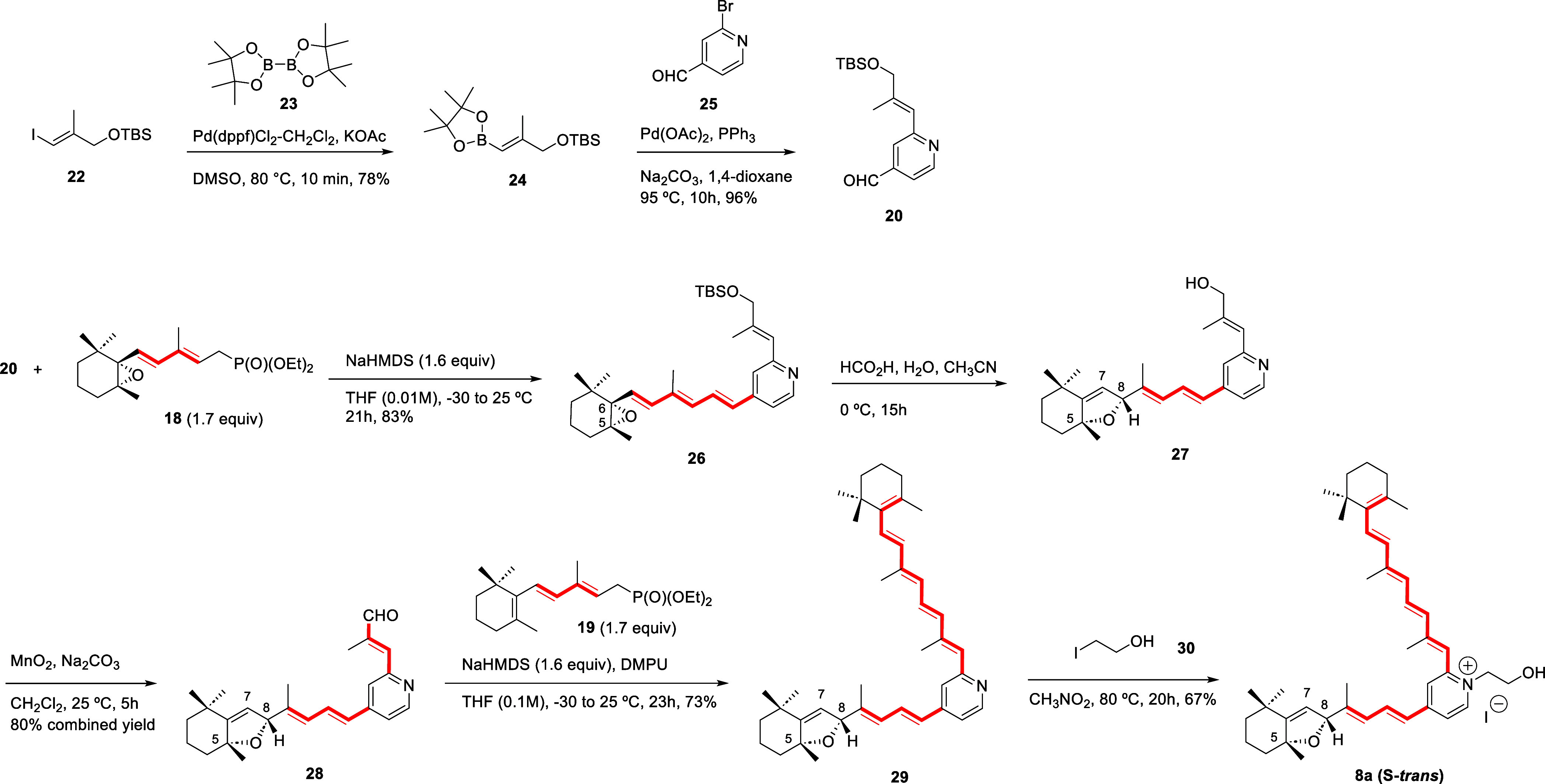
Bidirectional HWE Condensation Reaction for the Total Synthesis of Diastereomer **8a**

For the HWE condensation^37–42^ of aldehyde **20** and C_15_ phosphonate **18** ^33–36,47^ an excess (1.7 mol equivalents) of the anion generated using NaHMDS (1.6 mol equivalents) as base provided the trienylcyclohexene oxide fragment on the shorter arm of **26** with complete stereoselectivity as *E* isomer in 83% yield (Scheme 2). Under the classical phosphonate anion generation conditions (KO*t*Bu, THF, rt) used previously for the synthesis of related carotenoids,^34,35,49^ a 3:1 *trans*/*cis* isomer ratio was obtained (see Supporting Information).

Deprotection of the allylic alcohol under acidic conditions^32^ was accompanied by the rearrangement of the C5,C8-alkenylcyclohexene oxide fragment to the hexahydrobenzofuran of *R* configuration at C8 (**27**, Scheme 2) as major stereoisomer (5:1 dr) in excellent yields.^48^ Oxidation of the allylic alcohol **27** using MnO_2_ afforded the C2-methylpropenal-substituted pyridine **28** in 80% combined yield, which was subjected to the second HWE condensation.^37–42^ This step required optimization, since the use of NaHMDS as a base under the same conditions used for **26** (Scheme 2) afforded compound **29** incorporating the additional unsaturated fragment, but in low yields (27% at most). Fortunately, addition of DMPU to the reaction media increased the yield to 73% and afforded stereoselectively the thermodynamically favored^50^ all-trans isomer of the pentaene branch present in **29**. After HPLC purification (Chiralpak IA column, 25 × 1 cm; heptane/CH_2_Cl_2_/EtOH/Et_2_NH 70:30:1:0.1 v/v/v/v), full characterization confirmed the structure of the bis-polyenic pyridine **29**.

As discussed in the case of carotenoids with bis-hexahydrobenzofuran skeletons,^33^ structural confirmation of the C8 configuration in **29** and synthetic precursors (Scheme 2) rested on the chemical shift values for H7 and H8 of the hexahydrobenzofuran ring,^25^ which appear in the δ ≈ 5.0–5.3 ppm region of the ^1^H NMR spectra. To compare the data with those previously reported for bishexahydrobenzofuran-A2E (vide infra),^26^ the ^1^H NMR spectra were acquired in C_6_D_6_, and the data correlated with those of related carotenoids. In these more conjugated (bis)­hexahydrobenzofuran-containing carotenoids it has been shown that the chemical shift differences of H7 and H8 for the *trans* diastereomers of 8*R* relative configuration are very small (Δδ_H7–H8_ ≈ 0.02 ppm) and the signal for H7 appears as a broad singlet. In contrast, those for the 8*S* diastereomer show larger chemical shift differences (Δδ_H7–H8_ ≈ 0.15–0.22 ppm) and noticeable coupling constants (*J*_H7–H8_ > 1.4 Hz).^33–35^ Based on these precedents, the ^1^H NMR data for the major disubstituted pyridine **29** (and also for pyridinium salt **8a**, vide infra) showed full consistency with those of the 8*R* stereoisomer, given the small chemical shift differences noted for H7 and H8 (δ values of 5.22 and 5.15 ppm, respectively, in CD_2_Cl_2_ solutions).^26^ The *R* configuration of the newly formed C8 stereocenter in **27** was further supported by the NOE correlations observed between the methyl group at C5 and C8-H.

Lastly, alkylation of the pyridine was carried out under similar conditions to those reported for A2E (**6**),^29,51^ namely heating **29** with 2-iodoethanol (**30**) in nitromethane but at 80 °C instead of 100 °C for 20h, and (5*R*,8*R*)-S-*trans*-hexahydrobenzofuran-A2E (**8a**, Scheme 2) was obtained in 67% yield. The spectroscopic data of **8a** followed the same trends described above for the disubstituted pyridines and confirmed the formation of the hydroxyethylpyridinium ion,^26^ thus complementing the limited characterization data reported for the racemate.^25,26^

Inspired on the previous results with **8**, a similar approach but with the reversed order of the reacting phosphonates, was next adopted for the synthesis of enantiopure **9** (Scheme 3) containing a trienylhexahydrobenzofuran motif in the longer pentaenyl arm of A2E (**6**). The first HWE condensation reaction^37–42^ of the anion of trienylphosphonate **19** generated using NaHMDS at −30 °C with C2-substituted pyridine-2-carbaldehyde **20** took place in this case with a 3:1 *E/Z* diastereoselectivity of the newly formed olefin on the tetraene fragment.^52–54^ The geometric isomers **31** and **32** could be easily separated by column chromatography. Following the deprotection of the allylic alcohol of **31** (HCO_2_H, 89% yield), oxidation of **33** with MnO_2_ and Na_2_CO_3_ afforded enal **34** in 94% yield (Scheme 3A).

**Scheme 3:**
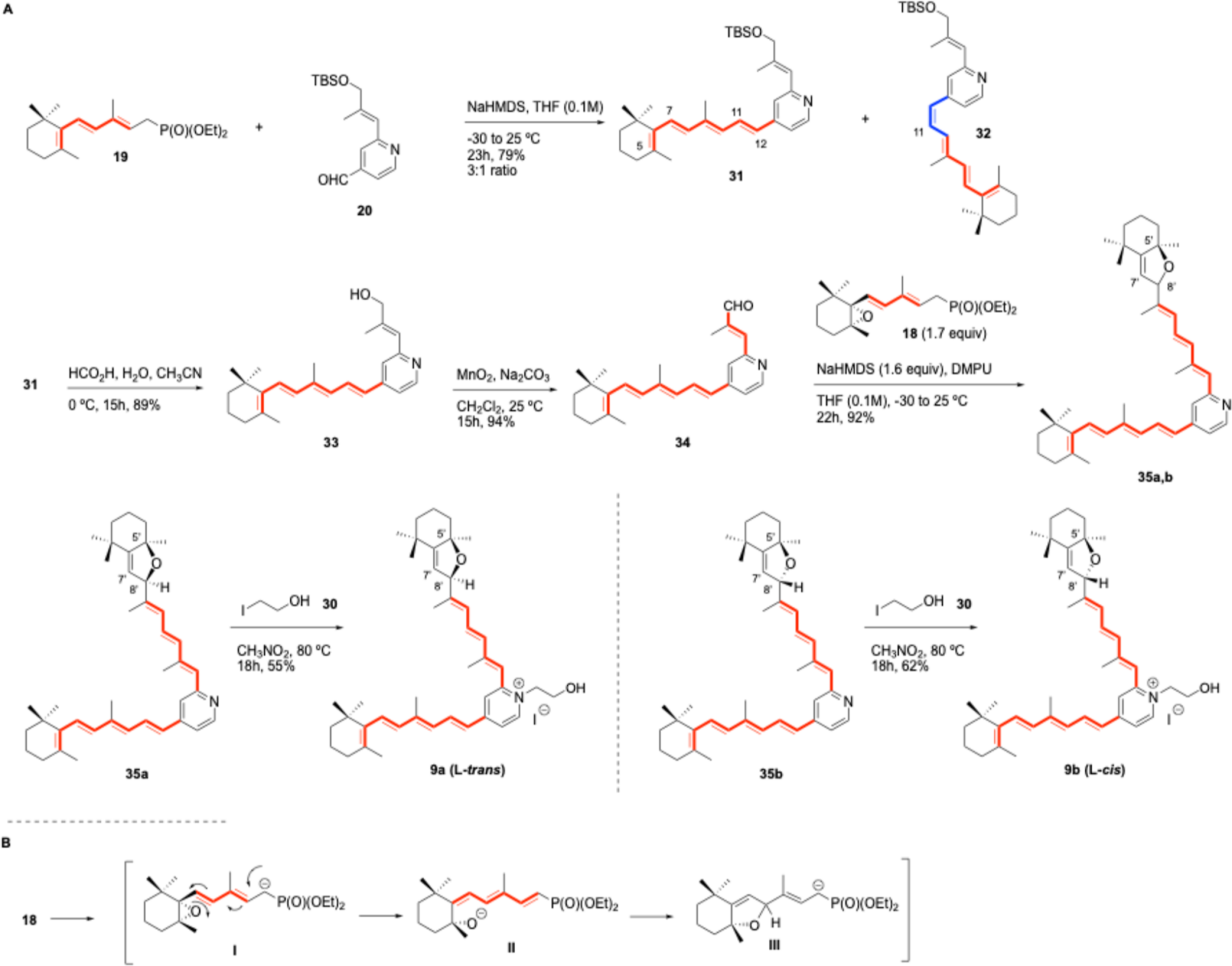
(A). Iterative HWE Reaction for the Total Synthesis of the Diastereomers of **9**: (B). Proposed Reaction Pathway for Formation of the Anion of Cyclohexene Oxide Dienylphosphonate **18** and Rearrangement of Reactive Anionic Species **I** to **III**

The second HWE condensation reaction of **34** with the anion of the enantiopure cyclohexene oxide pentadienylphosphonate **18** ^33–36,47^ generated using NaHMDS as a base and DMPU, under the same conditions described for the formation of **26** (Scheme 2), afforded the thermodynamically favored^50^ all-*trans* isomer of the newly formed unsaturated branch in excellent yield (92%). However, the NMR data analysis confirmed that the hexahydrobenzofuran skeleton was already present, and in contrast to the epoxytrienyl chain at C4, the rearrangement of the cyclohexene oxide fragment on the epoxytetraenyl chain at C2 of the pyridine ring had taken place under the basic reaction conditions. Moreover, a 50:50 mixture of diastereomers at the new stereocenter at C8 with polyene fragments of all-*trans* geometry was isolated (Scheme 3A).^55^

The finding is in agreement with the previously proposed^34,49^ reactivity of phosphonate anion **I**, which is stabilized through conjugation, and evolves by ring-opening of the 5,6-epoxide and subsequent ring-closure by conjugate addition of the generated alkoxide to the trienylphosphonate intermediate **II** (Scheme 3B) to afford the reacting 5,8-dihydrofurandienylphosphonate anion **III**,^49,55^ which would then be engaged in the condensation with alkenal **34** to provide **35** (Scheme 3A).^34,49^

In contrast to the experimental results on the rearrangement of **26** (Scheme 2) and to the general case of 5,6-cyclohexene oxides to 5,8-hexahydrobenzofurans promoted by formic acid in carotenoid structures,^32^ the formation of the hexahydrobenzofuran **35** did not show selectivity under basic conditions. To justify the diastereomeric ratio on the rearrangement of the trienylcyclohexene oxides to the hexahydrobenzofuran isomers under acidic conditions (see Scheme 2) and after formation of the phosphonate anion (Scheme 3B), these alternative mechanisms were computed by DFT using the **I** to **III** (Scheme 3B) model system (vide infra).

Diastereomers **35a** and **35b** were separated by HPLC under the same conditions and spectroscopically characterized. Each compound was converted (Scheme 3A) as described above into the corresponding pyridinium salts, namely (5′*R*,8′*R*)-L-*trans*- and (5′*R*,8′*S*)-L-*cis*-hexahydrobenzofuran-A2E (**9a** and **9b**, respectively).

^1^H NMR data of **9a** and **9b** showed full consistency with those of the 8′*R* and 8′*S* epimers, since as indicated for the smaller branch hexahydrobenzofuran **8a** (Scheme 2), the Δδ_H7′‑H8′_ for the formed diastereomer L-*trans* **9a** is very small (around 0.07 ppm in this case) and the signal for H7′ appears as a broad singlet, whereas chemical shift values for H7′ and H8′ on the L-*cis* epimer **9b** were different (δ ≈ 5.35 and 5.10 ppm, respectively) and a measurable coupling constant was clearly noted in the latter signal (*J*_H7′‑H8′_ = 1.9 Hz).^32–35^ In the case of the 8′*R* diastereomer **9a** the configuration of the newly formed C8′ stereocenter was further confirmed by the NOE effect observed between the methyl group at C5′ and the hydrogen at C8′ (Scheme 3).^56,57^

Lastly, bidirectional condensation of the conjugated anion of phosphonate **18** (NaHMDS, THF, −30 °C) with dialdehyde **21**,^29^ which was obtained from **20** after deprotection of the silyl ether with formic acid and oxidation of the allylic alcohol **36** (66% combined yield, Scheme 4), using the described Barbier conditions (addition of 3.6 equiv of **18**, from −30 to 0 °C) took place in comparable overall yields (74%) to those of the monocondensation process of **20** (Scheme 2). Surprisingly, the reaction time required for completion to obtain **37** (Scheme 4) was shorter (4 h), when compared to the case of the monocondensation (23 h). Moreover, although the main products were the hexahydrobenzofuran diastereomers at the conjugated chains (**37**), additional compounds were present in the more complex product mixture.

**Scheme 4:**
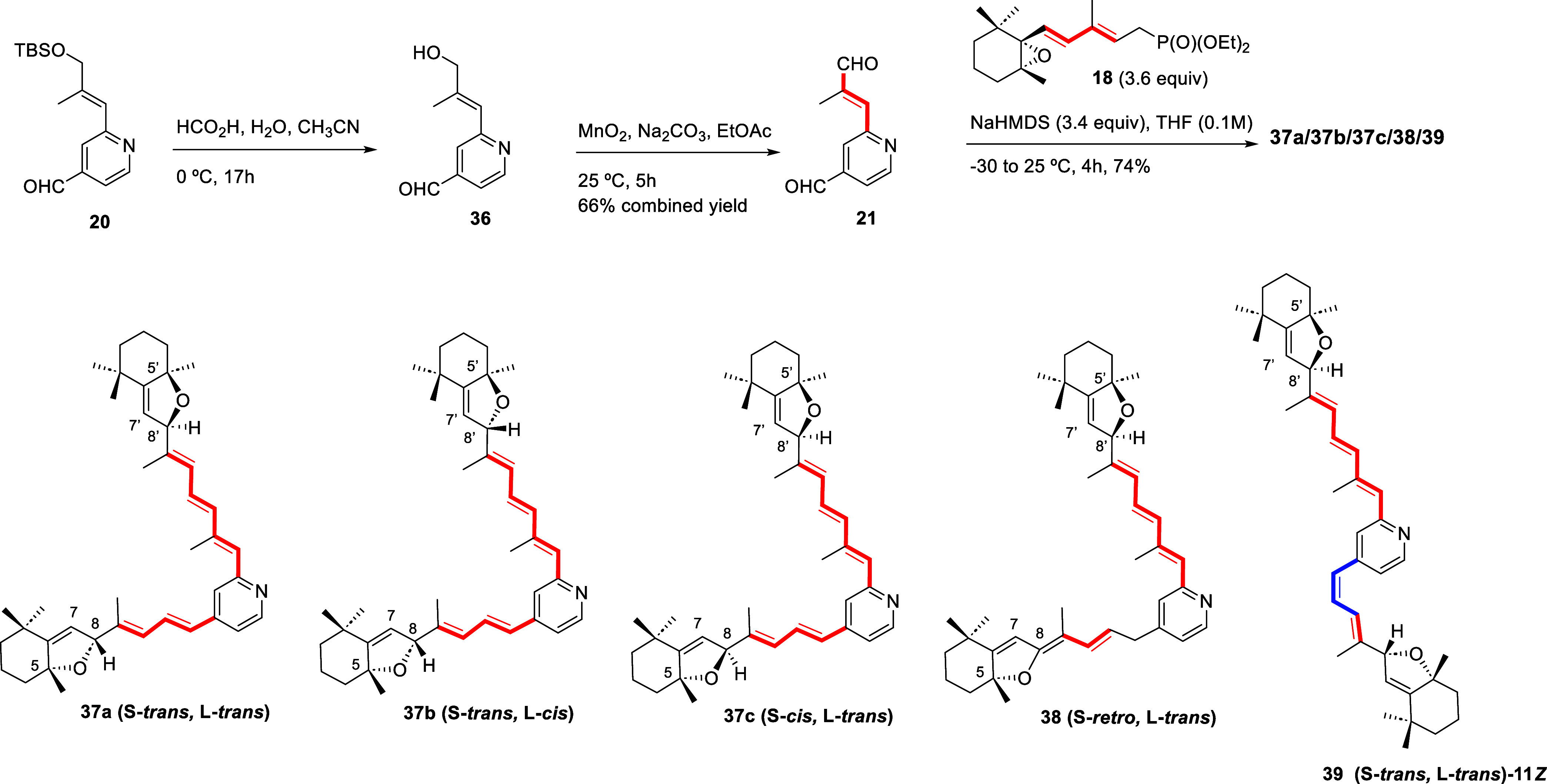
Bidirectional HWE Reaction for the Total Synthesis of Diastereomers **37a–c**, and Structures of Secondary Products **38** and **39**

HPLC purification as described above allowed to isolate compounds **37** *a* **/37** *b* **/37** *c* **/38/39** (Scheme 4) in a 1.0:0.5:0.4:0.25:0.12 relative ratio. The structure of the major components could be confirmed by comparison of their ^1^H NMR and UV data with those discussed above for the hexahydrobenzofurans (**29** and **35**). Using the same analysis of the chemical shift and coupling constant values for H7 and H8 in their ^1^H NMR spectra, the major bishexahydrobenzofuran (**37a**) was identified as the diastereomer with L-*trans*, S-*trans* relative configuration, accounting for the presence of NMR signals at δ values of 5.22, 5.21, 5.15, and 5.13 ppm, all of them as singlet, which were further assigned using NOE analysis to H7, H7́, H8, and H8′, respectively.^26^ Diastereomers at C8 and C8′ (**37b** and **37c**, respectively) were also obtained, and could be identified as those with the S-*trans*, L-*cis* (*J*_H7′‑H8′_ ≈ 1.9 Hz) and S-*cis,* L-*trans* (*J*_H7–H8_ ≈ 2.0 Hz) relative configurations, respectively, based on the coupling constant values. NOE correlations further confirmed the geometries of the conjugated skeletons and the relative and absolute configurations of the dihydrofurans fused to the cyclohexanes in these stereoisomers.

Furthermore, two minor condensation products (**38** and **39**, Scheme 4) were characterized, both of which showed structural differences on the smaller branch relative to **37**. Spectroscopic analysis was consistent with **38**, which we named S-*retro*, L-*trans*, being the product corresponding to the presumed rearrangement of the unsaturated fragment of either **37a** or **37c** (given the lack of the former H8 signals) across the C8-C12 region with formation of the alkylidenedihydrofuran structure.

The structural proposal for **39**, the stereoisomer of **37a** with 11-*cis* geometry, was based on comparison of the NMR data with those of **37a** and further analysis of the coupling constants values of the C11 = C12 double bond (overlapping of signals in CD_2_Cl_2_; *J*_H11–H12_ ≈ 11.6 Hz) and the presence of singlets assigned to H7, H7′, H8, and H8′ in the ^1^H NMR spectra. NOE correlations were fully consistent with the proposed geometries.

To improve the synthesis of the major diastereomer (**37a**), we took advantage of the diastereoselective formation of **27** from **26** under acidic conditions (Scheme 2) and subjected enal **28** to HWE condensation reaction with excess phosphonate **18**. Although the stereochemical outcome of the newly generated stereocenter was like that described for **35** (Scheme 3A), no further components were found in the reaction mixture, and bishexahydrobenzofurans **37a** and **37b** were isolated in similar yields, together with minor amounts of **37c** (Scheme 5).

**Scheme 5:**
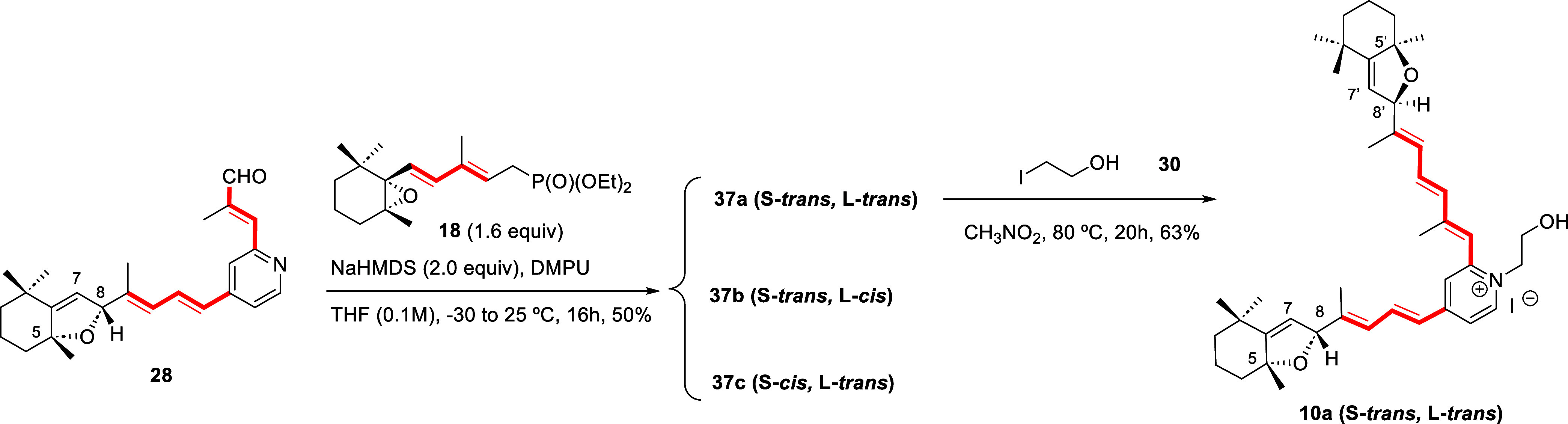
Stepwise HWE Reaction for the Total Synthesis of Diastereomers **37**, and Alkylation of the Major Diastereomer **37a** to Pyridinium Salt **10a**

Diastereomer **37a** was then treated with iodoethanol **30** as described before^29,51^ to afford (5*R*,8*R,*5′*R*,8′*R*)-S-*trans*-L-*trans*-bishexahydrobenzofuran-A2E (**10a**) in comparable yields to those of the hexahydrobenzofurans (Scheme 5). The ^1^H NMR data for this compound matched those reported for the racemate in this diastereomeric form,^26^ and the complete ^13^C NMR characterization data (previously reported^26^ only for the C5C6C7C8 dihydrofuran-containing unit) confirmed the formation of the hexahydrobenzofuran skeleton on each polyenyl branch.

In retrospect, since ^1^H NMR spectra in CD_3_OD for the synthetic hexahydrobenzofurans generated by MCPBA oxidation of A2E (**6**) showed signals at δ ≈ 5.15 ppm, δ ≈ 5.19 ppm, δ ≈ 5.22 ppm, and δ ≈ 5.26 ppm,^26^ which were assigned using HSQC to the dienyl (δ ≈ 5.15 ppm and δ ≈ 5.22 ppm) and the trienyl (δ ≈ 5.19 ppm and δ ≈ 5.26 ppm) branches, the diastereoselective synthesis along this work confirmed that the major diastereomer obtained in these experiments^26^ was the S-*trans*-L-*trans*-bishexahydrobenzofuran-A2E, presumably promoted by the acidic conditions used for HPLC purification. In natural settings, the rearrangement of the 5,6-cyclohexene oxide-A2E to the 5,8-hexahydrobenzofuran-A2E structural isomer could most likely occur under acidic conditions, in full consistency with the acidic medium of RPE lysosomes.

### Computational Studies

To justify the diastereoselective formation of hexahydrobenzofuran structures from cyclohexene oxide precursors under acidic conditions (**26** to **27**, Scheme 2), and the lack of stereoselectivity under basic conditions (**21** to **37a**–**c**) accompanied by the generation of alkylidene hexahydrobenzofuran rearrangement product **38** (Scheme 4), we carried out DFT studies on model systems **40**, **42** and **46** (Scheme 6) featuring when required one of the unsaturated side chains as substituent at C2 or C4 of the pyridine ring (see S. I. for details). In addition, we also addressed the lack of stereoselectivity on the rearrangement of the anion of cyclohexene oxide dienylphosphonate **18** (Scheme 3B) to the alkylidene hexahydrobenzofuran intermediate (**I** to **III**, Scheme 3B).

**Scheme 6:**
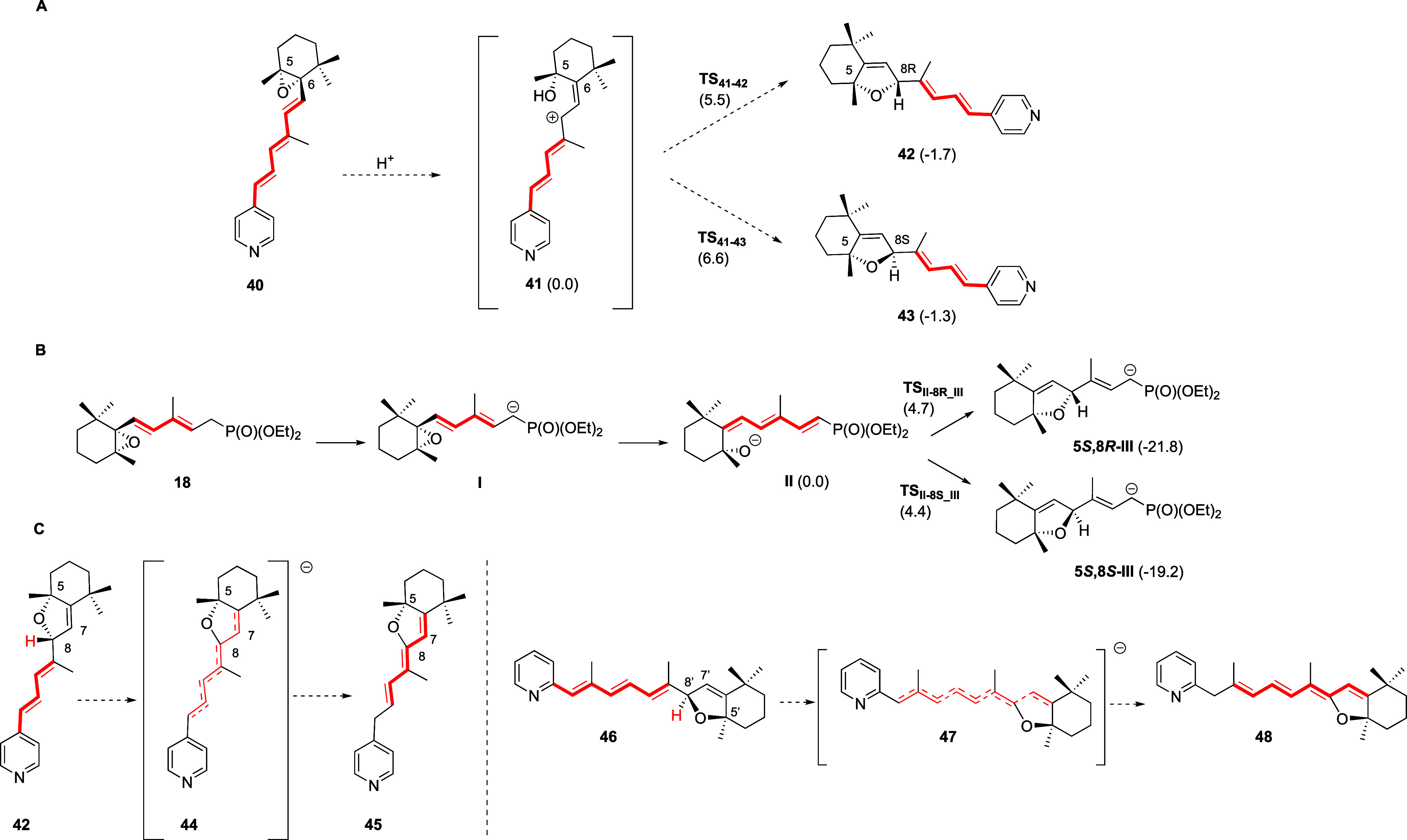
(A) Rearrangement of Model System **40** Under Acidic Conditions by Epoxide Ring Opening to **41** and Ring Closure to Diastereomers **42** and **43** (Δ*G* Values in kcal/mol are Shown in Brackets). (B) Rearrangement of Cyclohexene Oxide Dienylphosphonate **18** to Diastereomeric Hexahydrobenzofuran Alkenylphosphonates 5*S*,8*R*-**III** and 5*S*,8*S*-**III** Under Basic Conditions (Δ*G* Values in kcal/mol are Shown in Brackets). (C) Rearrangement of Model Systems **44** and **47** Under Basic Conditions Followed by Protonation to **46** and **49**, Respectively.

A. The rearrangement of pyridine-C4-trienylcyclohexene oxide model system **40** promoted by acid, which requires the ring opening of the oxirane to afford a carbocation intermediate and its trapping with the tertiary alcohol (**40** to **42** or **43**, Scheme 6A), was computed using the Gaussian 16 suite of programs.^58^ All calculations were performed at the wB97XD level,^59^ with def2svp as basis function^60^ and PCM as solvation model (in THF or CH_3_CN).^61^

For the acid-mediated rearrangement, the proposed carbenium ion intermediate **41** was shown by NBO analysis^62^ to be mostly located at C8 but enjoy stabilization by resonance given its bis-allylic structure (Figure 2). The additions to either face of the carbenium ion are exergonic, and the formation of diastereomeric hexahydrobenzofurans **42** and **43** proceeds through transition states TS_41–42_ and TS_41–43_ with computed activation energies of 5.5 and 6.6 kcal/mol, respectively (see Supporting Information). Therefore, the predicted diastereomeric ratio is consistent with that experimentally observed (ca. 5:1 *R/S*).

**Figure 2:**
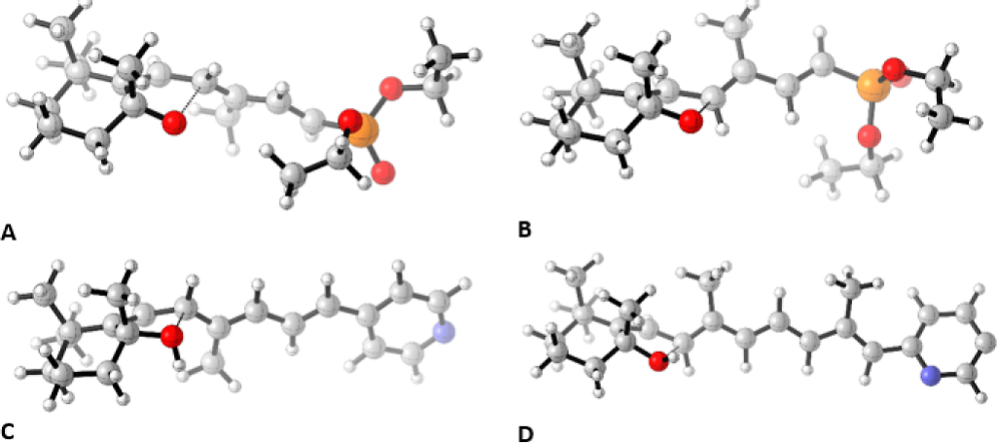
Top. Transition states TS_41–42_ (A, C^8^–O distance 2.41 Å) and TS_41–43_ (B, C^8^–O distance 2.48 Å, C^C5‑Me^-C^C9‑Me^ 5.06 Å) for the 5,6-cyclohexene oxide to 5,8-hexahydrobenzofuran rearrangement on the corresponding epoxydienylphosphonates promoted by base. Bottom. Transition states TS_II‑8*R*III_ (**C**, C^8^–O distance 2.12 Å) and TS_II‑8*S*III_ (D, C^8^–O distance 2.07 Å, C^C5‑Me^-C^C9‑Me^ 3.88 Å) for the rearrangement of 5,6-cyclohexene oxide trienylpyridine at C4 and 5,6-cyclohexene oxide tetraenylpyridine at C2, to the corresponding 5,8-hexahydrobenzofurans promoted by acid, computed at the wB97XD/def2svp (PCM, THF) level.^65^

B. We also addressed by DFT the lack of face selection, and therefore the lack of stereoselectivity on the rearrangement of the anion of phosphonate **18** in THF solution. As indicated in Scheme 3B, after ring-opening of the 5,6-epoxide, the ring-closure by conjugate addition of the generated alkoxide to C_8_ of the trienylphosphonate intermediate **II** (Scheme 6B) along the two available orientations generates the reacting diastereomeric 5,8-hexahydrobenzofuran alkenylphosphonate anions (5*R*,8*R*)**-III** and (5*R*,8*S*)**-III**.^49,55^

A conformationally stabilizing interaction between the heteroatom and H_8_ (H_8_···O distance of ca. 1.89 (Å) was characterized for alkoxide **II**, despite the 1,3-allylic interactions with the methyl groups at C1 and C5 (Scheme 6B). NBO analysis^62^ confirmed the alkoxytrienylphosphonate structure for intermediate **II** (Scheme 6B). The greater nucleophilicity of the alkoxide on **II**, as confirmed by the f(−) Fukui index,^63,64^ led to the formation of diastereomers (5*R*,8*R*)-**III** and (5*R*,8*S*)-**III** on highly exergonic processes by conjugate addition through transition states TS_II‑8*R*III_ and TS_II‑8*S*III_ (Figure 2), which have similar computed activation energies of 4.7 and 4.4 kcal/mol, respectively (Table S2). The activation energy values predict a 1:1.6 (5*R*,8*R*)-**35b**/(5*R*,8*S*)-**35b** product diastereomeric ratio, in accordance with the experimental findings of this process under kinetic control (Scheme 3).

C. The mechanism of the selective generation of alkylidenedihydrofuran on the shorter arm in compound **38** (Scheme 4) was likely due to a sequence of deprotonation at H8 taking place under the basic reaction conditions and further protonation at H12 during workup.

To validate this proposal, we estimated the p*K*_a_ of the hexahydrobenzofuran bis-allylic hydrogen on model systems **42** and **46**, as well as that of phosphonate **18**, using the simplest and direct method based on the computational determination of the free energy of the corresponding species AH, A^–^ and H_3_O^+^ by DFT (Scheme 6C) using M06-2X,^66^ with aug-cc-pVDZ as basis function,^67^ and SMD as solvation model (in water).^68^ Since the outcome of this method depends on the determination of the free energy of the solvated proton, we followed the described procedure^69^ (*G̅*_aq_H_3_O^+^ = −273.14 kcal/mol), which is based on the estimation of the average proton solvation energy from a training set of molecules. The free energies for G_aq_AH and G_aq_A^–^ were computed using the same level of theory (see Supporting Information).

The difference on p*K*_a_ values for the hexahydrobenzofuran hydrogen on model structures **42** (p*K*_a_ ≈ 19 for H_8_) relative to **46** (p*K*_a_ ≈ 22 for H_8’_) might justify the ability of the phosphonate anion (p*K*_a_ ≈ 22), itself formed by deprotonation of **18** with NaHMDS (Scheme 4), to deprotonate the hexahydrobenzofuran on the shorter arm (p*K*_a_ ≈ 19).^69^

The higher acidity of H_8_ on **42** when compared with H_8′_ on **46** could be explained by the greater stabilization of the electron density along the chain at position C4 of the pyridine ring. Electronic delocalization for both anions (**44** and **47**) was indirectly estimated by ACID (Anisotropy of the Current Induced Density) analysis,^70^ and the Critical Isosurface Values (CIVs) showed slightly stronger conjugation for **44** relative to **47** (0.056 versus 0.053, Figure S9).

### Aggregation Studies

The aggregation trend in biological media of these pyridinium bisretinoid hexahydrobenzofurans^8^ prompted us to study this behavior by UV–vis spectroscopy. Given the insolubility of all the compounds tested in water, we turned our attention to the use of a potential cosolvent fully miscible with water. This strategy has been effectively employed to aggregate not only a variety of small molecules but also of macromolecules.^71–73^ Methanol was the solvent of choice, since it solubilizes these pyridinium salts and is fully miscible with water. As shown in Figure 3, solutions of (5′*R*,8′*R*)-L-*trans*-hexahydrobenzofuran-A2E (**9a**) in CH_3_OH exhibited UV–vis spectra with absorption maxima at 292 and 425 nm. Upon gradually increasing the volume fraction of H_2_O, a concomitant hypochromic effect was observed, which is indicative of the aggregation of the molecules (see Figure S2–S6 for similar experiments performed with the analogues, and Figure S8 for UV spectra).

**Figure 3:**
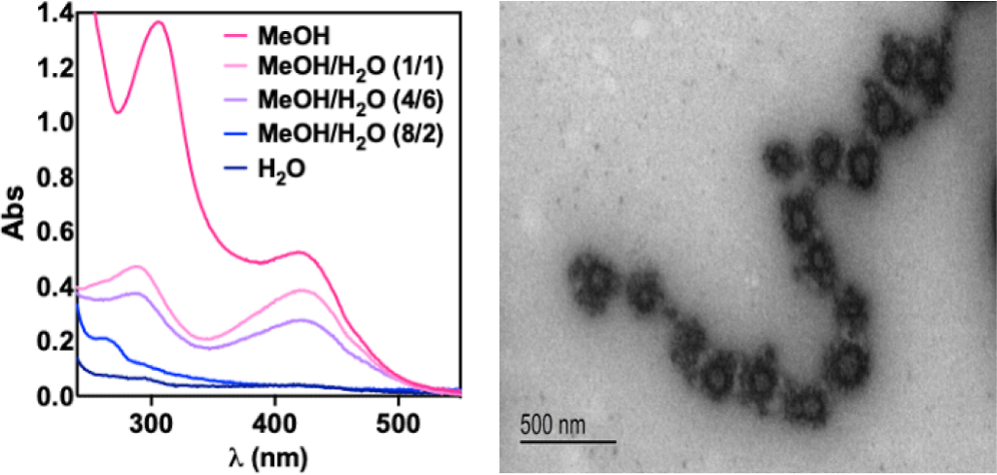
Left. UV–vis spectra of (5′*R*,8′*R*)-L-*trans*-hexahydrobenzofuran-A2E (**9a**) with different volume fractions of H_2_O in MeOH (*c*_T_ = 75 μM). Right. TEM image showing the formation of spherical aggregates of **9a** (*c*_T_ = 75 μM, 20/80 *v/v* MeOH/H_2_O).

Further evidence of the aggregation upon increasing the water volume fraction was obtained by ^1^H NMR experiments (Figure S7). In CD_3_OD, **9a** showed a well-defined NMR spectrum. However, progressive increase of D_2_O volume fractions led to a dramatical sharp decrease in NMR signal intensity, ultimately resulting in near-complete disappearance of signals in 20:80 (v/v) CD_3_OD/D_2_O solvent mixtures. This behavior is indicative of effective nanoprecipitation.

Finally, the morphology of the aggregates was studied by transmission electron microscopy (TEM). TEM images of (5′*R*,8′*R*)-L-*trans*-hexahydrobenzofuran-A2E (**9a**) showed the formation of spherical aggregates with diameters of ca. 175 nm (See Figure 3 for **9a**, Figures S2–S5 for the analogues, and Figure S6 for the estimation of the size of the aggregates).

The formation of spherical aggregates of **9a** might justify some of the deleterious effects of these compounds, since oxidized derivatives of A2E were also detected in the eye cups of mice with null mutations in *Abca4/Abcr*, the gene responsible for recessive Stargardt disease, which is the most common inherited macular dystrophy.^26,74^ Moreover, they were found to be more abundant in albino vs pigmented *abcr* ^*–/–*^ mice exposed to increasing ambient light,^28^ and were shown to also induce DNA damage,^17^ suggesting that these effects might also take place in vivo.^26^

## Conclusions

We have synthesized and fully characterized enantiopure hexahydrobenzofurans derived from the oxidation of all-*trans*-retinal condensation product A2E in the human retinal pigment epithelium (RPE). These conjugates have been shown to accumulate in human eyes with age,^75^ due to their slow or nonexistent breakdown by RPE cells. The Horner-Wadsworth-Emmons (HWE) condensation reaction conditions for olefination on the shorter arm (S) preserved the trienyl 5,6-cyclohexene oxide, which underwent stereoselective rearrangement to the hexahydrobenzofuran under acidic deprotection conditions of an allylic alcohol protected as silyl ether. The same HWE reaction for construction of the long arm (L) of the skeleton induced an unselective rearrangement which led to diastereomeric hexahydrobenzofurans on L or to bishexahydrobenzofurans starting from the dienylhexahydrobenzofuran on S. Computational studies justified the contrasting behavior of the unsaturation on each branch of the pyridine ring under acidic or basic conditions. The morphology of these diastereo- and enantiopure A2E-derived hexahydrobenzofurans was studied by TEM and shown for (5′*R*,8′*R*)-L-*trans*-hexahydrobenzofuran-A2E (**9a**) to form spherical aggregates upon nanoprecipitation using methanol/water solvent mixtures. Since A2E-derived hexahydrobenzofurans have also been detected in aged human donor RPE, and therefore contribute to the formation of lipofuscin, the toxic fluorescent material that accumulates in the retina, these synthetic spherical aggregates might be useful as models to study their formation and deposition on human eyes.

## Supplementary Material



## Data Availability

The data underlying this study are available in the published article and its Supporting Information.

## Notes

Experimental procedures, characterization data, computational methods, additional computational data, DFT energies and Cartesian coordinates for all optimized structures, and copies of NMR spectra (PDF)

## References

[1] Zhang J., Choi E. H., Tworak A., Salom D., Leinonen H., Sander C. L., Hoang T. V., Handa J. T., Blackshaw S., Palczewska G., Kiser P. D., Palczewski K. (2019). Photic generation
of 11-*cis*-retinal in bovine retinal pigment epithelium. J. Biol. Chem..

[2] Palczewski K., Kiser P. D. (2020). Shedding new light on the generation of the visual
chromophore. Proc. Natl. Acad. Sci. U.S.A..

[3] Chen S., Getter T., Salom D., Wu D., Quetschlich D., Chorev D. S., Palczewski K., Robinson C. V. (2022). Capturing a rhodopsin
receptor signalling cascade across a native membrane. Nature.

[4] Gruhl T., Weinert T., Rodrigues M. J., Milne C. J., Ortolani G., Nass K., Nango E., Sen S., Johnson P. J. M., Cirelli C., Furrer A., Mous S., Skopintsev P., James D., Dworkowski F., Båth P., Kekilli D., Ozerov D., Tanaka R., Glover H., Bacellar C., Brünle S., Casadei C. M., Diethelm A. D., Gashi D., Gotthard G., Guixà-González R., Joti Y., Kabanova V., Knopp G., Lesca E., Ma P., Martiel I., Mühle J., Owada S., Pamula F., Sarabi D., Tejero O., Tsai C.-J., Varma N., Wach A., Boutet S., Tono K., Nogly P., Deupi X., Iwata S., Neutze R., Standfuss J., Schertler G., Panneels V. (2023). Ultrafast structural changes direct
the first molecular events of vision. Nature.

[5] Hong J. D., Salom D., Kochman M. A., Kubas A., Kiser P. D., Palczewski K. (2022). Chromophore
hydrolysis and release from photoactivated
rhodopsin in native membranes. Proc. Natl. Acad.
Sci. U.S.A..

[6] Yakovleva M. A., Radchenko A. S., Feldman T. B., Kostyukov A. A., Arbukhanova P. M., Borzenok S. A., Kuzmin V. A., Ostrovsky M. A. (2020). Fluorescence
characteristics of lipofuscin fluorophores from human retinal pigment
epithelium. Photochem. Photobiol. Sci..

[7] Mata N. L., Weng J., Travis G. H. (2000). Biosynthesis of a major lipofuscin
fluorophore in mice and humans with ABCR-mediated retinal and macular
degeneration. Proc. Natl. Acad. Sci. U.S.A..

[8] Kim H. J., Sparrow J. R. (2021). Bisretinoid phospholipid and vitamin
A aldehyde: shining
light. J. Lipid Res..

[9] Bui T. V., Han Y., Radu R. A., Travis G. H., Mata N. L. (2006). Characterization
of Native Retinal Fluorophores Involved in Biosynthesis of A2E and
Lipofuscin-associated Retinopathies. J. Biol.
Chem..

[10] Wu Y., Zhou J., Fishkin N., Rittmann B. E., Sparrow J. R. (2011). Enzymatic
Degradation of A2E, a Retinal Pigment Epithelial Lipofuscin Bisretinoid. J. Am. Chem. Soc..

[11] Sparrow J. R., Gregory-Roberts E., Yamamoto K., Blonska A., Ghosh S. K., Ueda K., Zhou J. (2012). The bisretinoids of retinal pigment
epithelium. Prog. Ret. Eye Res..

[12] Sakai N., Decatur J., Nakanishi K., Eldred G. E. (1996). Ocular Age Pigment
“A2-E”: An Unprecedented Pyridinium Bisretinoid. J. Am. Chem. Soc..

[13] Sparrow J.
R., Fishkin N., Zhou J., Cai B., Jang Y. P., Krane S., Itagaki Y., Nakanishi K. (2003). A2E, a byproduct
of the visual cycle. Vision Res..

[14] Parish C. A., Hashimoto M., Nakanishi K., Dillon J., Sparrow J. (1998). Isolation
and one-step preparation of A2E and *iso*-A2E, fluorophores
from human retinal pigment epithelium. Proc.
Natl. Acad. Sci. U.S.A..

[15] Grey A. C., Crouch R. K., Koutalos Y., Schey K. L., Ablonczy Z. (2011). Spatial Localization
of A2E in the Retinal Pigment Epithelium. Invest.
Ophthalmol. Vis. Sci..

[16] Ben-Shabat S., Itagaki Y., Jockusch S., Sparrow J. R., Turro N. J., Nakanishi K. (2002). Formation of a Nonaoxirane from A2E, a Lipofuscin Fluorophore
related to Macular Degeneration, and Evidence of Singlet Oxygen Involvement. Angew. Chem., Int. Ed..

[17] Sparrow J. R., Vollmer-Snarr H. R., Zhou J., Jang Y. P., Jockusch S., Itagaki Y., Nakanishi K. (2003). A2E-epoxides Damage DNA in Retinal
Pigment Epithelial Cells: VITAMIN E AND OTHER ANTIOXIDANTS INHIBIT
A2E-EPOXIDE FORMATION. J. Biol. Chem..

[18] Sparrow J. R. (2010). Bisretinoids
of RPE Lipofuscin: Trigger for Complement Activation in Age-Related
Macular Degeneration. Adv. Exp. Med. Biol..

[19] Kiser P. D., Golczak M., Palczewski K. (2014). Chemistry
of the Retinoid (Visual)
Cycle. Chem. Rev..

[20] Wu Y., Yanase E., Feng X., Siegel M. M., Sparrow J. R. (2010). Structural
characterization of bisretinoid A2E photocleavage products and implications
for age-related macular degeneration. Proc.
Natl. Acad. Sci. U.S.A..

[21] Molday L. L., Rabin A. R., Molday R. S. (2000). ABCR expression in foveal cone photoreceptors
and its role in Stargardt macular dystrophy. Nat. Genet..

[22] Ahn J., Wong J. T., Molday R. S. (2000). The Effect
of Lipid Environment and
Retinoids on the ATPase Activity of ABCR, the Photoreceptor ABC Transporter
Responsible for Stargardt Macular Dystrophy. J. Biol. Chem..

[23] Kim S. R., Jang Y. P., Sparrow J. R. (2010). Photooxidation
of RPE lipofuscin
bisretinoids enhances fluorescence intensity. Vision Res..

[24] Avalle L. B., Wang Z., Dillon J. P., Gaillard E. R. (2004). Observation of A2E
oxidation products in human retinal lipofuscin. Exp. Eye Res..

[25] Dillon J., Wang Z., Avalle L. B., Gaillard E. R. (2004). The photochemical
oxidation of A2E results in the formation of a 5,8,5′,8’-bis-furanoid
oxide. Exp. Eye Res..

[26] Jang Y. P., Matsuda H., Itagaki Y., Nakanishi K., Sparrow J. R. (2005). Characterization of Peroxy-A2E and Furan-A2E Photooxidation
Products and Detection in Human and Mouse Retinal Pigment Epithelial
Cell Lipofuscin. J. Biol. Chem..

[27] Kim S. R., Jang Y. P., Jockusch S., Fishkin N. E., Turro N. J., Sparrow J. R. (2007). The all-trans-retinal dimer series
of lipofuscin pigments
in retinal pigment epithelial cells in a recessive Stargardt disease
model. Proc. Natl. Acad. Sci. U.S.A..

[28] Radu R. A., Mata N. L., Bagla A., Travis G. H. (2004). Light exposure
stimulates
formation of A2E oxiranes in a mouse model of Stargardt’s macular
degeneration. Proc. Natl. Acad. Sci. U.S.A..

[29] Ren R. X.-F., Sakai N., Nakanishi K. (1997). Total Synthesis
of the Ocular Age
Pigment A2-E: A Convergent Pathway. J. Am. Chem.
Soc..

[30] Feeney-Burns L., Berman E. R., Rothman H. (1980). Lipofuscin
of Human Retinal Pigment
Epithelium. Am. J. Ophthalmol..

[31] Baldas J., Porter Q. N., Cholnoky L., Szabolcs J., Weedon B. C. L. (1966). Mass
spectrometry of carotenoid epoxides and furanoid oxides. Chem. Commun..

[32] Otero L., Vaz B., Alvarez R., de Lera A. R. (2013). Total synthesis of (8*R*,6’*R*)-peridinin-5,8-furanoxide. Chem.
Commun..

[33] Rivas A., Castiñeira M., Álvarez R., Vaz B., de Lera A. R. (2022). Stereoselective
Synthesis of Bisfuranoxide (Aurochrome, Auroxanthin) and Monofuranoxide
(Equinenone 5′,8′-Epoxide) Carotenoids by Double Horner–Wadsworth–Emmons
Reaction. J. Nat. Prod..

[34] Acemoglu M., Prewo R., Bieri J. H., Eugster C. H. (1984). (6′*RS*,8′*RS*,2*E*)- und
(6′*RS*,8′*SR*,2*E*)-3-Methyl-3-(2y,2′,6′-trimethyl-7′-oxabicyclo­[4.3.0]­non-9′-en-8′-yl)-2-propenal
([(5*RS*,8*RS*)- und (5*RS*,8*SR*)-5,8-Epoxy-5,8-dihydro-ionyloinden]­acetaldehyd):
Synthese und Röntgenstrukturanalyse. Helv. Chim. Acta.

[35] Acemoglu M., Eugster C. H. (1984). 5*R*,6*S*,5′*R*,6′*S*-5,6,5′,6′-Diepoxy-β,β-cartin:
Sythese, Spectroskopische, chiroptische und chromatographische Eigenschaften. Helv. Chim. Acta.

[36] Vaz B., Domínguez M., Álvarez A., de Lera A. R. (2007). Total Synthesis
of Perididin and Structurally Related C37-Norcarotenoid Butenolides. Chem.?Eur. J..

[37] Horner L. (1964). Darstellung
und Eigenschaften optisch aktiver, tertiärer Phosphine. Pure Appl. Chem..

[38] Wadsworth W. S. (1977). Synthetic
Applications of Phosphoryl-Stabilized Anions. Org. React..

[39] Nicolaou K. C., Härter M. W., Gunzner J. L., Nadin A. (1997). The Wittig and Related
Reactions in Natural Product Synthesis. Liebigs
Ann..

[40] Gu, Y. ; Tian, S.-K. Olefination Reactions of Phosphorus-Stabilized Carbon Nucleophiles. In Stereoselective Alkene Synthesis; Wang, J. , Ed.; Springer: Berlin, 2012. 10.1007/128_2012_314 22371171

[41] Kobayashi K., Tanaka K., Kogen H. (2018). Recent topics of the natural product
synthesis by Horner–Wadsworth–Emmons reaction. Tetrahedron Lett..

[42] Roman D., Sauer M., Beemelmanns C. (2021). Applications
of the Horner–Wadsworth–Emmons
Olefination in Modern Natural Product Synthesis. Synthesis.

[43] Miyaura N., Suzuki A. (1995). Palladium-Catalyzed
Cross-Coupling Reactions of Organoboron
Compounds. Chem. Rev..

[44] Suzuki A. (2005). Carbon-carbon
bonding made easily. Chem. Commun..

[45] Suzuki A. (2011). Cross-Coupling
Reactions Of Organoboranes: An Easy Way To Construct C-C Bonds (Nobel
Lecture). Angew. Chem., Int. Ed..

[46] Jin B., Liu Q., Sulikowski G. A. (2005). Development of an end-game strategy
towards apoptolidin: a sequential Suzuki coupling approach. Tetrahedron.

[47] Azim E.-M., Auzeloux P., Maurizis J.-C., Braesco V., Grolier P., Veyre A., Madelmont J.-C. (1996). Synthesis
of all-*trans*-β-carotene, retinoids and derivatives
labeled with ^14^C. J. Labelled Compd.
Radiopharm..

[48] Baumeler A., Eugster C. H. (1992). Synthese von (6*R*,all-*E*)-Neoxanthin und verwandten Allen-Carotinoiden. Helv. Chim. Acta.

[49] Acemoglu M., Eugster C. H. (1984). Die diasteromeren
Aurochrome: Synthese, Analytik und
Chiroptische Eigenschaften. Helv. Chim. Acta.

[50] Thompson S. K., Heathcock C. H. (1990). Effect of cation, temperature, and
solvent on the stereoselectivity
of the Horner-Emmons reaction of trimethyl phosphonoacetate with aldehydes. J. Org. Chem..

[51] Sicre C., Cid M. M. (2005). Convergent Stereoselective Synthesis of the Visual
Pigment A2E. Org. Lett..

[52] Fontán N., Alvarez R., de Lera A. R. (2012). Stereoselective Synthesis by Olefin
Metathesis and Characterization of η-Carotene (7,8,7’,8’-tetrahydro-β,β-carotene). J. Nat. Prod..

[53] Vaz B., Fontan N., Castineira M., Alvarez R., de Lera A. R. (2015). Synthesis
of labile all-trans-7,8,7’,8’-bis-acetylenic carotenoids
by bi-directional Horner-Wadsworth-Emmons condensation. Org. Biomol. Chem..

[54] The Wittig olefination reaction of 2-alkenylisonicotinaldehyde **20** with the phosphorane generated from the treatment of the corresponding C_15_-trienyl phosphonium salt (not shown) with *n*-BuLi at −78 °C afforded in high yields a mixture of the corresponding *E/Z* isomers of the tetraenylpyridine **31** with the reverse diastereomeric ratio (1:3).

[55] Yamano Y., Ito M. (2007). Total synthesis of
capsanthin and capsorubin using Lewis acid-promoted
regio- and stereoselective rearrangement of tetrasubsutituted epoxides. Org. Biomol. Chem..

[56] The pyridinium ion of enal intermediate **34** has been isolated as one of the photocleavage products of A2E (A2E-oxidation-7).^57^ We have also attempted the alkylation of **34** at 80 °C, but the compound underwent extensive decomposition under those conditions.

[57] Wang Z., Keller L. M. M., Dillon J., Gaillard E. R. (2006). Oxidation of A2E
Results in the Formation of Highly Reactive Aldehydes and Ketones. Photochem. Photobiol..

[58] Frisch, M. J. ; Trucks, G. W. ; Schlegel, H. B. ; Scuseria, G. E. ; Robb, M. A. ; Cheeseman, J. R. ; Scalmani, G. ; Barone, V. ; Petersson, G. A. ; Nakatsuji, H. ; Li, X. ; Caricato, M. ; Marenich, A. V. ; Bloino, J. ; Janesko, B. G. ; Gomperts, R. ; Mennucci, B. ; Hratchian, H. P. ; Ortiz, J. V. ; Izmaylov, A. F. ; Sonnenberg, J. L. ; Williams-Young, D. ; Ding, F. ; Lipparini, F. ; Egidi, F. ; Goings, J. ; Peng, B. ; Petrone, A. ; Henderson, T. ; Ranasinghe, D. ; Zakrzewski, V. G. ; Gao, J. ; Rega, N. ; Zheng, G. ; Liang, W. ; Hada, M. ; Ehara, M. ; Toyota, K. ; Fukuda, R. ; Hasegawa, J. ; Ishida, M. ; Nakajima, T. ; Honda, Y. ; Kitao, O. ; Nakai, H. ; Vreven, T. ; Throssell, K. J. A. ; Montgomery, J. ; Peralta, J. E. ; Ogliaro, F. ; Bearpark, M. J. ; Heyd, J. J. ; Brothers, E. N. ; Kudin, K. N. ; Staroverov, V. N. ; Keith, T. A. ; Kobayashi, R. ; Normand, J. ; Raghavachari, K. ; Rendell, A. P. ; Burant, J. C. ; Iyengar, S. S. ; Tomasi, J. ; Cossi, M. ; Millam, J. M. ; Klene, M. ; Adamo, C. ; Cammi, R. ; Ochterski, J. W. ; Martin, R. L. ; Morokuma, K. ; Farkas, O. ; Foresman, J. B. ; Fox, A. D. J. Gaussian 16, Revision C.01; Gaussian, Inc.: Wallingford CT, 2016.

[59] Chai J.-D., Head-Gordon M. (2008). Long-range
corrected hybrid density functionals with
damped atom-atom dispersion corrections. Phys.
Chem. Chem. Phys..

[60] Weigend F., Ahlrichs R. (2005). Balanced basis sets
of split valence, triple zeta valence
and quadruple zeta valence quality for H to Rn: Design and assessment
of accuracy. Phys. Chem. Chem. Phys..

[61] Tomasi J., Mennucci B., Cammi R. (2005). Quantum Mechanical Continuum Solvation
Models. Chem. Rev..

[62] Foster J. P., Weinhold F. (1980). Natural hybrid orbitals. J. Am.
Chem. Soc..

[63] Bulat F. A., Chamorro E., Fuentealba P., Toro-Labbé A. (2004). Condensation
of Frontier Molecular Orbital Fukui Functions. J. Phys. Chem. A.

[64] Sánchez-Márquez J., García V., Zorrilla D., Fernández M. (2020). On Electronegativity,
Hardness, and Reactivity Descriptors: A New Property-Oriented Basis
Set. J. Phys. Chem. A.

[65] Legault, C. Y. CYLview20, Université de Sherbroke, 2020. http://www.cylview.org.

[66] Zhao Y., Truhlar D. G. (2008). The M06 suite of density functionals
for main group
thermochemistry, thermochemical kinetics, noncovalent interactions,
excited states, and transition elements: two new functionals and systematic
testing of four M06-class functionals and 12 other functionals. Theor. Chem. Acc..

[67] Papajak E., Zheng J., Xu X., Leverentz H. R., Truhlar D. G. (2011). Perspectives on Basis Sets Beautiful:
Seasonal Plantings
of Diffuse Basis Functions. J. Chem. Theor.
Comput..

[68] Marenich A. V., Cramer C. J., Truhlar D. G. (2009). Universal
Solvation Model Based on
Solute Electron Density and on a Continuum Model of the Solvent Defined
by the Bulk Dielectric Constant and Atomic Surface Tensions. J. Phys. Chem. B.

[69] Dutra F. R., Silva C. d. S., Custodio R. (2021). On the Accuracy
of the Direct Method
to Calculate pKa from Electronic Structure Calculations. J. Phys. Chem. A.

[70] Geuenich D., Hess K., Köhler F., Herges R. (2005). Anisotropy of the Induced
Current Density (ACID), a General Method To Quantify and Visualize
Electronic Delocalization. Chem. Rev..

[71] Korevaar P. A., Schaefer C., de Greef T. F. A., Meijer E. W. (2012). Controlling Chemical
Self-Assembly by Solvent-Dependent Dynamics. J. Am. Chem. Soc..

[72] Chen T., Peng Y., Qiu M., Yi C., Xu Z. (2023). Recent advances
in mixing-induced nanoprecipitation: from creating complex nanostructures
to emerging applications beyond biomedicine. Nanoscale.

[73] Kuddushi M., Kanike C., Xu B. B., Zhang X. (2025). Recent advances in
nanoprecipitation: from mechanistic insights to applications in nanomaterial
synthesis. Soft Matter.

[74] Sparrow J. R., Wu Y., Nagasaki T., Yoon K. D., Yamamoto K., Zhou J. (2010). Fundus autofluorescence
and the bisretinoids of retina. Photochem. Photobiol.
Sci..

[75] Sparrow J. R., Wu Y., Kim C. Y., Zhou J. (2010). Phospholipid meets all-*trans*-retinal: the making
of RPE bisretinoids. J.
Lipid Res..

