# Supplementary material for: Enantiopure Pyridinium Bisretinoids of Ocular Lipofuscin with Hexahydrobenzofuran Structure: Total Synthesis and Structure-Dependent Aggregated Morphology
Source: J Org Chem. 2026 Jan 27;91(5):2141–51. doi: 10.1021/acs.joc.5c02843 (PMC12888001; doi:10.1021/acs.joc.5c02843)
Supplement: Supplementary file 1 [file jo5c02843_si_001.pdf]

Supporting information (S.I.)

Enantiopure Pyridinium Bisretinoids of Ocular Lipofuscin with Hexahydrobenzofuran Structure: Total Synthesis and Structure-Dependent Aggregated Morphology

Brais Vidal,[a] Rafael Rodríguez,[a] Angeles Peña-Gallego,[b] Rosana Álvarez,[a,*] Claudio Martínez,[a,*] and Ángel R. de Lera[a,*]

[a] CINBIO, Departamento de Química Orgánica, Universidade de Vigo, IBIV, As Lagoas-Marcosende, 36310 Vigo, Spain

[b] Departamento de Química Física, Universidade de Vigo, As Lagoas-Marcosende, 36310 Vigo, Spain

(qolera@uvigo.es; rar@uvigo.es; claudiom@uvigo.es)

Table of Contents

1. General remarks 2
2. Experimental procedures 4
3. TEM Images 27
4. Comparison of the ^{1}H-NMR spectra of **9a** at different CD_3OD/D_2O ratios 29
5. Additional UV-Vis spectra of **9a** and analogues 30
6. DFT-simulation of pKa values 31
7. DFT studies of the epoxycyclohexanedienylphosphonate to hexahydrobenzofuran alkenylphosphonate rearrangement under basic conditions 33
8. DFT studies of the rearrangement of 4-epoxycyclohexanetrienylpyridine **40** under acidic conditions 35
9. References 37
10. Copies of NMR spectra 38

1. General remarks

Solvents were dried using a Puresolv^{TM} solvent purification system. All other reagents were commercial compounds of the highest purity available. If not specified, all reactions were carried out under an argon atmosphere. Those not involving aqueous reagents were carried out in oven dried glassware. For reactions that require heating, a metallic heating block was used and the indicated is the external temperature. All solvents and anhydrous solutions were transferred through syringes and cannulas previously dried in the oven for at least 12h and kept in a desiccator. Analytical TLC was performed on aluminium plates with Merck Kieselgel 60F$_{254}$ and visualized by UV irradiation (254 nm) or by staining with a solution of phosphomolybdic acid in ethanol. Flash column chromatography was carried out using Merck Kieselgel 60 (230–400 mesh) with a CombiFlash® Rf Teledyne Isco. HPLC separations were carried out using a Waters 1525 Binary Pump and a Waters 2487 Dual Absorbance Detector, and a Chiralpak® IA column, 4 µm, 250 x 20 mm.

HRMS (ESI$^+$) were measured with an FT-ICR-MS Solarix 7T mass spectrometer (Bruker Daltonics). ^{1}H-NMR spectra were recorded in C_6D_6, CD_2Cl_2 and CD_3OD at 298 K with a Bruker AMX-400 spectrometer at 400.16 MHz with residual protic solvent as the internal reference [C_6D_6, δ= 7.16 ppm; CD_2Cl_2, δ= 5.32 ppm; CD_3OD = 3.31 ppm]; chemical shifts (δ) are given in parts per million (ppm) and coupling constants (*J*) are given in Hertz (Hz). The proton spectra are reported as follows: δ (multiplicity, coupling constant *J*, number of protons). ^{13}C-NMR spectra were recorded in C_6D_6, CD_2Cl_2 and CD_3OD at 298 K with the same spectrometer operating at 100.63 MHz with the central peak of C_6D_6 (δ = 128.06 ppm), CD_2Cl_2 (δ = 53.84 ppm and CD_3OD (δ = 49.0 ppm) as the internal reference. DEPT-135 pulse sequences and HSQC bidimensional NMR spectra were used to aid in the assignment of signals in the ^{13}C- and ^{1}H-NMR spectra. Multiplicity and ^{13}C coupling constants are indicated in parenthesis after the ^{13}C-^{1}H multiplicity. Different NOE-1D experiments were also performed in selected cases. UV/Vis spectra were recorded on a Cary 100 Bio spectrophotometer. Infrared spectra (IR) were obtained on a JASCO FT/IR-4200 infrared spectrometer, from a thin film deposited onto a NaCl glass. IR data include only characteristic absorptions. Peaks are quoted in wave numbers (cm^{-1}), and their relative intensities are reported as follows: s = strong, m = medium, w = weak. Specific

optical rotations were measured on a JASCO P-1020 polarimeter with a Na lamp (glass cell, 3.5 x 100 mm). Transmission electron images (TEM) were carried out on a thermionic JEOL JEM1010 working at 100 kV. Samples were prepared by dropping the solutions onto a 400-mesh copper grid coated with formvar and carbon. Fluorescence emission spectra were measured using a Jasco fp-8550 spectrofluorometer with the excitation wavelength (λ_{ex}) at 430 nm.

2. Experimental procedures

Optimization of the HWE condensation reaction

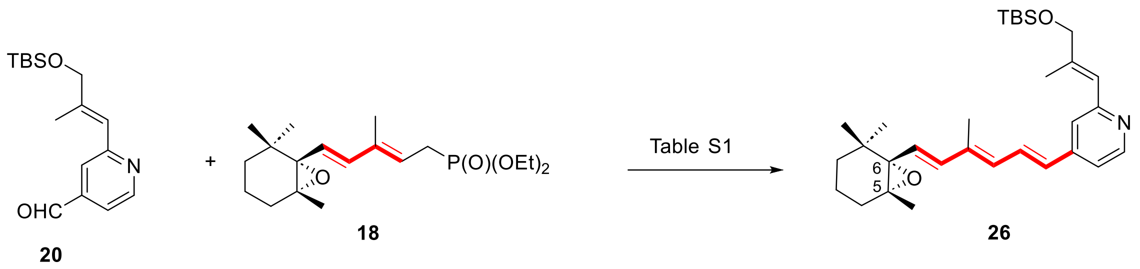

Table S1. Optimization of the Horner-Wadsworth-Emmons olefination of pyridine carbaldehyde **20** and phosphonate **18** (Scheme 2A).

Entry	Reaction conditions	Yield of 26(%)
**1**	**18** (1.3 equiv.), KO^{t}Bu (1.2 equiv.), THF, -30 to 0 to 25 °C, 24h[1-3]	53
**2**	**18** (1.7 equiv.), NaHMDS (1.6 equiv.), THF, -30 to 0 to 25 °C, 24h	83
**3**	**18** (1.7 equiv.), LDA (1.4 equiv.), THF, -30 to 0 to 25 °C, 24h	46
**4**	**18** (1.5 equiv.), *n*-BuLi (1.45 equiv.), THF, -30 to 0 to 25 °C, 24h	Decomposition
**5**	**18** (1.5 equiv.), *n*-BuLi (1.45 equiv.), DMPU, THF, -30 to 0 to 25 °C, 24h	44

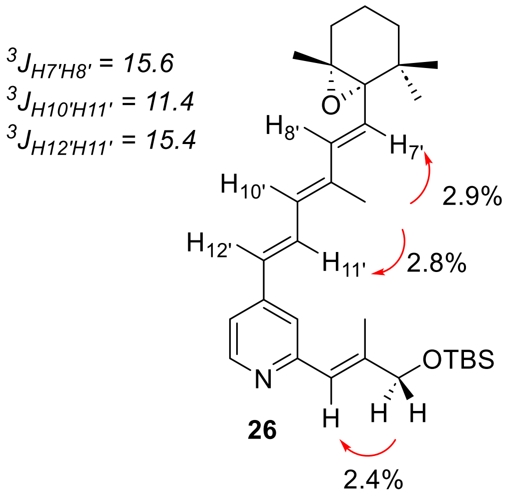

Figure S1. Coupling constants ($^3J_{H\text{-}H}$, values in Hz) of compound **26** and nuclear Overhauser effects (NOE, red) observed in ^{1}H-NMR experiments (400.16 MHz, CD_2Cl_2).

(*E*)-*tert*-Butyldimethyl((2-methyl-3-(4,4,5,5-tetramethyl-1,3,2-dioxaborolan-2-yl)allyl)oxy)silane 24.

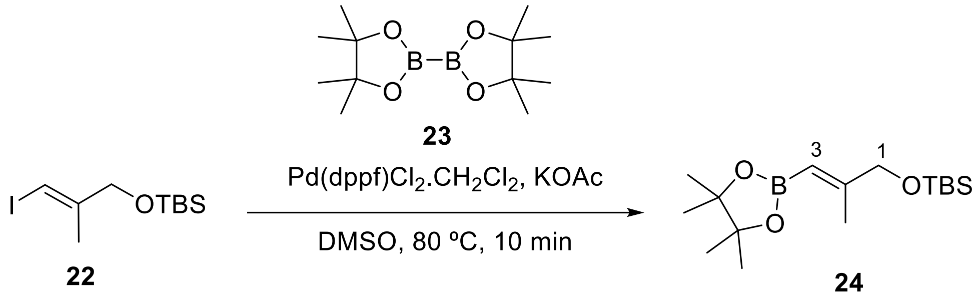

To a mixture of Pd(dppf)Cl_2·CH_2Cl_2 (0.08 g, 0.1 mmol), KOAc (0.94 g, 9.6 mmol) and bis(pinacolato)diboron **23** (2.4 g, 9.6 mmol), (*E*)-*tert*-butyl-(3-iodo-2-methylallyl)oxy-dimethylsilane **22** (1.0 g, 3.2 mmol) in DMSO (11 mL) was added and the solution was stirred at 80 °C for 10 min. The reaction mixture was cooled down to room temperature and then extracted with Et_2O (3x). The combined organic layers were washed with brine, dried over Na_2SO_4, filtered and the solvent was evaporated. The residue was purified by flash column chromatography (silica gel, from 100:0 to 90:10 (v/v) *n*-hexane/EtOAc) to afford 0.8 g (78% yield) of a colourless oil, which was identified as (*E*)-*tert*-butyldimethyl((2-methyl-3-(4,4,5,5-tetramethyl-1,3,2-dioxaborolan-2-yl)allyl)oxy)silane **24**. **^{1}H-NMR** (400.16 MHz, C_6D_6): δ 6.02 (s, 1H, H_3), 4.00 (s, 2H, $2H_1$), 2.10 (s, 3H, CH_3), 1.09 (s, 12H, 2xO-C(CH_3)$_2$), 0.95 (s, 9H, SiC(CH_3)$_3$), 0.03 (s, 6H, 2xSi-CH_3) ppm. **^{13}C{^{1}H}-NMR** (100.63 MHz, C_6D_6): δ 160.2, 111.0, 82.6, 68.7, 26.1 (3x), 25.0 (4x), 18.6 (2x), 17.8, -5.3 (2x) ppm. **IR** (NaCl): υ 2978 (w, C-H) 2956 (w, C-H), 2930 (w, C-H), 2857 (w, C-H), 1650 (m, C=C), 1335 (s, B-O), 1258 (m, C-O), 1115 (s, Si-O-C) cm^{-1}. **HRMS** (ESI$^+$): calcd. for $C_{16}H_{33}BO_3SiNa$ ([M+Na]$^+$), 335.2190; found, 335.2186.

(*E*)-2-(3-((*tert*-Butyldimethylsilyl)oxy)-2-methylprop-1-en-1-yl)isonicotinaldehyde 20.

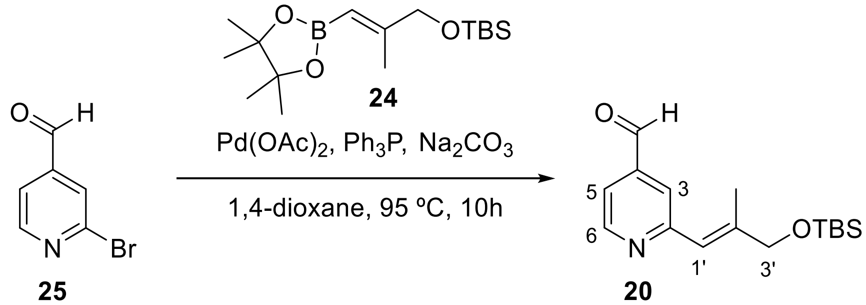

$Pd(OAc)_2$ (24.1 mg, 0.11 mmol), PPh_3 (70.5 mg, 0.27 mmol) and 2-bromoisonicotinaldehyde **25** (0.2 g, 1.07 mmol) were added to a sealing tube. Then, compound **24** (0.67 g, 2.15 mmol) in 1,4-dioxane (10 mL) and Na_2CO_3 (1.1 mL, 2M, 2.15 mmol) were added and the tube was sealed. The reaction mixture was stirred at 95 °C for 10h. The temperature was cooled down to room temperature, and the mixture was extracted with CH_2Cl_2 (3x). The combined organic layers were dried over Na_2SO_4, filtered and the solvent was evaporated. The residue was purified by flash column chromatography (silica gel, 98:2 (v/v) *n*-hexane/Et_3N; then, from 100:0 to 90:10 (v/v) *n*-hexane/EtOAc) to afford 0.30 g (96% yield) of a white solid, which was identified as (*E*)-2-(3-((*tert*-butyldimethylsilyl)oxy)-2-methylprop-1-en-1-yl)isonicotinaldehyde **20**. **^{1}H-NMR** (400.16 MHz, C_6D_6): δ 9.33 (s, 1H, CHO), 8.51 (d, *J* = 4.8 Hz, 1H, H_6), 7.18 (s, 1H, H_3), 6.87 (d, *J* = 4.8 Hz, 1H, H_5), 6.81 (s, 1H, $H_{1'}$), 4.07 (s, 2H, 2x$H_{3'}$), 2.17 (s, 3H, CH_3), 1.02 (s, 9H, $SiC(CH_3)_3$), 0.10 (s, 6H, 2xSi-CH_3) ppm. **^{13}C{^{1}H}-NMR** (100.63 MHz, C_6D_6): δ 191.2, 159.2, 150.5, 144.6, 142.1, 123.1, 122.1, 118.4, 68.4, 26.1 (3x), 18.6, 15.8, -5.2 (2x) ppm. **IR** (NaCl): υ 2927 (m, C-H), 2856 (m, C-H), 1705 (s, C=O), 1461 (w, C=C), 1255 (m, C-O), 1105 (s, Si-O-C) cm^{-1}. **HRMS** (ESI$^+$): calcd. for $C_{16}H_{25}NO_2SiNa$ ($[M+Na]^+$), 314.1552; found, 314.1550.

Compound 26.

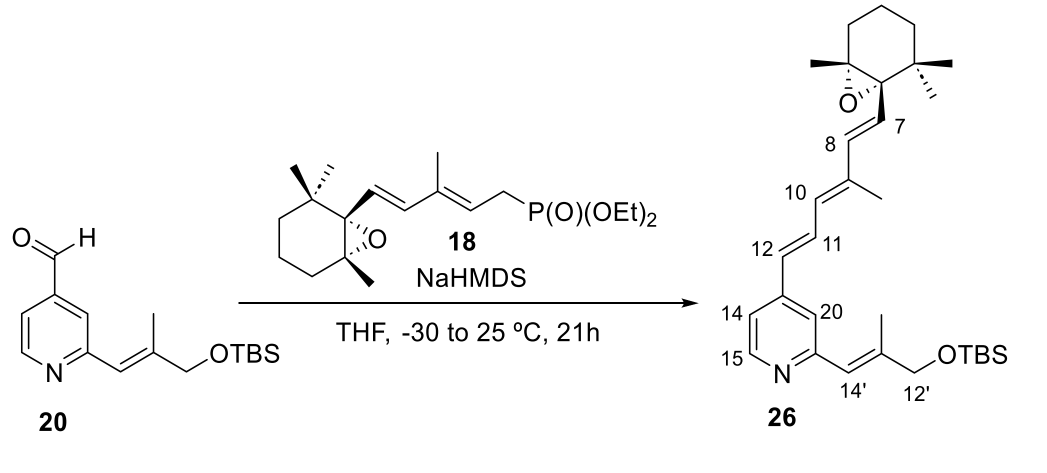

To a cooled (-30 °C) stirred solution of phosphonate **18** (41.6 mg, 0.12 mmol) in THF (3 mL), NaHMDS (0.11 mL, 1M in THF, 0.11 mmol) was added. After 30 min, compound **20** (20.0 mg, 0.07 mmol) in THF (3 mL) was added at 0 °C and the mixture was allowed to warm up to room temperature for 21h. A saturated aqueous solution of NH_4Cl was added and the mixture was extracted with CH_2Cl_2 (3x). The combined organic layers

were washed with a saturated aqueous solution of $NaHCO_3$, dried over Na_2SO_4, filtered and the solvent was evaporated. The residue was purified by flash column chromatography (silica gel, 98:2 (v/v) *n*-hexane/Et_3N; then, from 100:0 to 90:10 (v/v) *n*-hexane/EtOAc) to afford 28 mg (83% yield) of a yellow oil, which was identified as **26**. **$[\alpha]_D^{23}$** -10.51 (*c* 0.49, CH_2Cl_2). **^{1}H-NMR** (400.16 MHz, CD_2Cl_2): δ 8.47 (d, *J* = 5.2 Hz, 1H, H_{15}), 7.35 (dd, *J* = 15.4, 11.3 Hz, 1H, H_{11}), 7.18 (s, 1H, H_{20}), 7.11 (d, *J* = 5.2 Hz, 1H, H_{14}), 6.55 (s, 1H, $H_{14'}$), 6.51 (d, *J* = 15.4 Hz, 1H, H_{12}), 6.33 (d, *J* = 15.6 Hz, 1H, H_8), 6.26 (d, *J* = 11.4 Hz, 1H, H_{10}), 6.04 (d, *J* = 15.6 Hz, 1H, H_7), 4.20 (s, 2H, 2x$H_{12'}$), 2.06 (s, 3H, CH_3), 2.02 (s, 3H, CH_3), 1.91 - 1.71 (m, 2H, CH_2), 1.47 – 1.38 (m, 2H, CH_2), 1.31 – 1.19 (m, 2H, CH_2), 1.13 (s, 3H, CH_3), 1.11 (s, 3H, CH_3), 0.96 (s, 9H, $SiC(CH_3)_3$), 0.92 (s, 3H, CH_3), 0.13 (s, 6H, 2xSi-CH_3) ppm. **^{13}C{^{1}H}-NMR** (100.63 MHz, CD_2Cl_2): δ 158.0, 149.7, 145.3, 142.8, 138.3, 137.1, 130.6, 130.3, 129.5, 127.0, 123.0, 121.7, 117.8, 71.5, 68.6, 65.8, 36.2, 34.1, 30.5, 26.1 (4x), 26.0, 21.2, 18.7, 17.5, 15.5, 13.3, -5.2 (2x) ppm. **IR** (NaCl): υ 2954 (s, C-H), 2928 (s, C-H), 2855 (m, C-H), 1461 (m, C=C), 1252 (m, C-O), 1080 (m, Si-O-C) cm^{-1}. **HRMS** (ESI^+): calcd. for $C_{31}H_{48}NO_2Si$ ($[M+H]^+$) 494.3454; found, 494.3454.

Compounds (5*R*,8*R*)-28 and (5*R*,8*S*)-28.

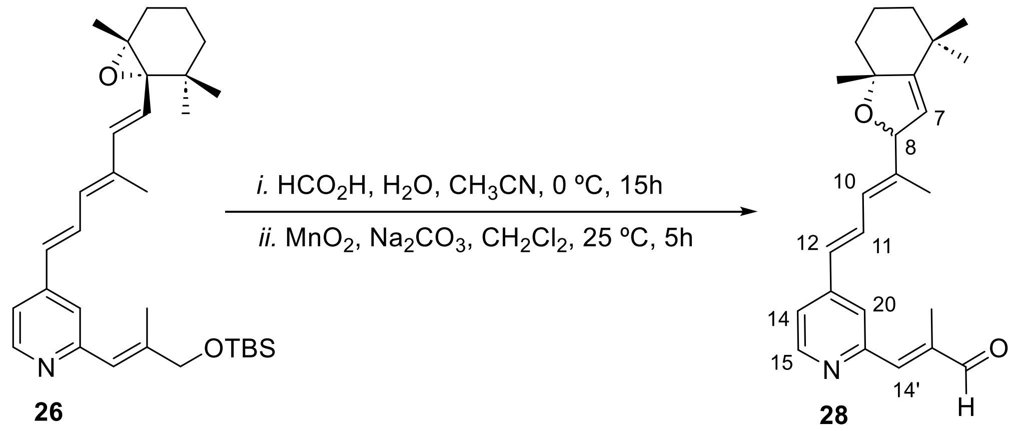

A cooled (0 °C) mixture of HCO_2H (1.5 mL, 38.64 mmol) and H_2O (0.45 mL, 25.28 mmol) was added to a cooled (0 °C) flask containing a solution of **26** (50.3 mg, 0.92 mmol) in CH_3CN (3 mL). After stirring the resulting mixture at 0 °C for 15h, a saturated aqueous solution of $NaHCO_3$ was added, and the mixture was extracted with EtOAc (3x). The combined organic layers were dried over Na_2SO_4, filtered and the solvent was evaporated.

To a solution of the crude obtained above (30.5 mg, 0.03 mmol) in CH_2Cl_2 (3 mL) at room temperature, Na_2CO_3 (0.18 g, 1.49 mmol) and MnO_2 (0.15 g, 1.49 mmol) were added. The resulting reaction mixture was stirred at room temperature for 15h. The mixture was filtered through Celite® washing with CH_2Cl_2 to afford 24.1 mg (80% yield) of a dark red oil, which was identified as a 1:5 mixture of the 5*R*,8*S*- and 5*R*,8*R*-diastereoisomers **28**. **^{1}H-NMR** (400.16 MHz, C_6D_6) (representative signals for the mixture of diastereomers): δ 9.47 (s, 1H, CHO), 8.45 (d, *J* = 5.1 Hz, 1H, H_{15}), 7. 18 – 7.10 (m, 1H, H_{11}), 6.94 (s, 1H, H_{20}), 6.73 (d, *J* = 1.5 Hz, 1H, $H_{14'}$), 6.67 (dd, *J* = 5.1, 1.7 Hz, 1H, H_{14}), 6.42 (dd, *J* = 11.1, 1.3 Hz, 1H, H_{10}), 6.20 (d, *J* = 15.5 Hz, 1H, H_{12}), 5.28 (s, 1H, H_7), 5.22 – 5.18 (m, 2H, H_7 + H_8), 5.12 (s, 1H, H_8), 2.48 (d, *J* = 1.4 Hz, 3H, CH_3), 2.05 – 2.00 (m, 1H, H_{4A}), 1.82 (d, *J* = 1.3 Hz, 3H, CH_3), 1.75 – 1.65 (m, 1H, H_{4B}), 1.49 (d, *J* = 1.0 Hz, 3H, CH_3), 1.48 – 1.41 (m, 2H, CH_2), 1.38 – 1.28 (m, 2H, CH_2), 1.06 (s, 3H, CH_3), 0.99 (s, 3H, CH_3) ppm.

Compounds (5*R*,8*R*)-29a and (5*R*,8*S*)-29b.

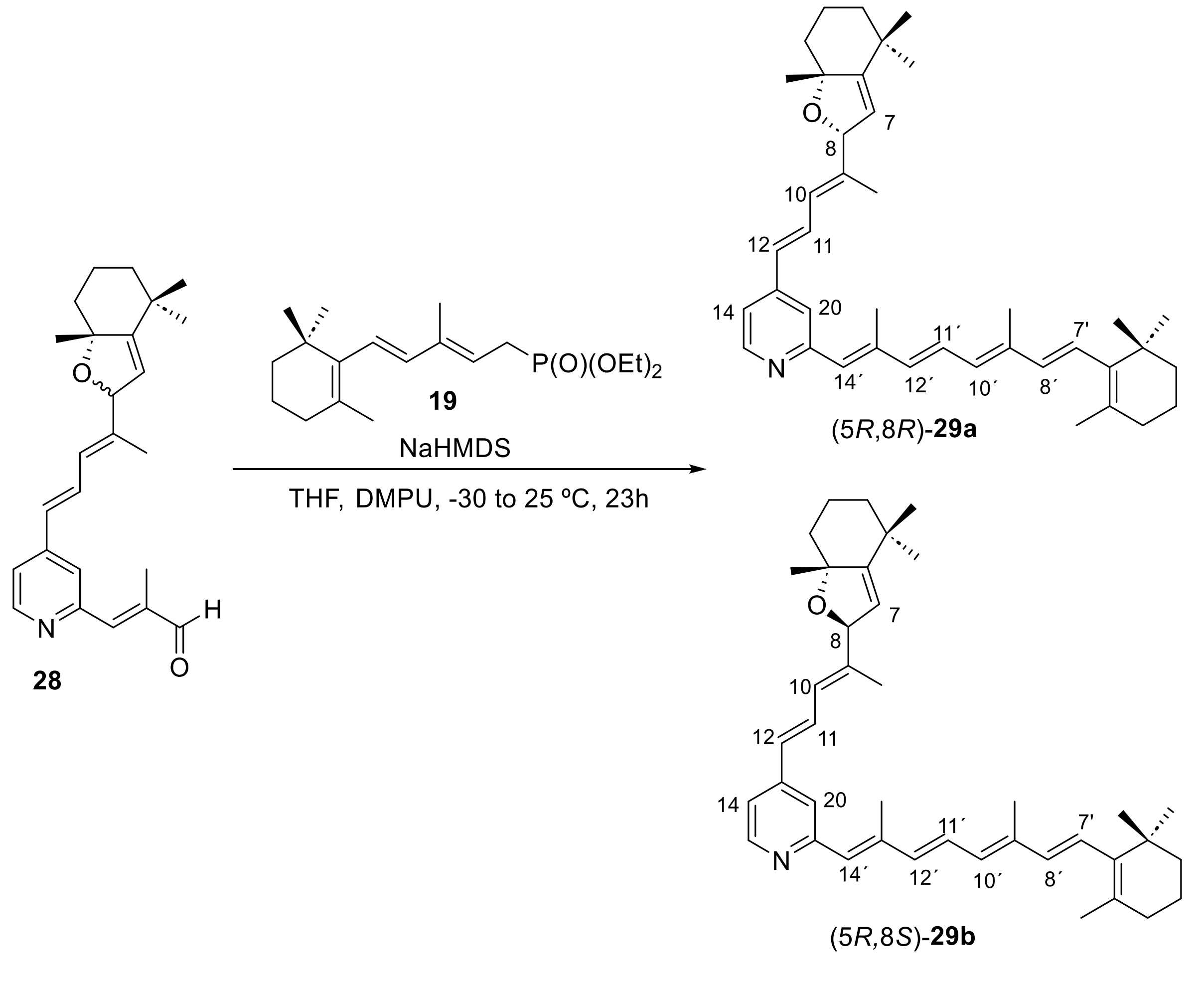

To a cooled (-30 °C) stirred solution of phosphonate **19** (36.9 mg, 0.11 mmol) in THF (0.3 mL), DMPU (0.16 mL, 1.32 mmol) and NaHMDS (0.10 mL, 1M in THF, 0.10 mmol) were added. Subsequently, **28** (24.1 mg, 0.06 mmol) in THF (0.3 mL) was added and the mixture was allowed to warm up to room temperature for 23h. A saturated aqueous solution of NH_4Cl was added and the mixture was extracted with CH_2Cl_2 (3x). The combined organic layers were washed with $NaHCO_3$, dried over Na_2SO_4, filtered and the solvent was evaporated. The residue was purified by flash column chromatography (CN silica gel, 98:2 (v/v) *n*-hexane/CH_2Cl_2; then, from 95:2.5:2.5 to 60:20:20 (v/v) *n*-hexane/EtOAc/CH_2Cl_2) to afford 26.1 mg (73% yield) of a dark red foam, which was identified as a 5:1 mixture of (5*R*,8*R*)-**29a** and (5*R*,8*S*)-**29b** diastereoisomers. This mixture was further purified by HPLC (Chiralpak® IA column, Hept/CH_2Cl_2/EtOH/Et_2NH 70:30:1:0.1 v/v/v/v, 1 mL/min; detection at 347 and 245 nm) to provide the expected pure products.

Data for (5*R*,8*R*)-**29a**: **$[\alpha]_D^{23}$** +20.19 (*c* 0.02, CH_2Cl_2). **UV** (CH_2Cl_2): λ_{max} 328 nm (ε = 33,300), 377 nm (ε = 35,200). **^{1}H-NMR** (400.16 MHz, CD_2Cl_2): δ 8.49 (d, *J* = 5.1 Hz, 1H, H_{15}), 7.26 (dd, *J* = 15.1, 11.3 Hz, 1H, H_{11}), 7.21 (s, 1H, H_{20}), 7.09 (dd, *J* = 5.1, 1.7 Hz, 1H, H_{14}), 6.86 (dd, *J* = 15.1, 11.3 Hz, 1H, $H_{11'}$), 6.51 – 6.44 (m, 3H, $H_{14'}$ + $H_{12'}$ + H_{12}), 6.29 (d, *J* = 11.2 Hz, 1H, H_{10}), 6.23 (d, *J* = 16.1 Hz, 1H, $H_{7'}$), 6.20 (d, *J* = 11.1 Hz, 1H, $H_{10'}$), 6.15 (d, *J* = 16.1 Hz, 1H, $H_{8'}$), 5.22 (s, 1H, H_7), 5.15 (s, 1H, H_8), 2.38 (d, *J* = 1.1 Hz, 3H, CH_3), 2.04 (t, *J* = 6.3 Hz, 2H, CH_2), 2.00 (d, *J* = 1.1 Hz, 3H, CH_3), 1.98 – 1.91 (m, 1H, H_{4A}), 1.82 (d, *J* = 1.3 Hz, 3H, CH_3), 1.73 (d, *J* = 1.0 Hz, 3H, CH_3), 1.66 – 1.58 (m, 4H, 2xCH_2), 1.56 – 1.47 (m, 5H, H_{4B} + 2xCH_2), 1.42 (s, 3H, CH_3), 1.16 (s, 3H, CH_3), 1.11 (s, 3H, CH_3), 1.04 (s, 6H, 2xCH_3) ppm. **^{13}C{^{1}H}-NMR** (100.63 MHz, CD_2Cl_2): δ 157.9, 155.3, 149.8, 145.4, 143.5, 141.0, 138.2, 138.2, 138.1, 137.3, 130.7, 130.4, 129.9, 129.8, 129.5, 127.6, 127.2, 125.4, 122.6, 119.0, 117.9, 88.1, 87.5, 41.8, 41.7, 40.1, 34.9, 34.6, 33.5, 30.8, 29.1 (2x), 26.2, 26.1, 21.9, 20.8, 19.7, 14.2, 13.2, 12.9 ppm. **HRMS** (ESI^+): calcd. for $C_{40}H_{54}NO$ ($[M+H]^+$) 564.4205; found, 564.4195. **UV** (CH_2Cl_2): λ_{max} 328 nm (ε = 33,333), 377 nm (ε = 35,161).

Data for (5*R*,8*S*)-**29b**: **^{1}H-NMR** (400.16 MHz, CD_2Cl_2): δ 8.49 (d, *J* = 5.2 Hz, 1H, H_{15}), 7.26 (dd, *J* = 15.4, 11.2 Hz, 1H, H_{11}), 7.23 – 7.18 (m, 2H, $H_{14'}$ + H_{20}), 7.09 (d, *J* = 5.2 Hz, 1H, H_{14}), 6.86 (dd, *J* = 15.1, 11.3 Hz, 1H, $H_{11'}$), 6.54 – 6.43 (m, 2H, H_{12} + $H_{12'}$), 6.29 (d, *J* = 11.2 Hz, 1H, H_{10}), 6.23 (d, *J* = 16.1 Hz, 1H, $H_{7'}$), 6.19 (d, *J* = 11.3 Hz, 1H, $H_{10'}$), 6.13 (d, *J* = 16.1 Hz,

1H, $H_{8'}$), 5.29 (d, *J* = 2.0 Hz, 1H, H_7), 5.06 (s, 1H, H_8), 2.38 (d, *J* = 1.0 Hz, 3H, CH_3), 2.03 (t, *J* = 6.6 Hz, 2H, CH_2), 2.00 (s, 3H, CH_3), 1.91 – 1.88 (m, 1H, H_{4A}), 1.87 (d, *J* = 1.3 Hz, 3H, CH_3), 1.73 (d, *J* = 0.9 Hz, 3H, CH_3), 1.65 – 1.60 (m, 4H, 2xCH_2), 1.56 – 1.47 (m, 3H, H_{4A} + CH_2), 1.46 (s, 3H, CH_3), 1.33 – 1.22 (m, 2H, CH_2), 1.18 (s, 3H, CH_3), 1.11 (s, 3H, CH_3), 1.04 (s, 6H, 2xCH_3) ppm. **$^{13}C\{^1H\}$-NMR** (100.63 MHz, CD_2Cl_2): δ 157.8, 154.4, 149.7, 145.5, 144.1, 138.2, 138.1, 138.0, 137.3, 130.7, 130.4, 129.9, 129.6 (2x), 129.5, 129.2, 127.6, 127.2, 124.3, 122.5, 117.9, 88.5, 88.0, 42.4, 41.9, 40.1, 35.4, 34.6, 33.5, 30.8, 29.1 (2x), 27.8, 25.7, 21.9, 21.1, 19.7, 14.2, 13.8, 12.9 ppm. **HRMS** (ESI^+): calcd. for $C_{40}H_{54}NO$ ($[M+H]^+$) 564.4205; found, 564.4195.

(5*R*,8*R*)-S-Monofuran-A2E (8a).

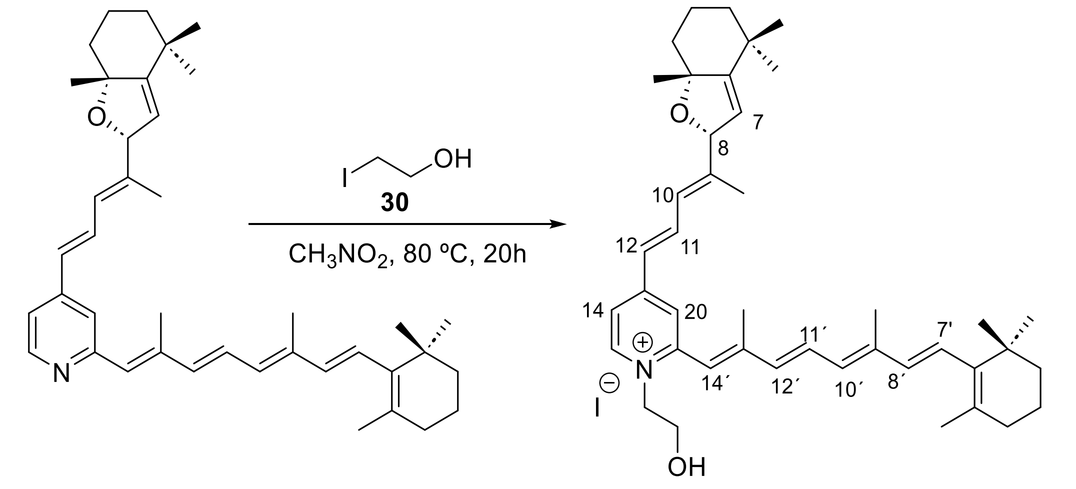

29a

8a, (5*R*,8*R*)-S-monofuran-A2E

To a solution of (5*R*,8*R*)-**29a** (4 mg, 0.01 mmol) in CH_3NO_2 (0.1 mL), 2-iodoethanol **30** (0.01 mL, 0.07 mmol) was added. The resulting solution was heated at 80 °C for 20h. The solvent was evaporated under vacuum, the residue was triturated with *n*-hexane and Et_2O mixtures and the solvents were removed to afford 2.9 mg (67% yield) of a dark red solid, which was identified as (5*R*,8*R*)-S-monofuran-A2E (**8a**). **$[\alpha]_D$**^{23} +61.00 (*c* 0.06, CH_3OH). **UV** (CH_3OH): λ_{max} 348 nm (ε = 20,000), 357 nm (ε = 20,000), 425 nm (ε = 9600). **^1H-NMR** (400.16 MHz, CD_3OD): δ 8.58 (d, *J* = 6.8 Hz, 1H, H_{15}), 7.96 (dd, *J* = 6.8, 2.1 Hz, 1H, H_{14}), 7.91 (d, *J* = 2.1 Hz, 1H, H_{20}), 7.85 (dd, *J* = 15.4, 11.2 Hz, 1H, H_{11}), 7.13 (dd, *J* = 15.0, 11.4 Hz, 1H, $H_{11'}$), 6.78 (d, *J* = 15.4 Hz, 1H, H_{12}), 6.71 (s, 1H, $H_{14'}$), 6.62 (d, *J* = 15.1 Hz, 1H, $H_{12'}$), 6.46 (d, *J* = 11.3 Hz, 1H, H_{10}), 6.35 (d, *J* = 16.1 Hz, 1H, H), 6.25 (d, *J* = 11.3 Hz, 1H, $H_{10'}$), 6.18 (d, *J* = 16.0 Hz, 1H), 5.31 (s, 1H, H_7), 5.24 (s, 1H, H_8), 4.58 – 4.55 (m,

2H, CH_2), 3.92 (t, *J* = 5.0 Hz, 2H, CH_2), 2.15 (d, *J* = 1.1 Hz, 3H, CH_3), 2.08 – 2.02 (m, 5H, CH_2 + CH_3), 2.00 – 1.92 (m, 5H, CH_2 + CH_3), 1.73 (d, *J* = 1.0 Hz, 3H, CH_3), 1.70 – 1.63 (m, 4H, 2xCH_2), 1.62 – 1.55 (m, 2H, CH_2), 1.53 – 1.48 (m, 2H, CH_2), 1.47 (s, 3H, CH_3), 1.19 (s, 3H, CH_3), 1.15 (s, 3H, CH_3), 1.05 (s, 6H, 2xCH_3) ppm. **^{13}C{^{1}H}-NMR** (100.63 MHz, CD_3OD): δ 156.3, 154.8, 153.7, 150.7, 149.1, 146.3, 141.0, 139.1, 138.9, 138.8, 135.5, 132.2, 130.9, 130.8, 129.9, 127.8, 127.5, 126.3, 121.5, 120.3, 119.4, 89.8, 88.4, 61.0, 60.2, 42.5, 42.4, 40.8, 35.7, 35.3, 34.0, 31.1, 29.4 (2x), 26.4 (2x), 21.9, 21.3, 20.3, 15.0, 13.7, 12.9 ppm. **HRMS** (ESI$^+$): calcd. for $C_{42}H_{58}NO_2^+$ ([M$^+$]) 608.4462; found, 608.4461.

Compounds (11*E*)-31 and (11*Z*)-32.

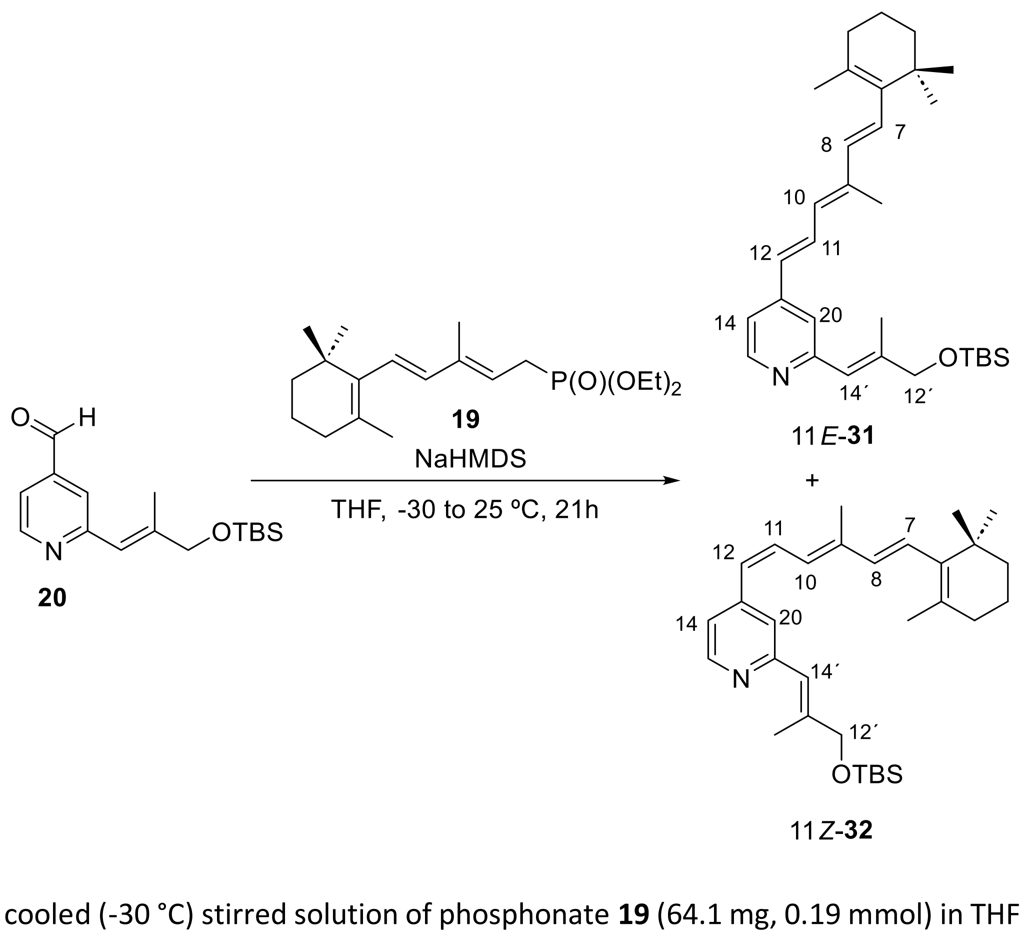

To a cooled (-30 °C) stirred solution of phosphonate **19** (64.1 mg, 0.19 mmol) in THF (0.5 mL), NaHMDS (0.18 mL, 1M in THF, 0.18 mmol) was added. Subsequently, **20** (32.3 mg, 0.11 mmol) in THF (0.5 mL) was added at -30 °C and the mixture was stirred for 21h at room temperature. A saturated aqueous solution of NH_4Cl was added and the mixture was extracted with CH_2Cl_2 (3x). The combined organic layers were washed with a saturated aqueous solution of $NaHCO_3$, dried over Na_2SO_4, filtered and the solvent was evaporated. The residue was purified by flash column chromatography (silica gel, 98:2

(v/v) *n*-hexane/Et_3N; then, from 100:0 to 90:10 (v/v) *n*-hexane/EtOAc) to afford 30 mg (60% yield) of a dark yellow oil, which was identified as 11*E*-**31** and 10 mg (19% yield) of a dark yellow oil, which was identified as 11*Z*-**32**.

Data for (11*E*)-**31:** ^{1}**H-NMR** (400.16 MHz, C_6D_6): δ 8.58 (d, *J* = 5.1 Hz, 1H, H_{15}), 7.22 (dd, *J* = 15.3, 11.3 Hz, 1H, H_{11}), 7.12 (s, 1H, H_{20}), 6.87 (s, 1H, $H_{14'}$), 6.75 (d, *J* = 5.1 Hz, 1H, H_{14}), 6.37 (d, *J* = 16.3 Hz, 1H, H_8), 6.31 (d, *J* = 16.3 Hz, 1H, H_7), 6.23 (d, *J* = 15.6 Hz, 1H, H_{12}), 6.17 (d, *J* = 12.3 Hz, 1H, H_{10}), 4.15 (s, 2H, $2H_{12'}$), 2.34 (s, 3H, CH_3), 1.97 (t, *J* = 6.1 Hz, 2H, CH_2), 1.84 (s, 3H, CH_3), 1.79 (s, 3H, CH_3), 1.65 - 1.55 (m, 2H, CH_2), 1.51 - 1.46 (m, 2H, CH_2), 1.14 (s, 6H, $2xCH_3$), 1.03 (s, 9H, $SiC(CH_3)_3$), 0.11 (s, 6H, 2xSi-CH_3) ppm. ^{13}**C{^{1}H}-NMR** (100.63 MHz, C_6D_6): δ 158.4, 149.9, 145.1, 142.9, 138.7, 138.2, 138.2, 130.4, 130.3, 129.9, 129.1, 128.5, 123.3, 122.0, 117.5, 68.8, 39.9, 34.6, 33.3, 29.2 (2x), 26.2 (3x), 22.0, 19.7, 18.7, 15.9, 12.9, -5.1 (2x) ppm. **IR** (NaCl): υ 2954 (s, C-H), 2929 (s, C-H), 2857 (m, C-H), 1587 (m, C=C), 1253 (m, C-O), 1108 (m, Si-O-C) cm^{-1}. **HRMS** (ESI$^+$): calcd. for $C_{31}H_{48}NOSi$ ([M+H]$^+$) 478.3505; found, 478.3499.

Data for (11*Z*)-**32:** ^{1}**H-NMR** (400.16 MHz, C_6D_6): 8.55 (d, *J* = 5.1 Hz, 1H, H_{15}), 7.21 (s, 1H, H_{20}), 6.86 (s, 1H, $H_{14'}$), 6.80 (d, *J* = 5.1 Hz, 1H, H_{14}), 6.72 (d, *J* = 12.0 Hz, 1H, H_{12}), 6.58 (t, *J* = 12.0 Hz, 1H, H_{11}), 6.32 (d, *J* = 16.1 Hz, 1H, H_8), 6.19 (d, *J* = 16.1 Hz, 1H, H_7), 6.14 (d, *J* = 12.0 Hz, 1H, H_{10}), 4.09 (s, 2H, $2xH_{12'}$), 2.22 (s, 3H, CH_3), 1.94 (t, *J* = 6.5 Hz, 2H, CH_2), 1.83 (s, 3H, CH_3), 1.69 (s, 3H, CH_3), 1.60 – 1.54 (m, 2H, CH_2), 1.48 – 143 (m, 2H, CH_2), 1.08 (s, 6H, $2xCH_3$), 1.01 (s, 9H, $SiC(CH_3)_3$), 0.08 (s, 6H, 2xSi-CH_3) ppm. ^{13}**C{^{1}H}-NMR** (100.63 MHz, C_6D_6): δ 158.0, 149.8, 145.2, 142.7, 139.9, 138.3, 138.0, 129.7, 129.5, 128.7, 127.4, 125.9, 124.5, 123.6, 120.9, 68.7, 39.9, 34.5, 33.2, 29.2, 26.2 (2x), 21.9 (3x), 19.7, 18.6, 15.9, 12.6, -5.1 (2x) ppm. **IR** (NaCl): υ 2954 (s, C-H), 2926 (s, C-H), 2856 (m, C-H), 1589 (m, C=C), 1253 (m, C-O), 1110 (m, Si-O-C) cm^{-1}. **HRMS** (ESI$^+$): calcd. for $C_{31}H_{48}NOSi$ ([M+H]$^+$) 478.3505; found, 478.3504.

Compound 33.

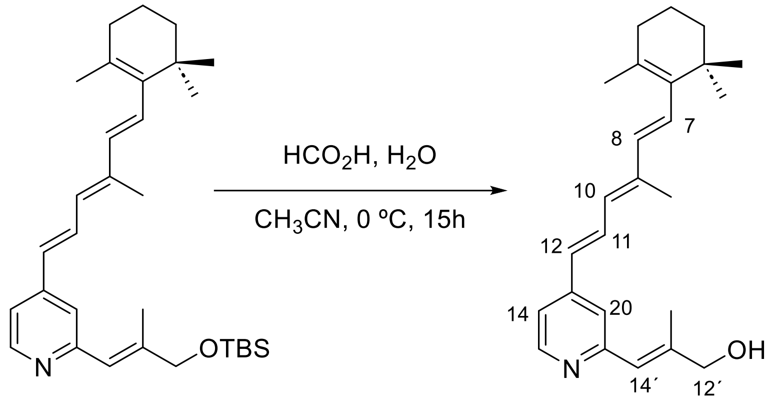

A cooled (0 °C) mixture of HCO_2H (1.5 mL, 38.68 mmol) and H_2O (0.48 mL, 26.50 mmol) was added to a cooled (0 °C) solution of **31** (53.6 mg, 0.11 mmol) in CH_3CN (3 mL). The mixture was stirred at 0 °C for 15h. After that, a saturated aqueous solution of $NaHCO_3$ was added and the mixture was extracted with EtOAc (3x). The combined organic layers were dried over Na_2SO_4, filtered and the solvent was evaporated. The residue was purified by flash column chromatography (silica gel, 98:2 (v/v) *n*-hexane/Et_3N; then, from 70:30 to 50:50 (v/v) *n*-hexane/EtOAc) to afford 36.2 mg (89% yield) of a dark orange oil, which was identified as **33**. **^{1}H-NMR** (400.16 MHz, CD_2Cl_2): δ 8.47 (d, *J* = 5.3 Hz, 1H, H_{15}), 7.41 (dd, *J* = 15.4, 11.4 Hz, 1H, H_{11}), 7.24 (s, 1H, H_{20}), 7.14 (d, *J* = 5.2 Hz, 1H, H_{14}), 6.60 (s, 1H, $H_{14'}$), 6.49 (d, *J* = 15.4 Hz, 1H, H_{12}), 6.33 (d, *J* = 16.2 Hz, 1H, H_8), 6.25 (d, *J* = 11.4 Hz, 1H, H_{10}), 6.19 (d, *J* = 16.2 Hz, 1H, H_7), 4.18 (s, 2H, $H_{12'}$), 2.09 – 1.98 (m, 8H, CH_2 + 2xCH_3), 1.74 (s, 3H, CH_3), 1.68 – 1.61 (m, 2H, CH_2), 1.55 – 1.46 (m, 2H, CH_2), 1.06 (s, 6H, 2xCH_3) ppm. **^{13}C{^{1}H}-NMR** (100.63 MHz, CD_2Cl_2): δ 157.8, 149.6, 145.9, 143.7, 140.2, 138.3, 137.8, 130.6, 130.2, 129.7, 129.6, 129.4, 123.5, 121.8, 118.3, 68.5, 40.2, 34.8, 33.6, 29.3 (2x), 22.0, 19.8, 15.9, 13.2 ppm. **IR** (NaCl): υ 3500 – 3200 (br, O-H), 2951 (s, C-H), 2925 (s, C-H), 2861 (m, C-H), 1589 (s, C=C) cm^{-1}. **HRMS** (ESI$^+$): calcd. for $C_{25}H_{34}NO$ ($[M+H]^+$) 364.2640; found, 364.2635.

Compound 34.

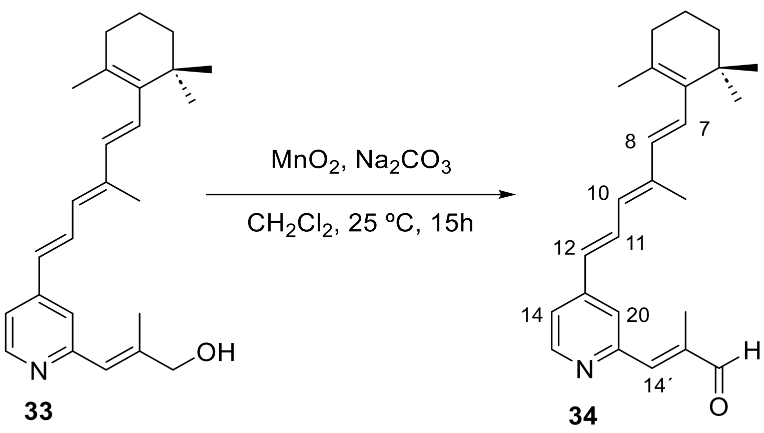

To a solution of **33** (78.0 mg, 0.22 mmol) in CH_2Cl_2 (6 mL) at room temperature, Na_2CO_3 (0.409 g, 3.86 mmol) and MnO_2 (0.336 g, 3.86 mmol) were added. The resulting reaction mixture was stirred at room temperature for 15h. The mixture was filtered through Celite® washing with CH_2Cl_2 to afford 73 mg (94% yield) of a dark red oil, which was identified as **34**. **^{1}H-NMR** (400.16 MHz, C_6D_6): δ 9.49 (s, 1H, CHO), 8.46 (d, *J* = 5.1 Hz, 1H, H_{15}), 7.23 (dd, *J* = 15.4, 11.4 Hz, 1H, H_{11}), 6.94 (s, 1H, H_{20}), 6.75 (s, 1H, $H_{14'}$), 6.69 (d, *J* = 5.1, 1H, H_{14}), 6.42 (d, *J* = 16.1 Hz, 1H, H_8), 6.35 (d, *J* = 16.1 Hz, 1H, H_7), 6.22 (d, *J* = 11.4 Hz, 1H, H_{10}), 6.19 (d, *J* = 15.3 Hz, 1H, H_{12}), 2.50 (s, 3H, CH_3), 1.98 (t, *J* = 6.4 Hz, 2H, CH_2), 1.91 (s, 3H, CH_3), 1.81 (s, 3H, CH_3), 1.63 – 1.57 (m, 2H, CH_2), 1.52 – 1.47 (m, 2H, CH_2), 1.15 (s, 6H, 2xCH_3) ppm. **^{13}C{^{1}H}-NMR** (100.63 MHz, C_6D_6): δ 194.9, 155.7, 150.4, 146.6, 145.4, 142.2, 139.7, 138.1, 137.9, 130.2, 129.9 (2x), 129.3, 129.2, 123.7, 119.7, 39.9, 34.6, 33.4, 29.2 (2x), 22.0, 19.7, 12.9, 11.4 ppm. **IR** (NaCl): υ 2924 (s, C-H), 2855 (m, C-H), 1686 (s, C=O), 1582 (m, C=C) cm^{-1}. **HRMS** (ESI^+): calcd. for $C_{25}H_{32}NO$ ($[M+H]^+$) 362.2484; found, 362.2478.

Compounds (5’*R*,8’*R*)-35a and (5’*R*,8’*S*)-35b.

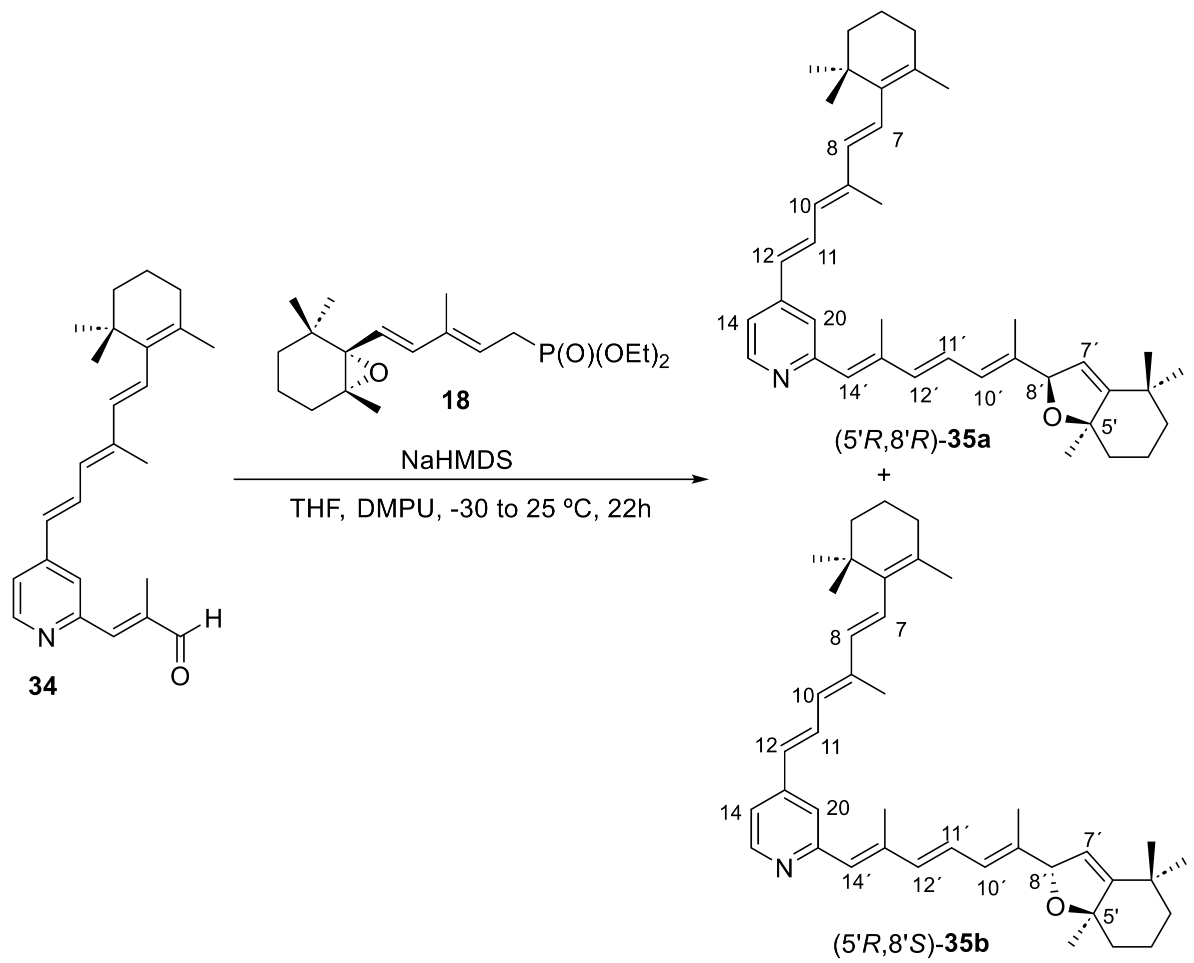

To a cooled (-30 °C) stirred solution of phosphonate **18** (0.12 g, 0.33 mmol) in THF (1 mL), DMPU (0.48 mL, 4 mmol) and NaHMDS (0.31 mL, 1M in THF, 0.31 mmol) were added. Subsequently, **34** (0.07 g, 0.19 mmol) in THF (1 mL) was added and the mixture was allowed to warm up to room temperature and stirred for 22h. A saturated aqueous solution of NH_4Cl was added and the mixture was extracted with CH_2Cl_2 (3x). The combined organic layers were washed with a saturated aqueous solution of $NaHCO_3$, dried over Na_2SO_4, filtered and the solvent was evaporated. The residue was purified by flash column chromatography (CN silica gel, 98:2 (v/v) *n*-hexane/CH_2Cl_2; then, from 95:2.5:2.5 to 60:20:20 (v/v) *n*-hexane/EtOAc/CH_2Cl_2) to afford 0.1 g (92% yield) of a dark red foam, which was identified as (5’*R*,8’*R*)-**35a** and (5’*R*,8’*S*)-**35b** as a 1:1 mixture of diastereoisomers. This mixture was further purified by HPLC (Chiralpak® IA column, Hept/CH_2Cl_2/EtOH/Et_2NH 60:40:1:0.1 v/v/v/v, 5 mL/min; detection at 347 and 245 nm) to provide the expected pure products.

Data for (5′*R*,8′*R*)-**35a**: **[α]$_D^{24}$** +30.42 (*c* 0.24, CH_2Cl_2). **UV** (CH_2Cl_2): λ_{max} 361 nm (ε = 26,900). **^{1}H-NMR** (400.16 MHz, C_6D_6): δ 8.58 (d, *J* = 5.1 Hz, 1H, H_{15}), 7.26 (dd, *J* = 15.3, 11.4 Hz, 1H, H_{11}), 7.02 (s, 1H, H_{20}), 6.90 (dd, *J* = 15.1, 11.0 Hz, 1H, $H_{11'}$), 6.71 (d, *J* = 5.1 Hz, 1H, H_{14}), 6.58 (d, *J* = 15.4 Hz, 1H, $H_{12'}$), 6.55 (s, 1H, $H_{14'}$), 6.47 (d, *J* = 11.0 Hz, 1H, $H_{10'}$), 6.39 (d, *J* = 16.3 Hz, 1H, H_7), 6.34 (d, *J* = 16.3 Hz, 1H, H_8), 6.25 (d, *J* = 15.1 Hz, 1H, H_{12}), 6.20 (d, *J* = 11.0 Hz, 1H, H_{10}), 5.32 (s, 1H, $H_{8'}$), 5.11 (s, 1H, $H_{7'}$), 2.71 (d, *J* = 1.0 Hz, 3H, CH_3), 2.00 – 1.96 (m, 4H, 2xCH_2), 1.89 (s, 3H, CH_3), 1.82 (d, *J* = 1.0 Hz, 3H, CH_3), 1.81 (s, 3H, CH_3), 1.74 – 1.65 (m, 2H, CH_2), 1.65 – 1.58 (m, 2H, CH_2), 1.52 – 1.47 (m, 5H, CH_2+CH_3), 1.44 – 1.42 (m, 2H, CH_2), 1.15 (s, 6H, 2xCH_3), 1.06 (s, 3H, CH_3), 0.99 (s, 3H, CH_3) ppm. **^{13}C{^{1}H}-NMR** (100.63 MHz, C_6D_6): δ 158.4, 154.7, 150.0, 145.0, 141.2, 140.0, 138.9, 138.8, 138.2, 138.1, 130.7, 130.3 (2x), 129.9, 129.2, 128.6, 126.9, 126.7, 122.8, 119.7, 117.6, 88.0, 87.6, 41.8, 41.7, 39.9, 34.6, 33.3, 30.9, 29.5, 29.2 (2x), 26.4, 26.1, 22.0, 20.7, 19.7, 14.5, 13.1, 12.9 ppm. **IR** (NaCl): υ 2924 (s, C-H), 2854 (m, C-H), 1592 (m, C=C), 1457 (w, C=C) cm^{-1}. **HRMS** (ESI$^+$): calcd. for $C_{40}H_{54}NO$ ([M+H]$^+$) 564.4205; found, 564.4203.

Data for (5′*R*,8′*S*)-**35b**: **[α]$_D^{24}$** -68.56 (*c* 0.32, CH_2Cl_2). **UV** (CH_2Cl_2): λ_{max} 361 nm (ε = 28,200). **^{1}H-NMR** (400.16 MHz, C_6D_6): δ 8.58 (d, *J* = 5.4 Hz, 1H, H_{15}), 7.25 (dd, *J* = 15.3, 11.4 Hz, 1H, H_{11}), 7.02 (s, 1H, H_{20}), 6.89 (dd, *J* = 15.1, 11.0 Hz, 1H, $H_{11'}$), 6.71 (d, *J* = 5.1 Hz, 1H, H_{14}), 6.60 (d, *J* = 15.1 Hz, 1H, $H_{12'}$), 6.55 (s, 1H, $H_{14'}$), 6.52 (d, *J* = 11.3 Hz, 1H, $H_{10'}$), 6.39 (d, *J* = 16.5 Hz, 1H, H_7), 6.34 (d, *J* = 16.5 Hz, 1H, H_8), 6.27 (d, *J* = 15.1 Hz, 1H, H_{12}), 6.22 (d, *J* = 10.8 Hz, 1H, H_{10}), 5.23 (s, 1H, $H_{8'}$), 5.20 (d, *J* = 1.9 Hz, 1H, $H_{7'}$), 2.71 (d, *J* = 1.0 Hz, 3H, CH_3), 2.03 – 1.94 (m, 4H, 2xCH_2), 1.89 (s, 3H, CH_3), 1.84 (d, *J* = 1.0 Hz, 3H, CH_3), 1.81 (s, 3H, CH_3), 1.63 – 1.59 (m, 2H, CH_2), 1.54 (s, 3H, CH_3), 1.52 – 1.47 (m, 2H, CH_2), 1.46 – 1.41 (m, 2H, CH_2), 1.37 – 1.30 (m, 2H, CH_2), 1.15 (s, 6H, 2xCH_3), 1.09 (s, 3H, CH_3), 0.99 (s, 3H, CH_3) ppm. **^{13}C{^{1}H}-NMR** (100.63 MHz, C_6D_6): δ 158.5, 154.0, 150.0, 145.0, 141.3, 140.5, 138.8, 138.7, 138.2, 138.1, 130.6, 130.3, 130.2, 129.9, 129.2, 128.6, 126.8, 125.8, 122.8, 118.6, 117.5, 88.3, 88.0, 42.3, 42.1, 39.9, 35.1, 34.6, 33.4, 30.8, 29.2 (2x), 27.9, 25.7, 22.0, 21.1, 19.7, 14.5, 13.7, 12.9 ppm. **IR** (NaCl): υ 2926 (s, C-H), 2861 (m, C-H), 1592 (m, C=C), 1456 (w, C=C) cm^{-1}. **HRMS** (ESI$^+$): calcd. for $C_{40}H_{54}NO$ ([M+H]$^+$) 564.4205; found, 564.4202.

(5'*R*,8'*R*)-L-Monofuran-A2E (9a).

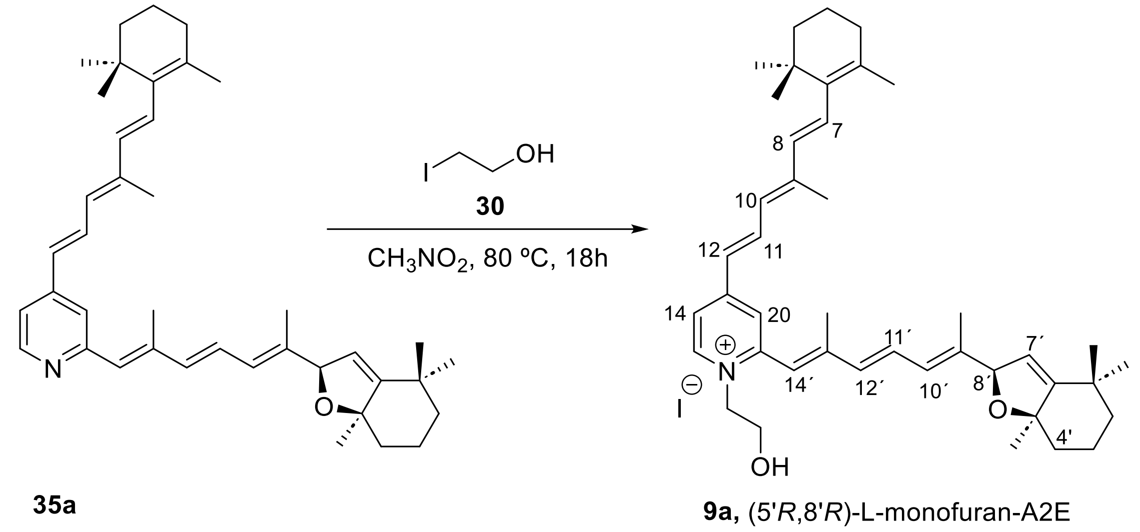

To a solution of **35a** (10 mg, 0.02 mmol) in CH_3NO_2 (0.4 mL), 2-iodoethanol **30** (0.01 mL, 0.20 mmol) was added. The resulting solution was heated at 80 °C for 18h. The solvent was evaporated, the residue was triturated with *n*-hexane and Et_2O mixtures, and the solvents were removed. The reaction mixture was concentrated under reduced pressure to afford 7.2 mg (55% yield) of a dark red solid, which was identified as (5'*R*,8'*R*)-L-monofuran-A2E (**9a**). **$[\alpha]_D^{24}$** +67.79 (*c* 0.02, CH_3OH). **UV** (CH_3OH): λ_{max} 292 nm (ε = 20,450), 425 nm (ε = 21,970). **FES** (430 nm) = λ_{max} 597 nm. **^{1}H-NMR** (400.16 MHz, CD_3OD): δ 8.55 (d, *J* = 6.8 Hz, 1H, H_{15}), 8.01 (dd, *J* = 15.2, 11.4 Hz, 1H, H_{11}), 7.95 (d, *J* = 6.7 Hz, 1H, H_{14}), 7.87 (s, 1H, H_{20}), 6.97 (dd, *J* = 15.1, 11.0 Hz, 1H, $H_{11'}$), 6.77 (d, *J* = 15.2 Hz, 1H, H_{12}), 6.71 (s, 1H, $H_{14'}$), 6.61 (d, *J* = 15.3 Hz, 1H, $H_{12'}$), 6.55 (d, *J* = 16.0 Hz, 1H, H_7), 6.42 (d, *J* = 11.5 Hz, 1H, H_{10}), 6.31 (d, *J* = 11.3 Hz, 1H, $H_{10'}$), 6.28 (d, *J* = 16.0 Hz, 1H, H_8), 5.27 (s, 1H, $H_{7'}$), 5.20 (s, 1H, $H_{8'}$), 4.54 (t, *J* = 5.0 Hz, 2H, CH_2), 3.91 (t, *J* = 5.0 Hz, 2H, CH_2), 2.18 (s, 3H, CH_3), 2.12 (s, 3H, CH_3), 2.07 (t, *J* = 6.6 Hz, 2H, CH_2), 1.99 – 1.92 (m, 1H, $H_{4'A}$), 1.82 (s, 3H, CH_3), 1.75 (s, 3H, CH_3), 1.71 – 1.63 (m, 4H, 2xCH_2), 1.61 – 1.55 (m, 2H, CH_2), 1.53 – 1.49 (m, 2H, CH_2), 1.46 (s, 3H, CH_3), 1.32 – 1.24 (m, 1H, $H_{4'B}$), 1.19 (s, 3H, CH_3), 1.14 (s, 3H, CH_3), 1.06 (s, 6H, 2xCH_3) ppm. **^{13}C{^{1}H}-NMR** (100.63 MHz, CD_3OD): δ 156.1, 154.9, 153.3, 148.6, 146.9, 146.1, 143.7, 139.5, 138.9, 138.4, 136.1, 133.0, 132.2, 131.3, 130.5, 127.3, 127.1, 127.0, 121.4, 120.8, 119.8, 89.5, 88.8, 61.0, 60.1, 42.5, 42.3, 40.8, 35.7, 35.3, 34.1, 31.1, 29.4 (2x), 26.4 (2x), 22.0, 21.3, 20.2, 14.9, 13.2, 13.0 ppm. **HRMS** (ESI$^+$): calcd. for $C_{42}H_{58}NO_2^+$ ([M$^+$]), 608.4462; found, 608.4462.

(5'*R*,8'*S*)-L-Monofuran-A2E (9b).

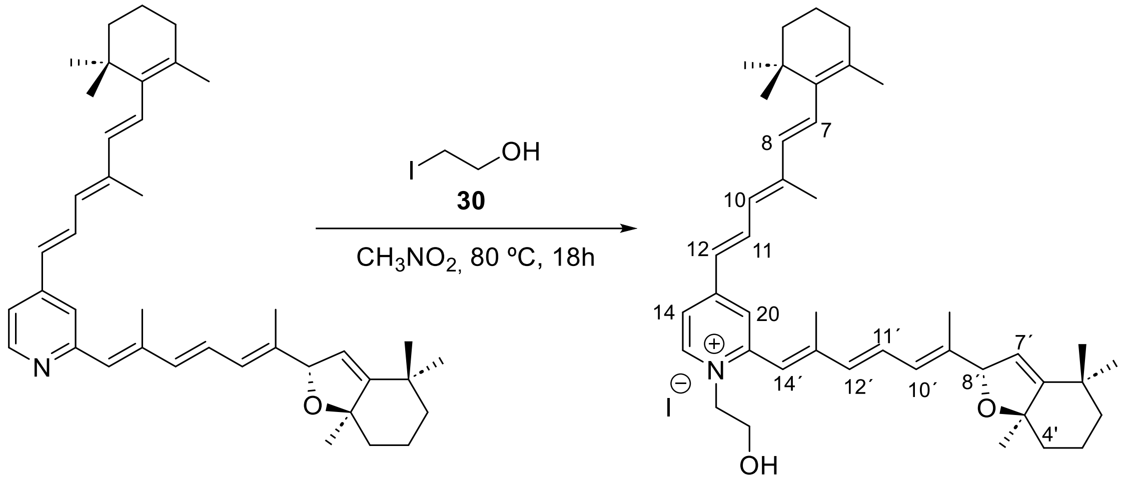

35b

9b, (5'*R*,8'*S*)-L-monofuran-A2E

To a solution of **35b** (10 mg, 0.02 mmol) in CH_3NO_2 (0.4 mL), 2-iodoethanol (0.01 mL, 0.20 mmol) was added. The resulting solution was heated at 80 °C for 18h. The solvent was evaporated, the residue was triturated with *n*-hexane and Et_2O mixtures and the solvent were removed to afford 8.1 mg (62% yield) of a dark red solid, which was identified as (5'*R*,8'*S*)-L-monofuran-A2E (**9b**). **$[\alpha]_D^{24}$** -58.31 (*c* 0.01, CH_3OH). **UV** (CH_2Cl_2): λ_{max} 297 nm (ε = 13,800), 423 nm (ε = 17,800). **FES** (430 nm) = λ_{max} 604 nm. **^{1}H-NMR** (400.16 MHz, CD_3OD): δ 8.55 (d, *J* = 6.8 Hz, 1H, H_{15}), 8.00 (dd, *J* = 15.2, 11.6 Hz, 1H, H_{11}), 7.95 (d, *J* = 6.8 Hz, 1H, H_{14}), 7.87 (s, 1H, H_{20}), 6.97 (dd, *J* = 15.3, 11.0 Hz, 1H, $H_{11'}$), 6.77 (d, *J* = 15.2 Hz, 1H, H_{12}), 6.71 (s, 1H, $H_{14'}$), 6.60 (d, *J* = 15.3 Hz, 1H, $H_{12'}$), 6.53 (d, *J* = 16.1 Hz, 1H, H_7), 6.42 (d, *J* = 11.5 Hz, 1H, H_{10}), 6.31 (d, *J* = 11.1 Hz, 1H, $H_{10'}$), 6.28 (d, *J* = 16.1 Hz, 1H, H_8), 5.35 (d, *J* = 1.9 Hz, 1H, $H_{7'}$), 5.10 (s, 1H, $H_{8'}$), 4.54 (t, *J* = 5.1 Hz, 2H, CH_2), 3.91 (t, *J* = 5.1 Hz, 2H, CH_2), 2.18 (s, 3H, CH_3), 2.12 (s, 3H, CH_3), 2.08 (t, *J* = 6.2 Hz, 2H, CH_2), 1.96 – 1.90 (m, 1H, $H_{4'A}$), 1.87 (s, 3H, CH_3), 1.75 (s, 3H, CH_3), 1.71 – 1.64 (m, 4H, 2x CH_2), 1.62 – 1.56 (m, 1H, $H_{4'B}$), 1.54 – 1.50 (m, 2H, CH_2), 1.49 (s, 3H, CH_3), 1.23 – 1.20 (m, 5H, CH_2 + CH_3), 1.15 (s, 3H, CH_3), 1.06 (s, 6H, 2xCH_3) ppm. **^{13}C{^{1}H}-NMR** (100.63 MHz, CD_3OD): δ 155.2, 154.8, 153.4, 148.6, 146.9, 146.1, 144.3, 139.5, 138.9, 138.4, 135.9, 133.0, 132.2, 131.4, 130.5, 127.1, 127.0, 126.1, 121.4, 120.7, 118.7, 89.9, 89.2, 61.0, 60.1, 43.1, 42.6, 40.8, 36.1, 35.3, 34.1, 31.1, 29.4 (2x), 28.0, 26.0, 22.0, 21.6, 20.2, 14.9, 13.6, 13.2 ppm. **HRMS** (ESI$^+$): calcd. for $C_{42}H_{58}NO_2^+$ ([M$^+$]) 608.4462; found, 608.4439.

(*E*)-2-(2-Methyl-3-oxoprop-1-en-1-yl)isonicotinaldehyde 21.

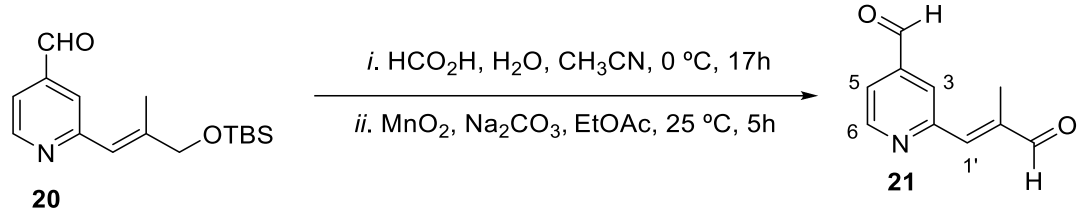

A cooled (0 °C) mixture of HCO_2H (10.2 mL, 270.67 mmol) and H_2O (3.3 mL, 172.95 mmol) was added to a cooled (0 °C) flask containing **20** (0.23 g, 0.78 mmol) and CH_3CN (21 mL). The mixture was stirred at 0 °C for 17h. A saturated aqueous solution of $NaHCO_3$ was added and the mixture was extracted with EtOAc (3x). The combined organic layers were dried over anhydrous Na_2SO_4, filtered and the solvent was evaporated.

Over a solution of the crude obtained above (0.11 g, 0.62 mmol) in EtOAc (16 mL) at room temperature, and Na_2CO_3 (1.18 g, 11.17 mmol) and MnO_2 (0.97 g, 11.17 mmol) were added. The resulting reaction mixture was stirred at room temperature for 5h. The mixture was filtered through Celite® washing with EtOAc to afford 0.073 g (66% yield) of a pink solid, which was identified as (*E*)-2-(2-methyl-3-oxoprop-1-en-1-yl)isonicotinaldehyde **21**. The spectroscopic data of the obtained compound matched those for the same product previously reported in the literature.[4] **^{1}H-NMR** (400.16 MHz, C_6D_6): δ 9.38 (s, 1H, CHO), 9.31 (s, 1H, CHO), 8.39 (d, *J* = 4.8 Hz, 1H, H_6), 7.05 (s, 1H, H_3), 6.75 (d, *J* = 4.8 Hz, 1H, H_5), 6.59 (s, 1H, $H_{1'}$), 2.33 (s, 3H, CH_3) ppm. **^{13}C{^{1}H}-NMR** (100.63 MHz, C_6D_6): δ 194.5, 190.4, 156.4, 151.0, 144.8, 143.0, 142.0, 124.1, 121.1, 11.3 ppm.

Compounds (5*R*,5′*R*,8′*R*)-38, (5*R*,8*R*,5′*R*,8′*R*)-37a, (5*R*,8*R*,5′*R*,8′*S*)-37b, (5*R*,8*S*,5′*R*,8′*R)*-37c and (5*R*,8*R*,11*Z*,5′*R*,8′*R*)-39.

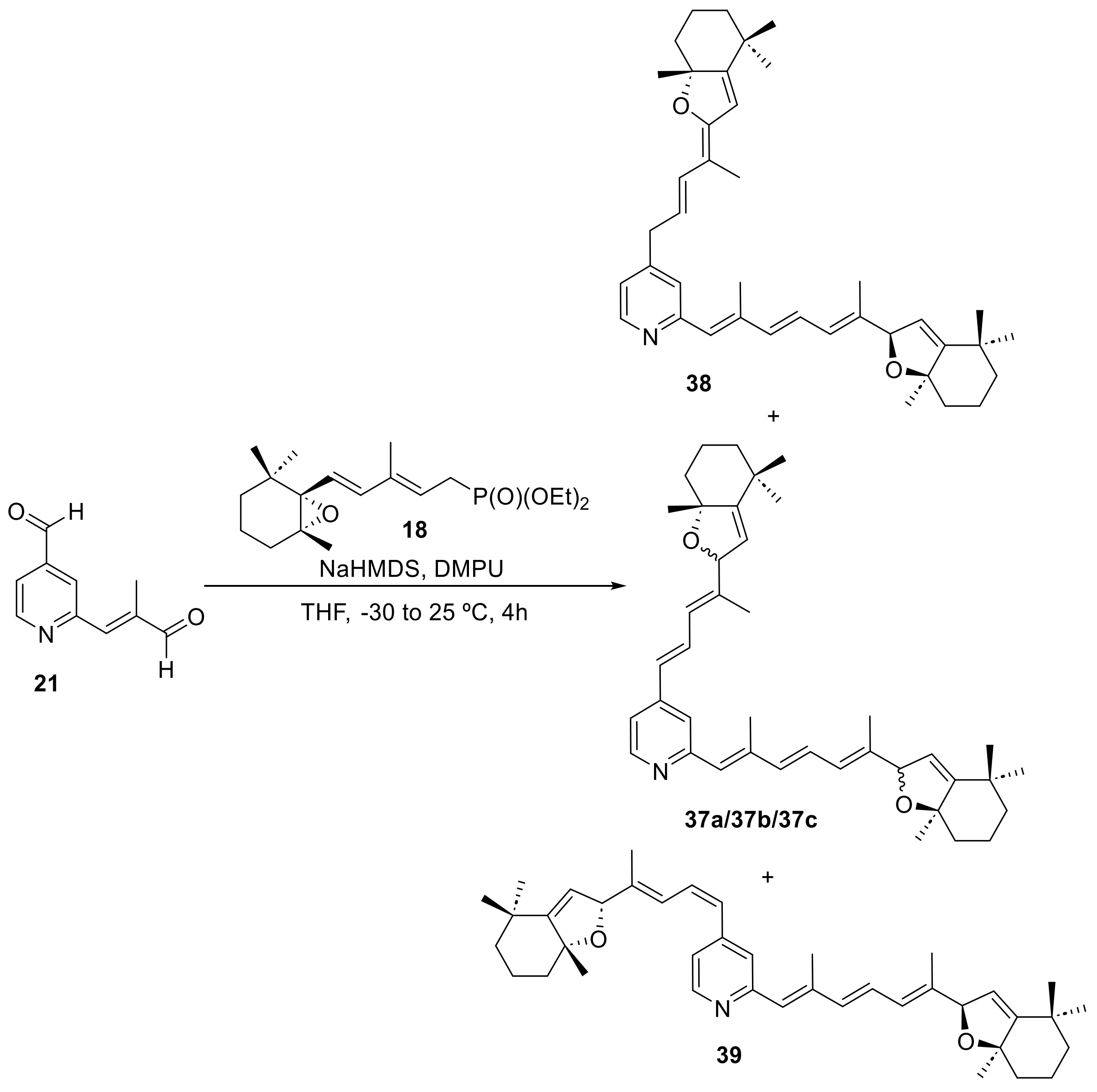

To a cooled (-30 °C) stirred solution of phosphonate **18** (100 mg, 0.29 mmol) in THF (0.4 mL), DMPU (0.21 mL, 1.77 mmol) and NaHMDS (0.27 mL, 1M in THF, 0.27 mmol) were added. A solution of **21** (15.0 mg, 0.08 mmol) in THF (0.4 mL) was then added and the mixture was allowed to warm up to room temperature for 4h. A saturated aqueous solution of NH_4Cl was added and the mixture was extracted with CH_2Cl_2 (3x). The combined organic layers were washed with a saturated aqueous solution of $NaHCO_3$, dried over Na_2SO_4, filtered and the solvent was evaporated. The residue was purified by flash column chromatography (CN silica gel, 98:2 (v/v) *n*-hexane/CH_2Cl_2; then, from

95:2.5:2.5 to 60:20:20 (v/v/v) *n*-hexane/EtOAc/CH_2Cl_2) to afford 36.7 mg (74% yield) of a dark red foam, which was identified as a mixture of (5*R*,5'*R*,8'*R*)-**38**, (5*R*,8*R*,5'*R*,8'*R*)-**37a**, (5*R*,8*R*,5'*R*,8'*S*)-**37b**, (5*R*,8*S*,5'*R*,8'*R*)-**37c** and (5*R*,8*R*,11*Z*,5'*R*,8'*R*)-**39** diastereomers in a (0.25:1:0.5:0.25:0.12) ratio. This mixture was further purified by HPLC (Chiralpak® IA column, Hept/CH_2Cl_2/EtOH/Et_2NH 60:40:1:0.1 v/v/v/v, 5 mL/min, detection at 347 and 245 nm) to afford 1.2 mg (2%) of (5*R*,5'*R*,8'*R*)-**38**, 5.0 mg (10%) of (5*R*,8*R*,5'*R*,8'*R*)-**37a**, 2.6 mg (5%) of (5*R*,8*R*,5'*R*,8'*S*)-**37b**, 1.2 mg (2%) of (5*R*,8*S*,5'*R*,8'*R*)-**37c** and 0.6 mg (1%) of (5*R*,8*R*,11*Z*,5'*R*,8'*R*)-**39**.

Data for (5*R*,5'*R*,8'*R*)-**38**: **$[\alpha]_D$**^{22} +10.08 (*c* 0.024, CH_2Cl_2). **UV** (CH_2Cl_2): λ_{max} 326 nm (ε = 34,800). **^{1}H-NMR** (400.16 MHz, CD_2Cl_2): δ 8.45 (d, *J* = 4.6 Hz, 1H, H_{15}), 7.11 (s, 1H, H_{20}), 6.95 (d, *J* = 4.3 Hz, 1H, H_{14}), 6.74 (d, *J* = 15.6 Hz, 1H, H_{10}), 6.68 (dd, *J* = 15.2, 10.9 Hz, 1H, $H_{11'}$), 6.46 (s, 1H, $H_{14'}$), 6.43 (d, *J* = 15.2 Hz, 1H, $H_{12'}$), 6.21 (d, *J* = 10.9 Hz, 1H, $H_{10'}$), 5.99 (s, 1H, H_7), 5.46 (dt, *J* = 15.6, 7.3 Hz, 1H, H_{11}), 5.20 (s, 1H, $H_{7'}$), 5.13 (s, 1H, $H_{8'}$), 3.45 (d, *J* = 7.2 Hz, 2H, $2H_{12}$), 2.33 (s, 3H, CH_3), 2.07 – 1.92 (m, 2H, CH_2), 1.75 (s, 3H, CH_3), 1.70 – 1.60 (m, 4H, 2xCH_2), 1.59 – 1.50 (m, 4H, 2xCH_2), 1.46 (s, 3H, CH_3), 1.41 (s, 3H, CH_3), 1.32 – 1.27 (m, 2H, CH_2), 1.26 (s, 3H, CH_3), 1.23 (s, 3H, CH_3), 1.16 (s, 3H, CH_3), 1.15 (s, 3H, CH_3), 1.11 (s, 3H, CH_3) ppm. **^{13}C{^{1}H}-NMR** (100.63 MHz, CD_2Cl_2): δ 161.1, 157.4, 155.9, 155.1, 151.0, 149.4, 140.4, 140.2, 138.3, 130.6, 130.2, 126.5, 126.4, 125.4, 121.5 (2x), 119.3, 114.3, 100.5, 90.7, 87.9, 87.8, 42.2, 41.8, 41.7, 40.7, 39.4, 35.8, 34.9, 30.9, 30.4, 30.1, 26.2 (2x), 25.8, 25.5, 20.8, 20.7, 14.1, 13.3, 12.9 ppm. **HRMS** (ESI$^+$): calcd. for $C_{40}H_{54}NO_2$ ($[M+H]^+$) 580.4155; found, 580.4152.

Data for (5*R*,8*R*,5'*R*,8'*R*)-**37a**: **$[\alpha]_D$**^{23} +99.55 (*c* 0.049, CH_2Cl_2). **UV** (CH_2Cl_2): λ_{max} 313 nm (ε = 21,900). **^{1}H-NMR** (400.16 MHz, CD_2Cl_2): δ 8.48 (d, *J* = 5.1 Hz, 1H, H_{15}), 7.25 (dd, *J* = 15.5, 11.1 Hz, 1H, H_{11}), 7.21 (s, 1H, H_{20}), 7.09 (dd, *J* = 5.2, 1.5 Hz, 1H, H_{14}), 6.71 (dd, *J* = 15.2, 11.0 Hz, 1H, $H_{11'}$), 6.51 – 6.46 (m, 2H, $H_{12'}$ + $H_{14'}$), 6.44 (d, *J* = 15.2 Hz, 1H, H_{12}), 6.28 (d, *J* = 11.2 Hz, 1H, H_{10}), 6.22 (d, *J* = 11.1 Hz, 1H, $H_{10'}$), 5.22 (s, 1H, H_7), 5.21 (s, 1H, $H_{7'}$), 5.15 (s, 1H, H_8), 5.13 (s, 1H, $H_{8'}$), 2.35 (d, *J* = 1.1 Hz, 3H, CH_3), 1.96 – 1.91 (m, 2H, CH_2), 1.82 (d, *J* = 1.1 Hz, 3H, CH_3), 1.76 (d, *J* = 1.1 Hz, 3H, CH_3), 1.68 – 1.61 (m, 4H, 2xCH_2), 1.59 – 1.51 (m, 4H, 2xCH_2), 1.42 (s, 3H, CH_3), 1.41 (s, 3H, CH_3), 1.29 – 1.23 (m, 2H, CH_2), 1.16 (s, 6H, 2xCH_3), 1.11 (s, 6H, 2xCH_3) ppm. **^{13}C{^{1}H}-NMR** (100.63 MHz, CD_2Cl_2): δ 157.9, 155.3, 155.1, 149.8, 145.4, 143.5, 140.8, 140.4, 138.2, 130.4, 129.8, 129.5, 126.6, 126.5, 125.4,

122.5, 119.3, 119.0, 117.9, 88.1, 87.9, 87.8, 87.5, 41.8 (2x), 41.7 (2x), 34.9 (2x), 30.9, 30.8, 26.2 (4x), 20.8 (2x), 14.2, 13.2, 13.0 ppm. **HRMS** (ESI^+): calcd. for $C_{40}H_{54}NO_2$ ($[M+H]^+$) 580.4155; found, 580.4149.

Data for (5*R*,8*R*,5'*R*,8'*S*)-**37b**: $[\alpha]_D^{24}$ +30.53 (*c* 0.034, CH_2Cl_2). **UV** (CH_2Cl_2): λ_{max} 314 nm (ε = 19,200). **^{1}H-NMR** (400.16 MHz, CD_2Cl_2): δ 8.48 (d, *J* = 5.1 Hz, 1H, H_{15}), 7.25 (dd, *J* = 15.5, 11.1 Hz, 1H, H_{11}), 7.21 (s, 1H, H_{20}), 7.09 (d, *J* = 5.2 Hz, 1H, H_{14}), 6.71 (dd, *J* = 15.1, 11.0 Hz, 1H, $H_{11'}$), 6.48 (s, 1H, $H_{14'}$), 6.47 (d, *J* = 15.5 Hz, 1H, H_{12}), 6.45 (d, *J* = 15.1 Hz, 1H, $H_{12'}$), 6.27 (d, *J* = 11.2 Hz, 1H, H_{10}), 6.22 (d, *J* = 11.0 Hz, 1H, $H_{10'}$), 5.27 (d, *J* = 1.9 Hz, 1H, $H_{7'}$), 5.22 (s, 1H, H_7), 5.15 (s, 1H, H_8), 5.04 (s, 1H, $H_{8'}$), 2.35 (d, *J* = 1.0 Hz, 3H, CH_3), 1.99 – 1.85 (m, 2H, CH_2), 1.82 (d, *J* = 1.1 Hz, 3H, CH_3), 1.81 (d, *J* = 1.1 Hz, 3H, CH_3), 1.66 – 1.61 (m, 4H, 2xCH_2), 1.57 – 1.50 (m, 4H, 2xCH_2), 1.45 (s, 3H, CH_3), 1.42 (s, 3H, CH_3), 1.30 – 1.24 (m, 2H, CH_2), 1.18 (s, 3H, CH_3), 1.16 (s, 3H, CH_3), 1.12 (s, 3H, CH_3), 1.11 (s, 3H, CH_3) ppm. **^{13}C{^{1}H}-NMR** (100.63 MHz, CD_2Cl_2): δ 157.9, 155.3, 154.2, 149.8, 145.4, 143.5, 140.9 (2x), 138.1, 130.2, 129.8, 129.5, 126.7, 125.4 (2x), 122.5, 119.0, 118.3, 117.9, 88.30, 88.27, 88.1, 87.5, 42.5, 42.0, 41.8, 41.7, 35.3, 34.9, 30.8 (2x), 27.7, 26.2 (2x), 25.7, 21.1, 20.8, 14.2, 13.6, 13.2 ppm. **HRMS** (ESI^+): calcd. for $C_{40}H_{54}NO_2$ ($[M+H]^+$) 580.4155; found, 580.4151.

Data for (5*R*,8*S*,5'*R*,8'*R*)-**37c**: $[\alpha]_D^{24}$ -28.56 (*c* 0.050, CH_2Cl_2). **UV** (CH_2Cl_2): λ_{max} 314 nm (ε = 23,100). **^{1}H-NMR** (400.16 MHz, CD_2Cl_2): δ 8.48 (d, *J* = 5.2 Hz, 1H, H_{15}), 7.25 (dd, *J* = 15.5, 11.2 Hz, 1H, H_{11}), 7.21 (br s, 1H, H_{20}), 7.09 (dd, *J* = 5.2, 1.5 Hz, 1H, H_{14}), 6.71 (dd, *J* = 15.2, 11.0 Hz, 1H, $H_{11'}$), 6.48 (s, 1H, $H_{14'}$), 6.47 (d, *J* = 15.0 Hz, 1H, $H_{12'}$), 6.44 (d, *J* = 15.3 Hz, 1H, H_{12}), 6.29 (d, *J* = 11.2 Hz, 1H, H_{10}), 6.22 (d, *J* = 11.1 Hz, 1H, $H_{10'}$), 5.29 (d, *J* = 2.0 Hz, 1H, H_7), 5.21 (s, 1H, $H_{7'}$), 5.13 (s, 1H, $H_{8'}$), 5.06 (s, 1H, H_8), 2.35 (d, *J* = 1.0 Hz, 3H, CH_3), 1.96 – 1.90 (m, 2H, CH_2), 1.86 (d, *J* = 1.1 Hz, 3H, CH_3), 1.76 (d, *J* = 1.1 Hz, 3H, CH_3), 1.67 – 1.61 (m, 4H, 2xCH_2), 1.55 – 1.51 (m, 4H, 2xCH_2), 1.46 (s, 3H, CH_3), 1.41 (s, 3H, CH_3), 1.34 – 1.19 (m, 2H, CH_2), 1.18 (s, 3H, CH_3), 1.16 (s, 3H, CH_3), 1.11 (s, 3H, CH_3), 1.10 (s, 3H, CH_3) ppm. **^{13}C{^{1}H}-NMR** (100.63 MHz, CD_2Cl_2): δ 157.9, 155.1, 154.4, 149.8, 145.5, 144.0, 140.8, 140.4, 138.2, 130.4, 129.6 (2x), 126.6, 126.5, 124.3, 122.5, 119.3, 118.0, 117.9, 88.5, 88.0, 87.9, 87.8, 42.4, 41.9, 41.8, 41.7, 35.4, 34.9, 30.9, 30.8, 27.8, 26.3, 26.2, 25.7, 21.1, 20.8, 14.2, 13.8, 13.0 ppm. **HRMS** (ESI^+): calcd. for $C_{40}H_{54}NO_2$ ($[M+H]^+$) 580.4155; found, 580.4151.

Data for (5*R*,8*R*,11*Z*,5'*R*,8'*R*)-**39**: **[α]$_D$**^{24} -30.21 (*c* 0.033, CH_2Cl_2). **UV** (CH_2Cl_2): λ_{max} 308 nm (ε = 33,600). **^{1}H-NMR** (400.16 MHz, CD_2Cl_2): δ 8.51 (d, *J* = 5.0 Hz, 1H, H_{15}), 7.18 (br s, 1H, H_{20}), 7.02 (dd, *J* = 5.0, 1.4 Hz, 1H, H_{14}), 6.70 (dd, *J* = 15.1, 11.0 Hz, 1H, $H_{11'}$), 6.65 – 6.61 (m, 2H, H_{11} + H_{12}), 6.47 (s, 1H, H_{14}), 6.43 (d, *J* = 15.2 Hz, 1H, $H_{12'}$), 6.36 – 6.31 (m, 1H, H_{10}), 6.22 (d, *J* = 10.9 Hz, 1H, $H_{10'}$), 5.22 (s, 1H, H_7), 5.21 (s, 1H, $H_{7'}$), 5.13 (s, 1H, $H_{8'}$), 5.11 (s, 1H, H_8), 2.35 (d, *J* = 1.0 Hz, 3H, CH_3), 1.92 (t, *J* = 10.7 Hz, 2H, CH_2), 1.79 (s, 3H, CH_3), 1.76 (d, *J* = 1.2 Hz, 3H, CH_3), 1.67 – 1.60 (m, 4H, 2xCH_2), 1.54 – 1.49 (m, 4H, 2xCH_2), 1.41 (s, 3H, CH_3), 1.39 (s, 3H, CH_3), 1.28 – 1.18 (m, 2H, CH_2), 1.16 (s, 3H, CH_3), 1.15 (s, 3H, CH_3), 1.11 (s, 3H, CH_3), 1.10 (s, 3H, CH_3) ppm. **^{13}C{^{1}H}-NMR** (100.63 MHz, CD_2Cl_2): δ 157.5, 155.1, 155.0, 149.5, 145.4, 144.6, 140.7, 140.4, 138.2, 130.4, 129.7, 127.0, 126.6, 126.5, 125.3, 121.2, 121.1, 119.3, 118.9, 88.2, 87.9, 87.8, 87.4, 41.8 (3x), 41.7, 34.9, 34.9, 30.9 (2x), 26.3 (2x), 26.2, 26.1, 20.8, 20.7, 14.2, 13.2, 13.0 ppm. **HRMS** (ESI$^+$): calcd. for $C_{40}H_{54}NO_2$ ($[M+H]^+$) 580.4155; found, 580.4150.

Compounds (5*R*,8*R*,5'*R*,8'*R*)-37a, (5*R*,8*R*,5'*R*,8'*S*)-37b and (5*R*,8*S*,5'*R*,8'*R*)-37c.

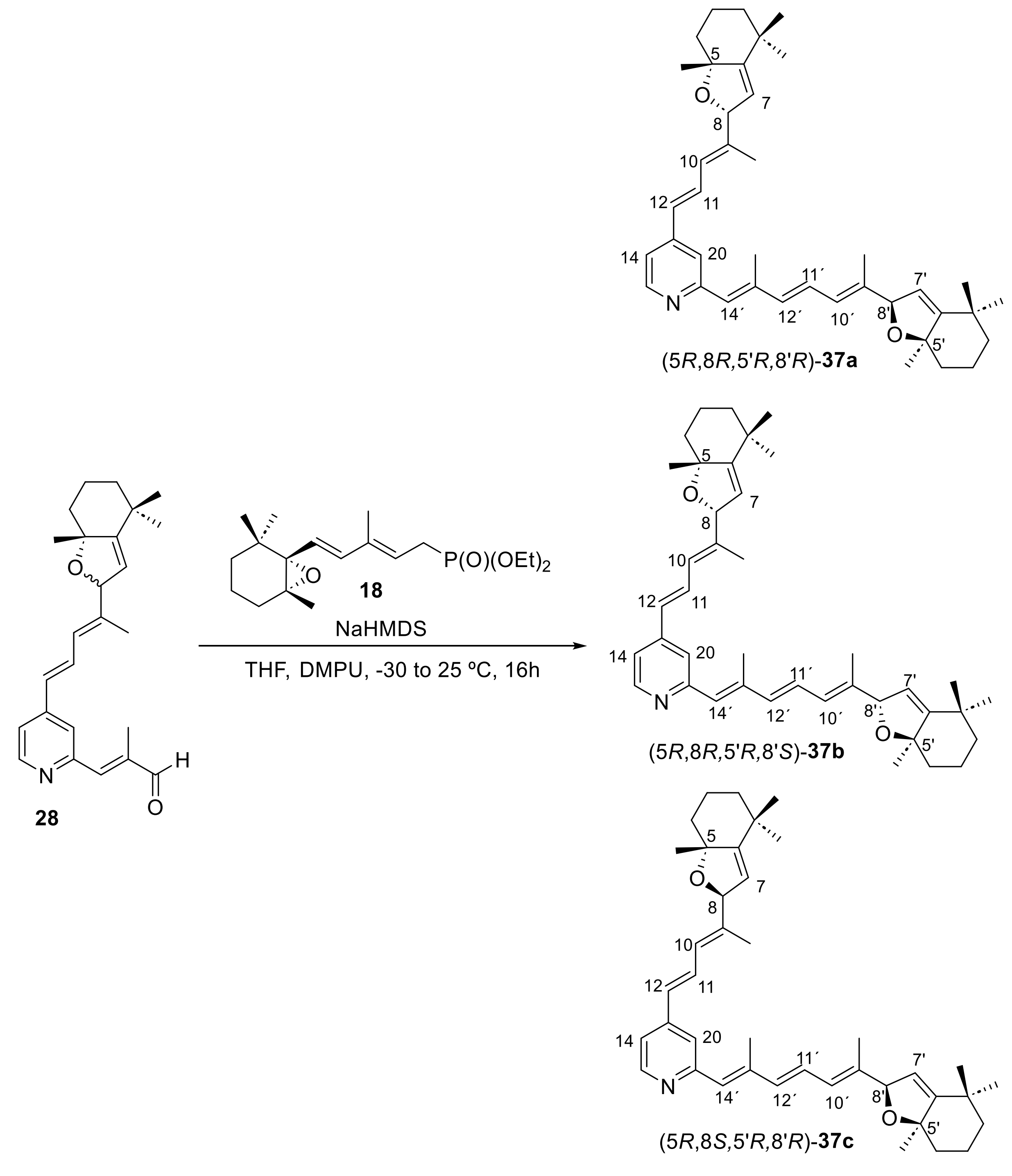

To a cooled (-30 °C) stirred solution of phosphonate **18** (30.2 mg, 0.08 mmol) in THF (0.3 mL), DMPU (0.16 mL, 1.32 mmol) and NaHMDS (0.10 mL, 1M in THF, 0.10 mmol) were added. Subsequently, **28** (18.8 mg, 0.05 mmol) in THF (0.3 mL) was added and the mixture was allowed to warm up to room temperature for 16h. A saturated aqueous solution of NH_4Cl was added and the mixture was extracted with CH_2Cl_2 (3x). The combined organic layers were washed with a saturated aqueous solution of $NaHCO_3$,

dried over Na_2SO_4, filtered and the solvent was evaporated. The residue was purified by flash column chromatography (CN silica gel, 98:2 (v/v) *n*-hexane/CH_2Cl_2; then, from 95:2.5:2.5 to 60:20:20 (v/v) *n*-hexane/EtOAc/CH_2Cl_2) to afford 14.2 mg (50% yield) of a dark red foam, which was identified as a mixture of the (5*R*,8*R*,5'*R*,8'*R*)-**37a**, (5*R*,8*R*,5'*R*,8'*S*)-**37b**, (5*R*,8*S*,5'*R*,8'*R*)-**37c** diastereomers in a (1:0.5:0.25) ratio.

(5*R*,8*R*,5'*R*,8'*R*)-Bisfuran-A2E (10a).

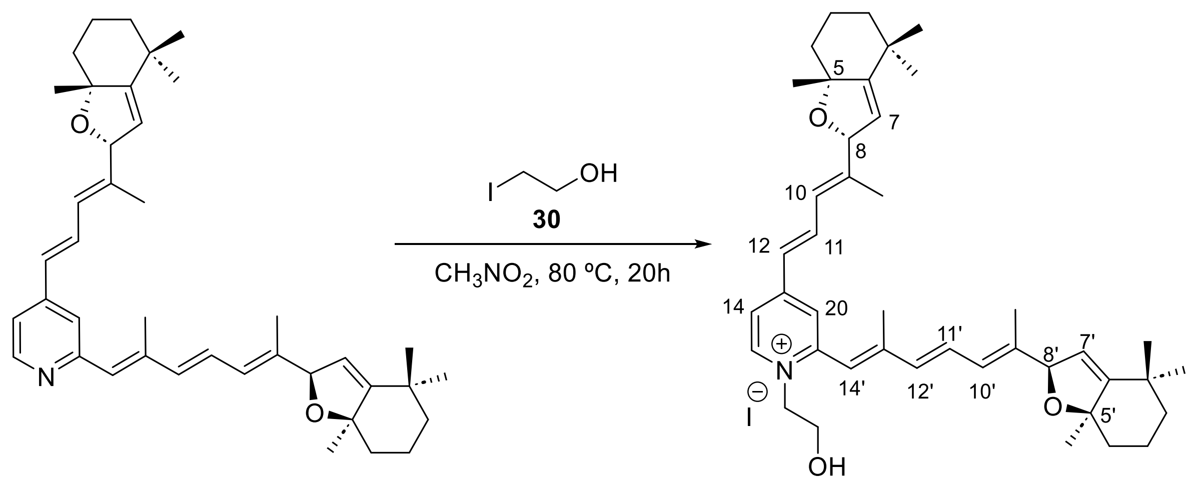

37a

10a, (5*R*,8*R*,5'*R*,8'*R*)-bisfuran-A2E

To a solution of (5*R*,8*R*,5'*R*,8'*R*)-**37a** (6.0 mg, 0.01 mmol) in CH_3NO_2 (0.2 mL), 2-iodoethanol (0.01 mL, 0.10 mmol) was added. The resulting solution was heated at 80 °C for 20h. The solvent was evaporated, the residue was triturated with *n*-hexane and Et_2O mixtures, and the solvents were removed to afford 4.1 mg (63% yield) of a dark red solid, which was identified as (5*R*,8*R*,5'*R*,8'*R*)-bisfuran-A2E (**10a**). **$[\alpha]_D^{25}$** +10.14 (*c* 0.01, CH_3OH). **UV** (CH_3OH): λ_{max} 293 nm (ε = 10,400), 368 nm (ε = 14,800). **FES** (430 nm) = λ_{max} 543 nm. **^{1}H-NMR** (400.16 MHz, CD_3OD): δ 8.59 (d, *J* = 6.8 Hz, 1H, H_{15}), 7.97 (dd, *J* = 6.8, 2.1 Hz, 1H, H_{14}), 7.91 (d, *J* = 2.1 Hz, 1H, H_{20}), 7.85 (dd, *J* = 15.4, 11.2 Hz, 1H, H_{11}), 6.98 (dd, *J* = 15.2, 11.0 Hz, 1H, $H_{11'}$), 6.78 (d, *J* = 15.4 Hz, 1H, H_{12}), 6.72 (s, 1H, $H_{14'}$), 6.60 (d, *J* = 15.3 Hz, 1H, $H_{12'}$), 6.47 (d, *J* = 11.1 Hz, 1H, H_{10}), 6.31 (d, *J* = 11.1 Hz, 1H, $H_{10'}$), 5.31 (s, 1H, H_7), 5.27 (s, 1H, $H_{7'}$), 5.24 (s, 1H, H_8), 5.20 (s, 1H, $H_{8'}$), 4.61 – 4.54 (m, 2H, CH_2), 3.92 (t, *J* = 5.0 Hz, 2H, CH_2), 2.12 (d, *J* = 1.1 Hz, 3H, CH_3), 2.01 – 1.92 (m, 5H, CH_2 + CH_3), 1.82 (d, *J* = 1.3 Hz, 3H, CH_3), 1.72 – 1.67 (m, 4H, 2xCH_2), 1.63 – 1.54 (m, 4H, 2xCH_2), 1.46 (s, 3H, CH_3), 1.45 (s, 3H, CH_3), 1.30 – 1.21 (m, 2H, CH_2), 1.21 – 1.17 (m, 6H, 2xCH_3), 1.15 (s, 6H, 2xCH_3) ppm. The ^{1}H NMR data for the racemate has been reported.[5] **^{1}H-NMR** (500 MHz, CD_3OD):

8.53 (d, J = 7.0 Hz, 1H), 7.91 (d, J = 7.0 Hz, 1H), 7.86 (s, 1H), 7.81 (dd, J = 15.3, 11.2 Hz, 1H), 6.93 (dd, J = 15.0, 11.3 Hz, 1H), 6.73 (d, J = 15.3 Hz, 1H), 6.66 (s, 1H), 6.56 (d, J = 15.6 Hz, 1H), 6.43 (d, J = 11.2 Hz, 1H), 6.26 (d, J = 11.3 Hz, 1H), 5.26 (s, 1H, $H_{7'}$), 5.22 (s, 1H, H_7), 5.19 (s, 1H, $H_{8'}$), 5.15 (s, 1H, H_8), 4.50 (s, 2H), 3.87 (s, 2H), 2.07 (s, 3H), 1.91 (s, 3H), 1.77 (s, 3H), 1.42 (s, 3H), 1.41 (s, 3H), 1.14 (s, 6H) ppm. HSQC (500 MHz, CD_3OD): 86.7 (C-8), 86.8 (C-8′), 117.6 (C-7), 117.6 (C-7′). **$^{13}C\{^1H\}$-NMR** (100.63 MHz, CD_3OD): δ 156.3, 156.1, 154.9, 153.6, 150.8, 148.8, 146.4, 143.8, 138.9, 136.0, 131.4, 127.7, 127.5, 127.3, 126.3, 121.7, 120.7, 119.8, 119.4, 89.8, 89.5, 88.8, 88.3, 61.0, 60.2, 42.5 (2x), 42.4, 42.3, 35.7 (2x), 31.1 (2x), 26.4 (4x), 21.3 (2x), 14.9, 13.7, 13.0 ppm. **HRMS** (ESI^+): calcd. for $C_{42}H_{58}NO_3^+$ ($[M^+]$) 624.4411; found, 624.4412.

3. TEM Images

Transmission electron microscopy (TEM) images were obtained on a Thermoionic JEOL JEM1010 working at 100 kV. Samples were prepared by dropping the solutions onto a 400-mesh copper grid coated with formvar and carbon.

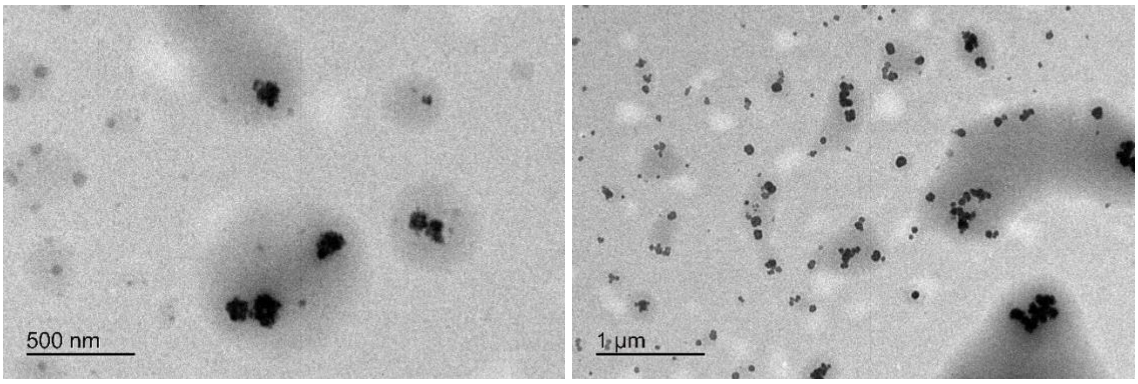

Figure S2. Additional TEM images of **8a** at a 20:80 *v/v* MeOH/H_2O ratio (75 µM).

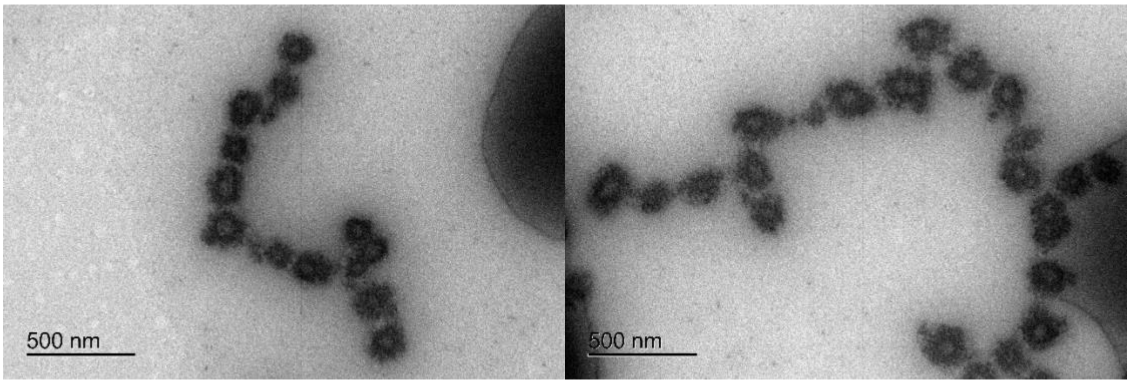

Figure S3. Additional TEM images of **9a** at a 20:80 *v/v* MeOH/H_2O ratio (75 µM).

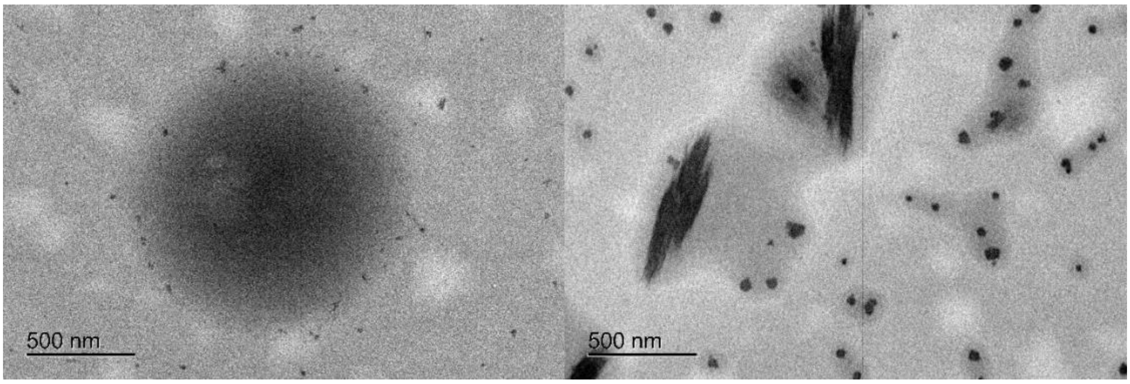

Figure S4. Additional TEM images of **9b** at a 20:80 *v/v* MeOH/H_2O ratio (75 µM).

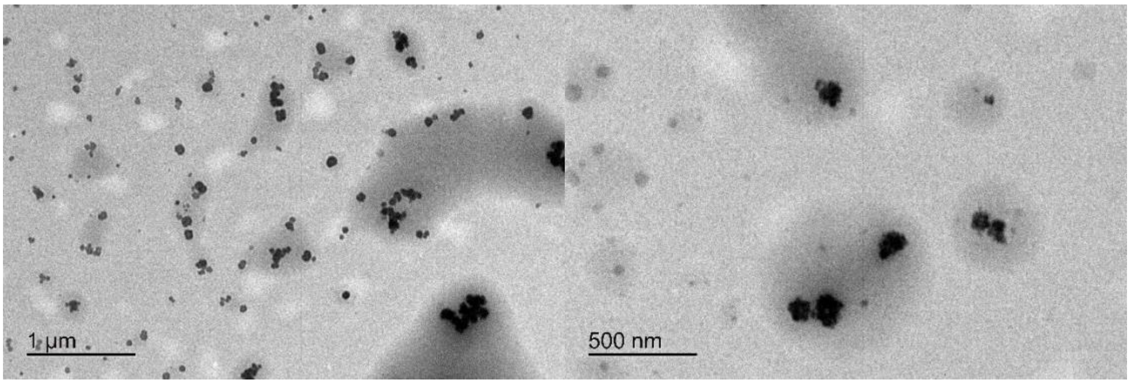

Figure S5. Additional TEM images of **10a** at a 20:80 *v/v* MeOH/H_2O ratio (75 µM).

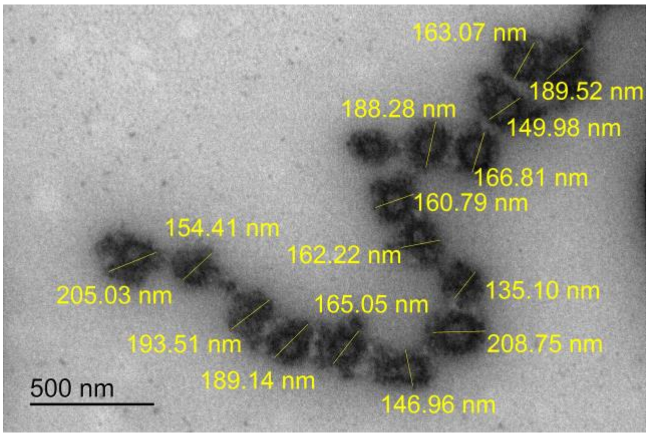

Figure S6. TEM image showing the size of the aggregates of **9a**.

4. Comparison of the ^{1}H-NMR spectra of 9a at different CD_3OD/D_2O ratios

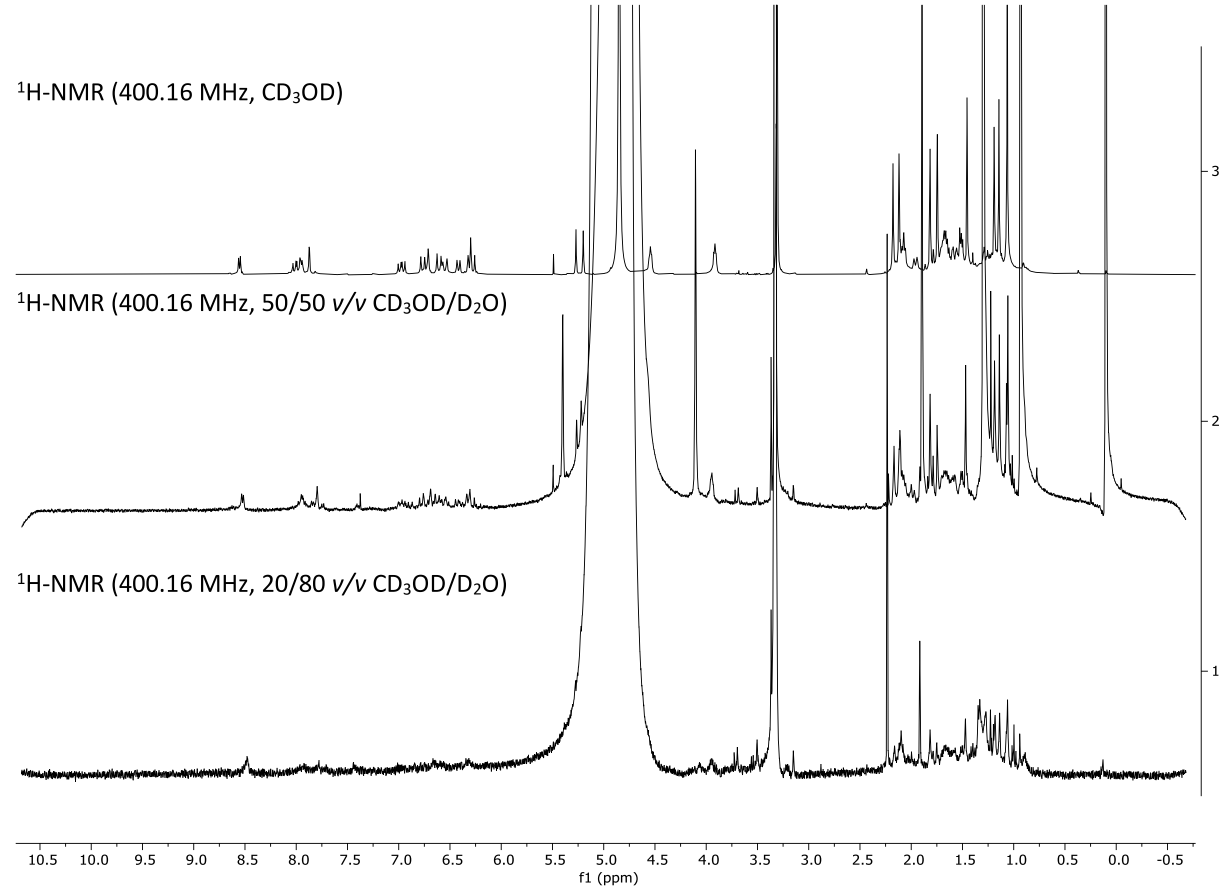

Figure S7. Comparison of the ^{1}H NMR spectra of (5'*R*,8'*R*)-L-monofuran-A2E **9a** (1 mM) in CD_3OD, and in 50/50 *v/v* CD_3OD/D_2O and 20/80 *v/v* CD_3OD/D_2O solvent mixtures.

5. Additional UV-Vis spectra of 9a and analogues

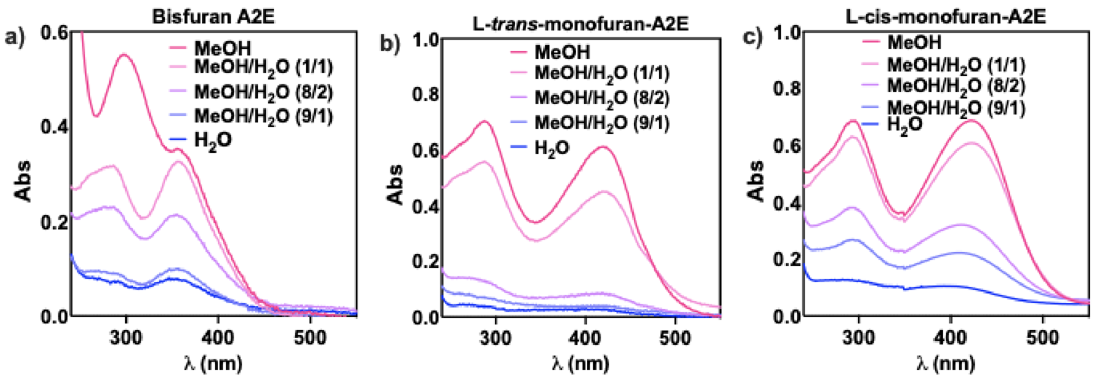

Figure S8. Comparison among UV-Vis spectra of a) **10a**, b) **9a**, and c) **9b** using different volume fractions of H_2O in MeOH (c_T = 50 µM).

6. DFT-simulation of pKa values

The free energies for $G_{aq}AH$ and $G_{aq}A^-$ were computed using the Gaussian 16 suite of programs[6] at 298K and SMD as solvation model (in water).[7] All calculation were performed using M06-2X, [8] and aug-cc-pVDZ,[9] as basic functions.

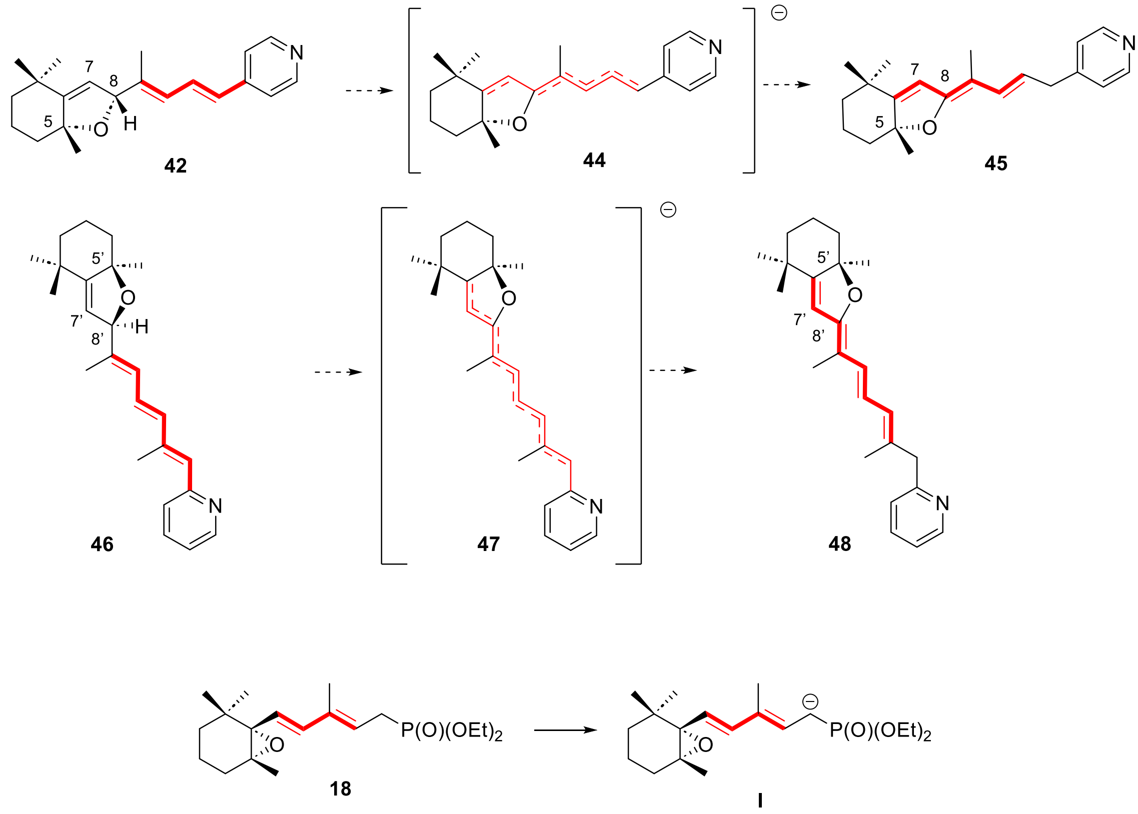

$$K_a = \frac{[H_3O][A^-]}{[AH]} \qquad pK_a = (1/RT\ln 10) \times [G_{aq}(A^-) + \bar{G}_{aq}(H_3O^+) - G_{aq}(AH)]$$

Scheme S1. Simulation of pKa values.

Table S2. DFT-energies of the deprotonated and neutral species from deprotonation of models **42** and **46** and of phosphonate **18** at the M062X/aug-cc-pVDZ(SDD, water) level.

	SCF	SCF+zpve	Enthalpy	TS	Free energies
**44**	-593423,365	-593158,465	-593144,173	46,2148565	-593190,388
**45**	-593731,622	-593457,838	-593443,537	46,5028836	-593490,040
**47**	-666637,920	-666334,429	-666317,904	51,6572507	-666369,562
**48**	-666949,273	-666637,082	-666620,473	51,9157848	-666672,388
**I**	-819999,855	-819731,721	-819715,058	52,0695248	-819767,128
**18**	-820311,481	-820035,088	-820018,419	52,1247457	-820070,544

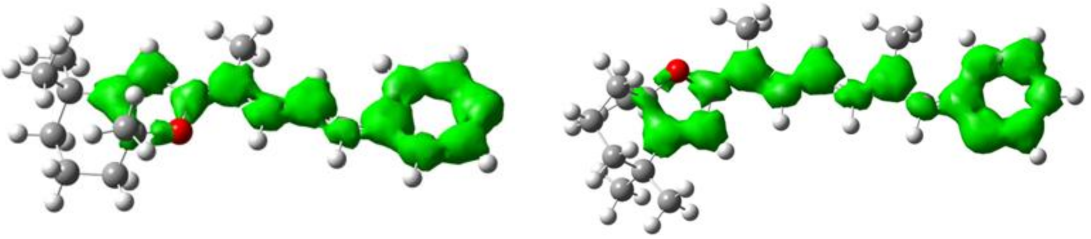

Figure S9. ACID plots at an isosurface value of 0.055 of **44** (left, CIV 0.056) and **47** (right, CIV 0.053).[10]

7. DFT studies of the epoxycyclohexanedienylphosphonate to hexahydrobenzofuran alkenylphosphonate rearrangement under basic conditions

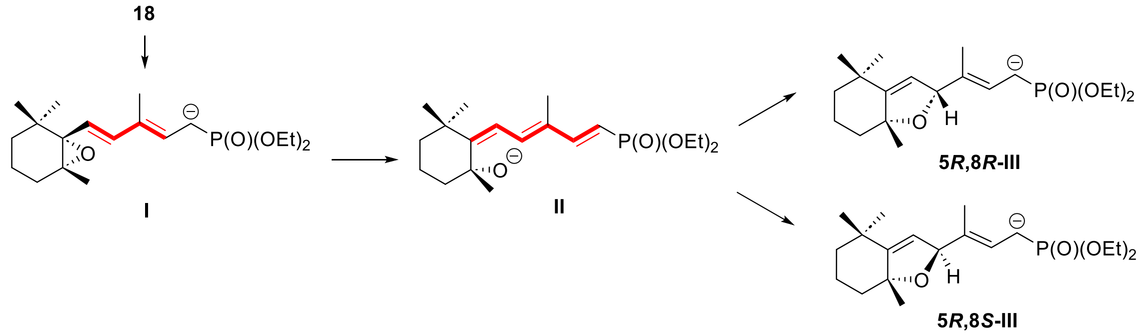

Scheme S2. Rearrangement of the epoxycyclohexanedienylphosphonate to the hexahydrobenzofuran alkenylphosphonate.

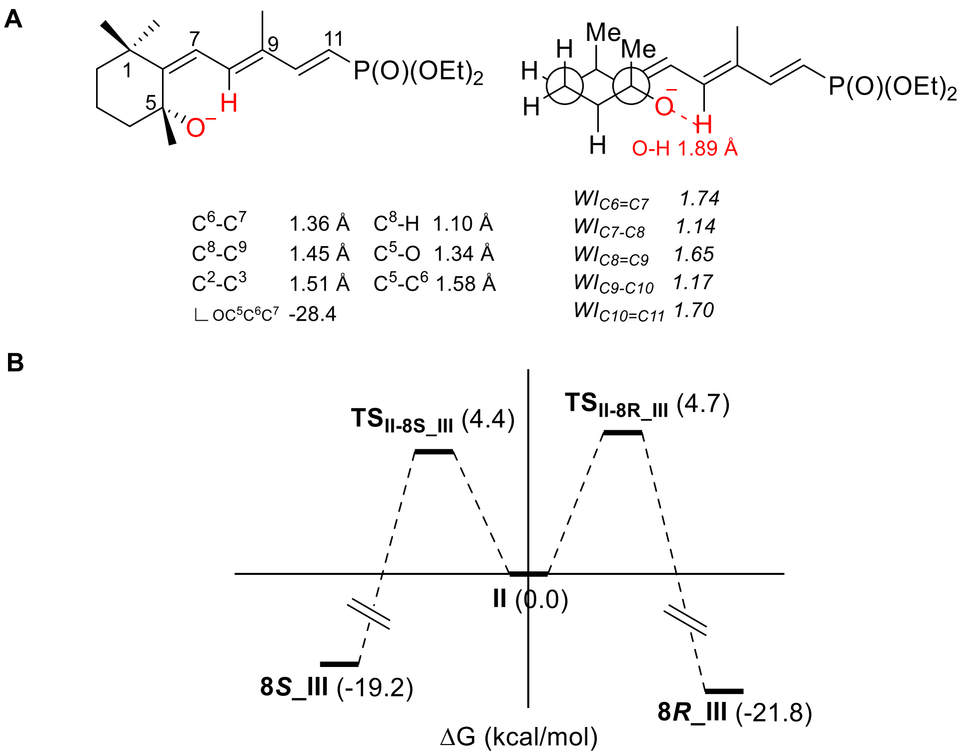

Figure S10. A. Selected geometric parameters for the epoxycyclohexenedienyl phosphonate **II**; the Wiberg index obtained by NBO are shown. B. DFT-profile (in kcal/mol, [wB97XD/def2svp (PCM, THF)]) for the rearrangement under basic conditions of epoxycyclohexanedienylphosphonate to hexahydrobenzofuran alkenylphosphonate.

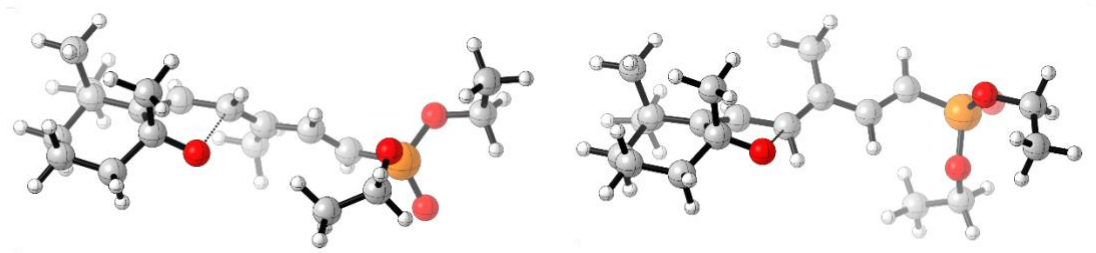

Figure S11. Transition states **TS$_{II-8R_III}$** (left, C^8-O distance of 2.20 Å) and **TS$_{II-8S_III}$** (right, C^8-O distance of 2.48 Å) computed at the [wB97XD/def2svp (PCM, THF)][11-13] level for the rearrangement of the epoxycyclohexenedienyl phosphonate to the hexahydrobenzofuran alkenylphosphonate (http://www.cylview.org).[14]

Table S3. DFT-energies (in kcal/mol) of the mechanism of epoxycyclohexenedienylphosphonate to hexahydrobenzofuran alkenylphosphonate rearrangement under basic conditions [wB97XD/def2svp (PCM, THF)].

	SCF	SCF+zpve	Enthalpy	TS	Free	ΔG
**II**	-868772,014	-868467,623	-868449,64	55,662647	-868505,303	0.0
**TS$_{II-8R_III}$**	-868769,18	-868464,748	-868447,42	53,1670398	-868500,587	***4.7***
**8*R*_III**	-868795,687	-868490,322	-868472,725	54,3360909	-868527,061	-21.8
**TS$_{II-8S_III}$**	-868769,349	-868465,309	-868448,335	52,5671402	-868500,902	***4.4***
**8*S*_III**	-868795,011	-868488,888	-868471,583	52,8997205	-868524,483	-19.2

8. DFT studies for the rearrangement of 4-epoxycyclohexanetrienylpyridine 40 under acidic conditions.

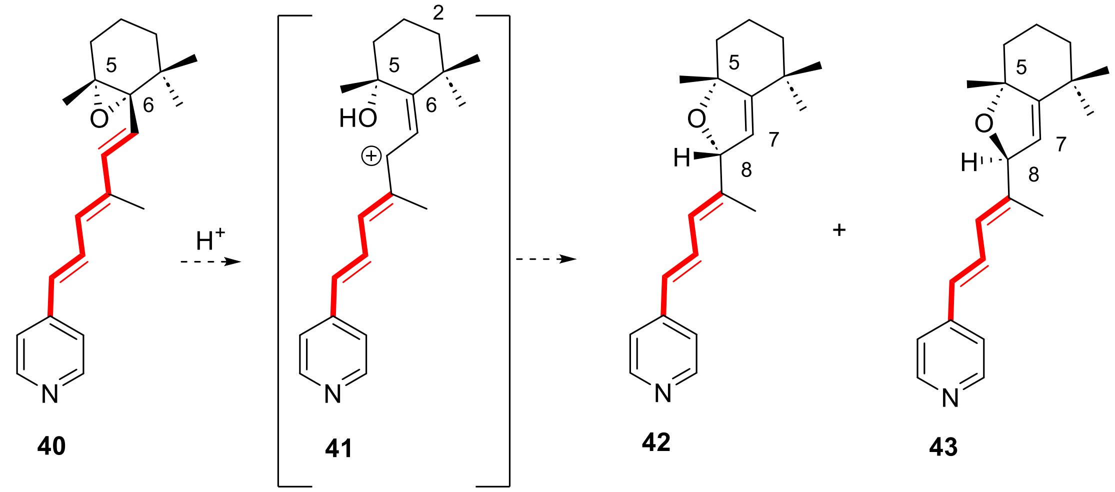

C^6C^7 1.37 Å
C^8C^9 1.40 Å
C^2C^3 1.52 Å
C^5O 1.41 Å
C^5C^6 1.54 Å

$<OC^5C^6C^7$ -29.8º

Scheme S3. Rearrangement of **40**, and selected bond distances and angles for intermediate **41**.

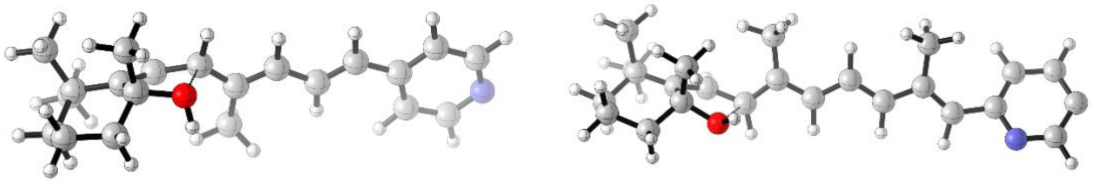

Figure S12. Transition states **TS_{41-42}** (left, C^8-O distance 2.12 Å) and **TS_{41-43}** (right, C^8-O distance 2.07 Å) computed at the wB97XD/def2svp (PCM, THF) level.[14] (http://www.cylview.org)

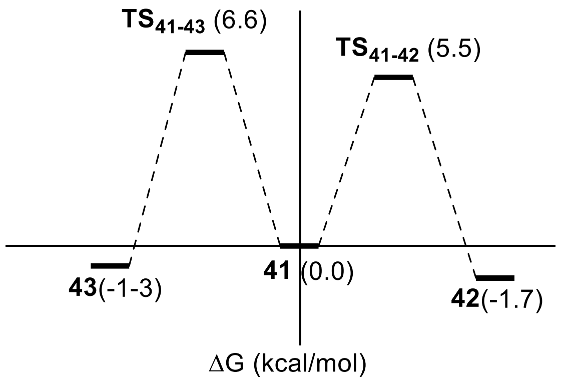

Figure S13. DFT-profile (in kcal/mol, [wB97XD/def2svp (PCM, THF)]) of the rearrangement of 4-epoxycyclohexanetrienylpyridine **41** under acidic conditions.

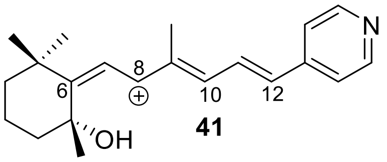

41

Table S4. Most relevant stabilizing interactions of **41**, characterized by NBO (or E(2) analysis).

Donor NBO (i)	Acceptor NBO (j)	E(2) (kcal/mol)	E(j)-E(i) (a.u.)	F(I,j) (a.u.)
C6-C7	C8	95.16	0.22	0.146
C9-C10	C8	126.49	0.21	0.160
C9-C10	C11-C12	13.06	0.41	0.071
C11-C12	C9-C10	42.26	0.42	0.120

Table S5. DFT-energies (in kcal/mol) for the mechanism of 4-epoxycyclohexanetrienylpyridine rearrangement under acidic conditions at the wB97XD/def2svp (PCM, CH_3CN) level.

	SCF	SCF+zpve	Enthalpy	TS	Free	ΔG
**41**	-593554,02	-593272,39	-593257,25	49,15	-593306,39	0.00
**TS$_{41-42}$**	-593548,86	-593267,95	-593253,30	47,57	-593300,87	***5.5***
**42**	-593557,43	-593275,10	-593260,40	47,66	-593308,06	-1.7
**TS$_{41-43}$**	-593548,32	-593267,33	-593252,77	47,01	-593299,79	***6.6***
**43**	-593557,59	-593274,95	-593260,37	47,31	-593307,69	-1.3

9. References

(1) Acemoglu, M.; Prewo, R.; Bieri, J. H.; Eugster, C. H. *Helv. Chim. Acta* **1984**, *67*, 175.

(2) Acemoglu, M.; Eugster, C. H. *Helv. Chim. Acta* **1984**, *67*, 184.

(3) Baumeler, A.; Eugster, C. H. *Helv. Chim. Acta* **1992**, *75*, 773.

(4) Ren, R. X.-F.; Sakai, N.; Nakanishi, K. *J. Am. Chem. Soc.* **1997**, *119*, 3619.

(5) Jang, Y. P.; Matsuda, H.; Itagaki, Y.; Nakanishi, K.; Sparrow, J. R. *J. Biol. Chem.* **2005**, *280*, 39732.

(6) Frisch, M. J.; Trucks, G. W.; Schlegel, H. B.; Scuseria, G. E.; Robb, M. A.; Cheeseman, J. R.; Scalmani, G.; Barone, V.; Petersson, G. A.; Nakatsuji, H.; Li, X.; Caricato, M.; Marenich, A. V.; Bloino, J.; Janesko, B. G.; Gomperts, R.; Mennucci, B.; Hratchian, H. P.; Ortiz, J. V.; Izmaylov, A. F.; Sonnenberg, J. L.; Williams-Young, D.; Ding, F.; Lipparini, F.; Egidi, F.; Goings, J.; Peng, B.; Petrone, A.; Henderson, T.; Ranasinghe, D.; Zakrzewski, V. G.; Gao, J.; Rega, N.; Zheng, G.; Liang, W.; Hada, M.; Ehara, M.; Toyota, K.; Fukuda, R.; Hasegawa, J.; Ishida, M.; Nakajima, T.; Honda, Y.; Kitao, O.; Nakai, H.; Vreven, T.; Throssell, K.; J. A. Montgomery, J.; Peralta, J. E.; Ogliaro, F.; Bearpark, M. J.; Heyd, J. J.; Brothers, E. N.; Kudin, K. N.; Staroverov, V. N.; Keith, T. A.; Kobayashi, R.; Normand, J.; Raghavachari, K.; Rendell, A. P.; Burant, J. C.; Iyengar, S. S.; Tomasi, J.; Cossi, M.; Millam, J. M.; Klene, M.; Adamo, C.; Cammi, R.; Ochterski, J. W.; Martin, R. L.; Morokuma, K.; Farkas, O.; Foresman, J. B.; Fox, D. J. Gaussian 16, Revision C.01, Gaussian Inc., Wallingford, CT. **2016**.

(7) Marenich, A. V.; Cramer, C. J.; Truhlar, D. G. *J. Phys. Chem. B* **2009**, *113*, 6378.

(8) Zhao, Y.; Truhlar, D. G. *Theor. Chem. Acc.* **2008**, *120*, 215.

(9) Papajak, E.; Zheng, J.; Xu, X.; Leverentz, H. R.; Truhlar, D. G. *J. Chem. Theor. Comput.* **2011**, *7*, 3027.

(10) Dennington, R.; Keith, T. A.; Millam, J. M. **2016**. GaussView, v6; Semichem Inc., Shawnee, Mission, KS.

(11) Chai, J.-D.; Head-Gordon, M. *PhysChemChemPhys* **2008**, *10*, 6615.

(12) Weigend, F.; Ahlrichs, R. *Phys. Chem. Chem. Phys.* **2005**, *7*, 3297.

(13) Tomasi, J.; Mennucci, B.; Cammi, R. *Chem. Rev.* **2005**, *105*, 2999.

(14) Legault, C. Y. CYLview20, Université de Sherbroke, **2020**. (http://www.cylview.org)

10. Copies of NMR spectra

^{1}H-NMR (400.16 MHz, C_6D_6)

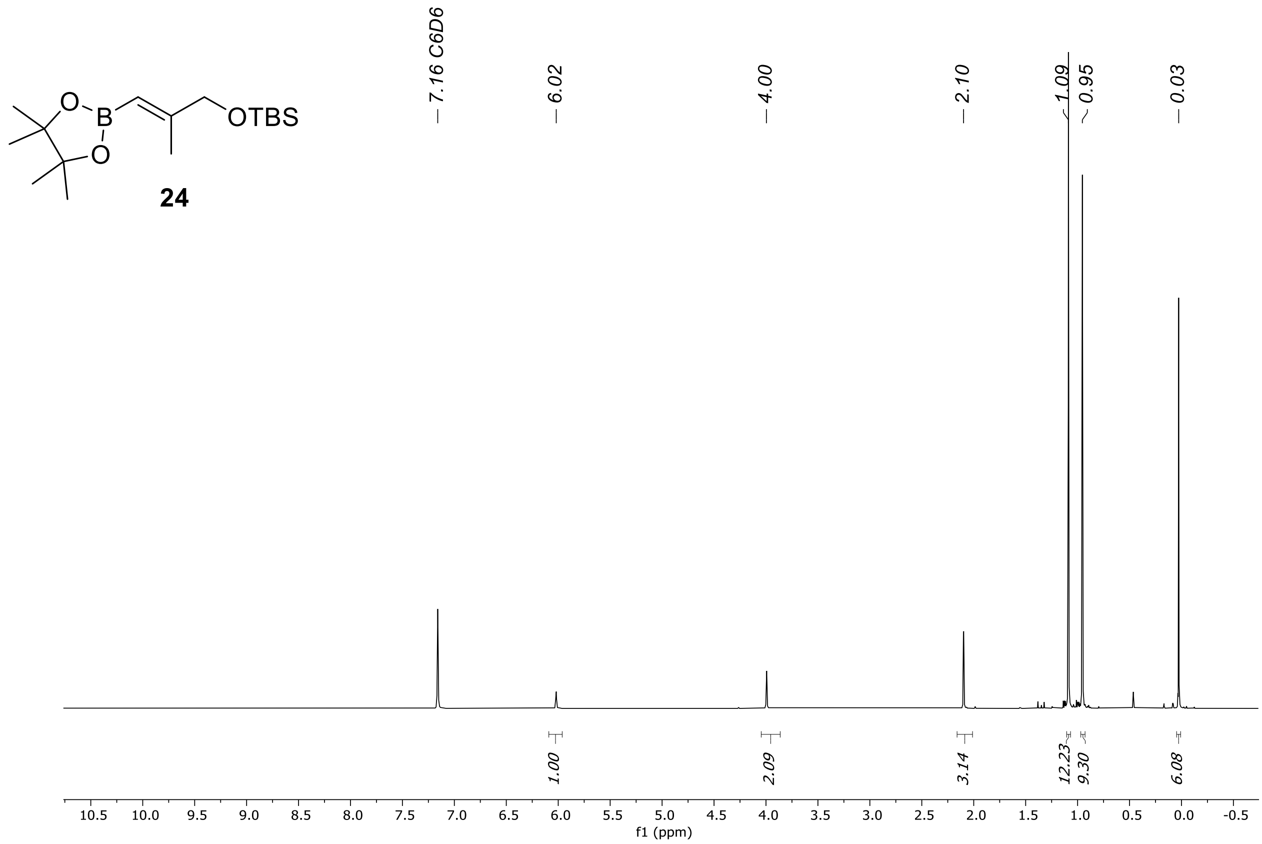

^{13}C{^{1}H}-NMR (100.63 MHz, C_6D_6)

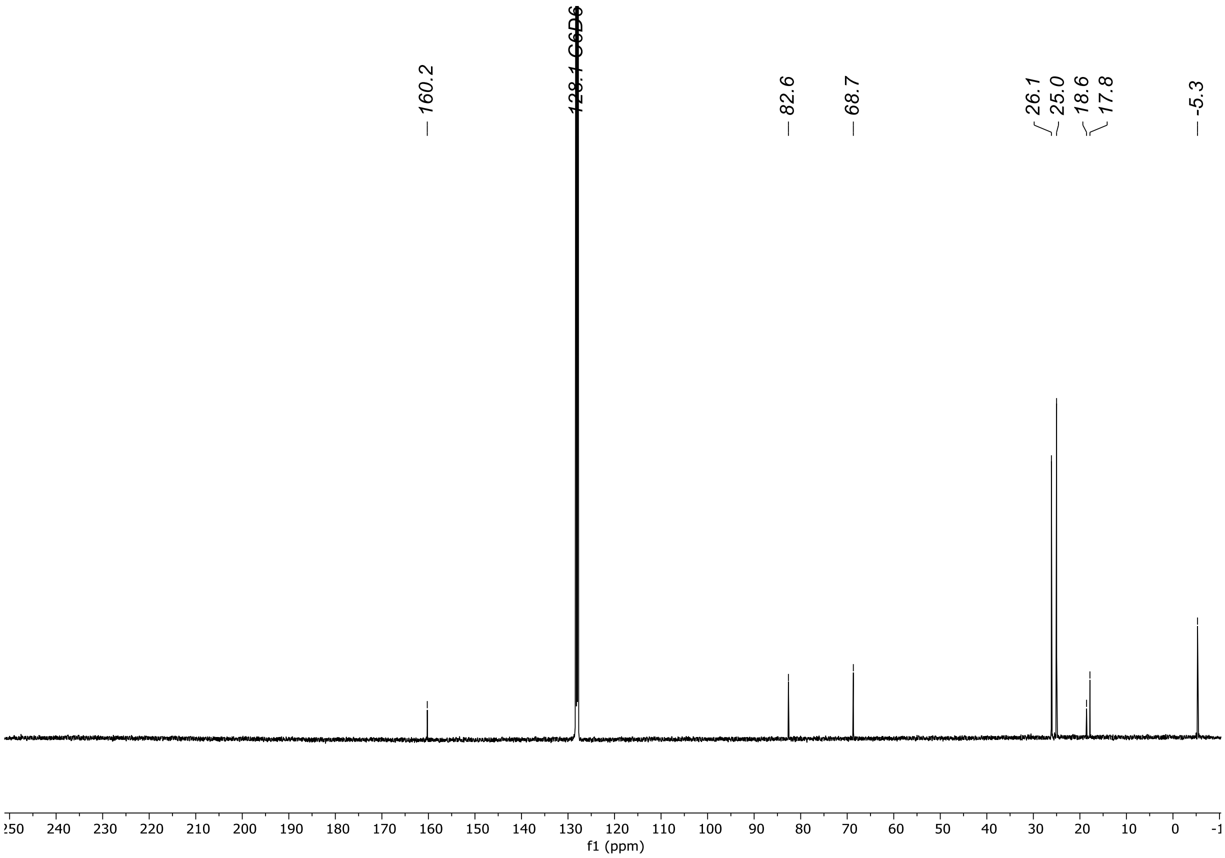

COSY (C_6D_6)

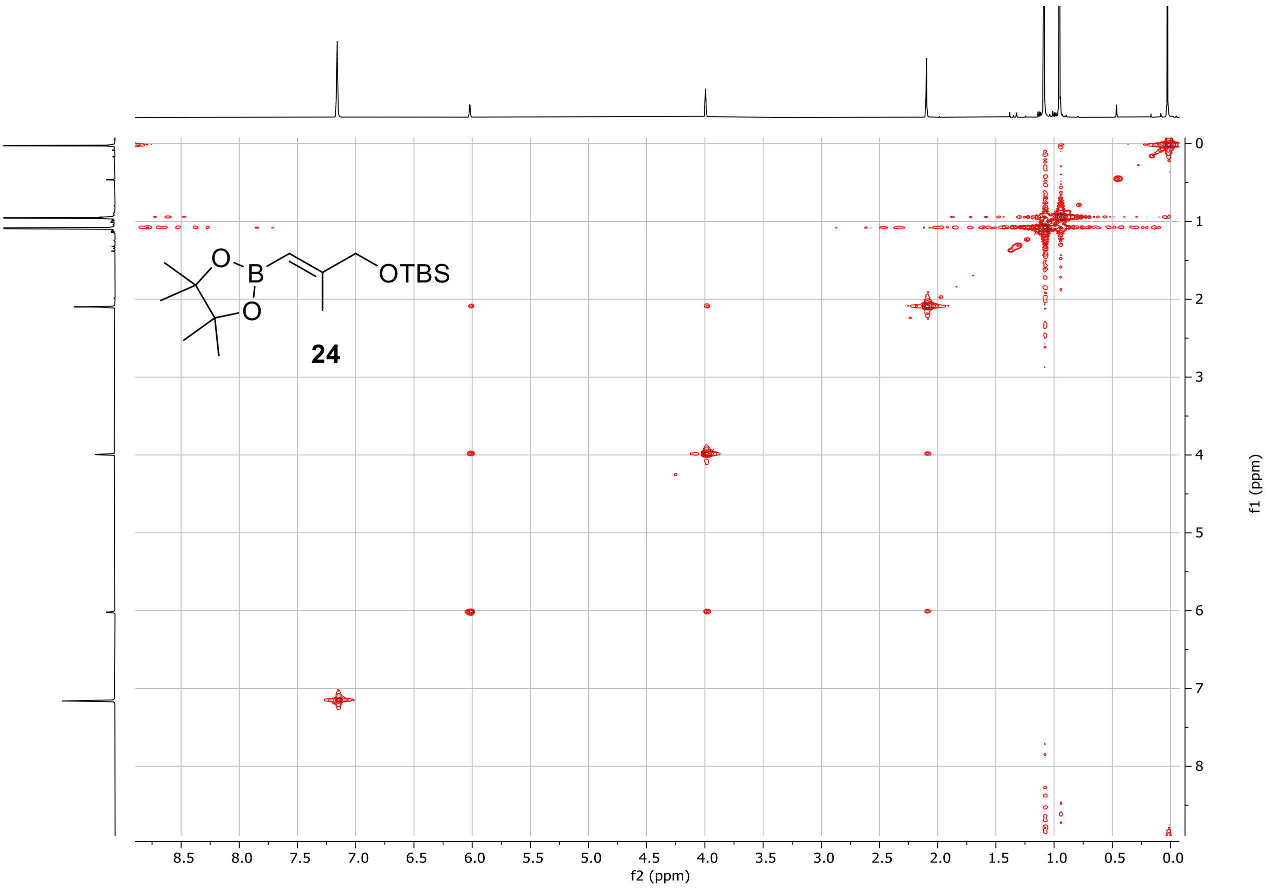

HSQC (C_6D_6)

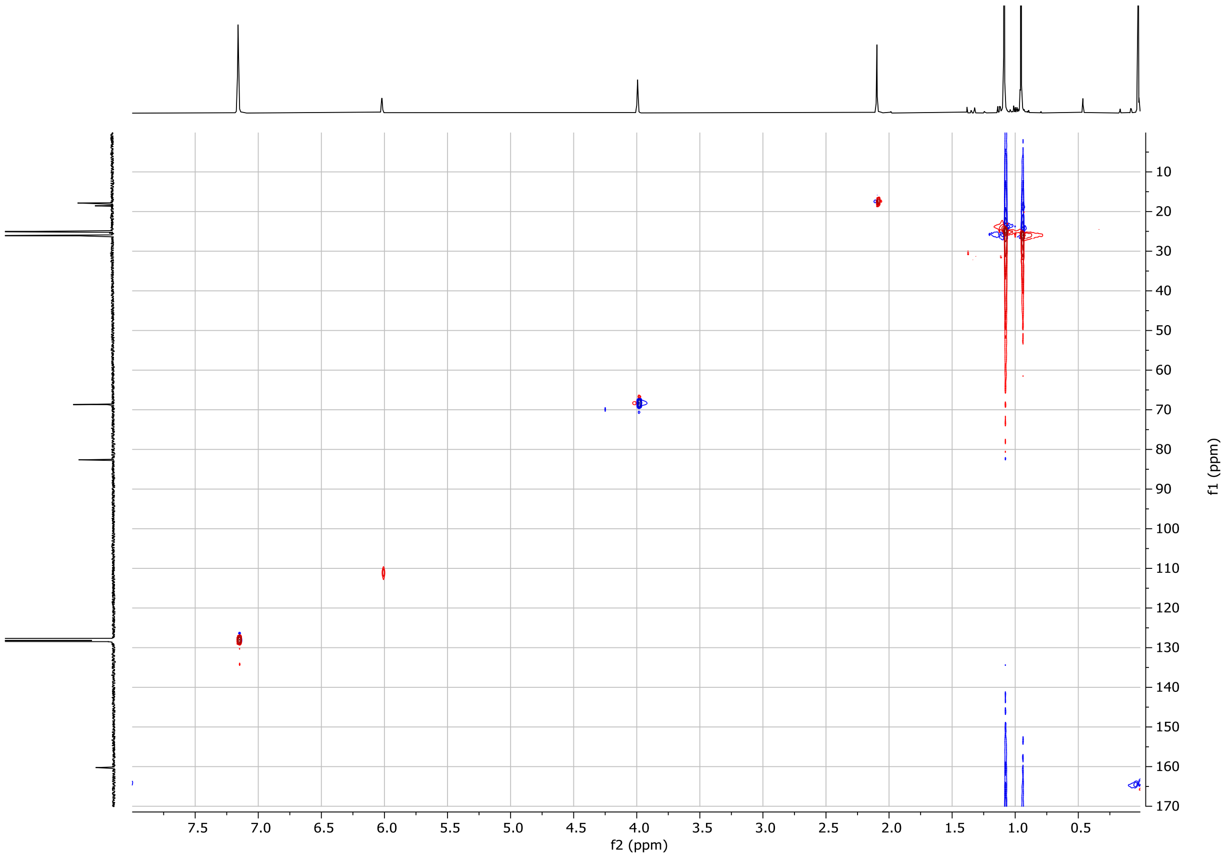

^{1}H-NMR (400.16 MHz, C_6D_6)

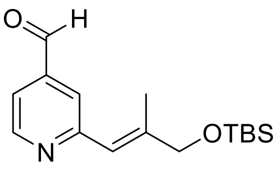

20

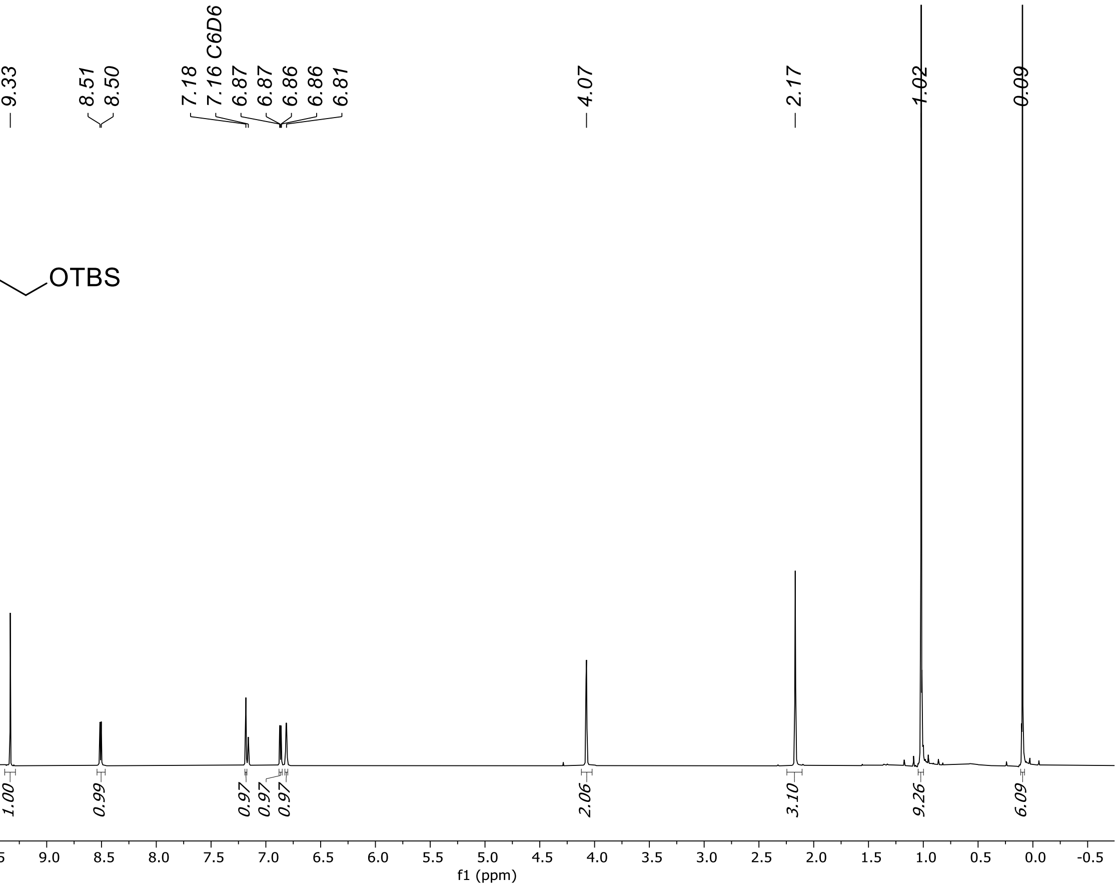

^{13}C{^{1}H}-NMR (100.63 MHz, C_6D_6)

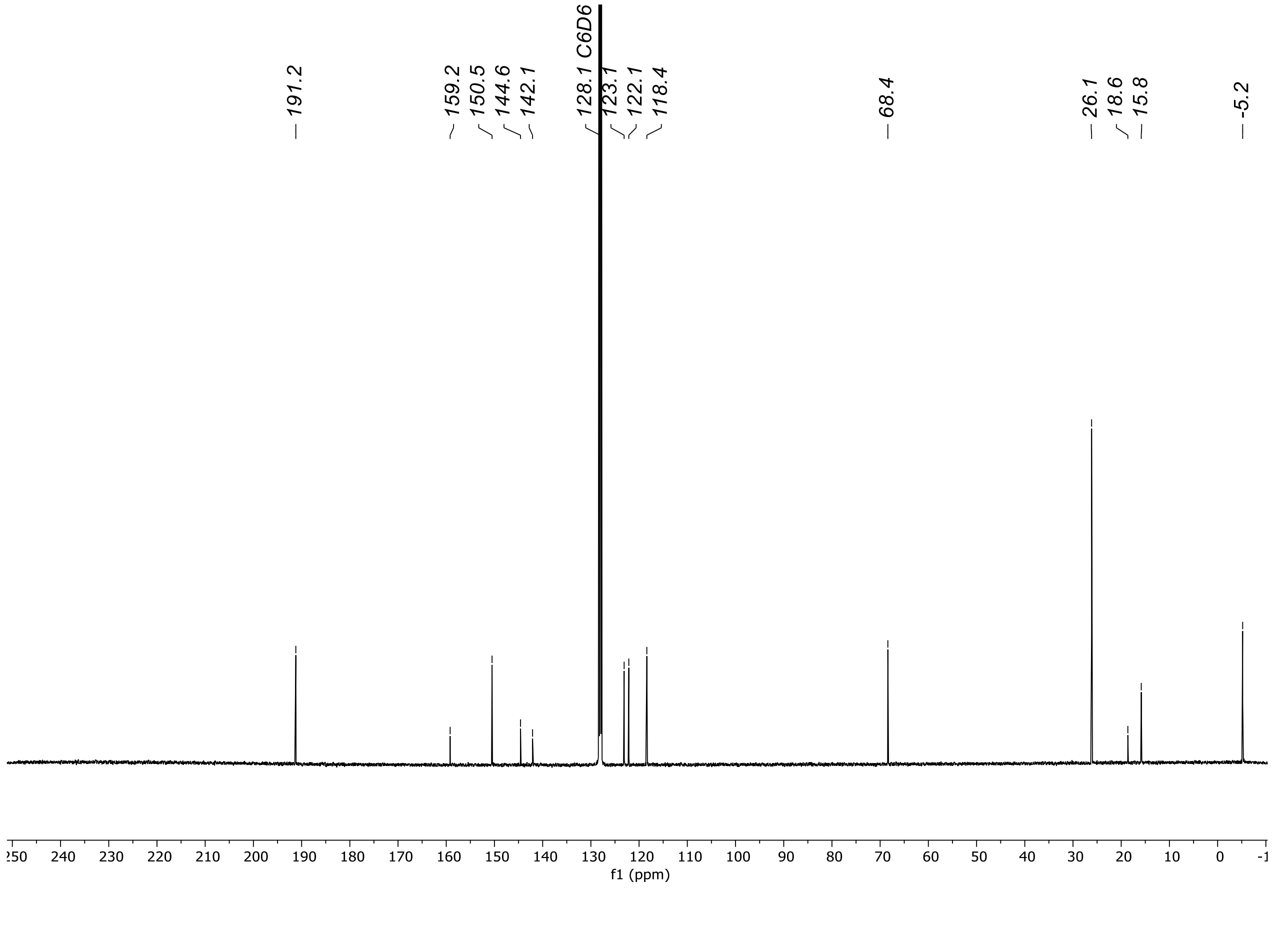

COSY (C_6D_6)

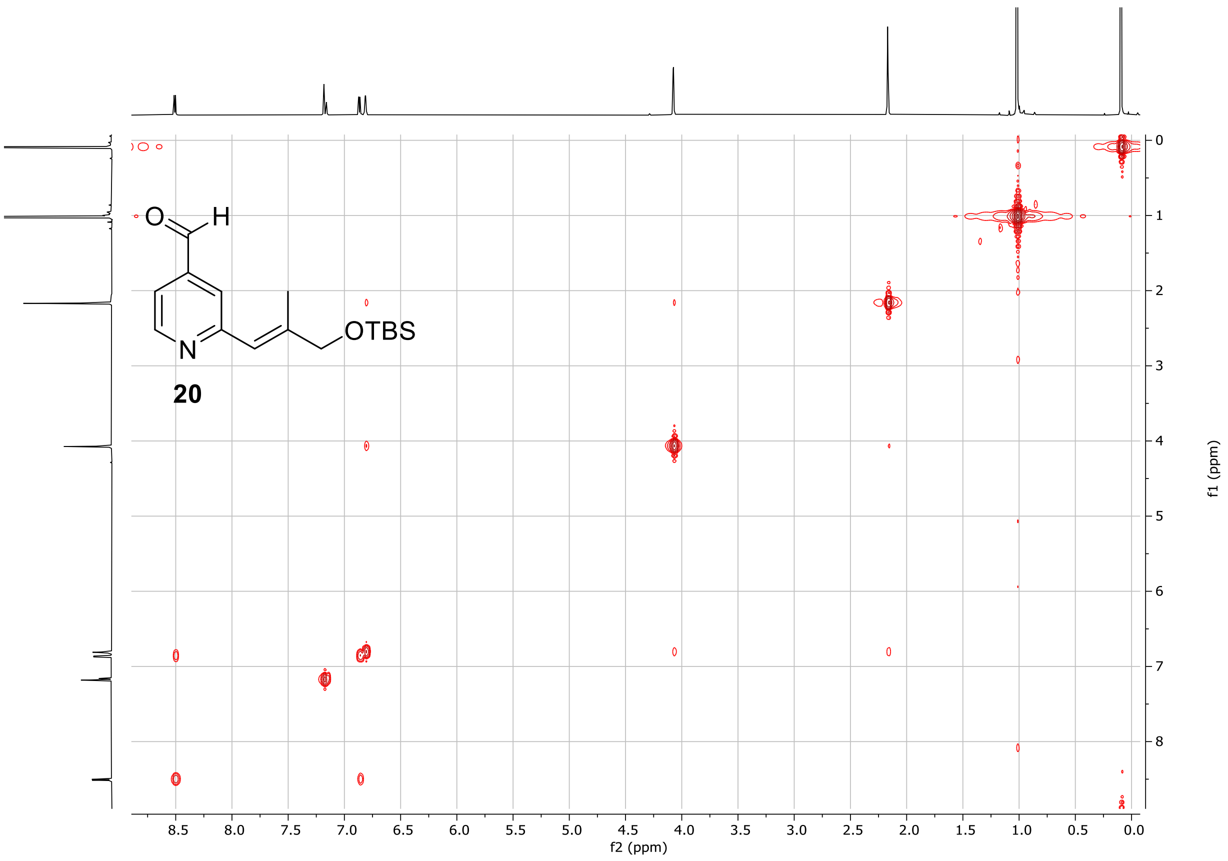

^{1}H-NMR (400.16 MHz, CD_2Cl_2)

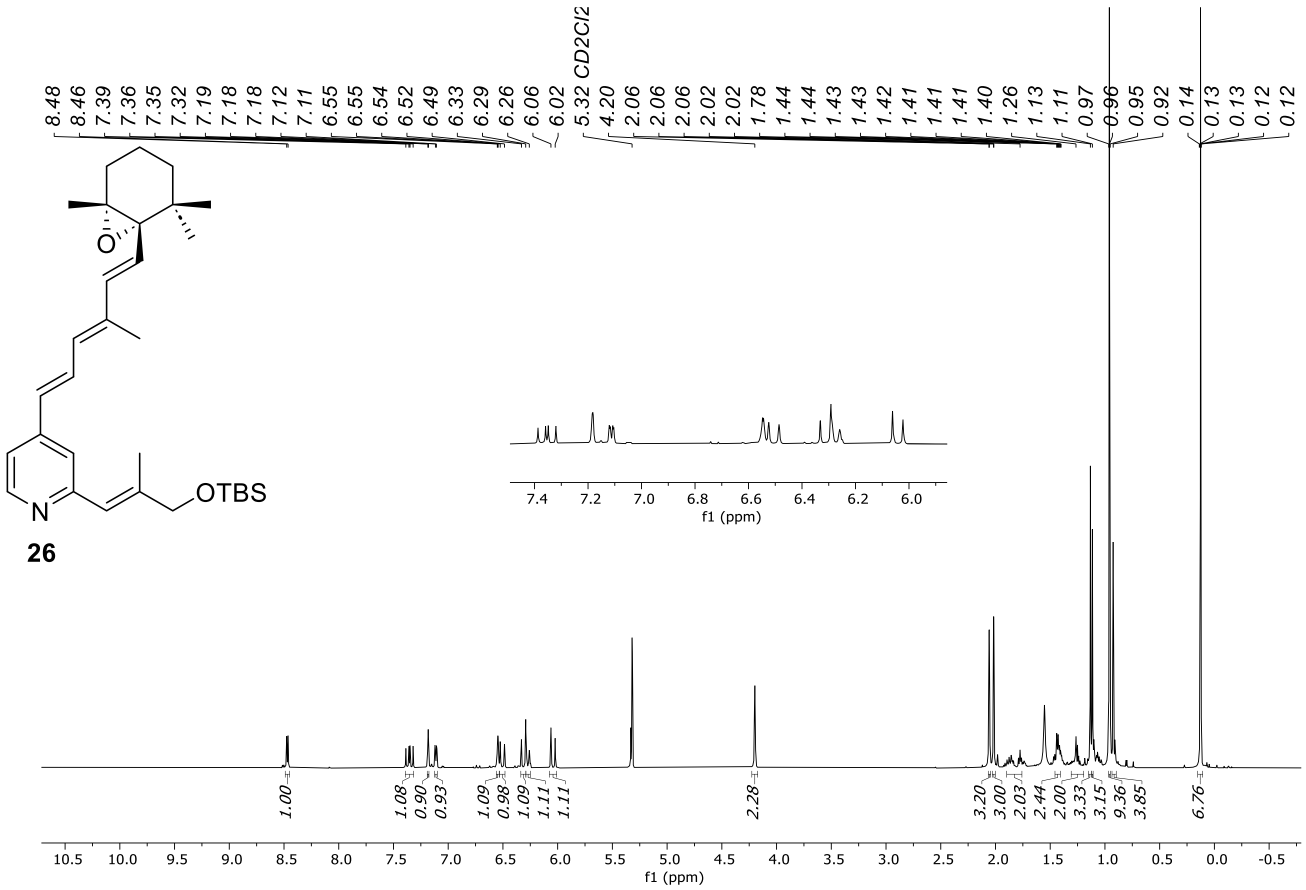

^{13}C{^{1}H}-NMR (100.63 MHz, CD_2Cl_2)

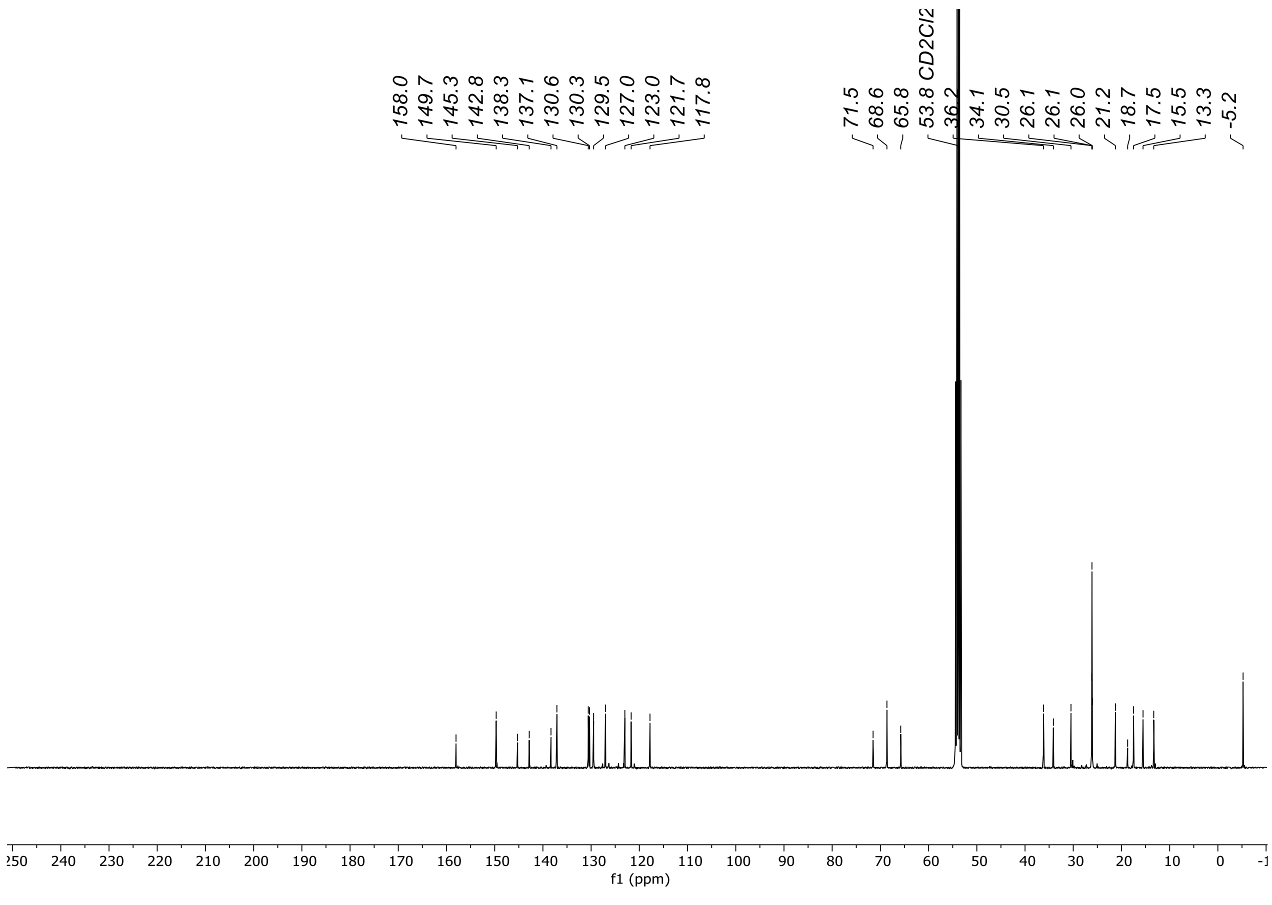

COSY (CD_2Cl_2)

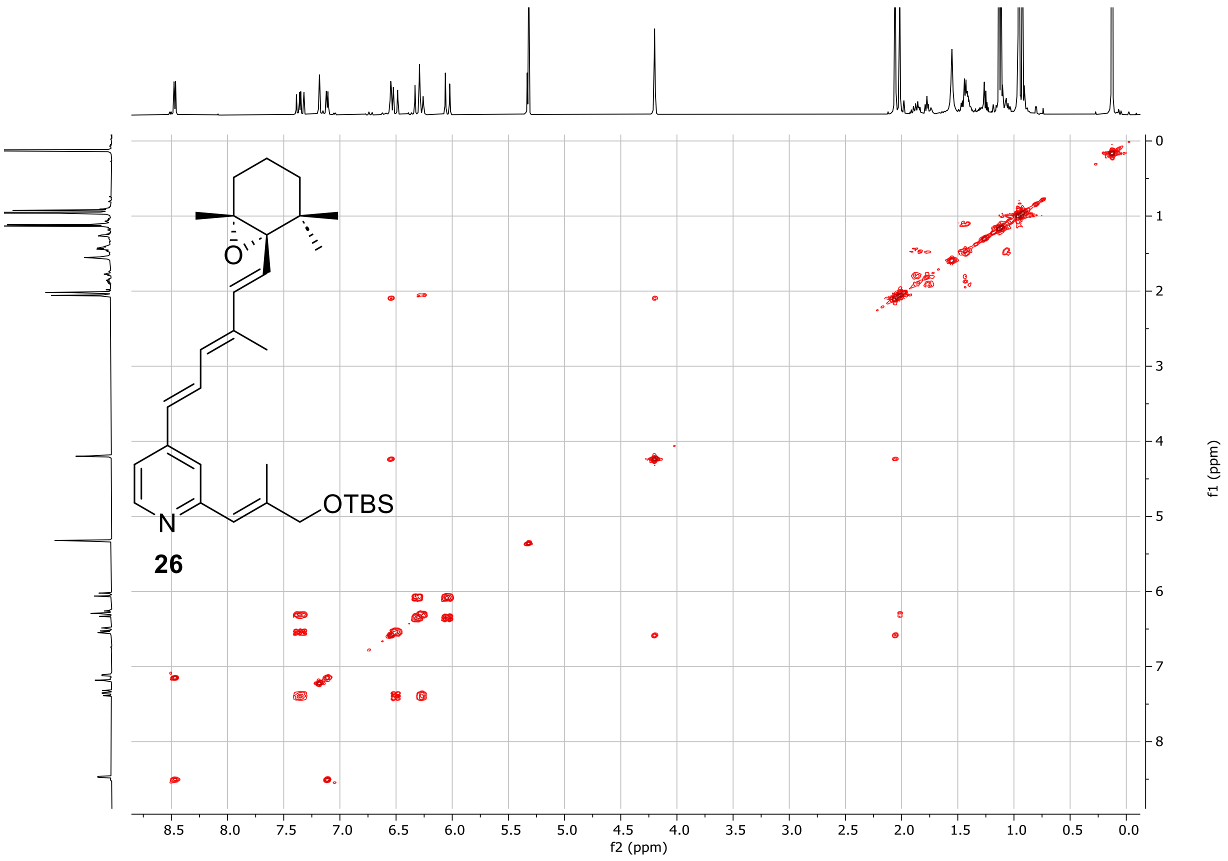

HSQC (CD_2Cl_2)

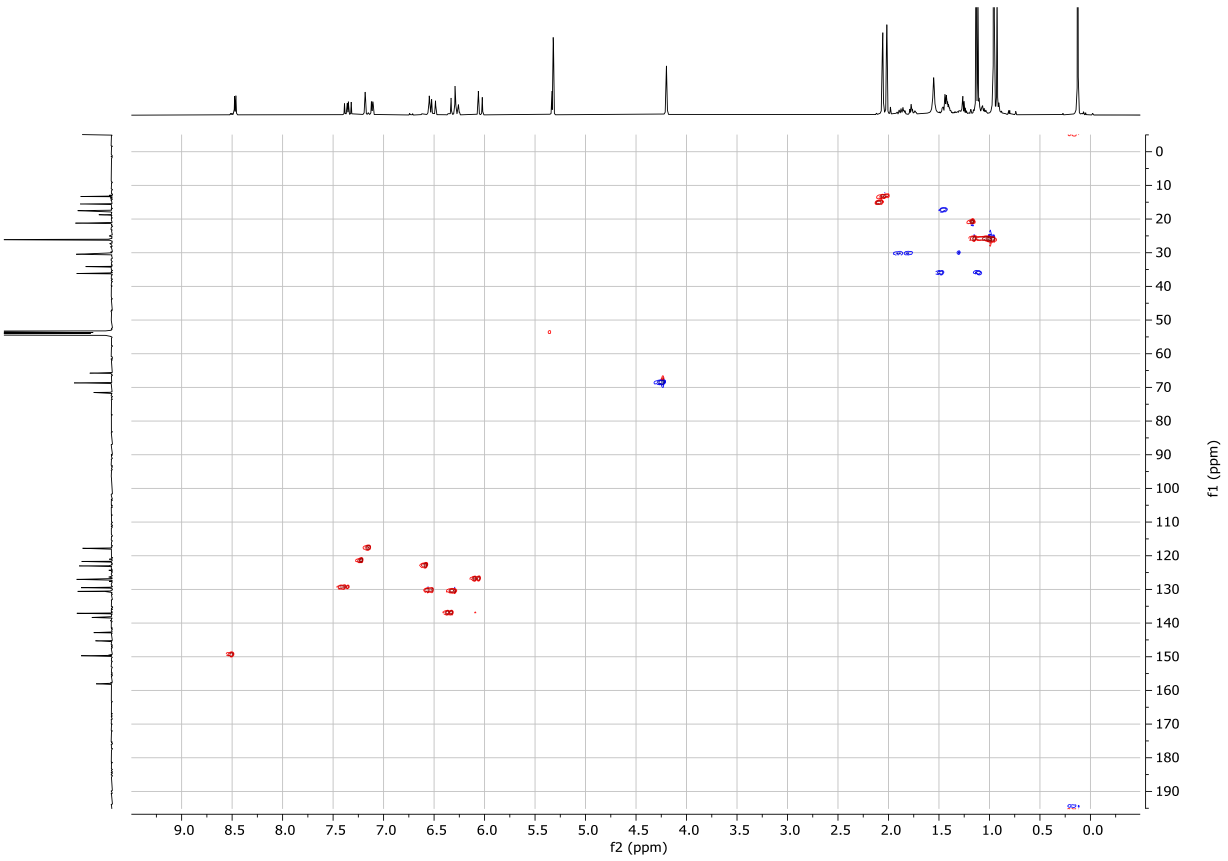

HMBC (CD_2Cl_2)

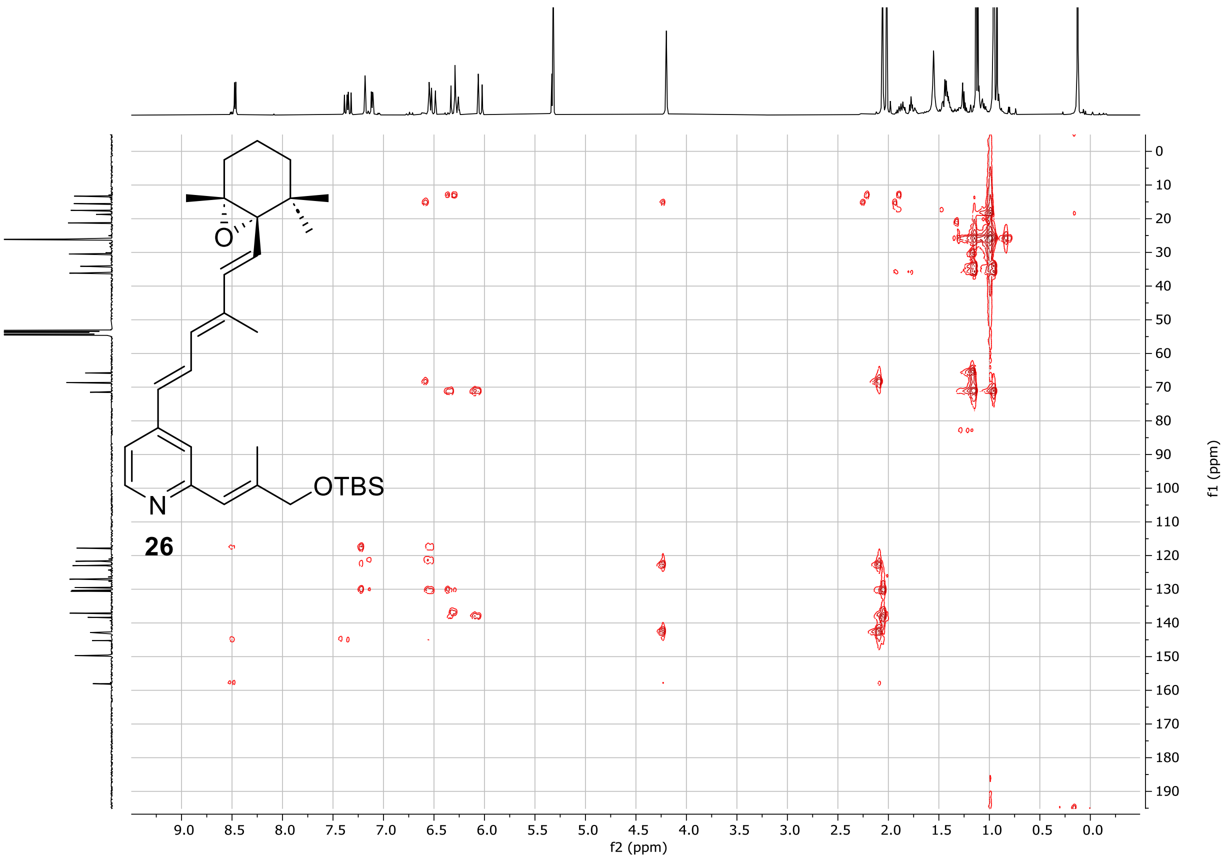

NOE-1D (400.16 MHz, freq. 4.20 ppm, CD_2Cl_2)

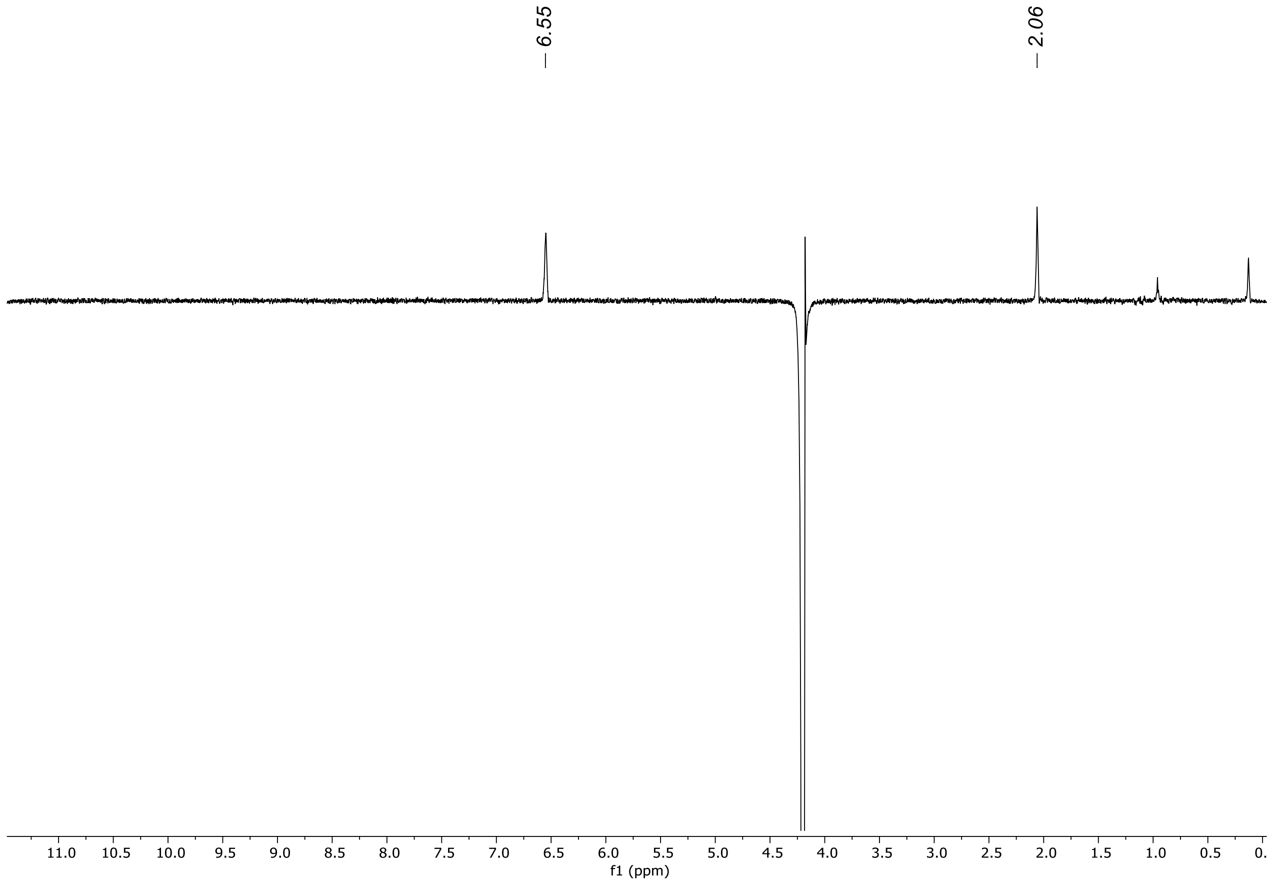

NOE-1D (400.16 MHz, freq. 2.02 ppm, CD_2Cl_2)

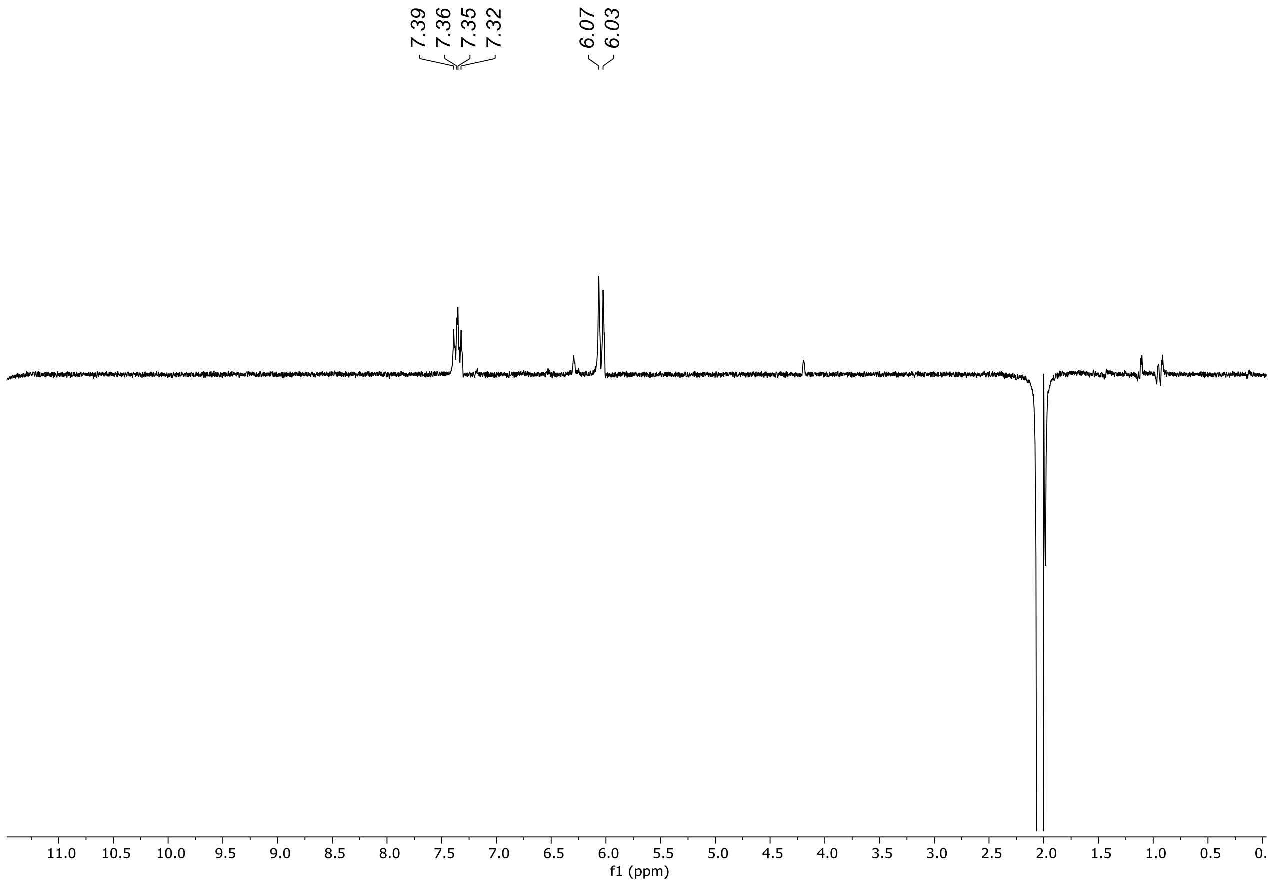

^{1}H-NMR (400.16 MHz, C_6D_6)

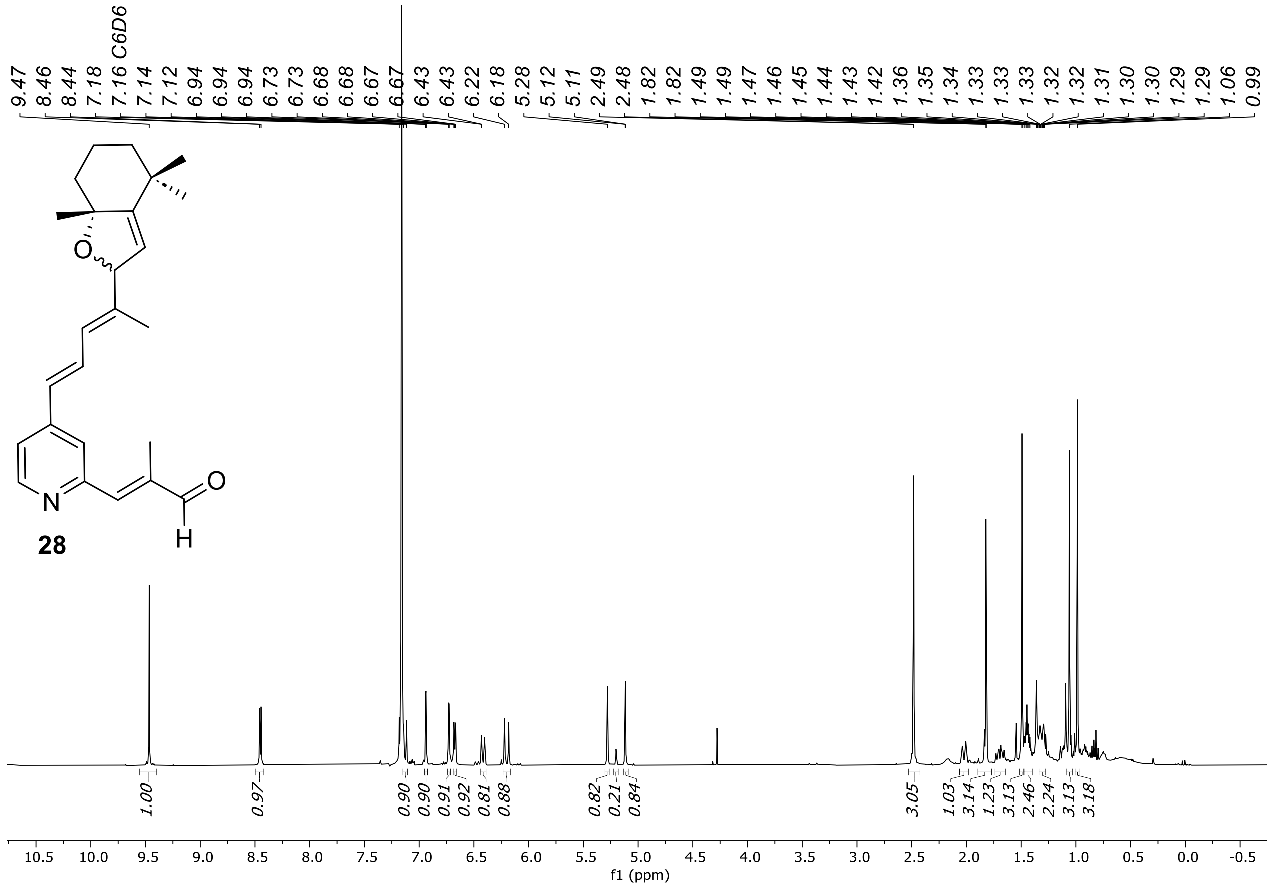

^{1}H-NMR (400.16 MHz, CD_2Cl_2)

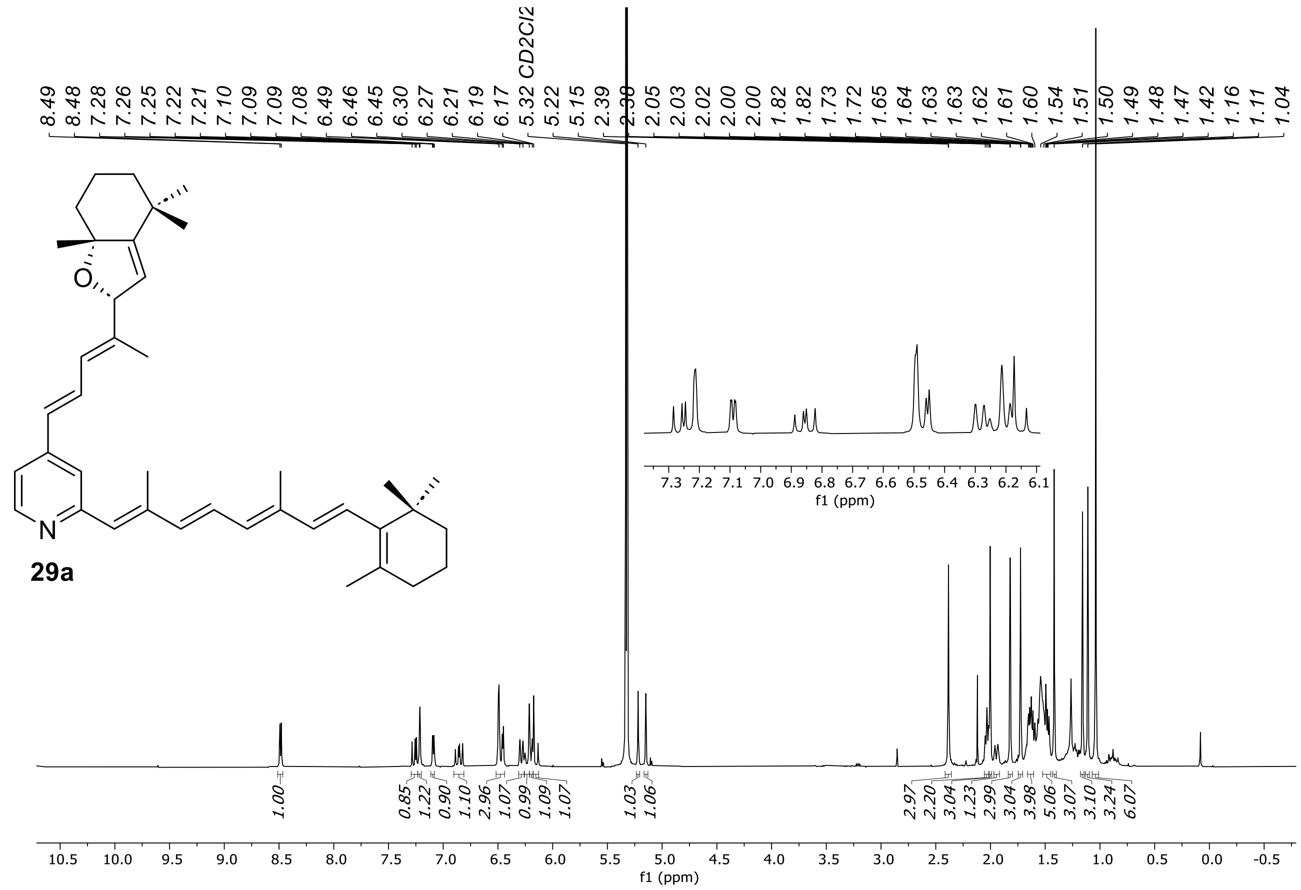

29a

^{13}C{^{1}H}-NMR (100.63 MHz, CD_2Cl_2)

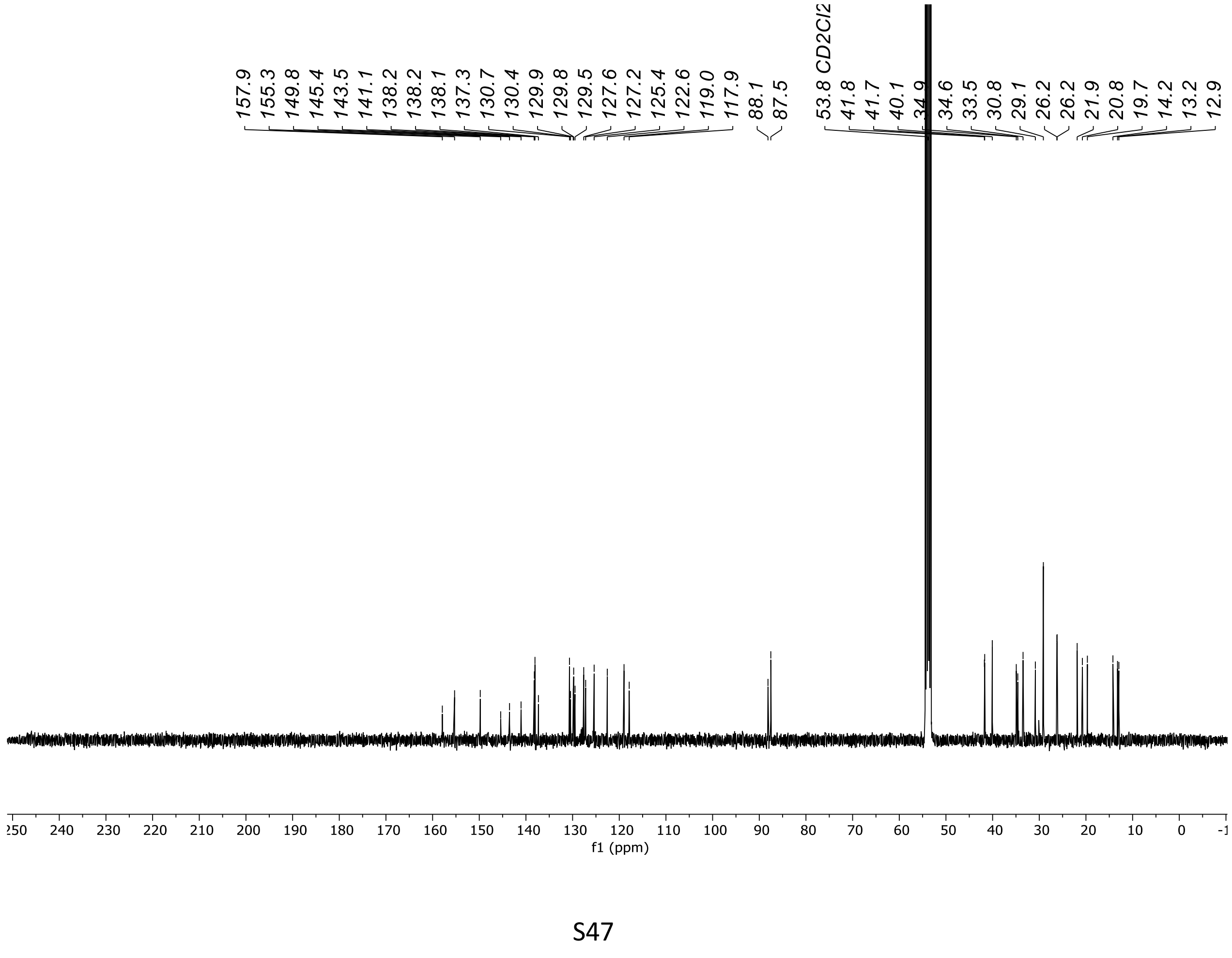

COSY (CD_2Cl_2)

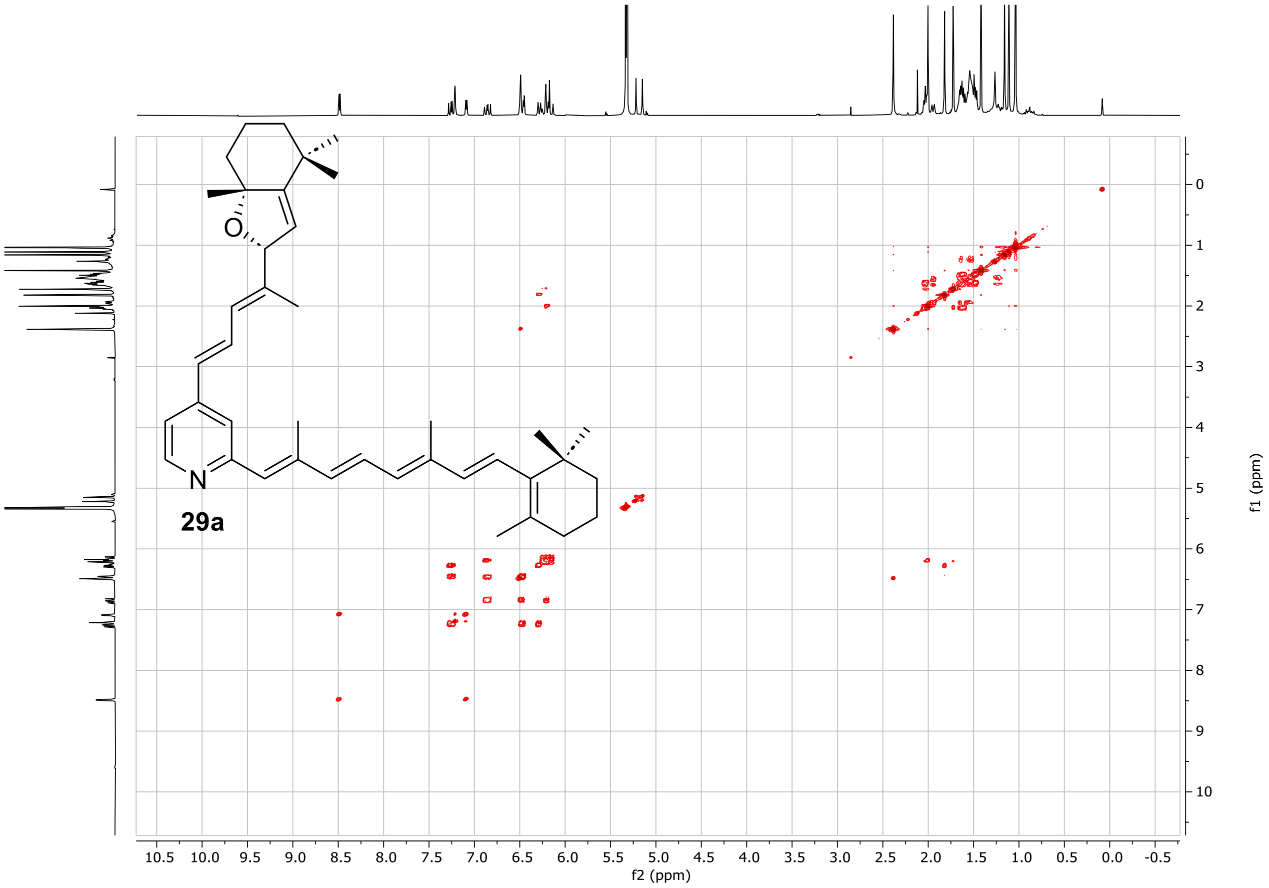

HSQC (CD_2Cl_2)

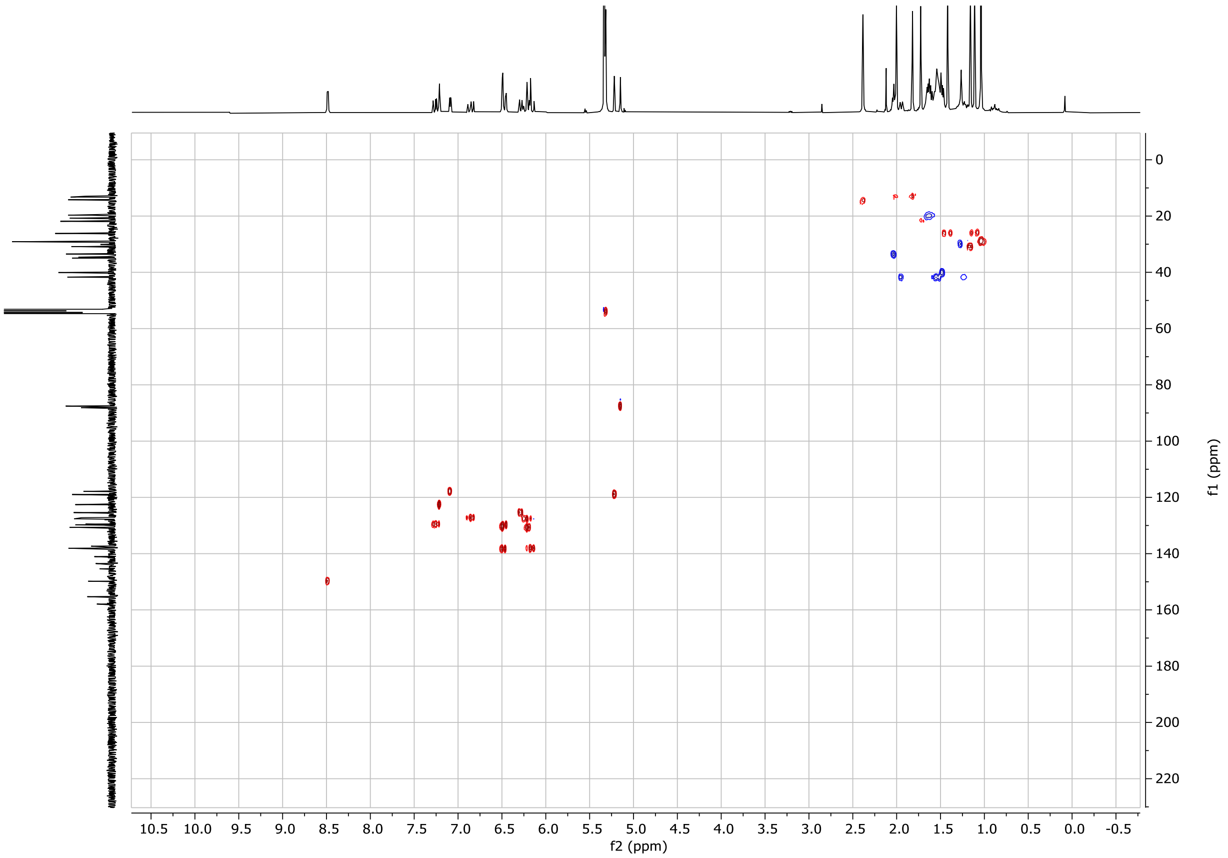

HMBC (CD_2Cl_2)

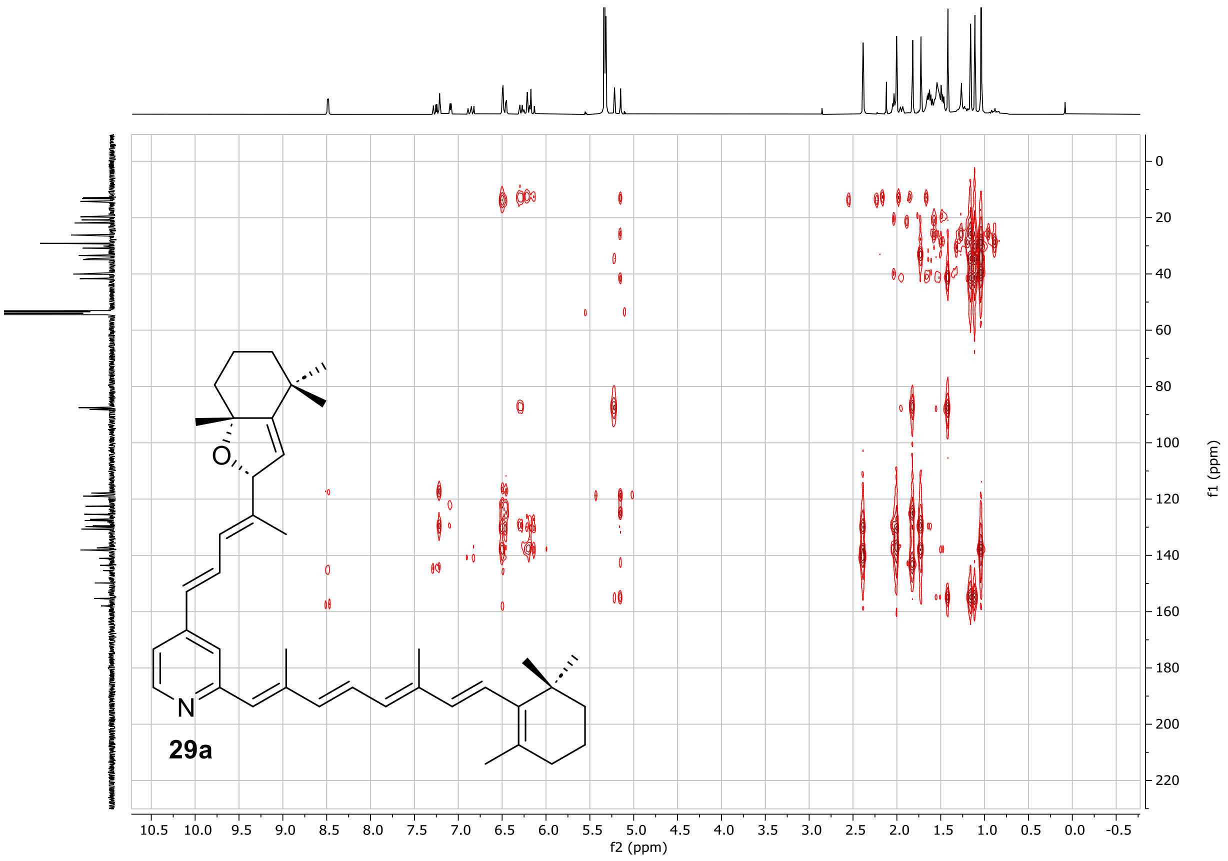

NOE-1D (400.16 MHz, freq. 5.15 ppm, C_6D_6)

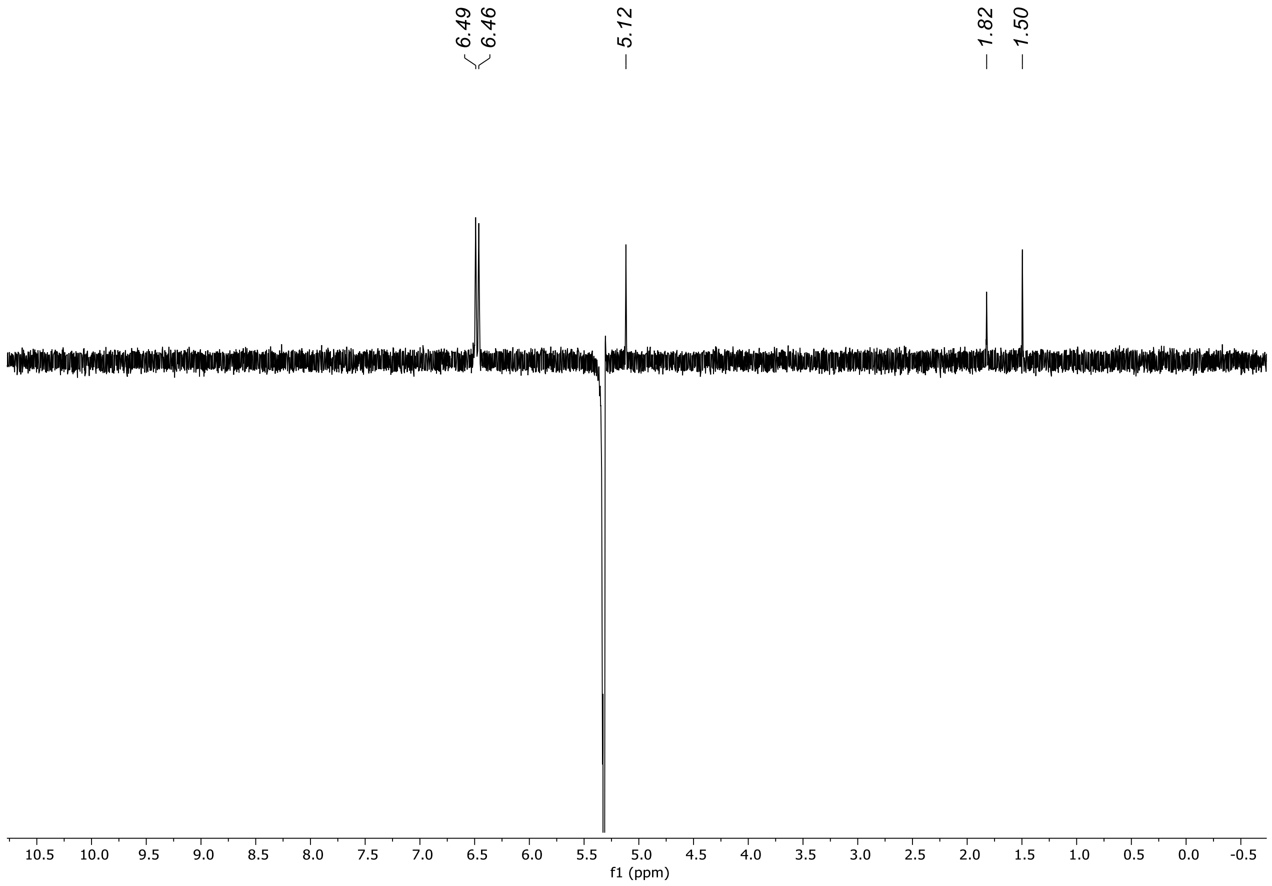

^{1}H-NMR (400.16 MHz, CD_2Cl_2)

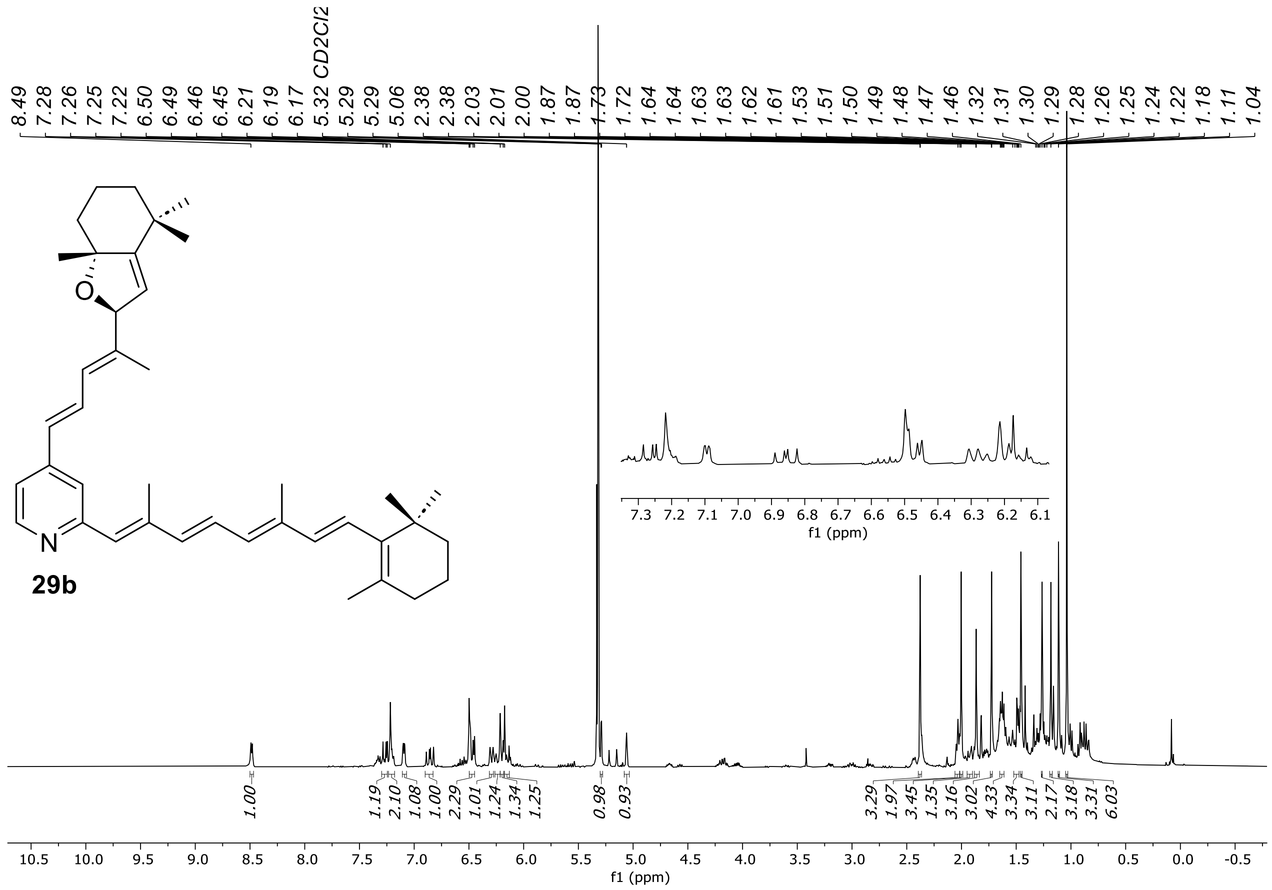

^{13}C{^{1}H}-NMR (100.63 MHz, CD_2Cl_2)

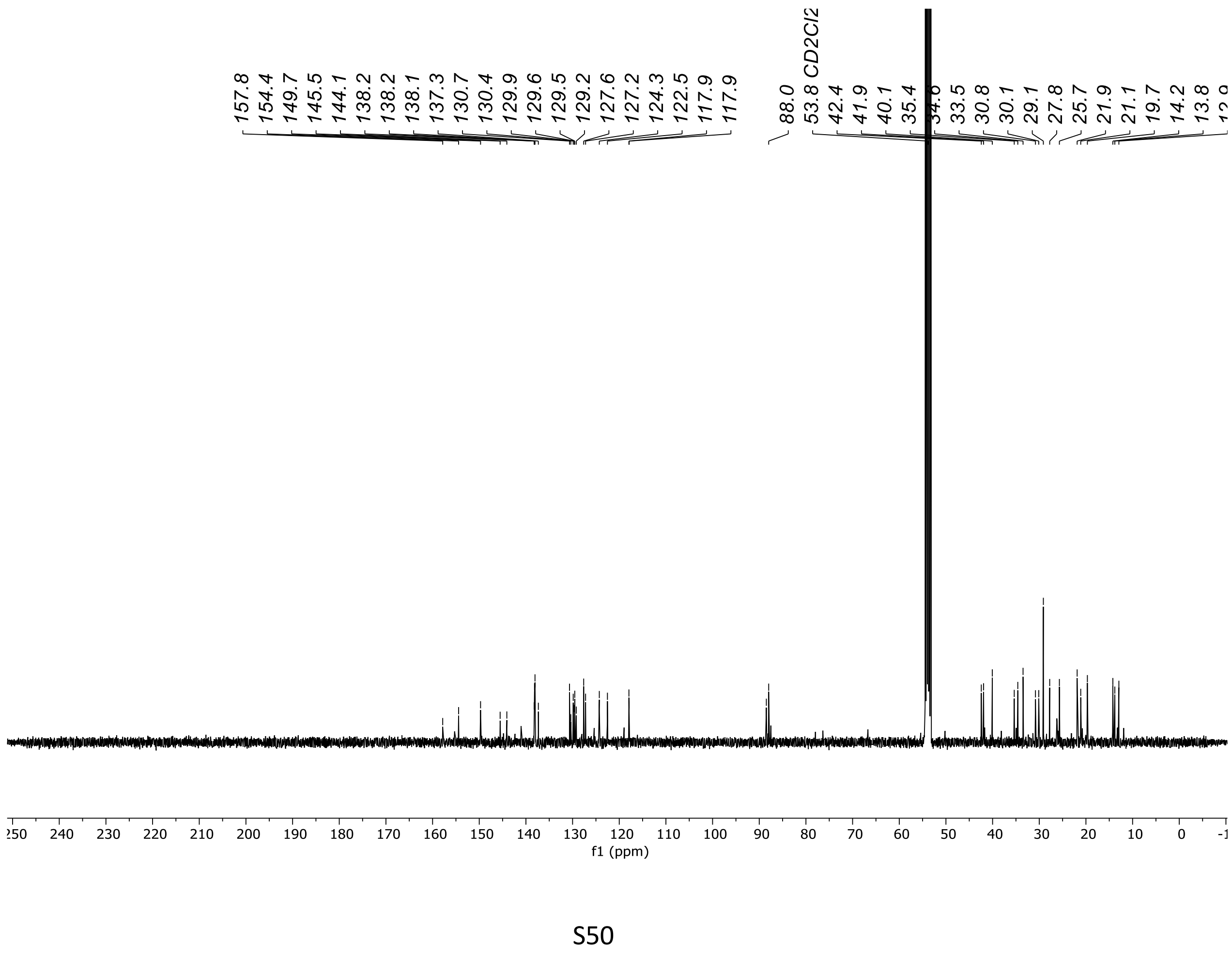

COSY (CD_2Cl_2)

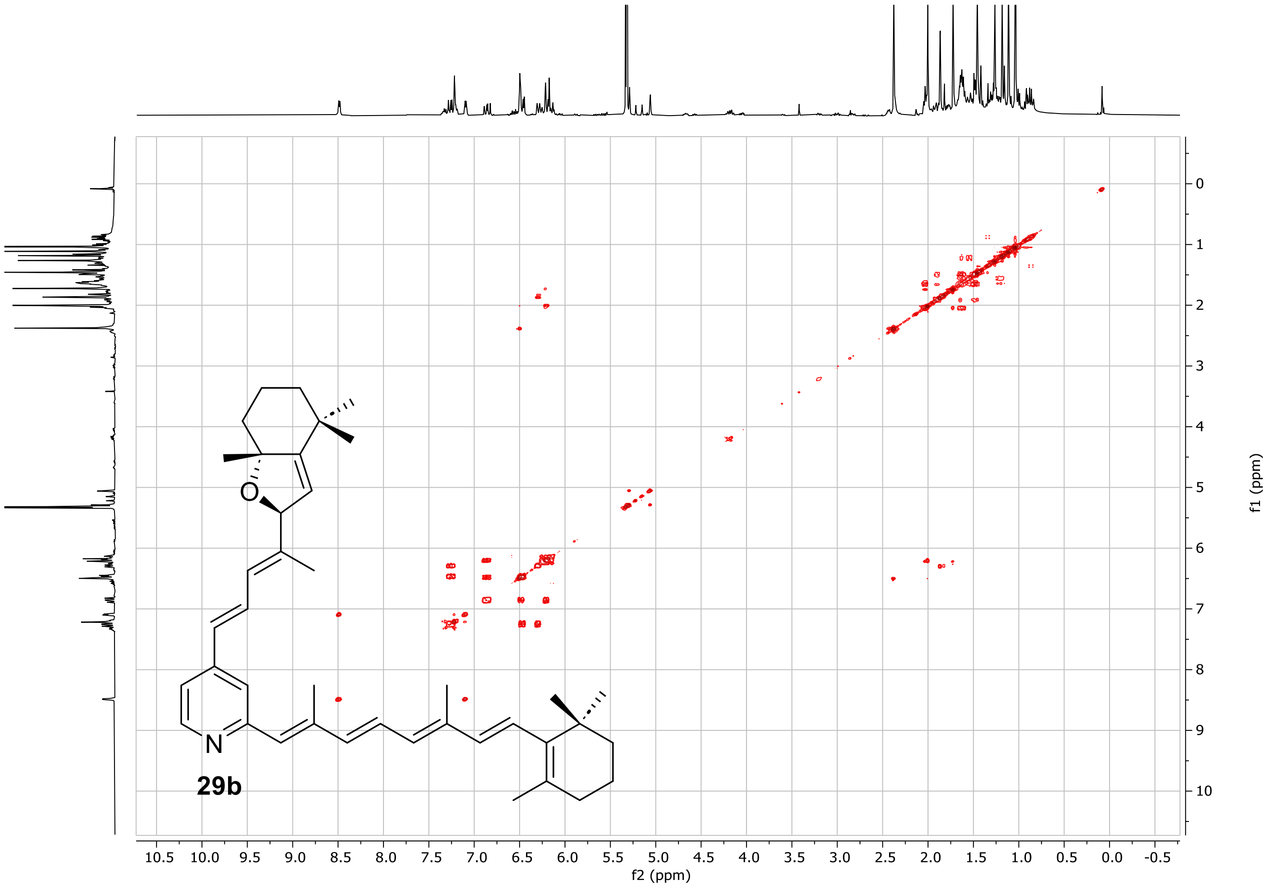

HSQC (CD_2Cl_2)

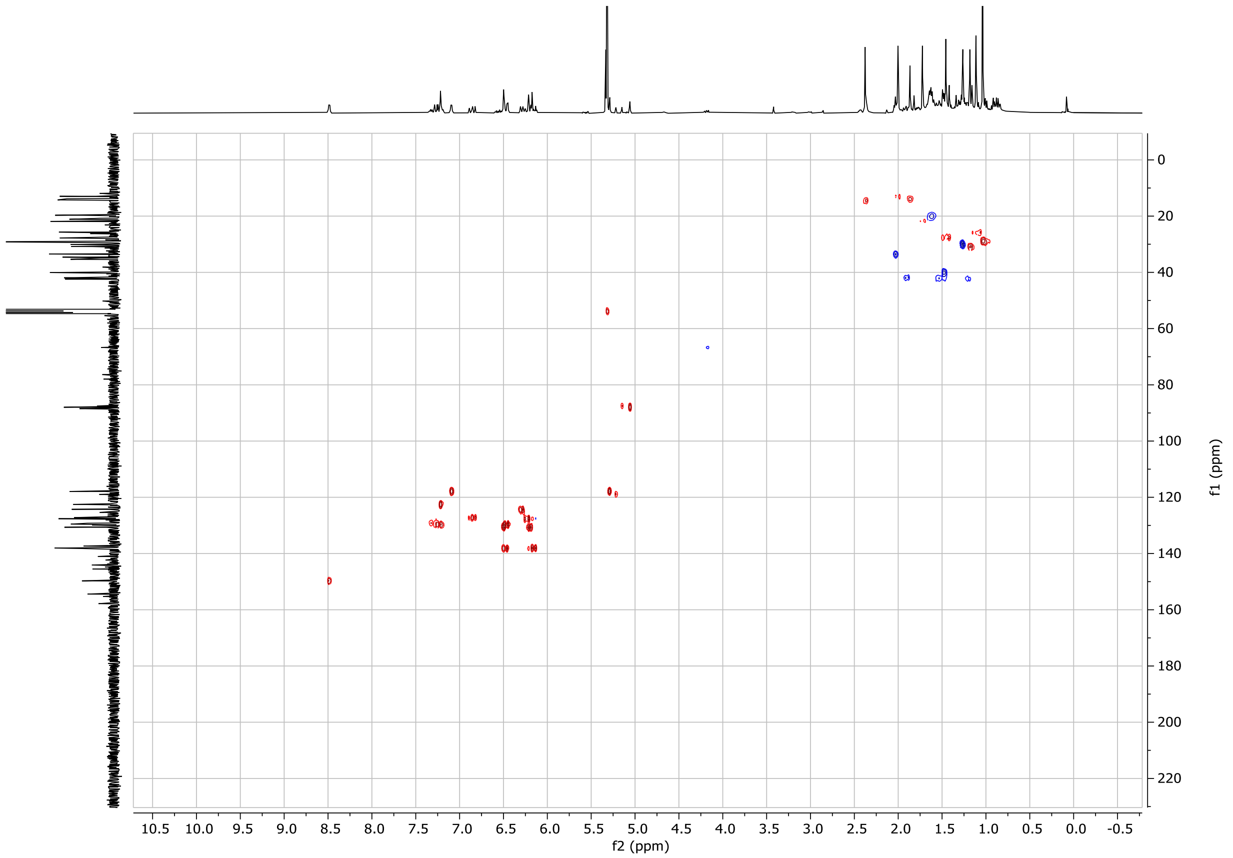

HMBC (CD_2Cl_2)

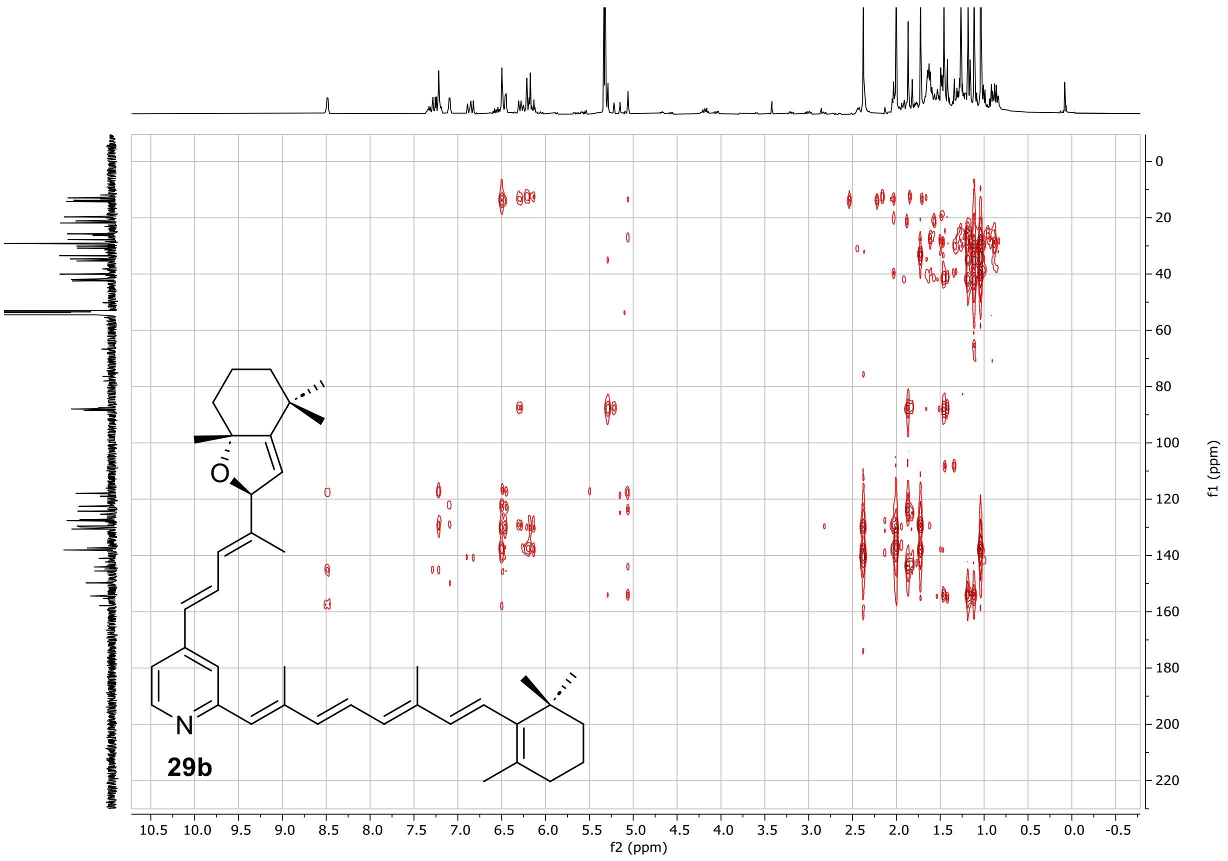

^{1}H-NMR (400.16 MHz, CD_3OD)

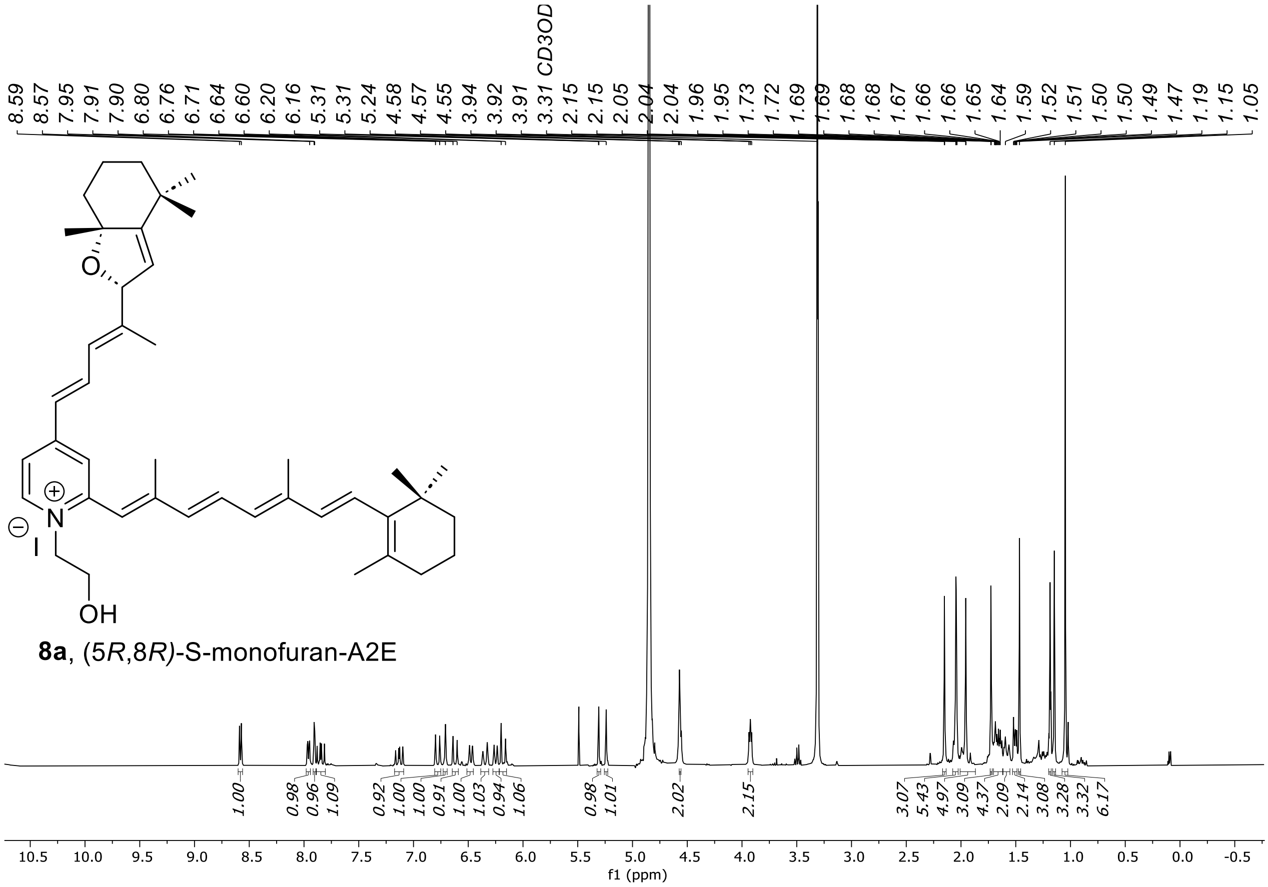

8a, (5*R*,8*R*)-S-monofuran-A2E

^{13}C{^{1}H}-NMR (100.63 MHz, CD_3OD)

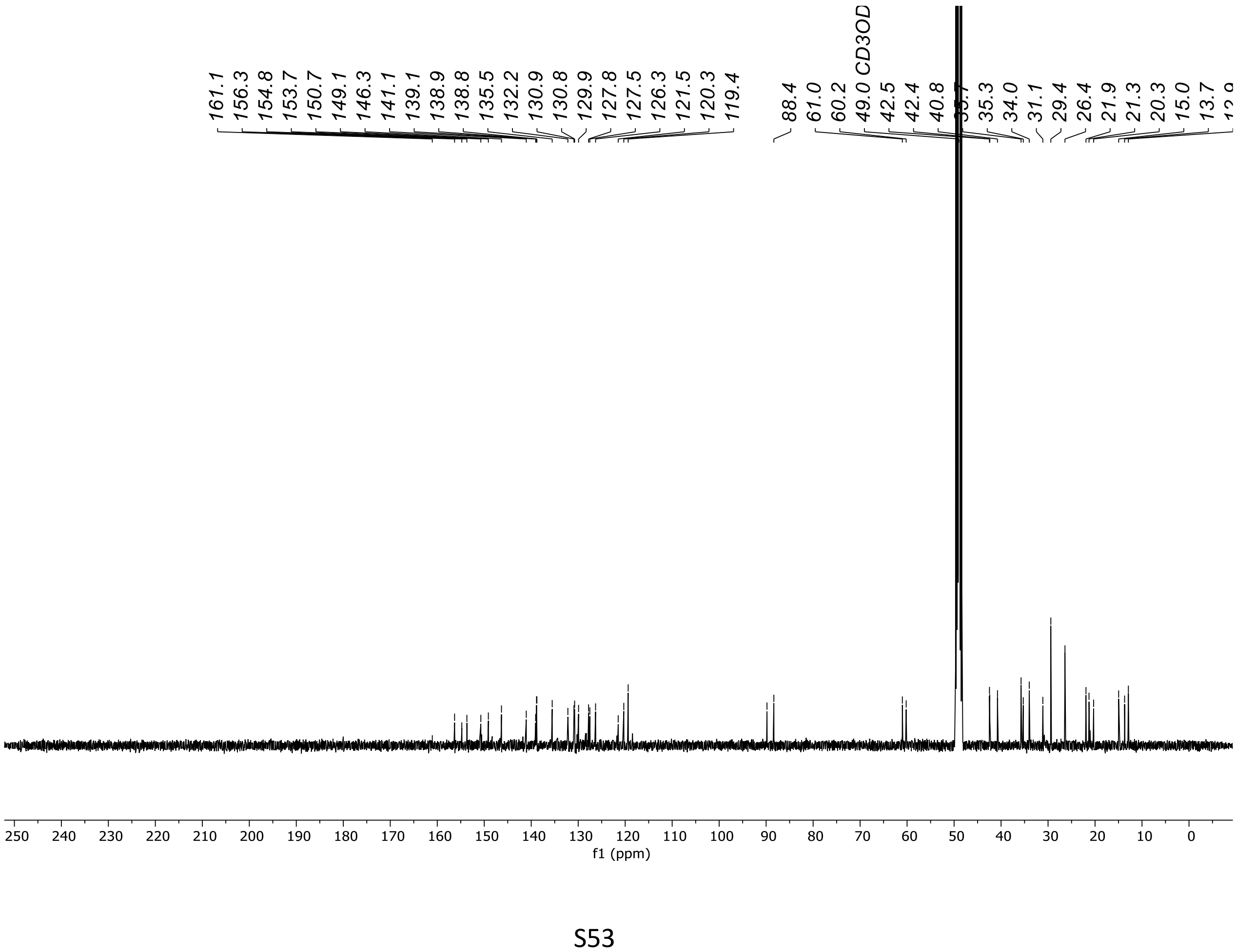

COSY (CD_3OD)

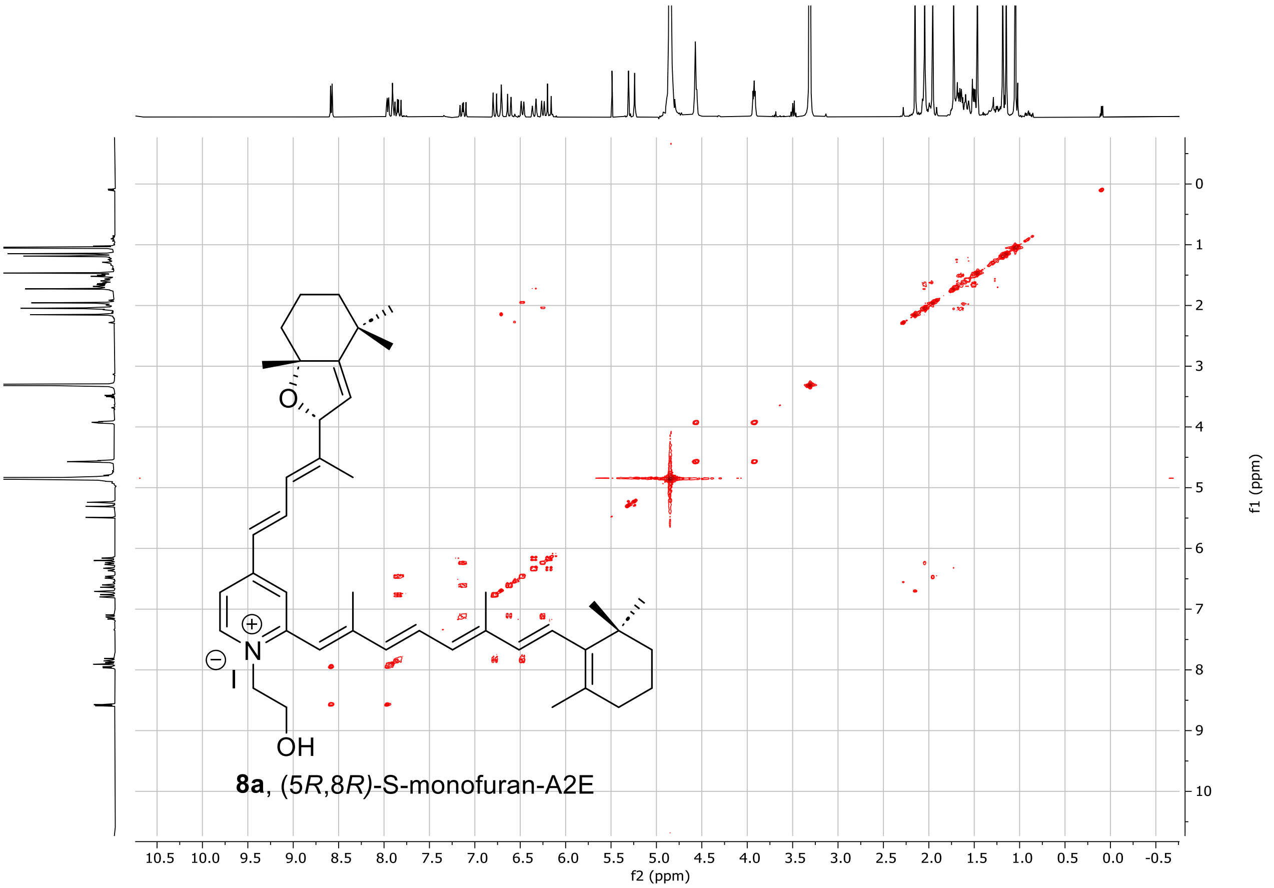

HSQC (CD_3OD)

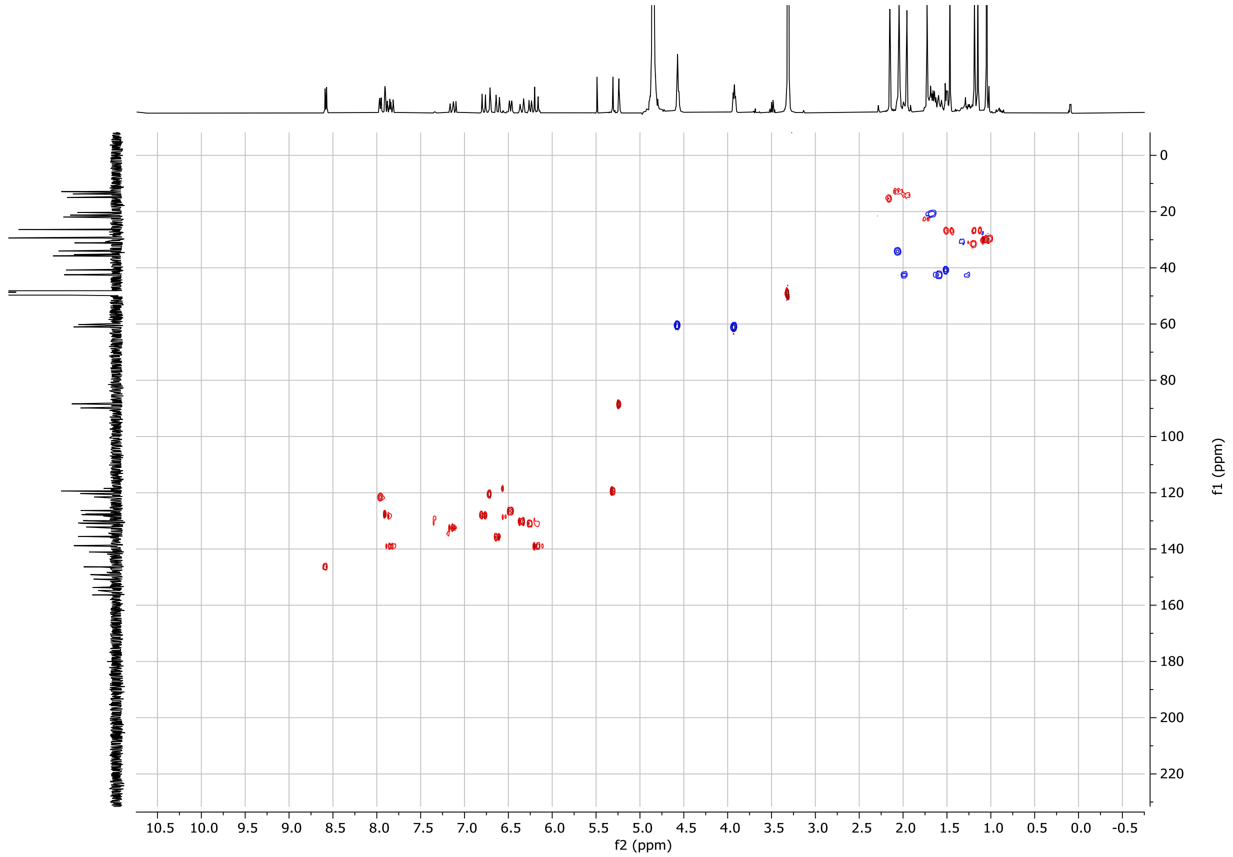

HMBC (CD_3OD)

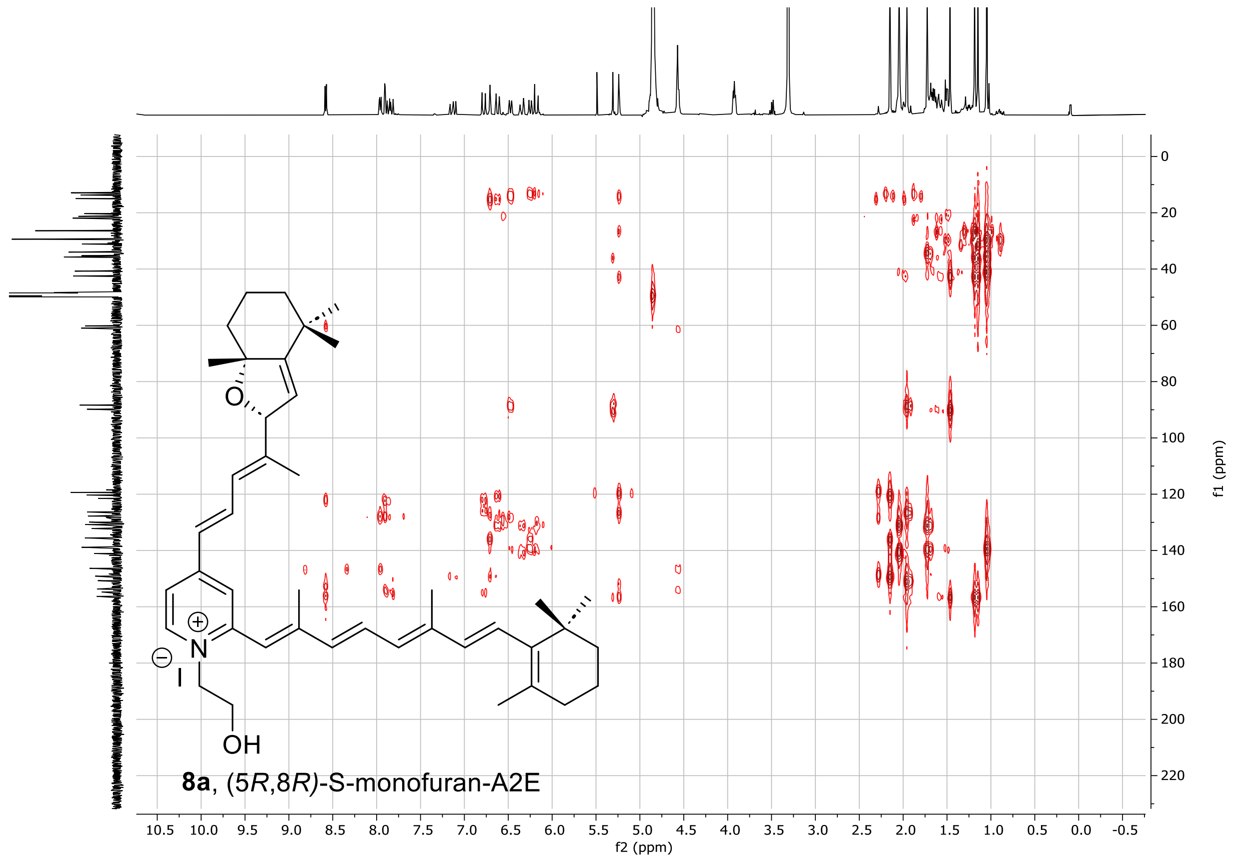

8a, (5*R*,8*R*)-S-monofuran-A2E

NOE-1D (400.16 MHz, freq. 7.13 ppm, CD_3OD)

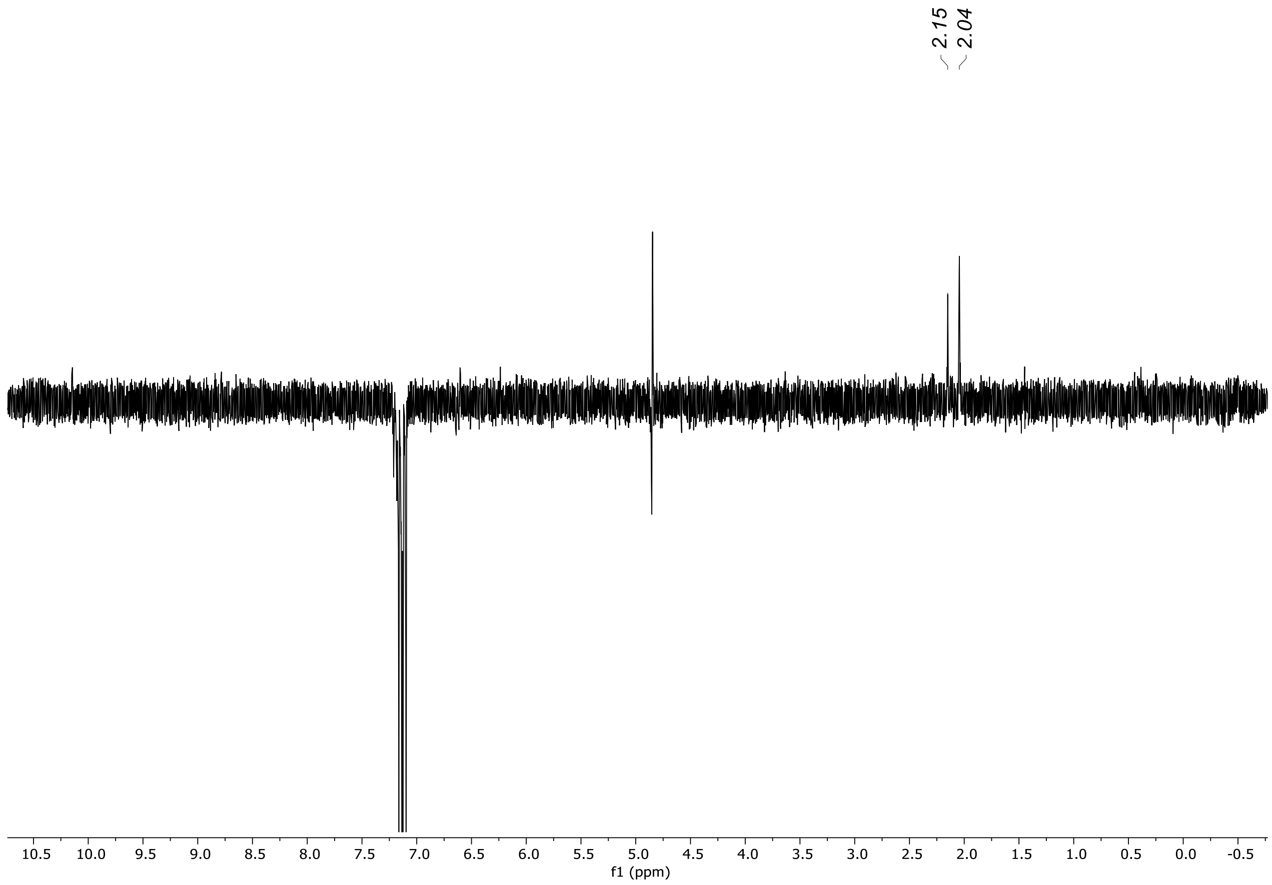

NOE-1D (400.16 MHz, freq. 1.95 ppm, CD_3OD)

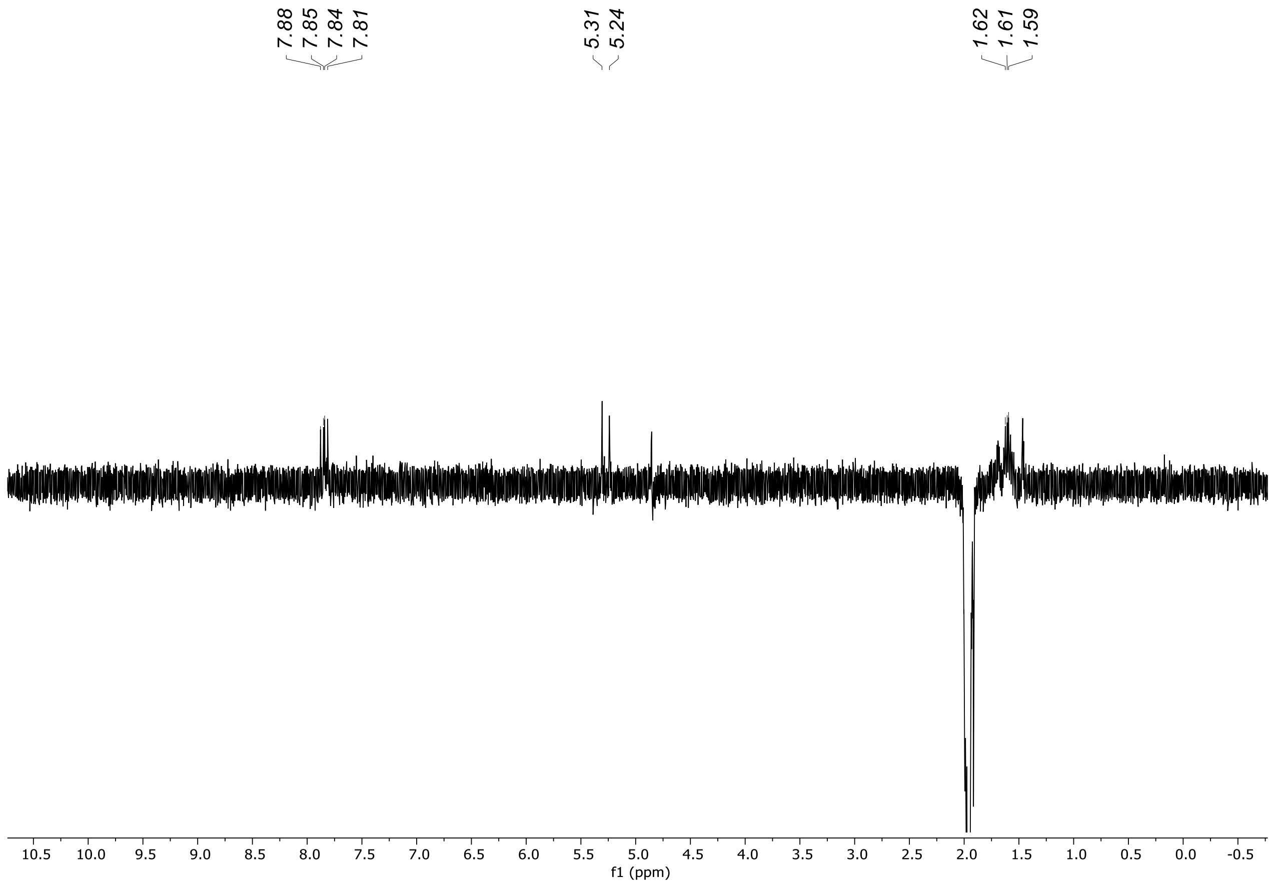

^{1}H-NMR (400.16 MHz, C_6D_6)

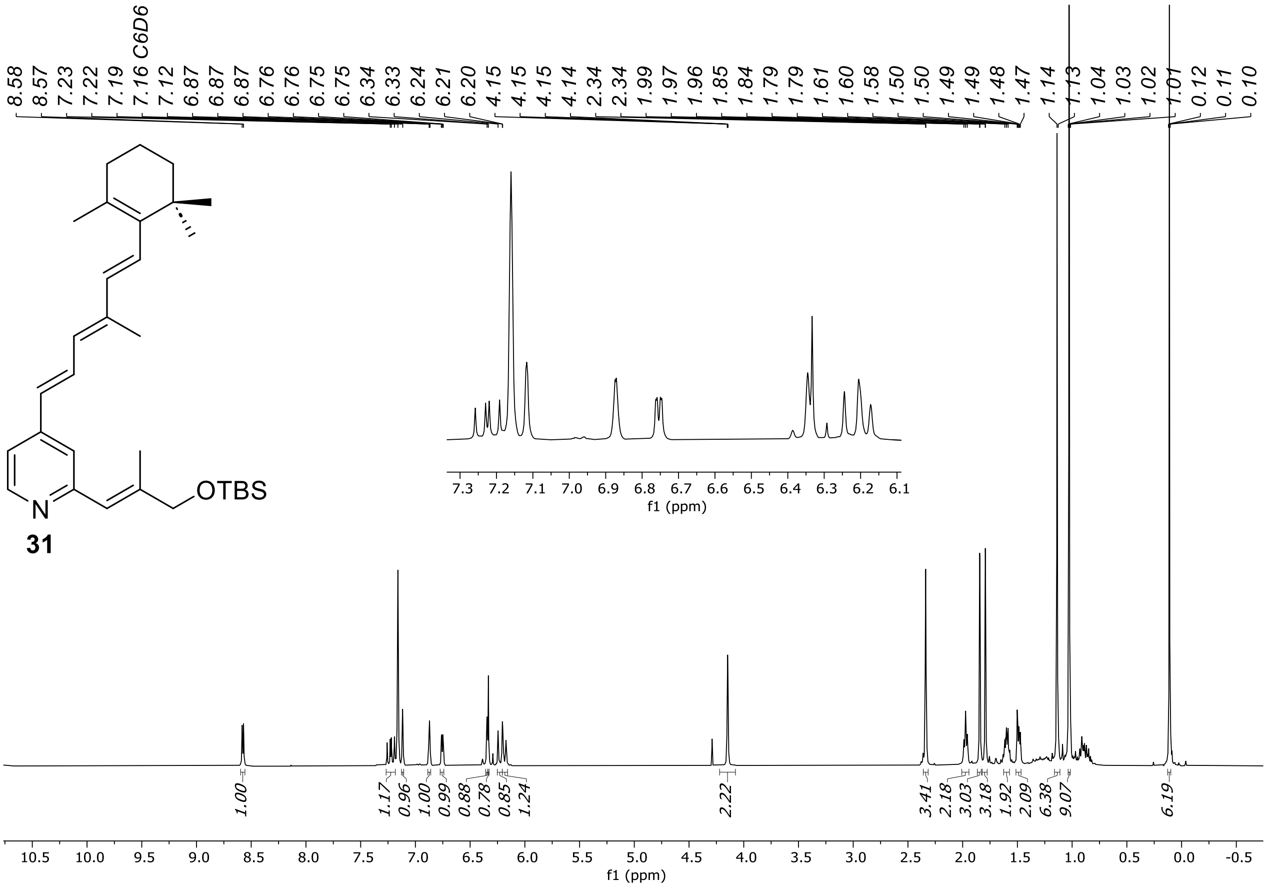

^{13}C{^{1}H}-NMR (100.63 MHz, C_6D_6)

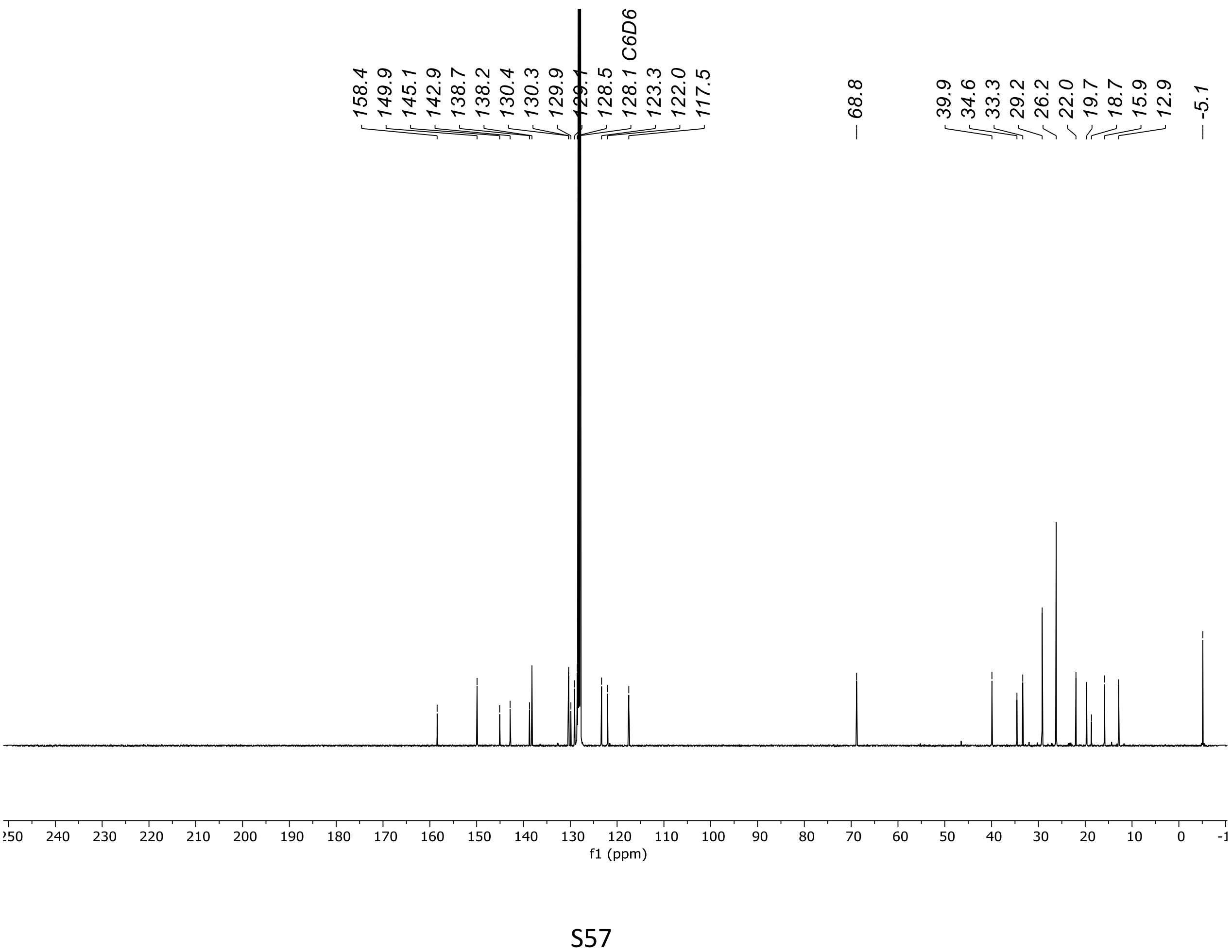

COSY (C_6D_6)

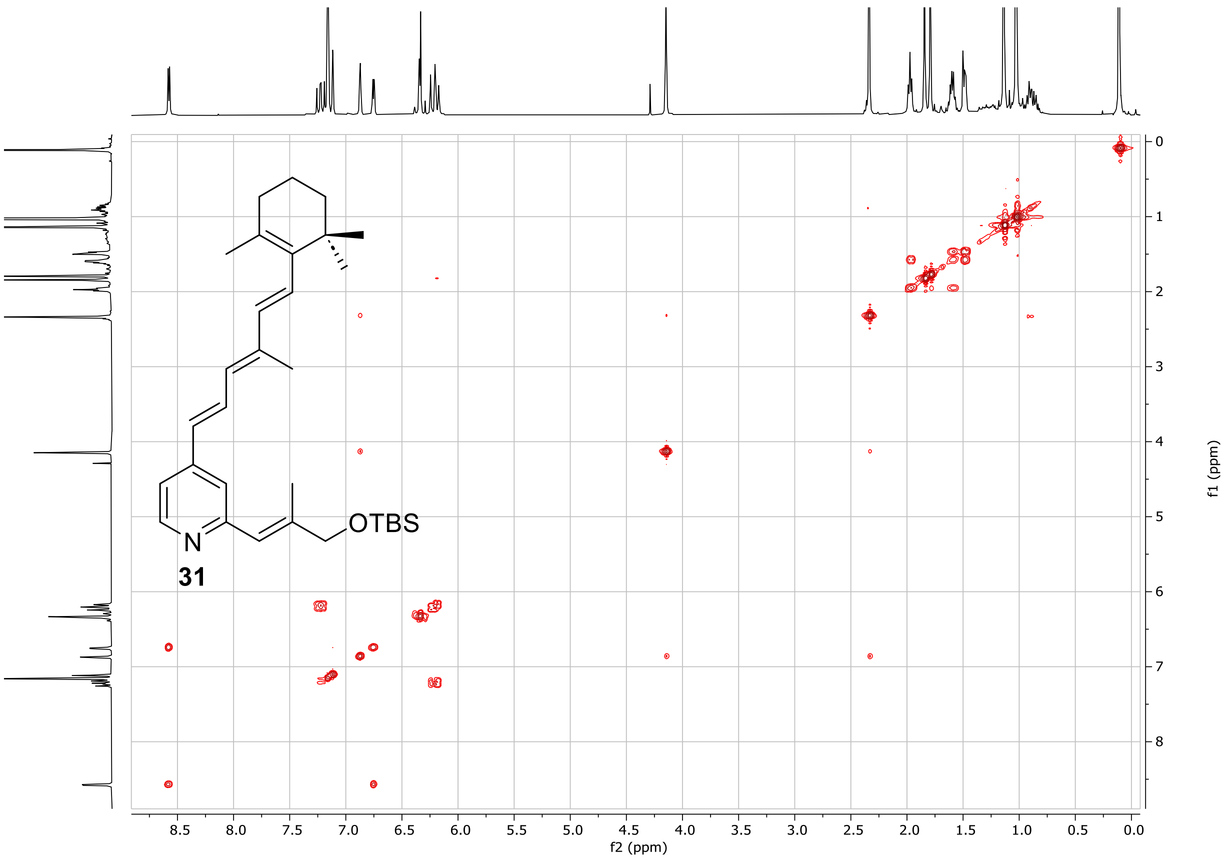

HSQC (C_6D_6)

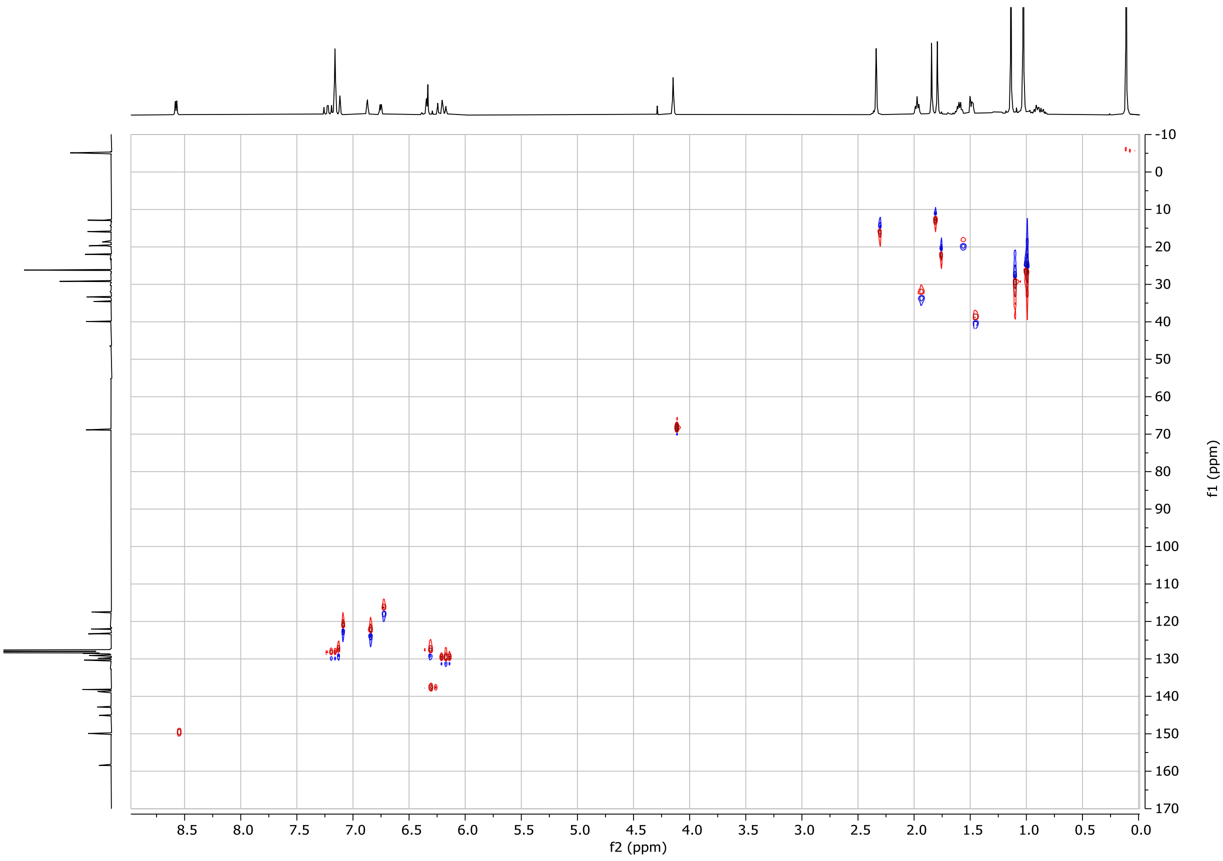

HMBC (C_6D_6)

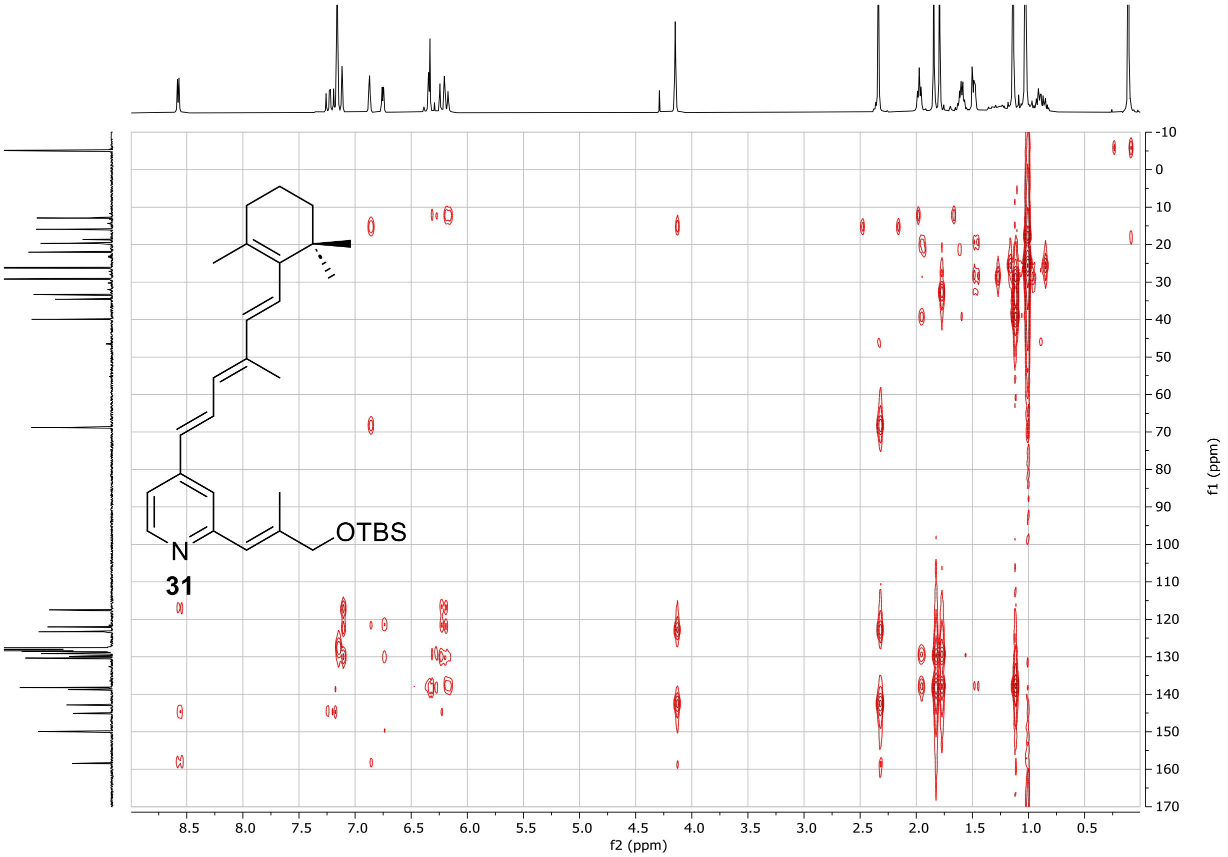

^{1}H-NMR (400.16 MHz, C_6D_6)

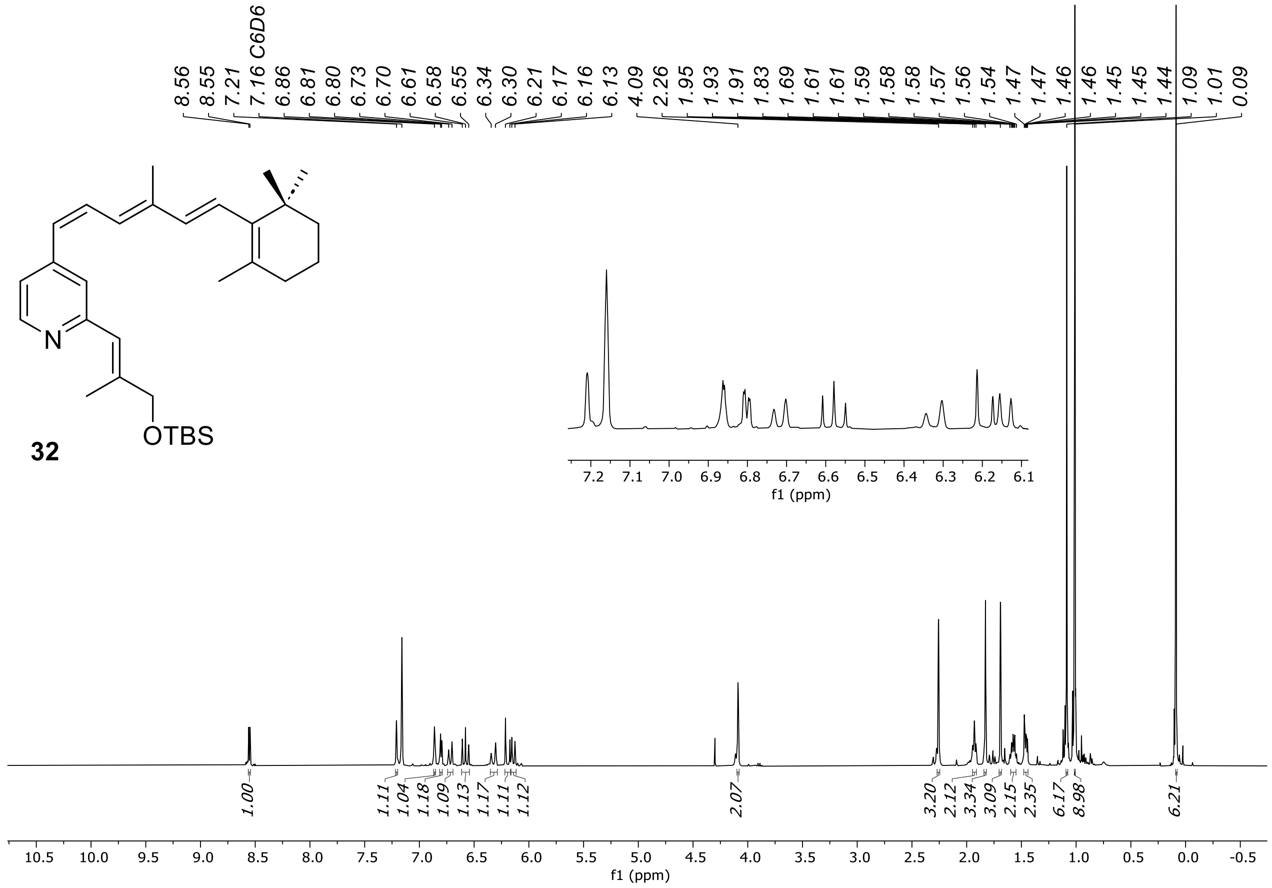

32

^{13}C{^{1}H}-NMR (100.63 MHz, C_6D_6)

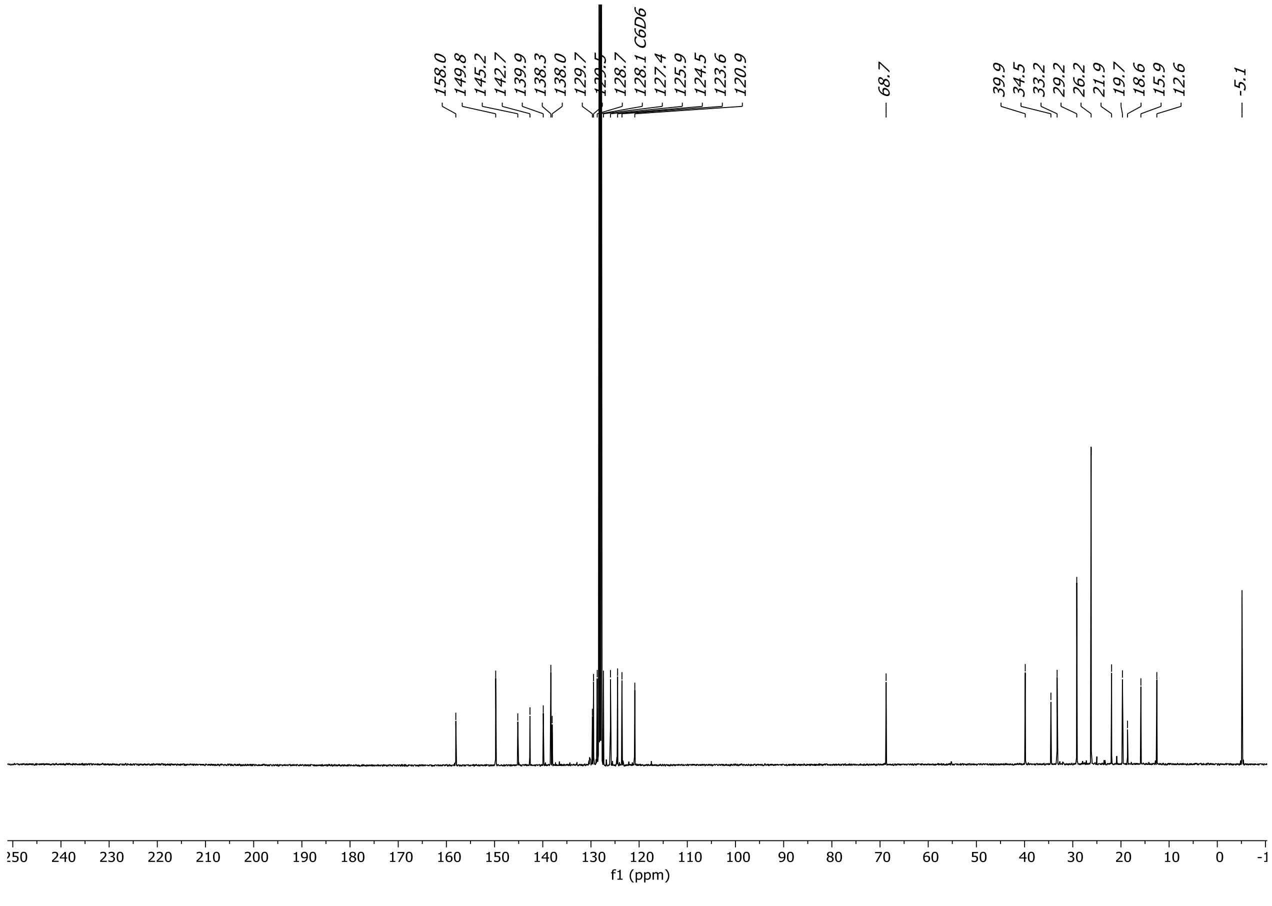

COSY (C_6D_6)

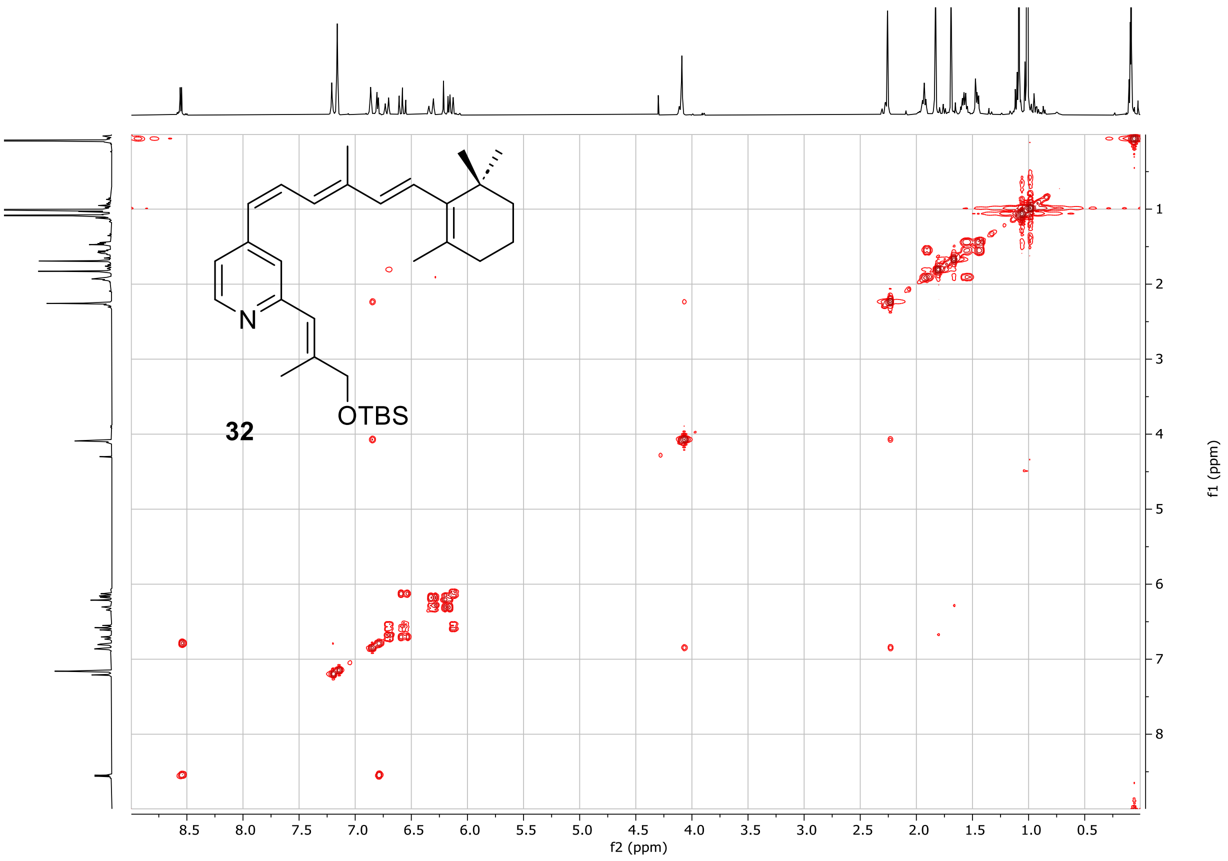

HSQC (C_6D_6)

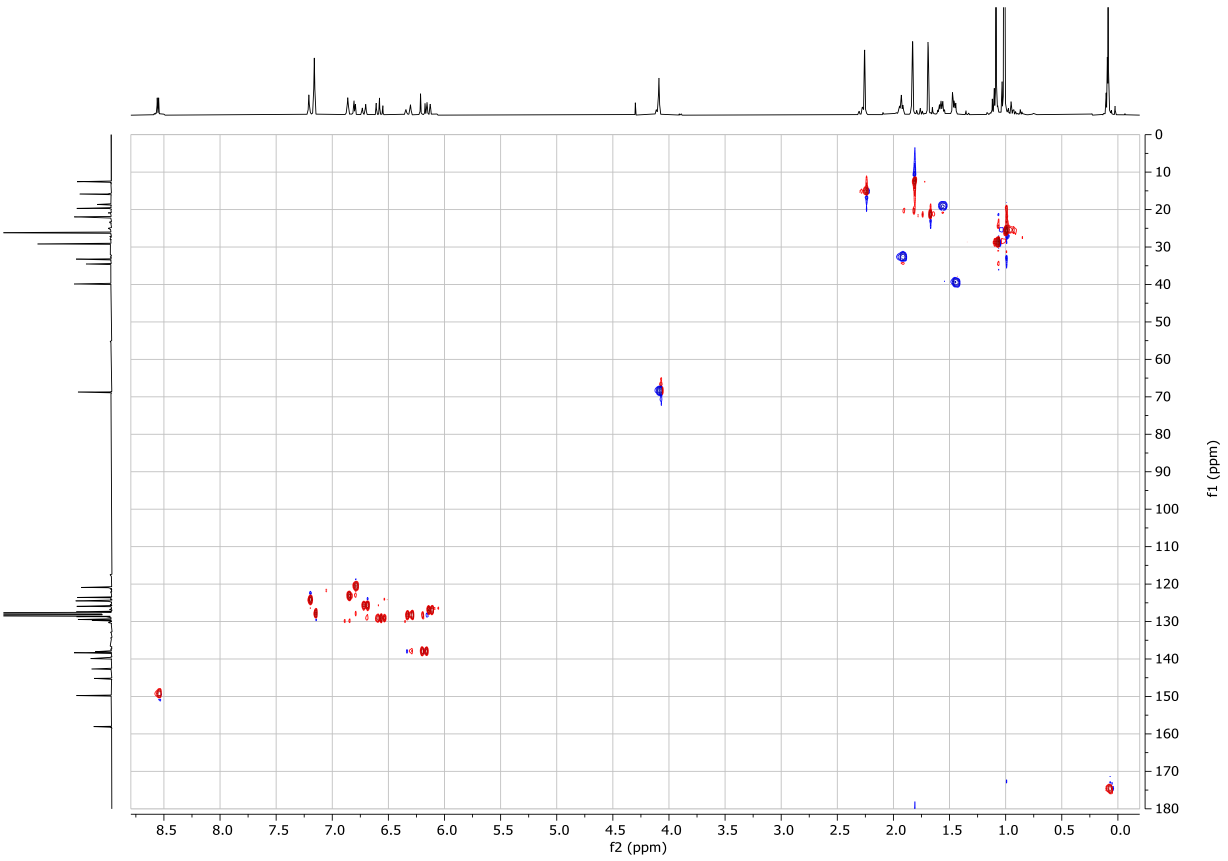

HMBC (C_6D_6)

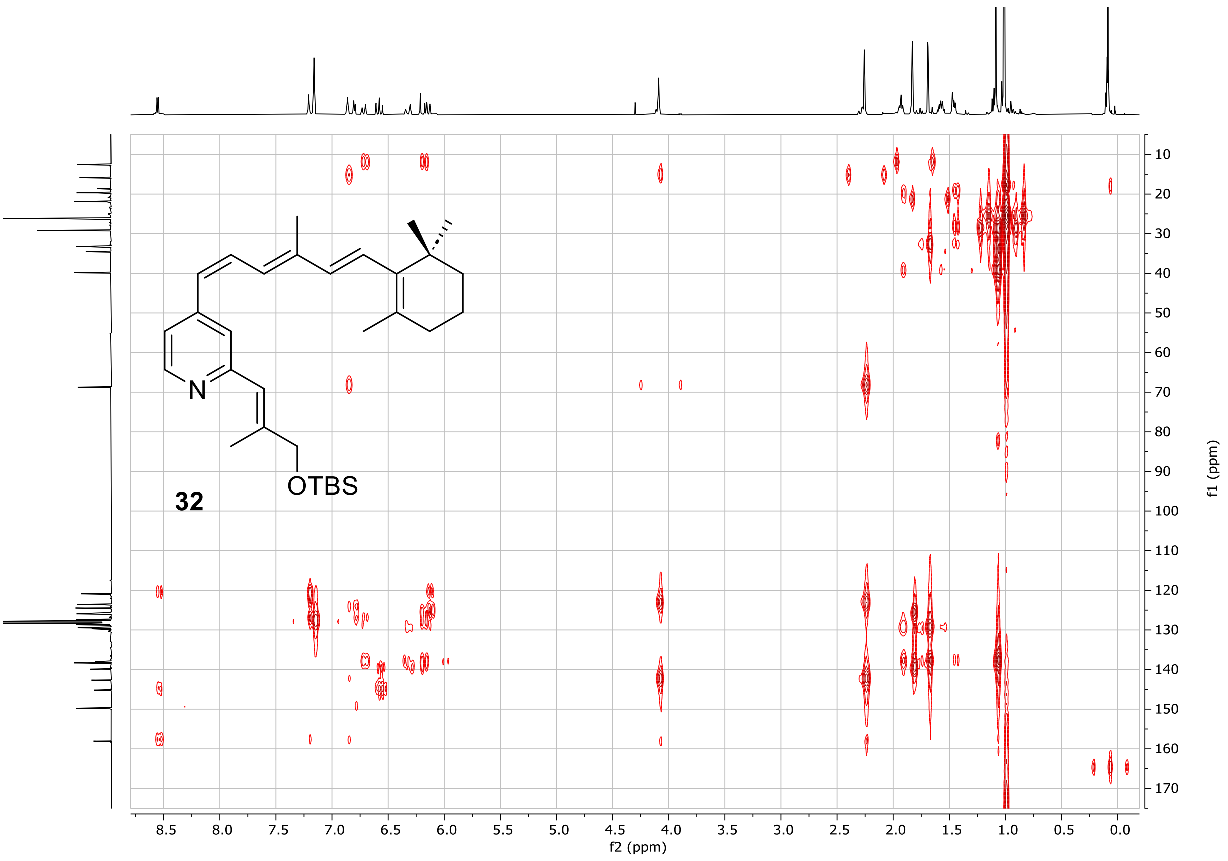

^{1}H-NMR (400.16 MHz, CD_2Cl_2)

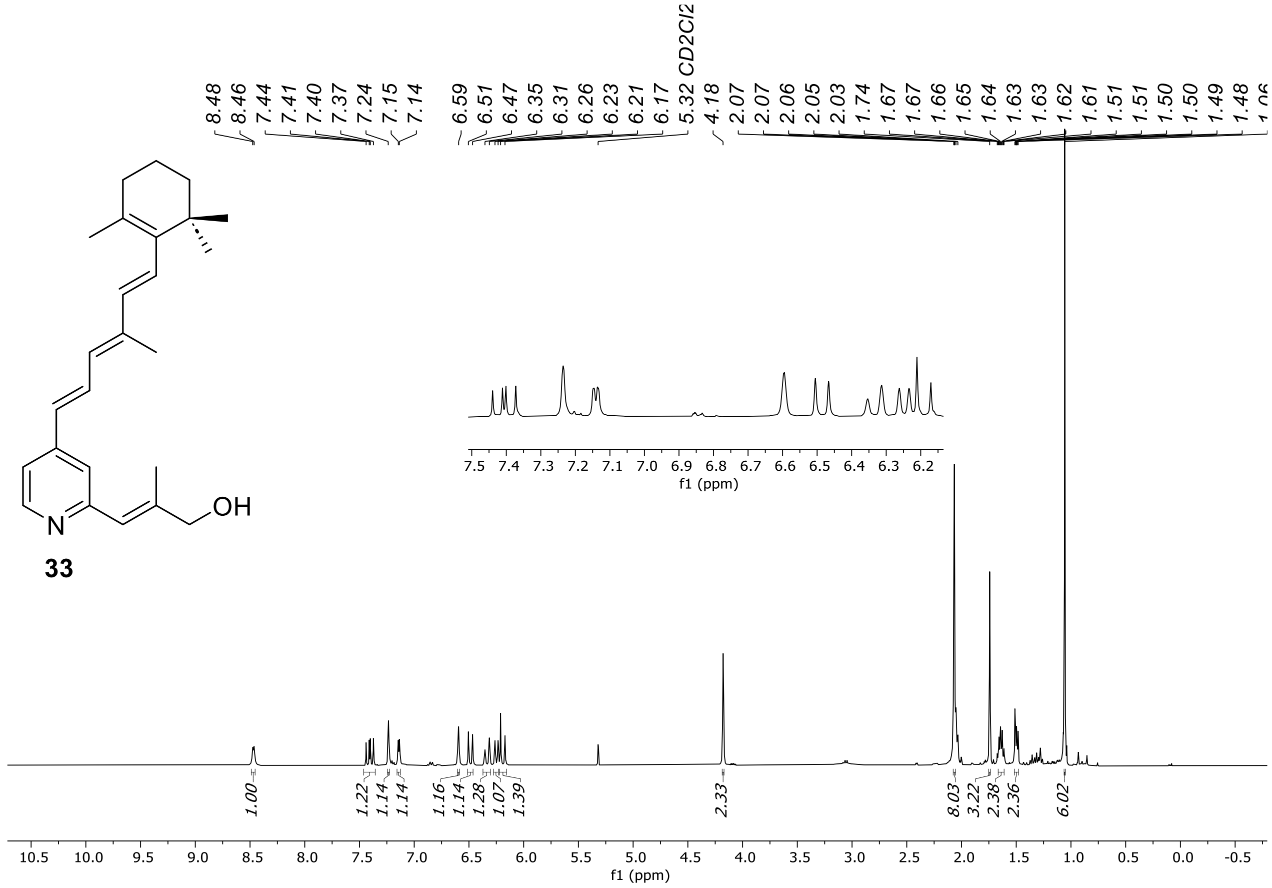

^{13}C{^{1}H}-NMR (100.63 MHz, CD_2Cl_2)

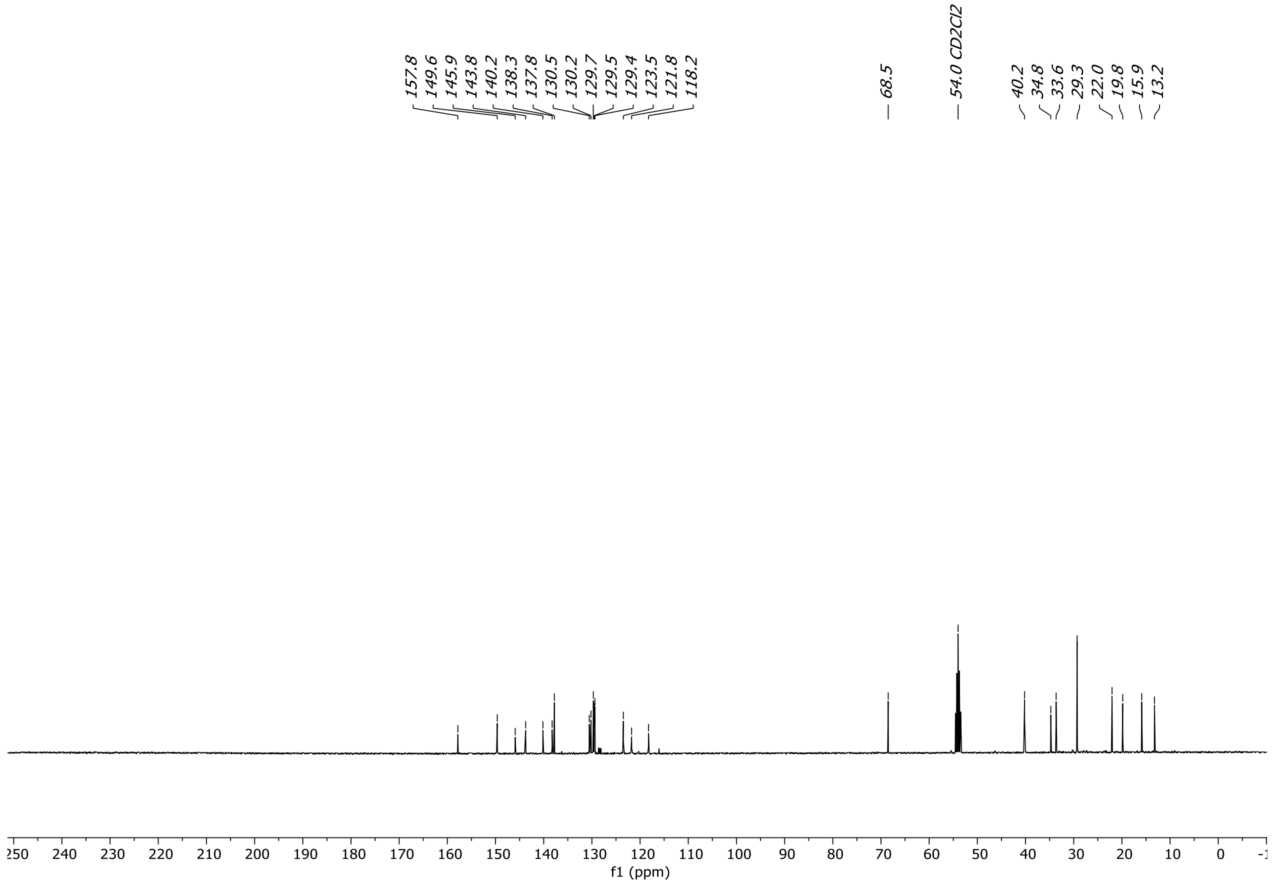

COSY (CD_2Cl_2)

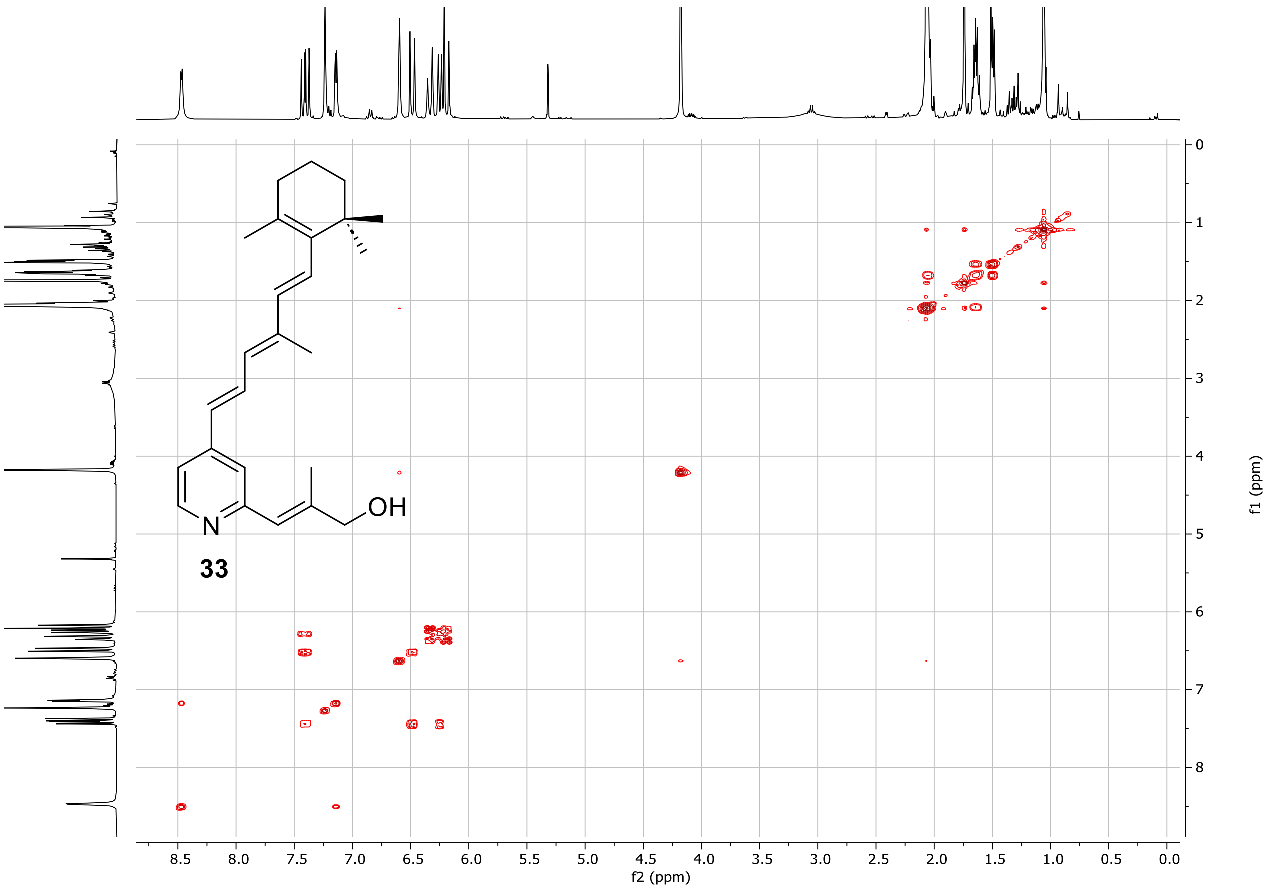

HSQC (CD_2Cl_2)

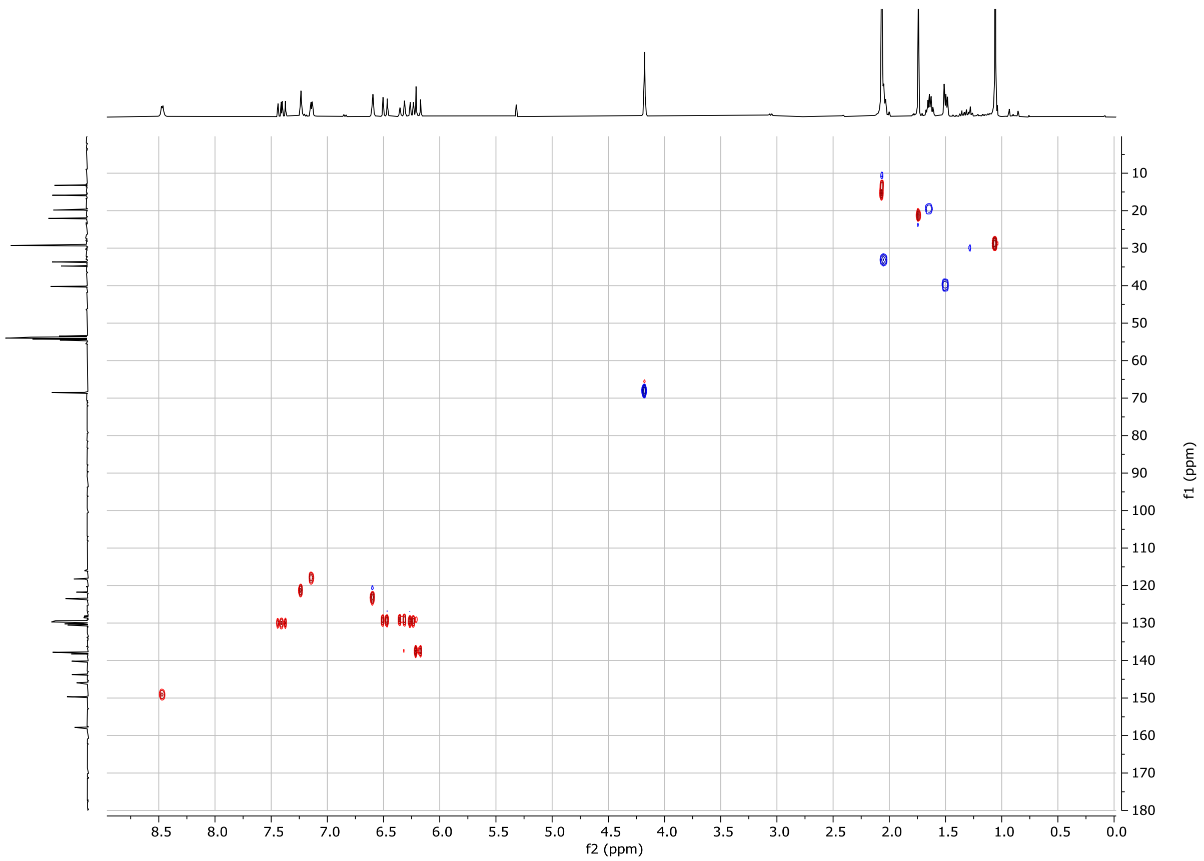

HMBC (CD_2Cl_2)

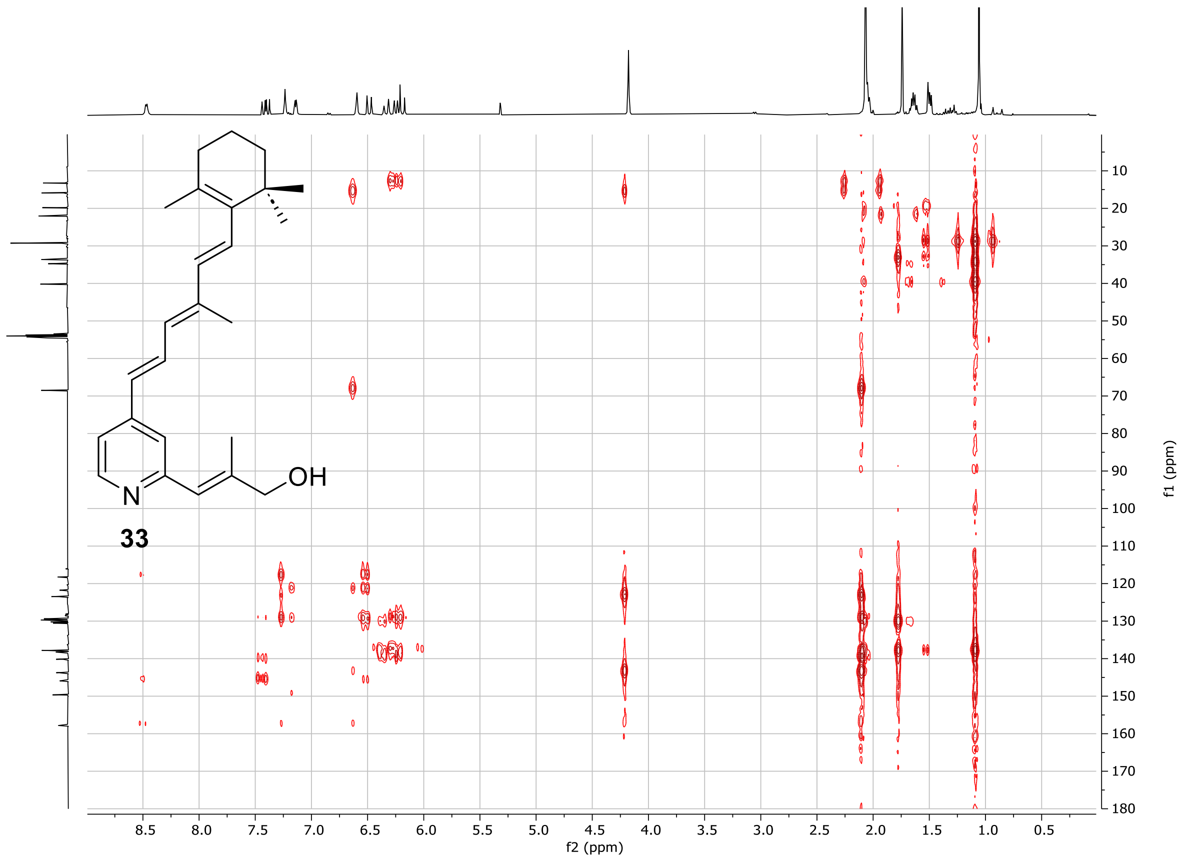
OH
N
33
8.5
8.0
7.5
7.0
6.5
6.0
5.5
5.0
4.5
4.0
3.5
3.0
2.5
2.0
1.5
1.0
0.5
f2 (ppm)
10
20
30
40
50
60
70
80
90
100
110
120
130
140
150
160
170
180
f1 (ppm)

^{1}H-NMR (400.16 MHz, C_6D_6)

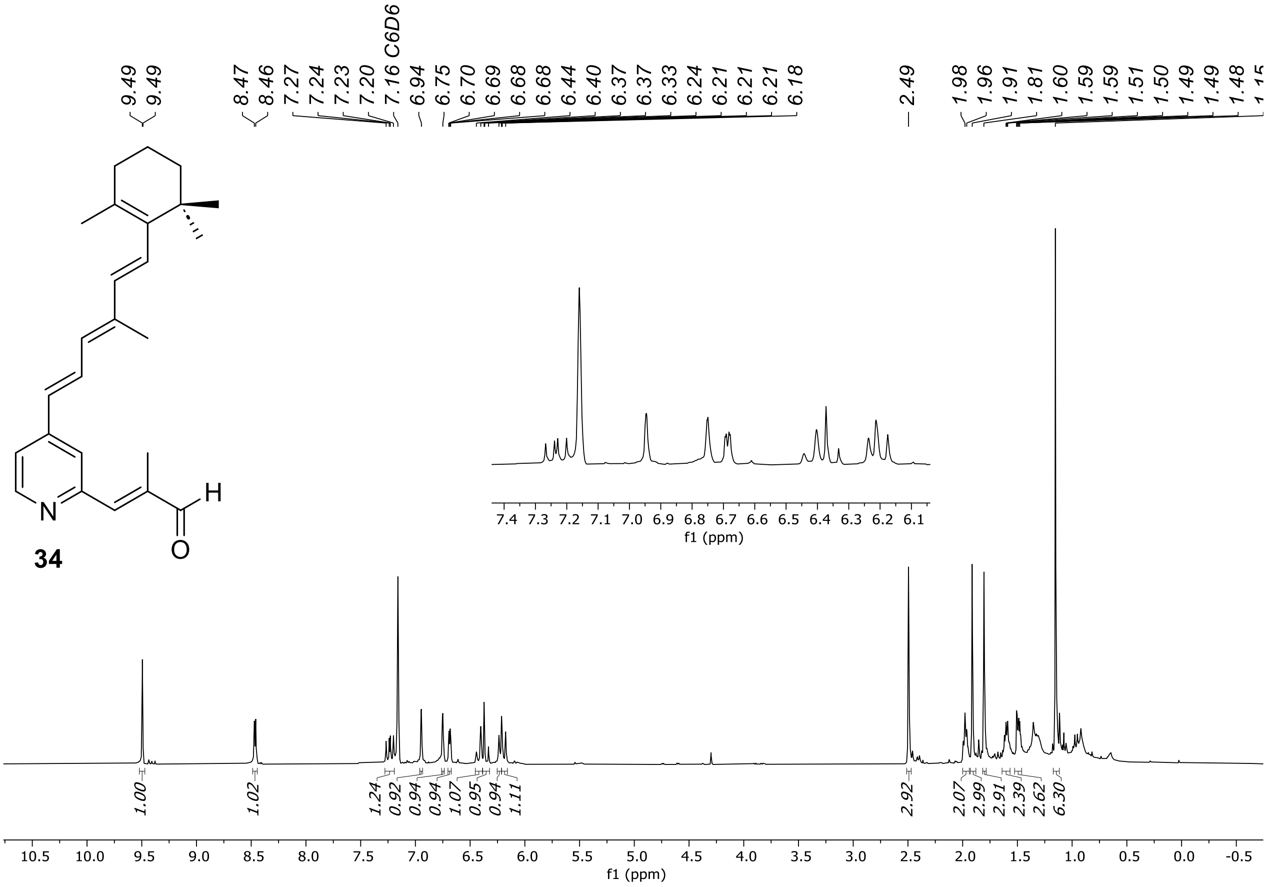

^{13}C{^{1}H}-NMR (100.63 MHz, C_6D_6)

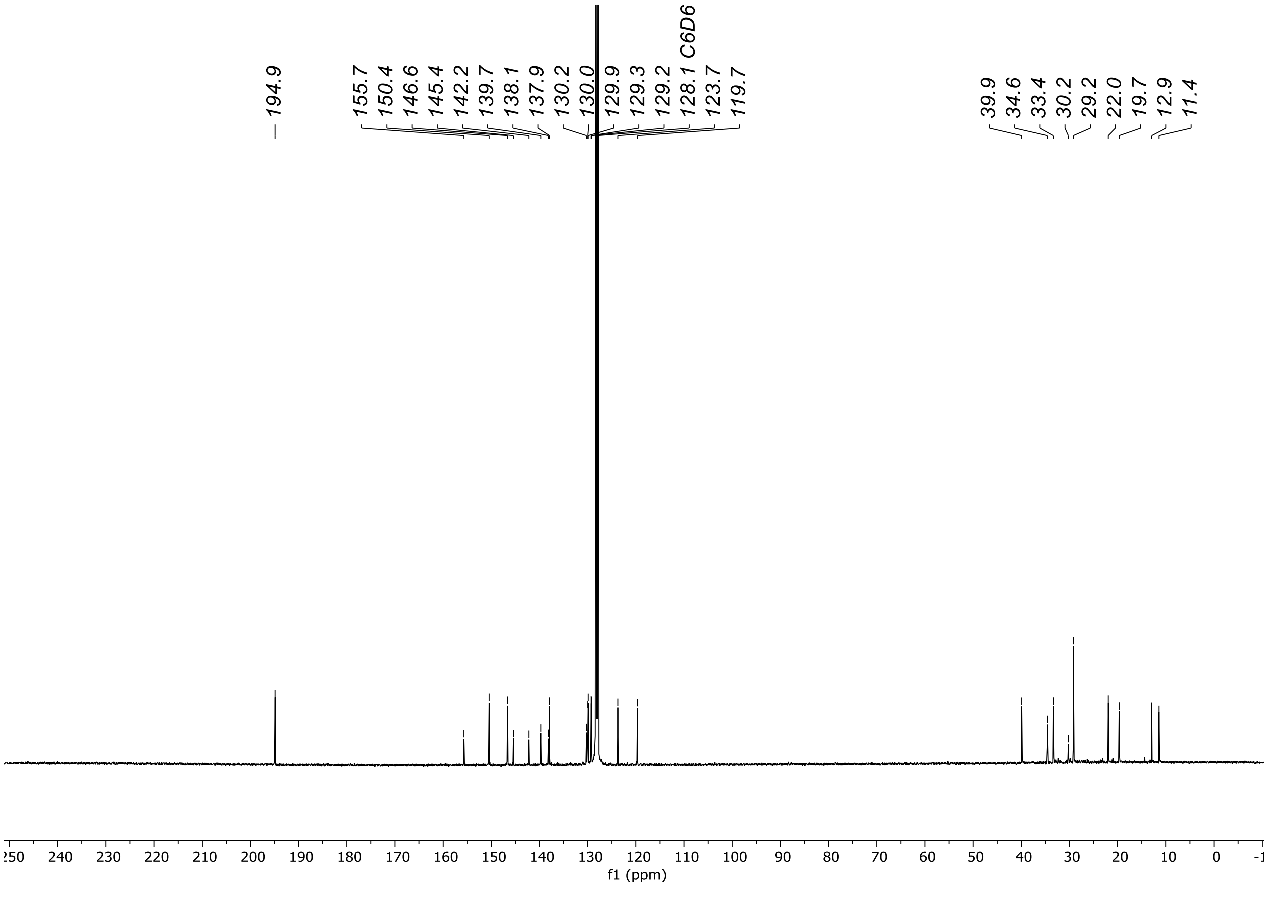

COSY (C_6D_6)

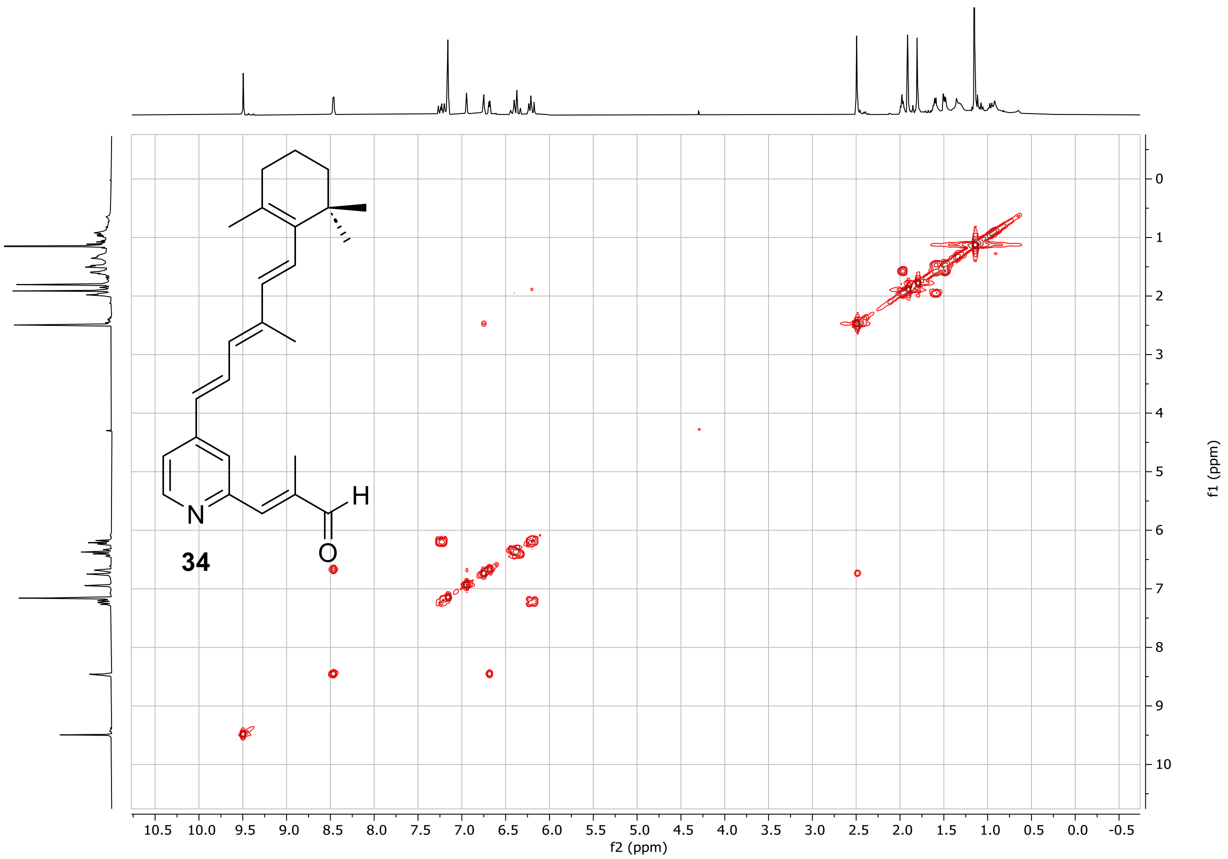

HSQC (C_6D_6)

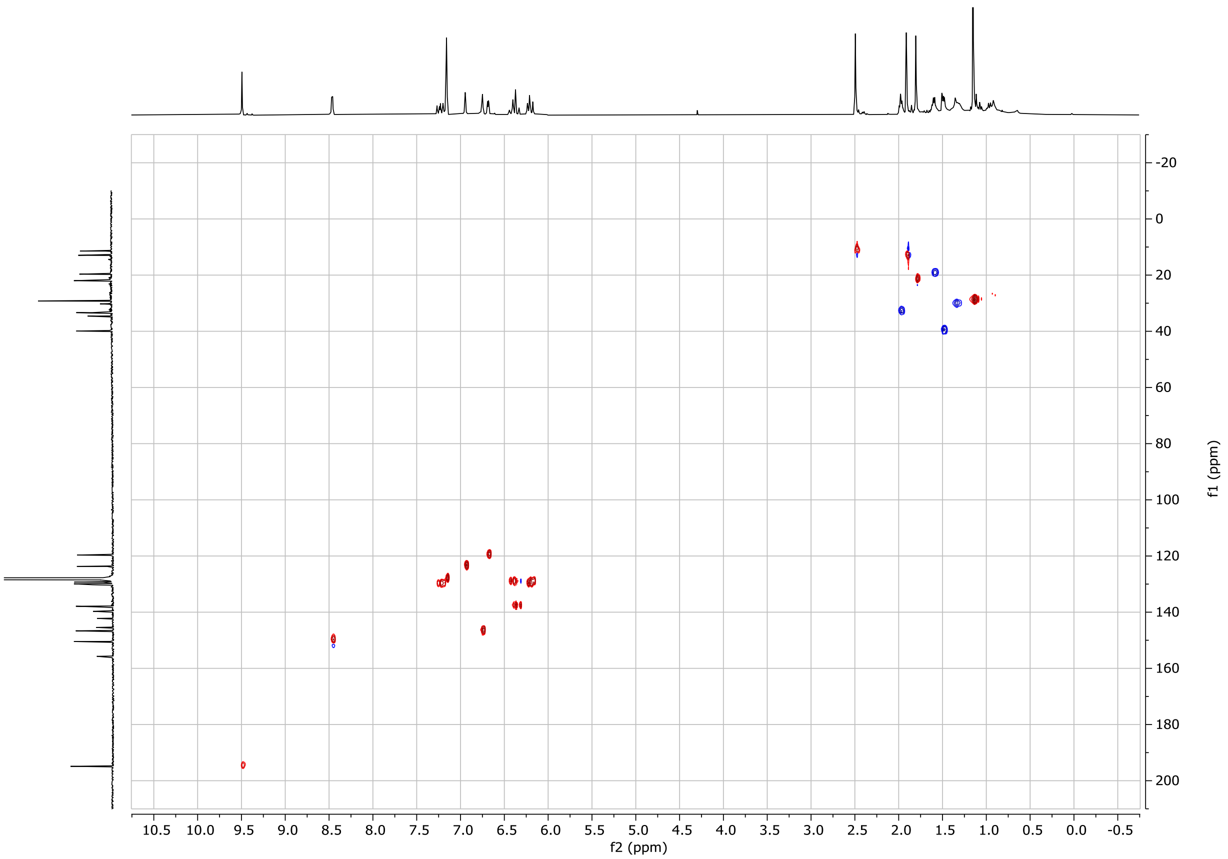

HMBC (C_6D_6)

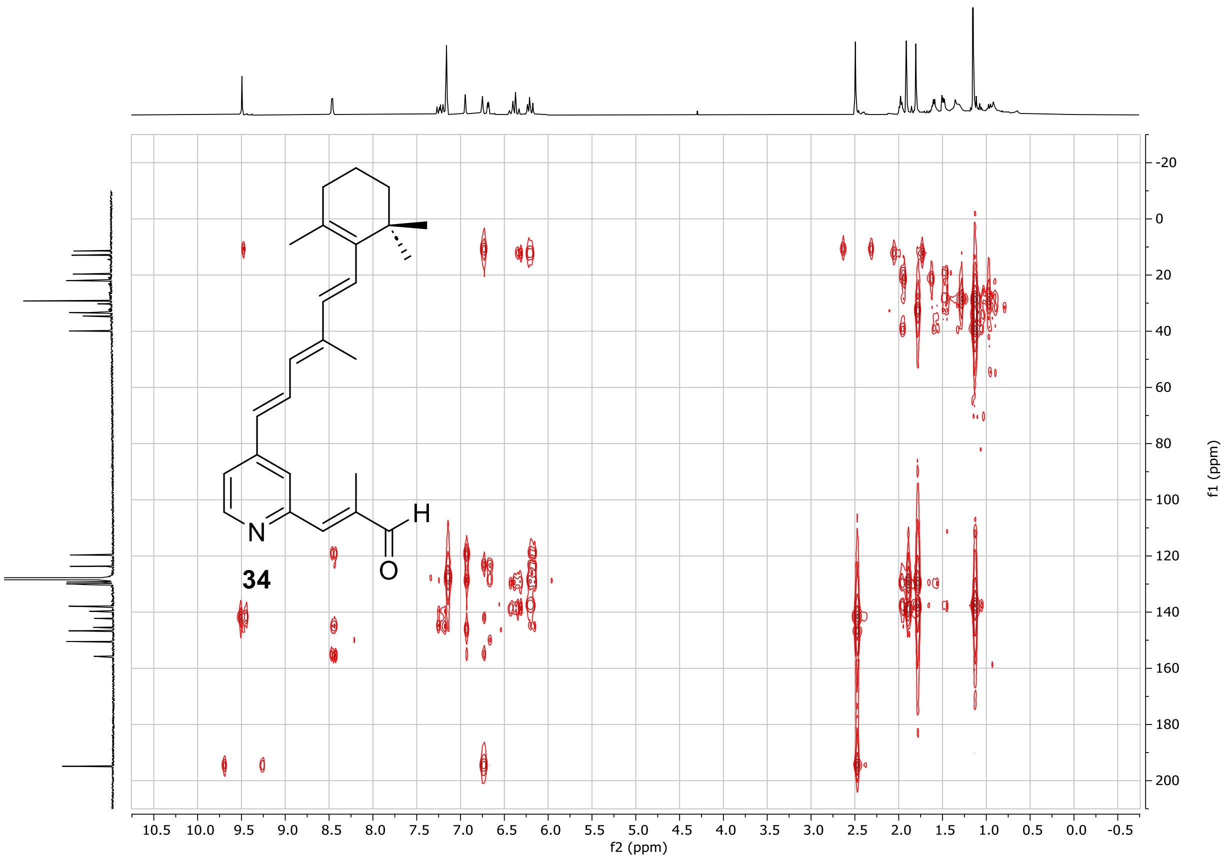

^{1}H-NMR (400.16 MHz, C_6D_6)

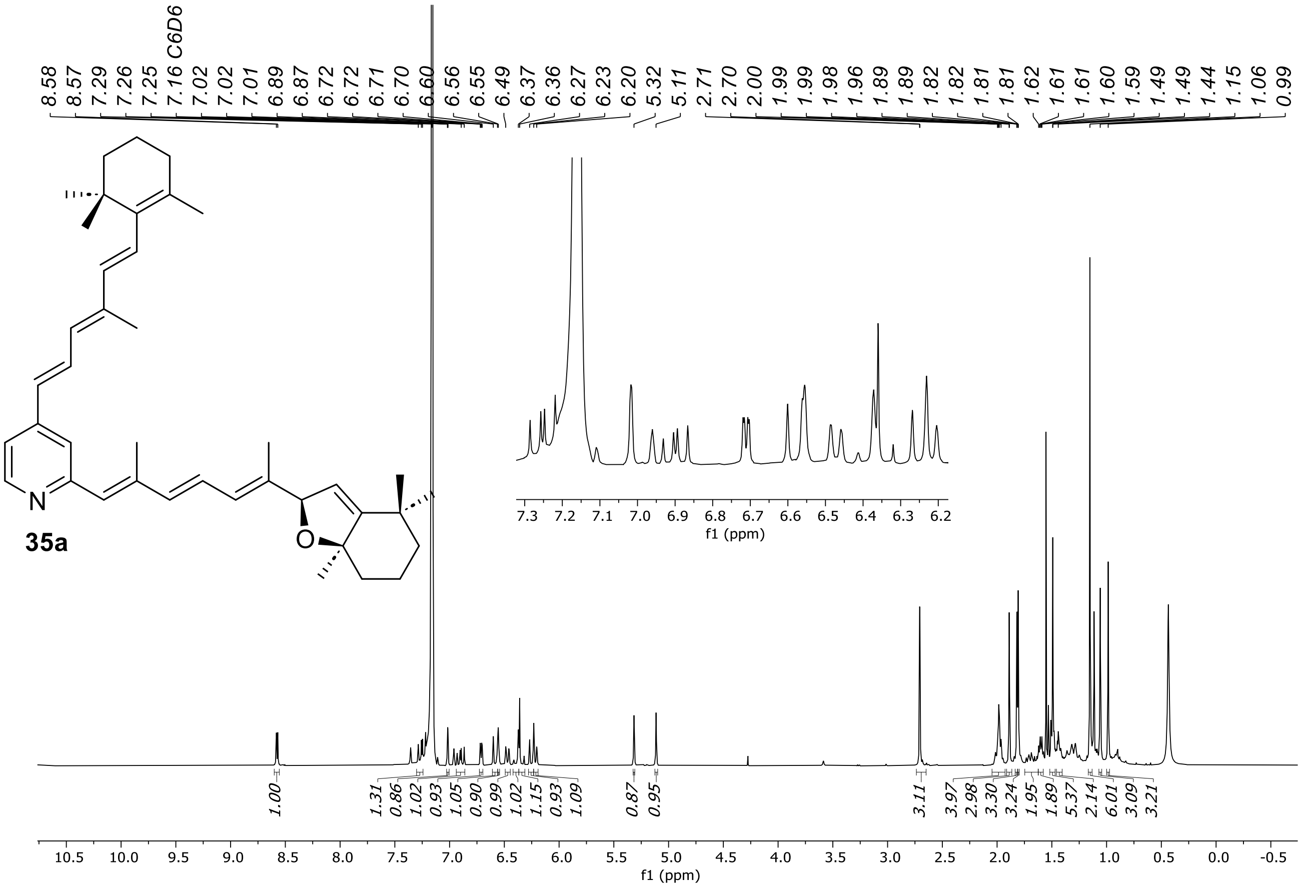

^{13}C{^{1}H}-NMR (100.63 MHz, C_6D_6)

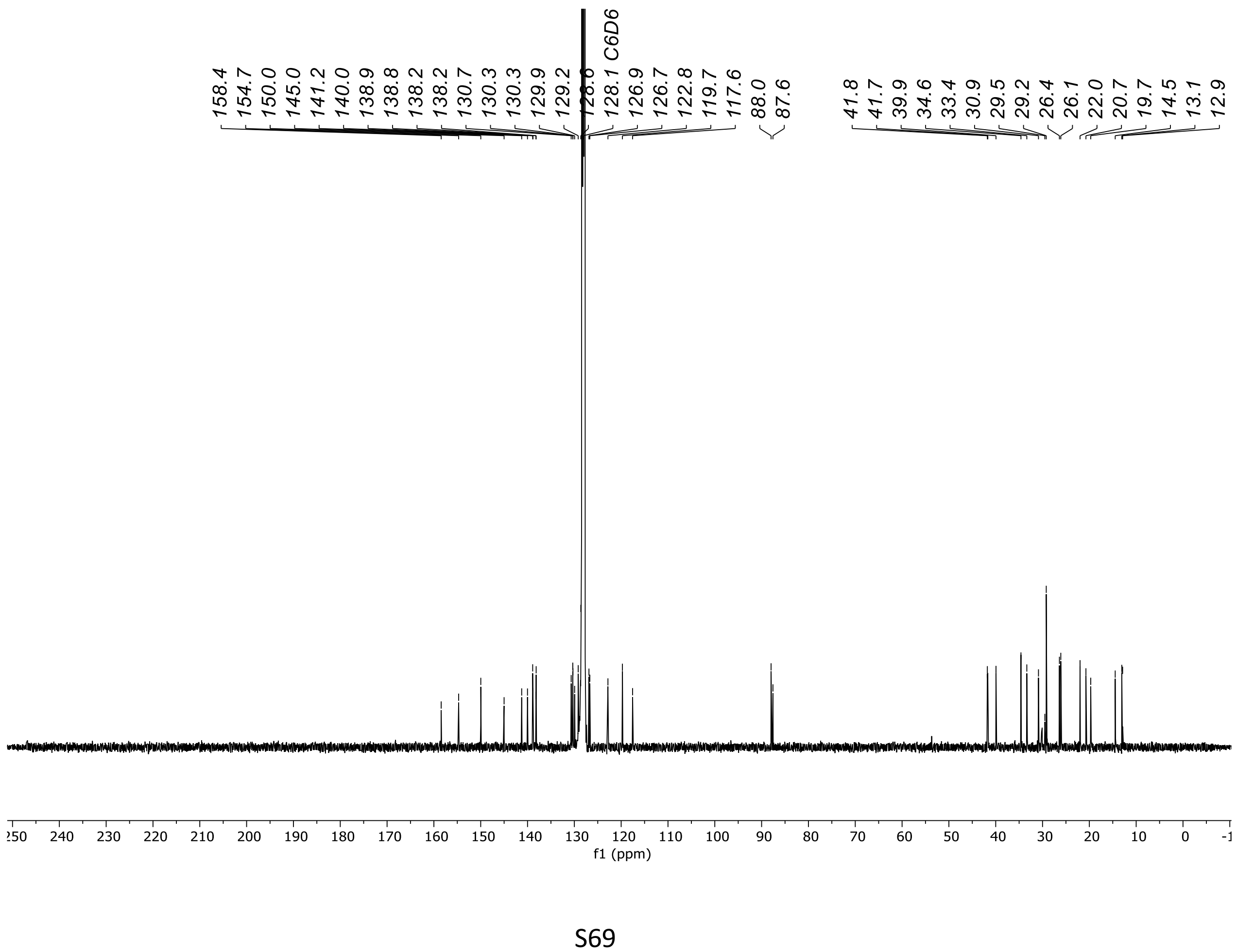

COSY (C_6D_6)

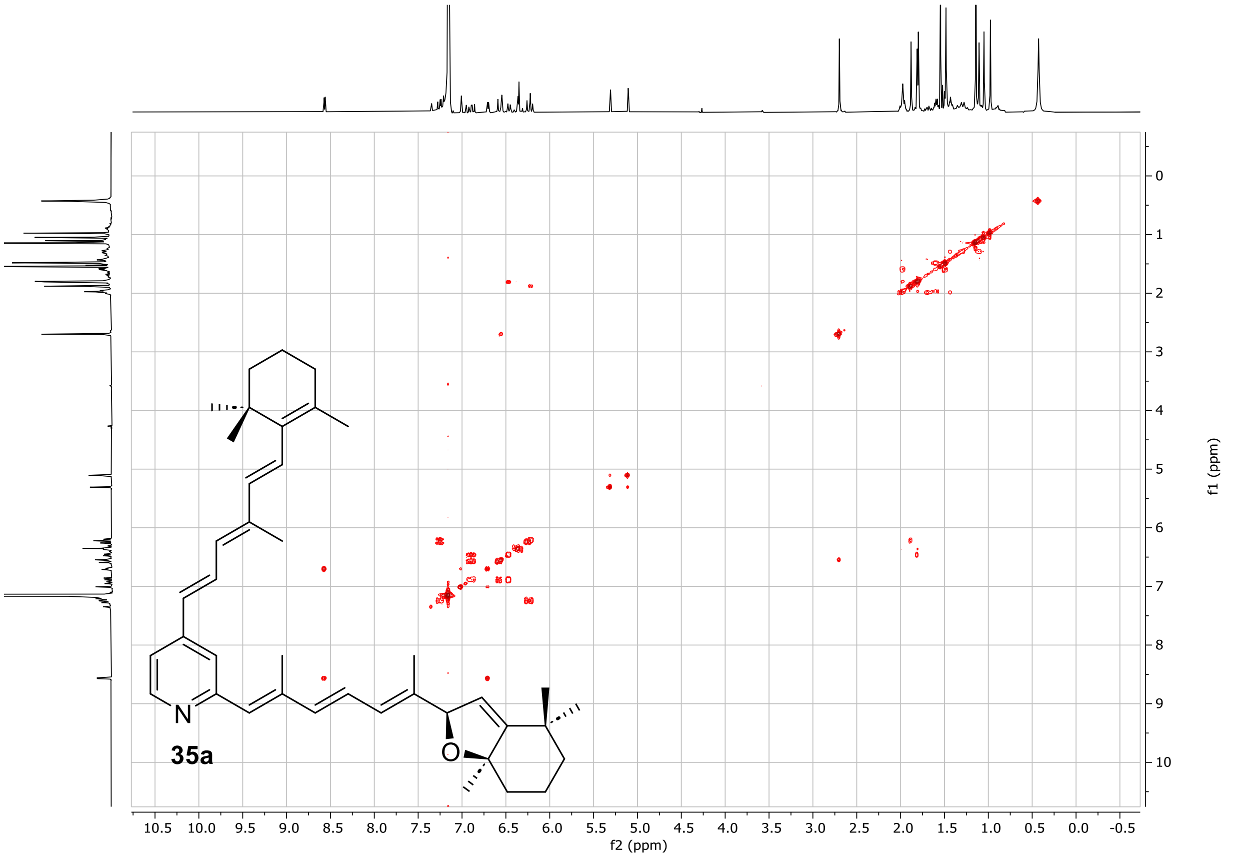

HSQC (C_6D_6)

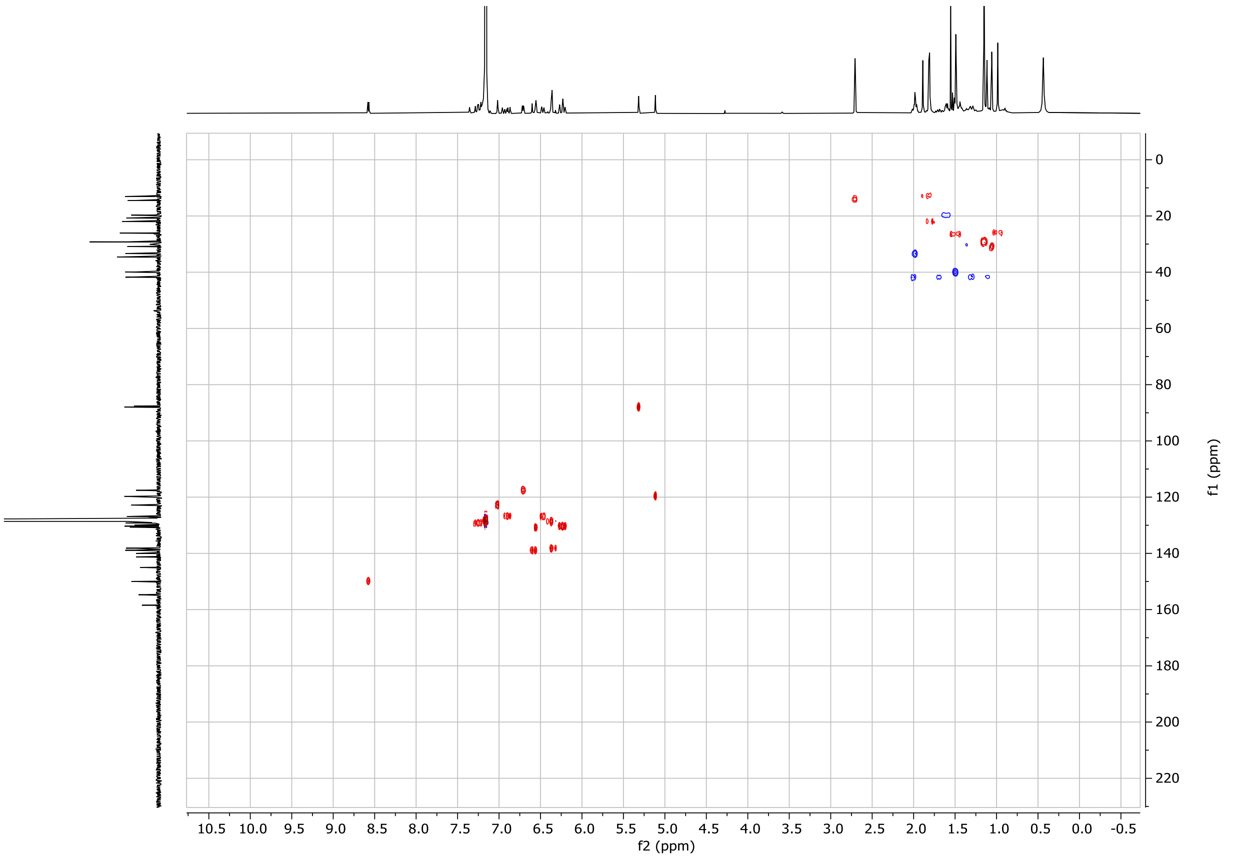

HMBC (C_6D_6)

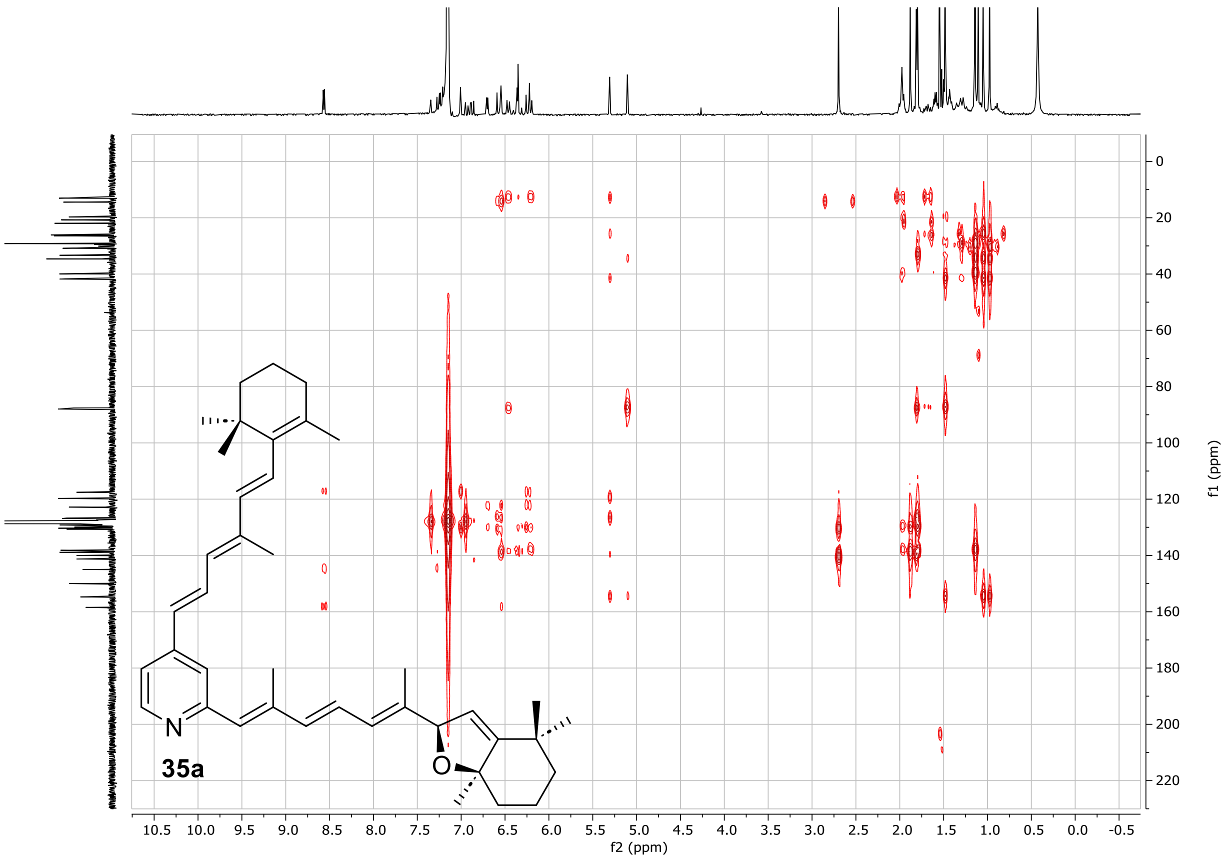

NOE-1D (400.16 MHz, freq. 5.30 ppm, C_6D_6)

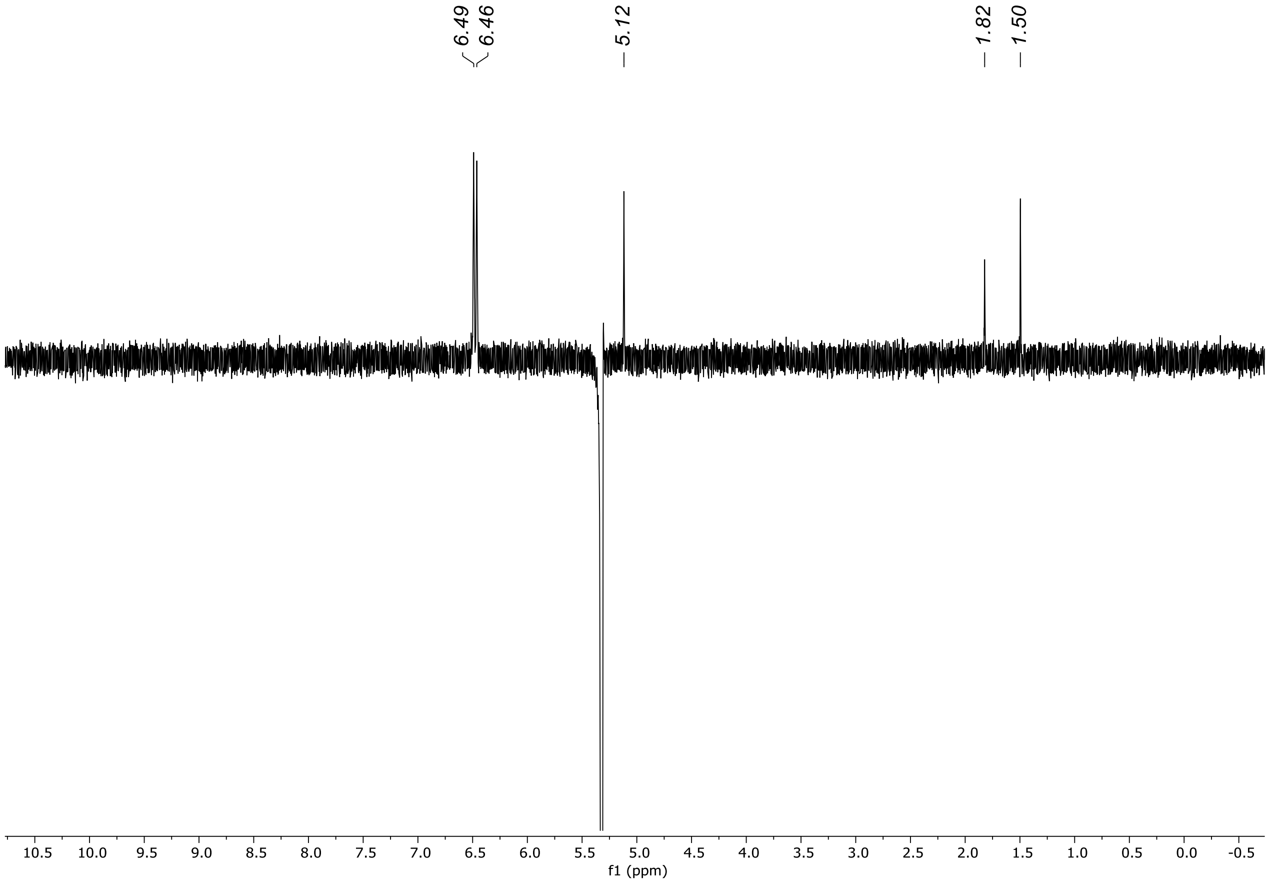

^{1}H-NMR (400.16 MHz, C_6D_6)

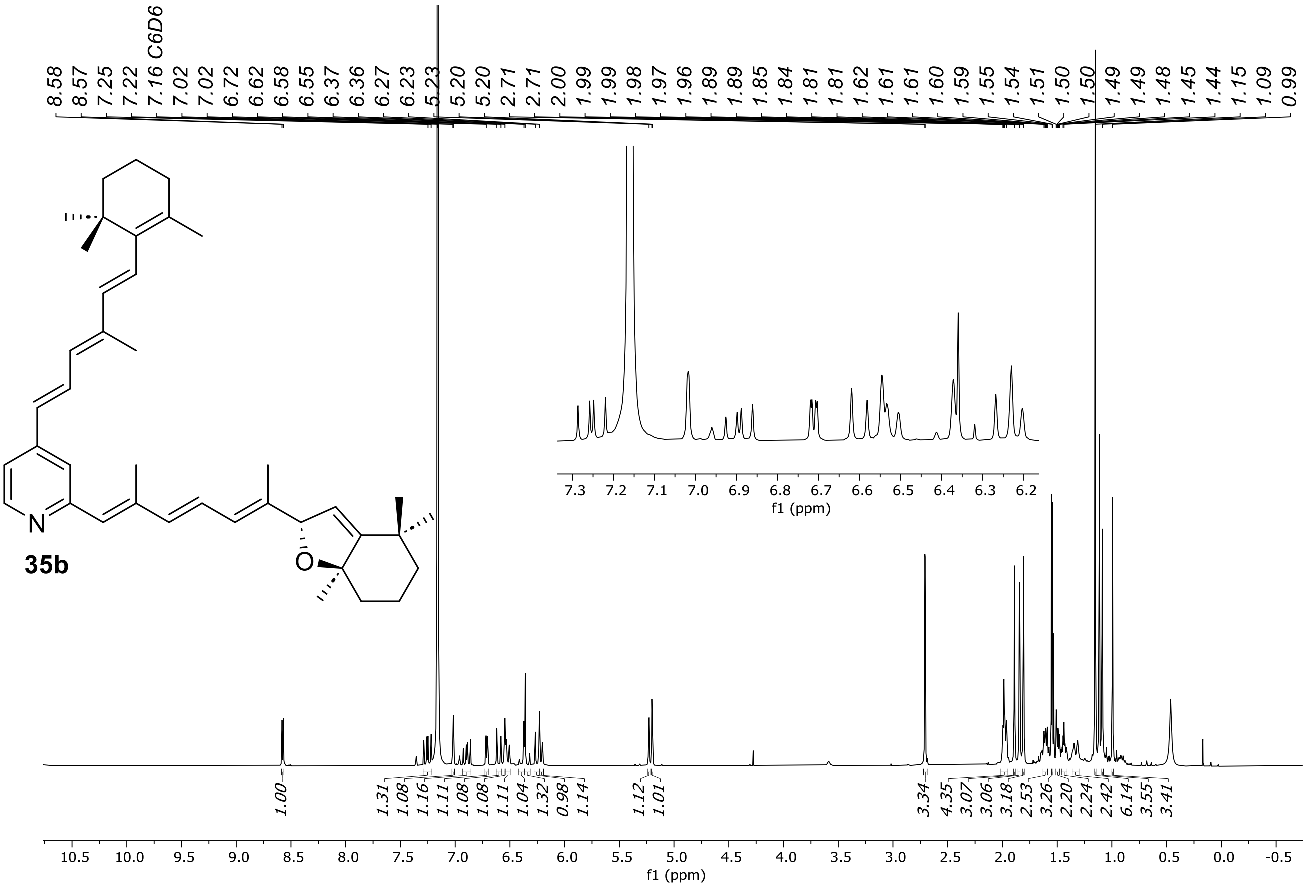

35b

^{13}C{^{1}H}-NMR (100.63 MHz, C_6D_6)

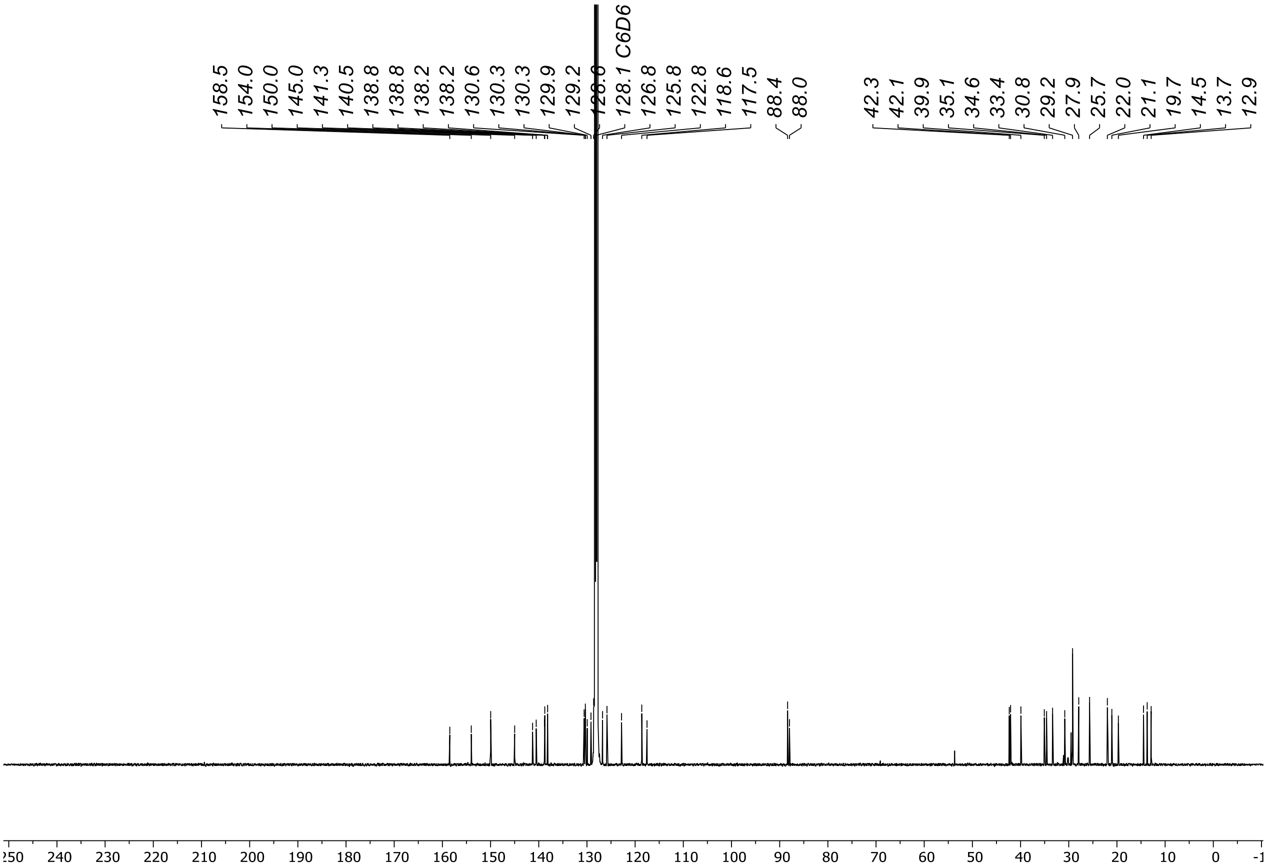

COSY (C_6D_6)

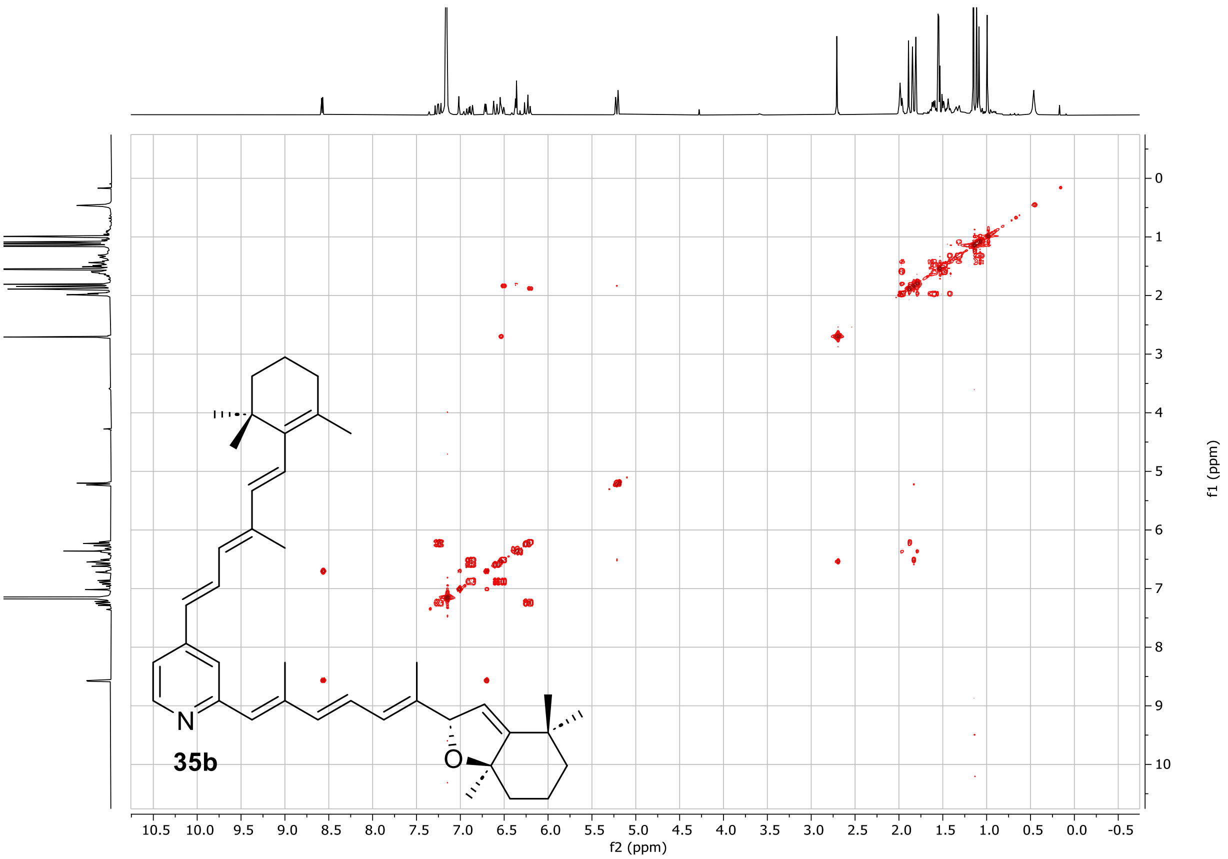

HSQC (C_6D_6)

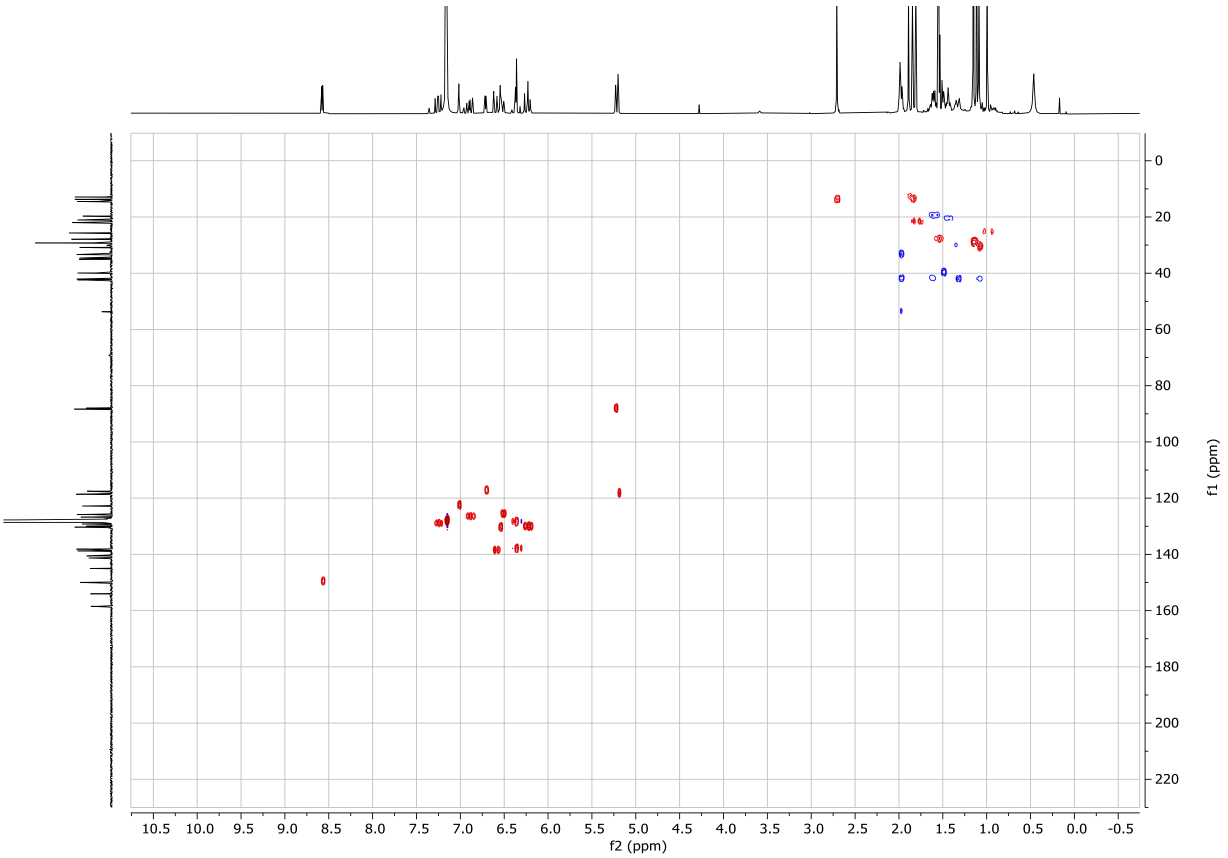

HMBC (C_6D_6)

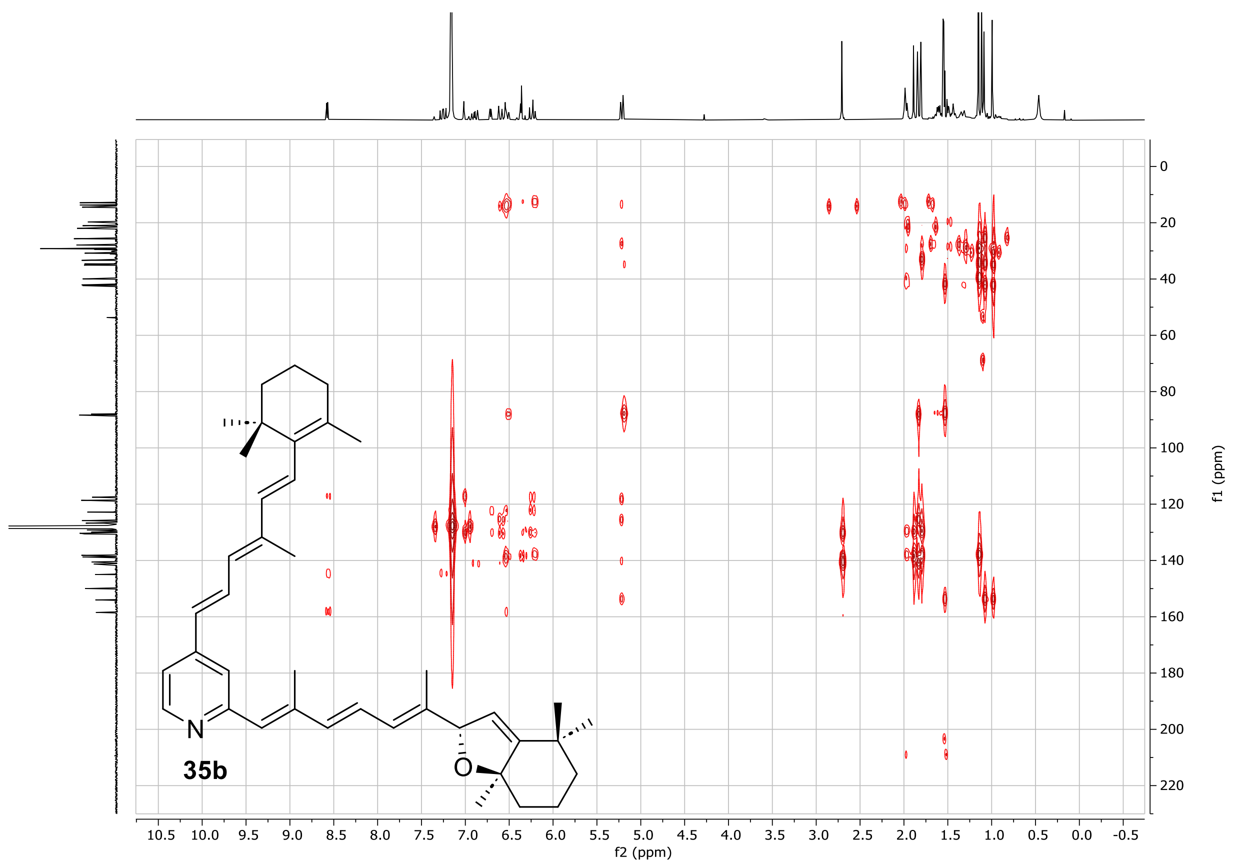

NOE-1D (400.16 MHz, freq. 5.22 ppm, C_6D_6)

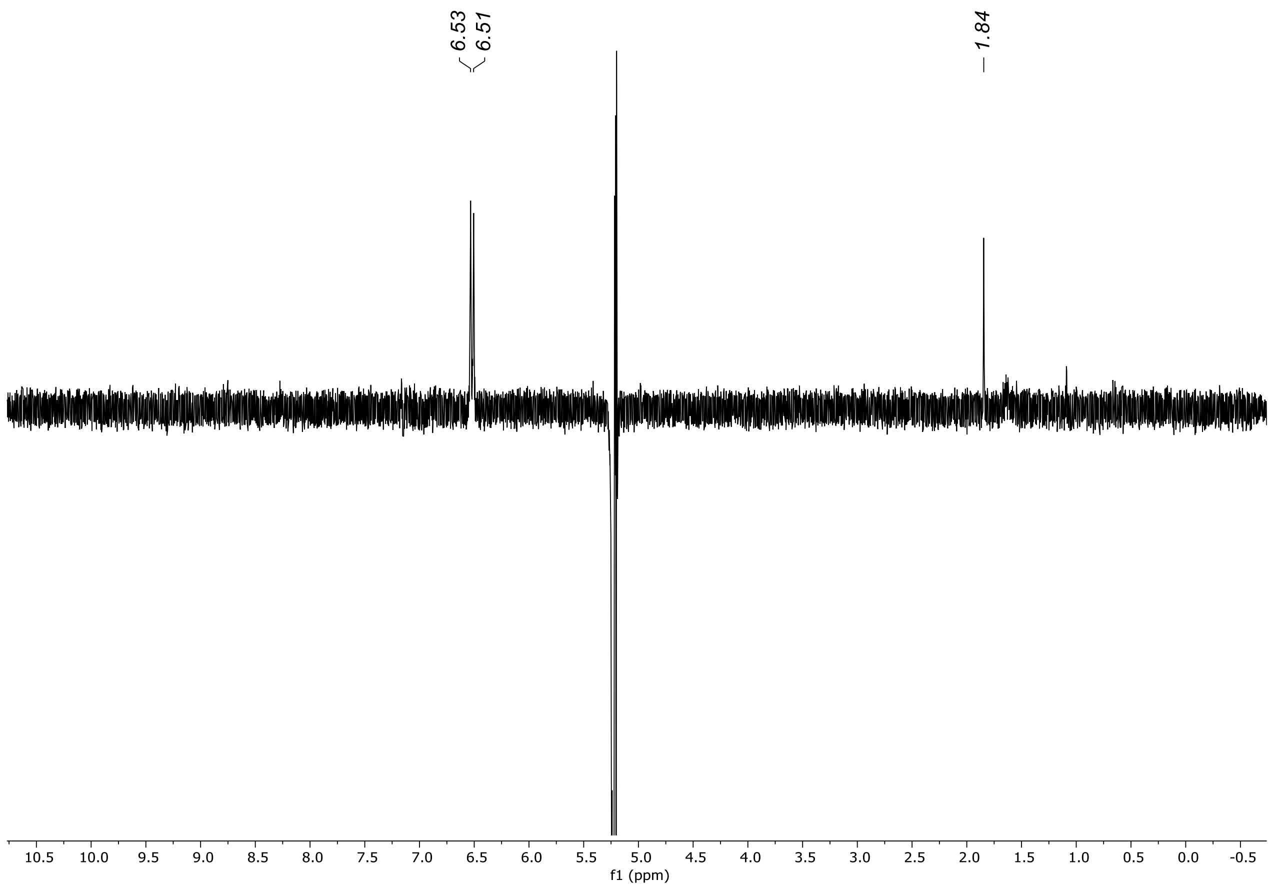

^{1}H-NMR (400.16 MHz, CD_3OD)

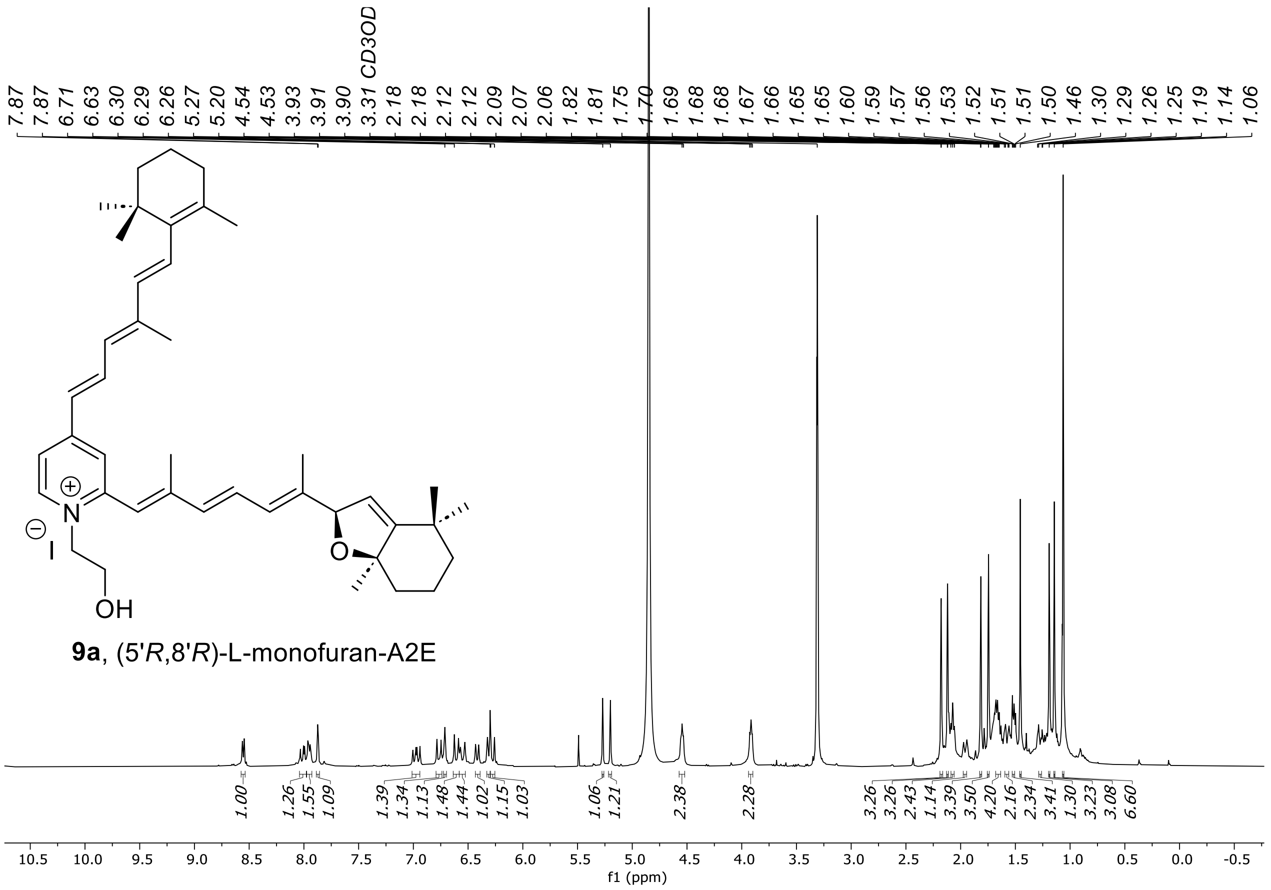

^{13}C{^{1}H}-NMR (100.63 MHz, CD_3OD)

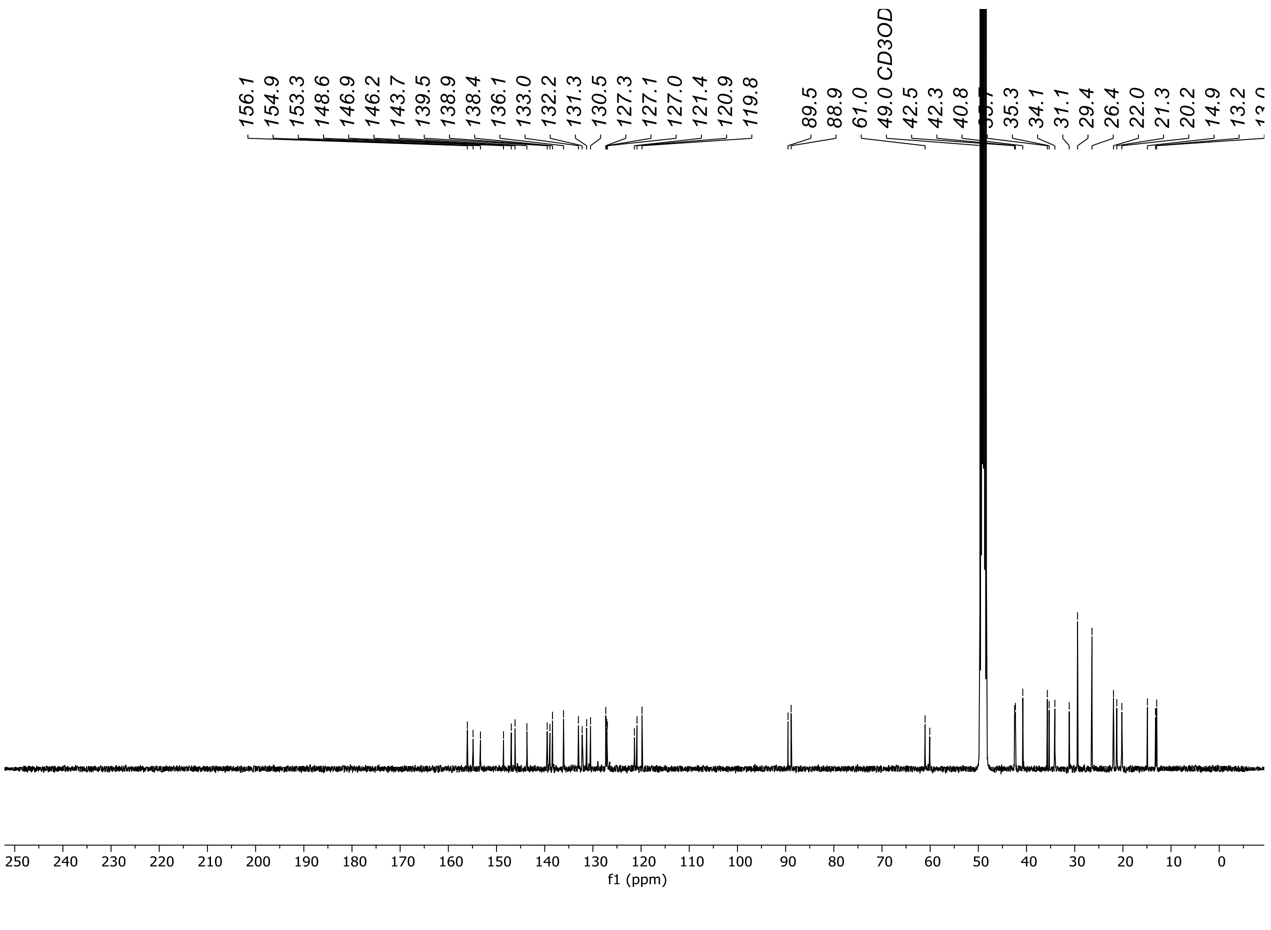

COSY (CD$_3$OD)

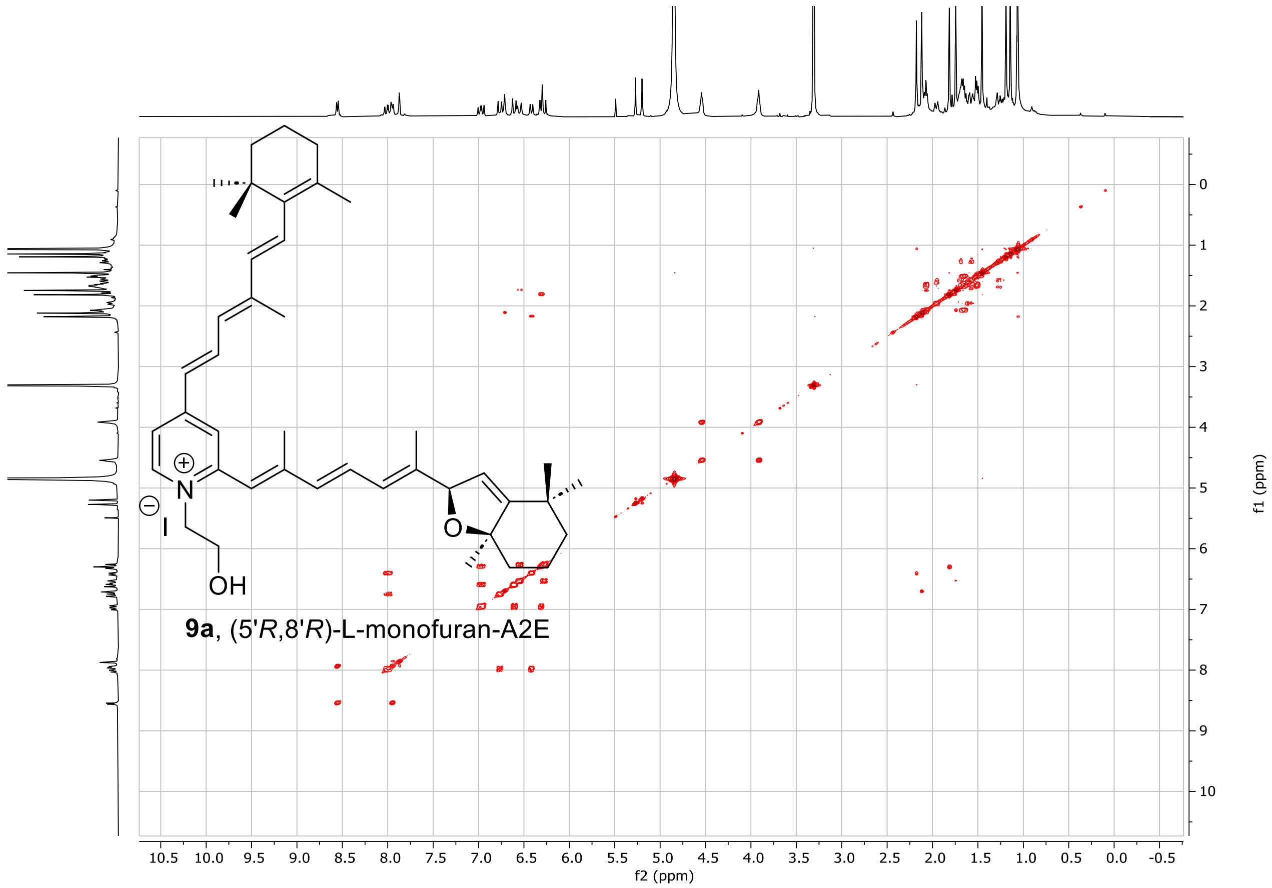

HSQC (CD$_3$OD)

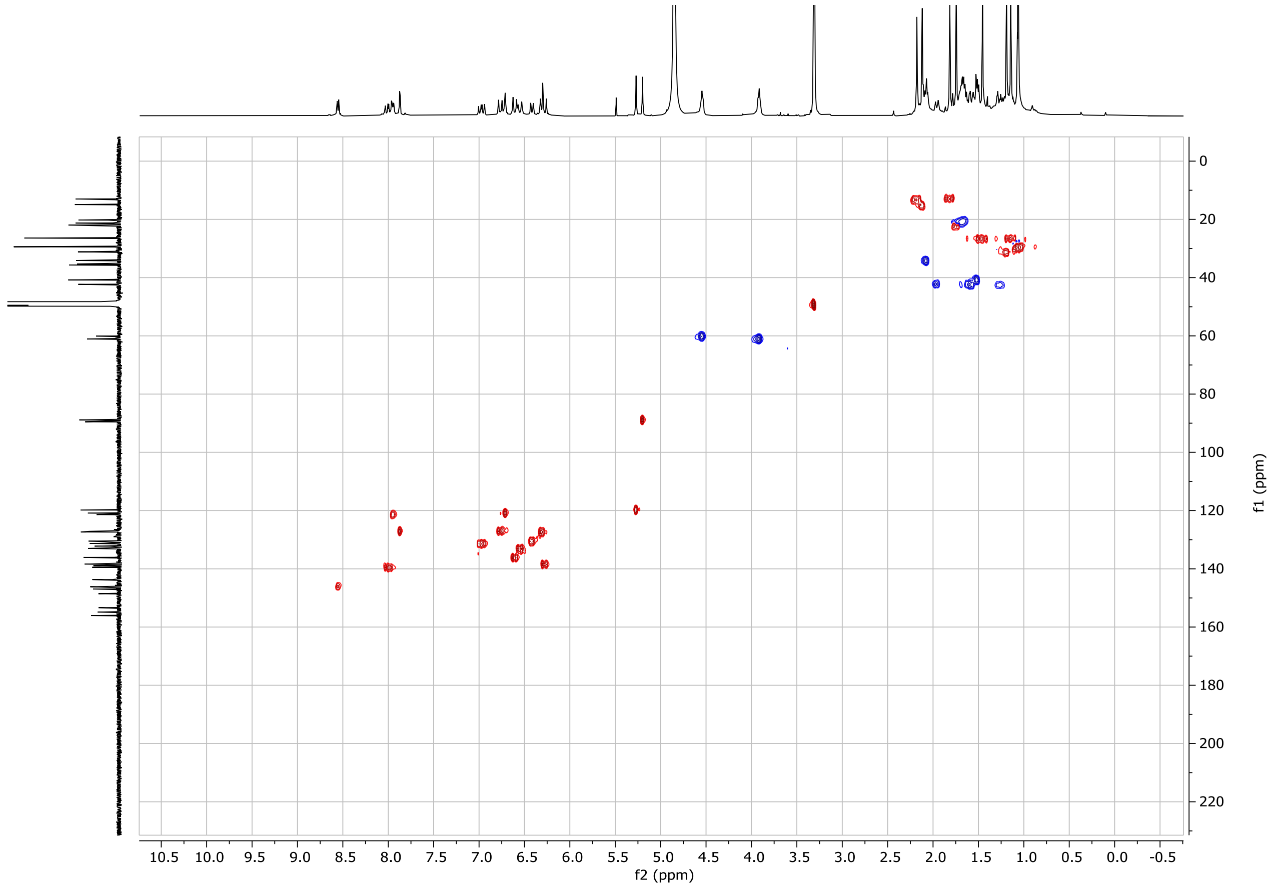

HMBC (CD_3OD)

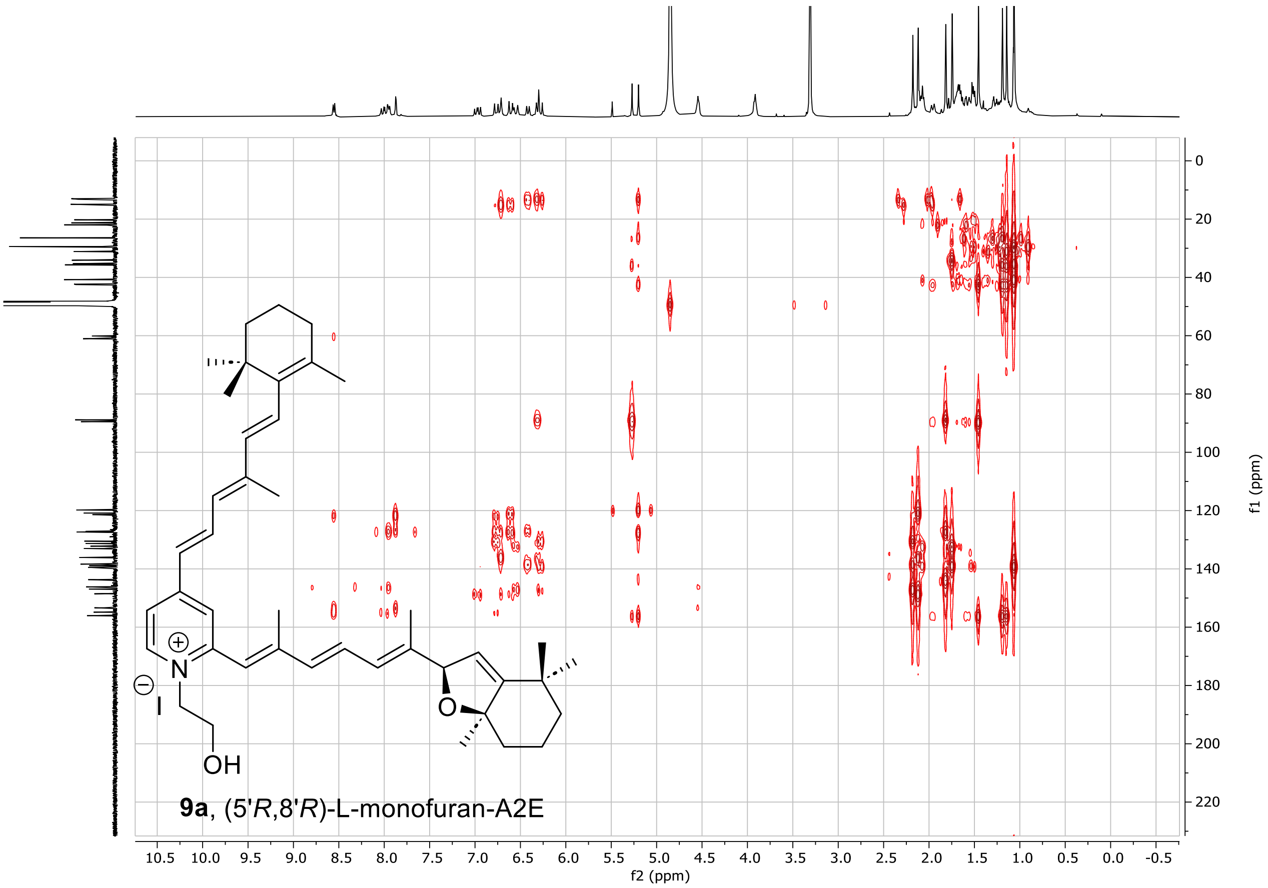

NOE-1D (400.16 MHz, freq. 6.98 ppm, CD_3OD)

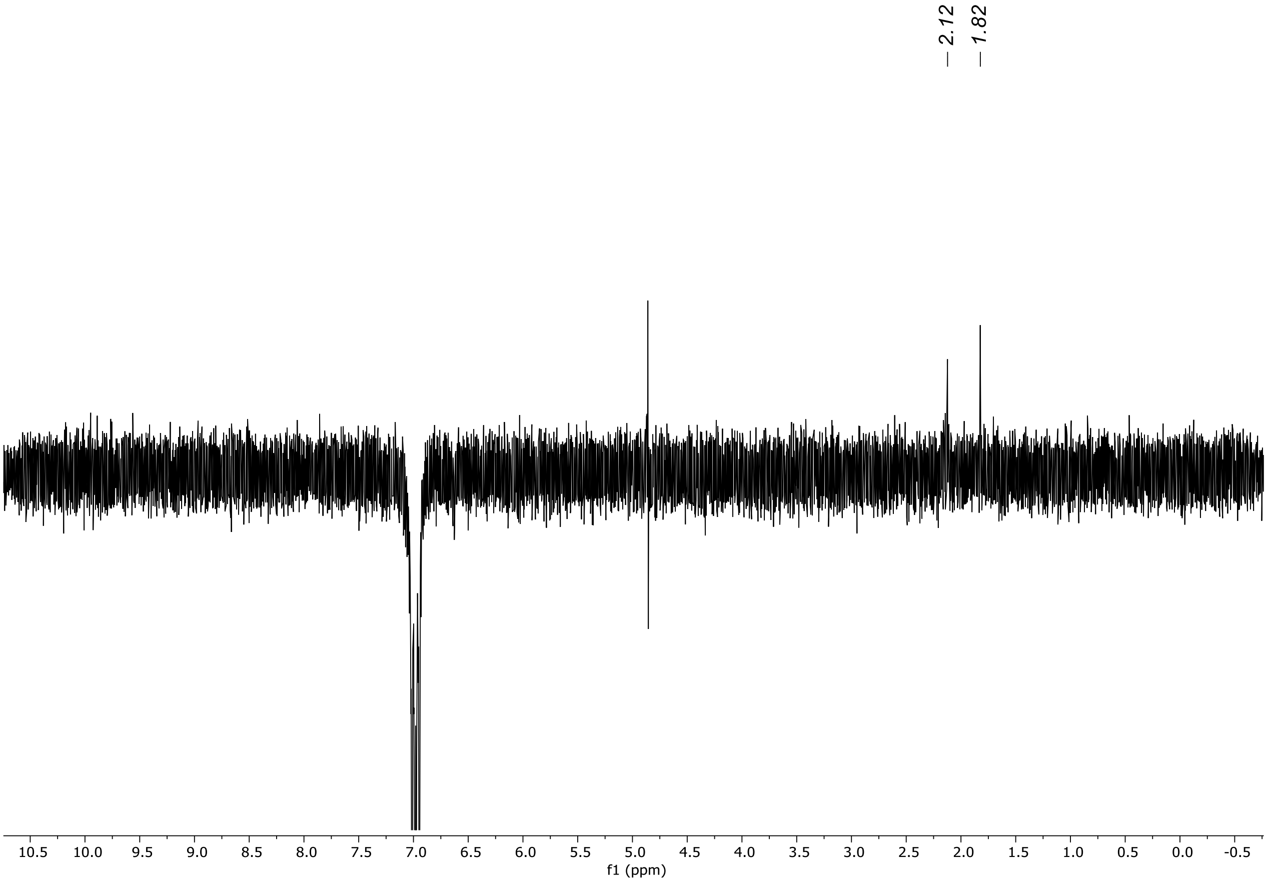

^{1}H-NMR (400.16 MHz, CD_3OD)

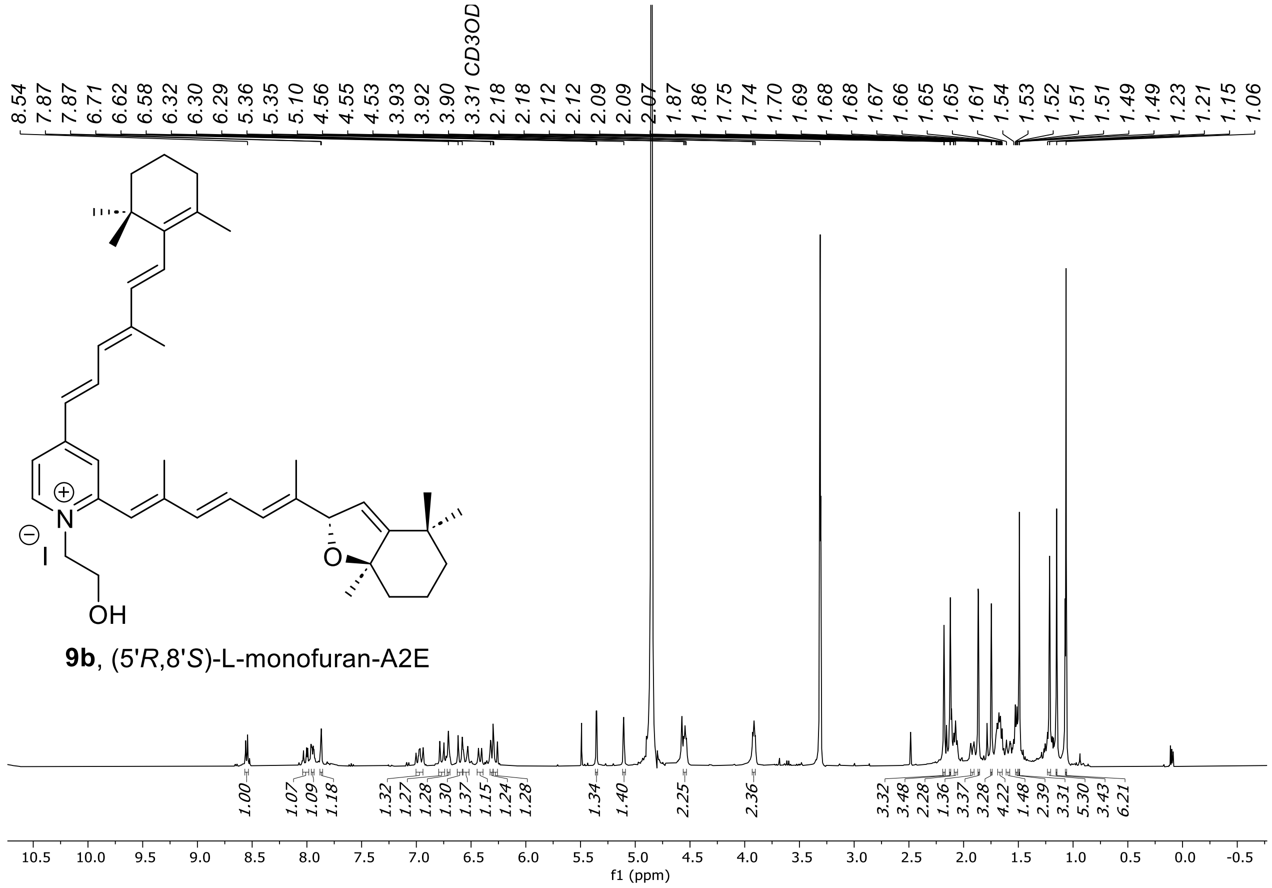

9b, (5'*R*,8'*S*)-L-monofuran-A2E

^{13}C{^{1}H}-NMR (100.63 MHz, CD_3OD)

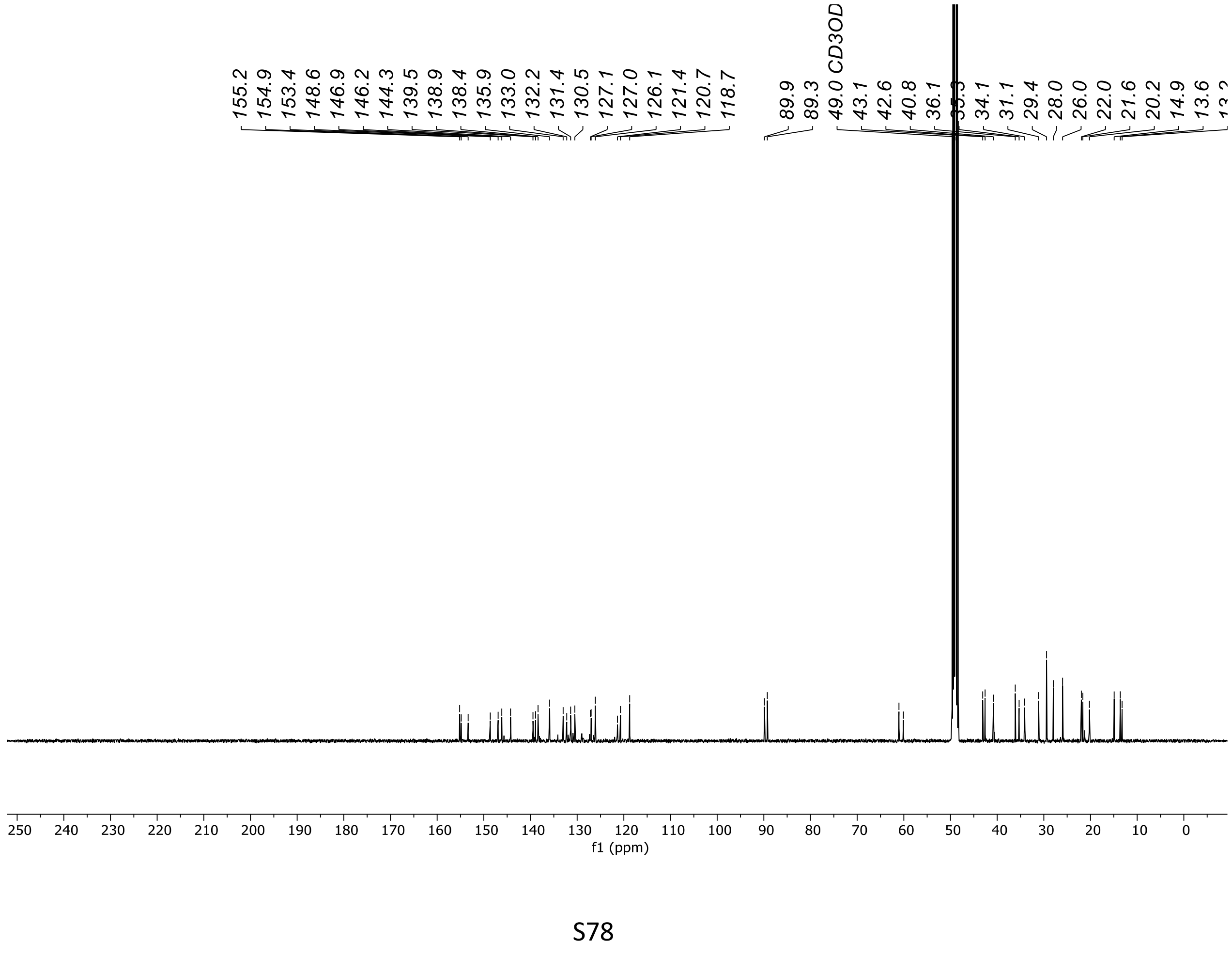

COSY (CD_3OD)

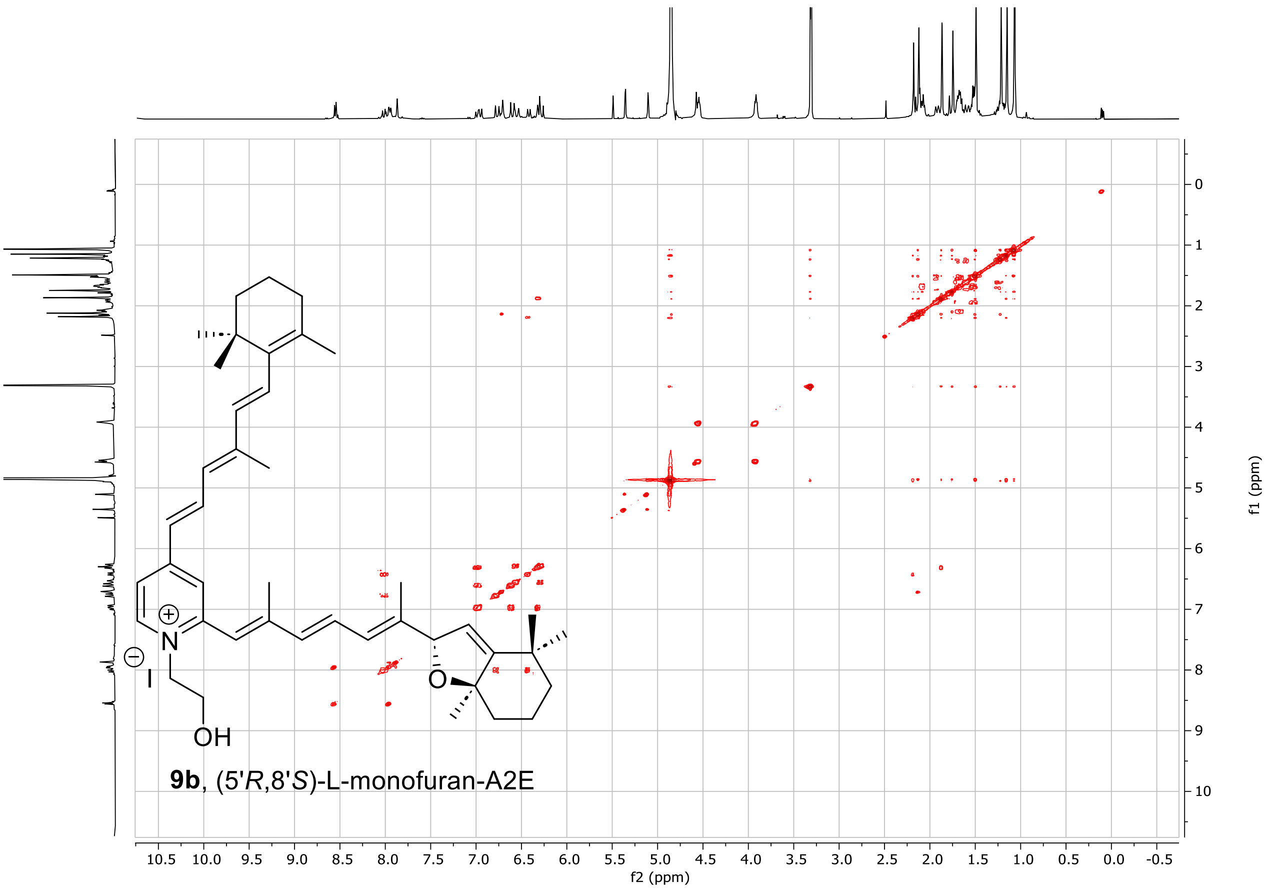

HSQC (CD_3OD)

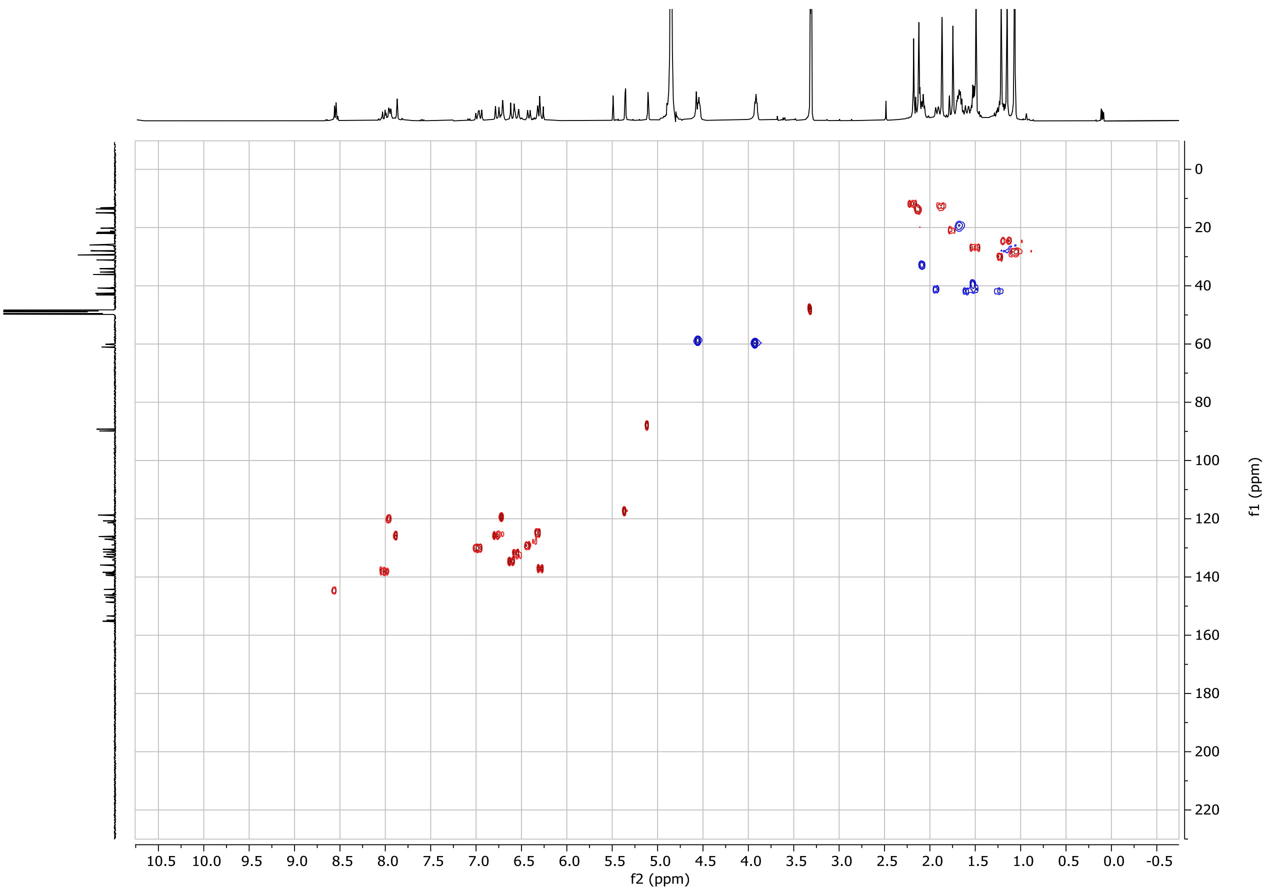

HMBC (CD_3OD)

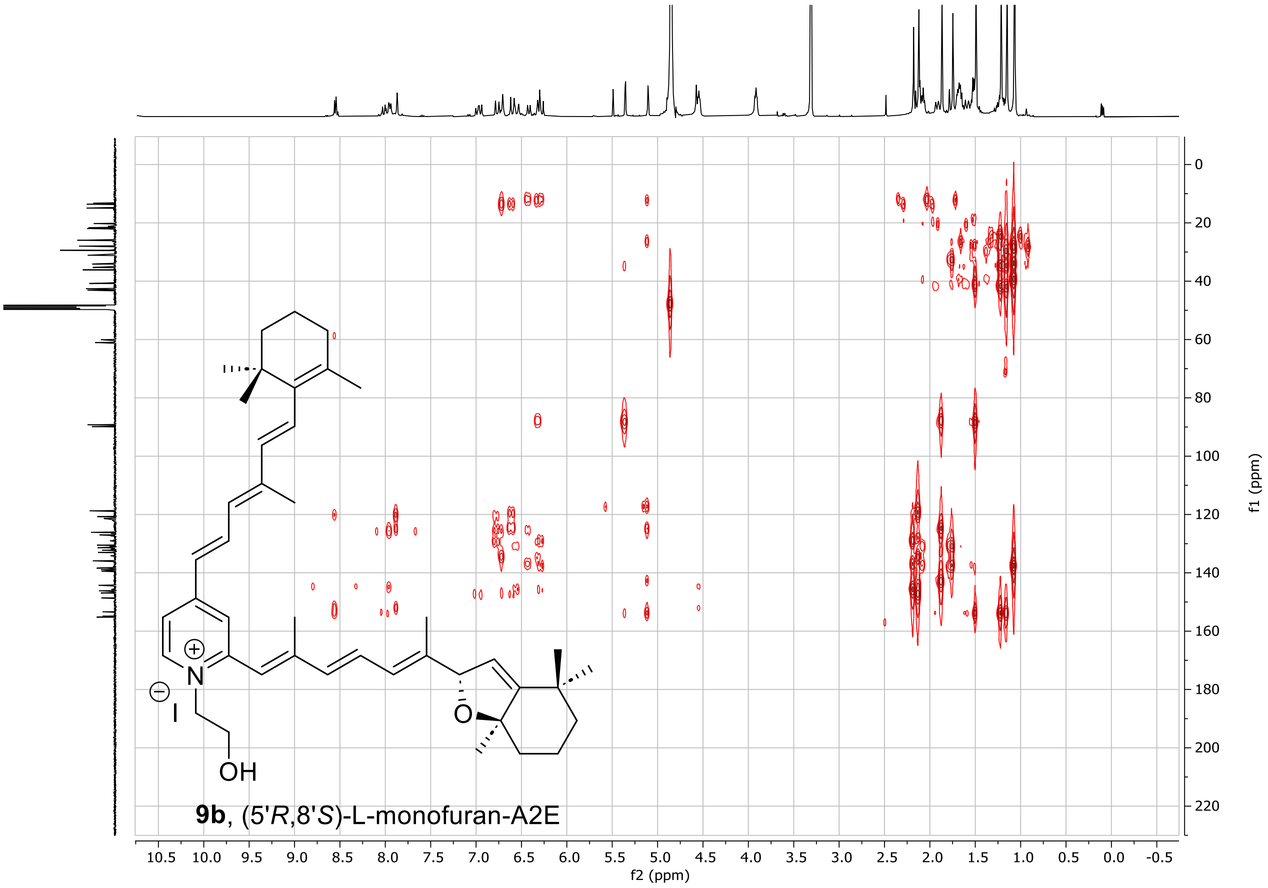

NOE-1D (400.16 MHz, freq. 6.98 ppm, CD_3OD)

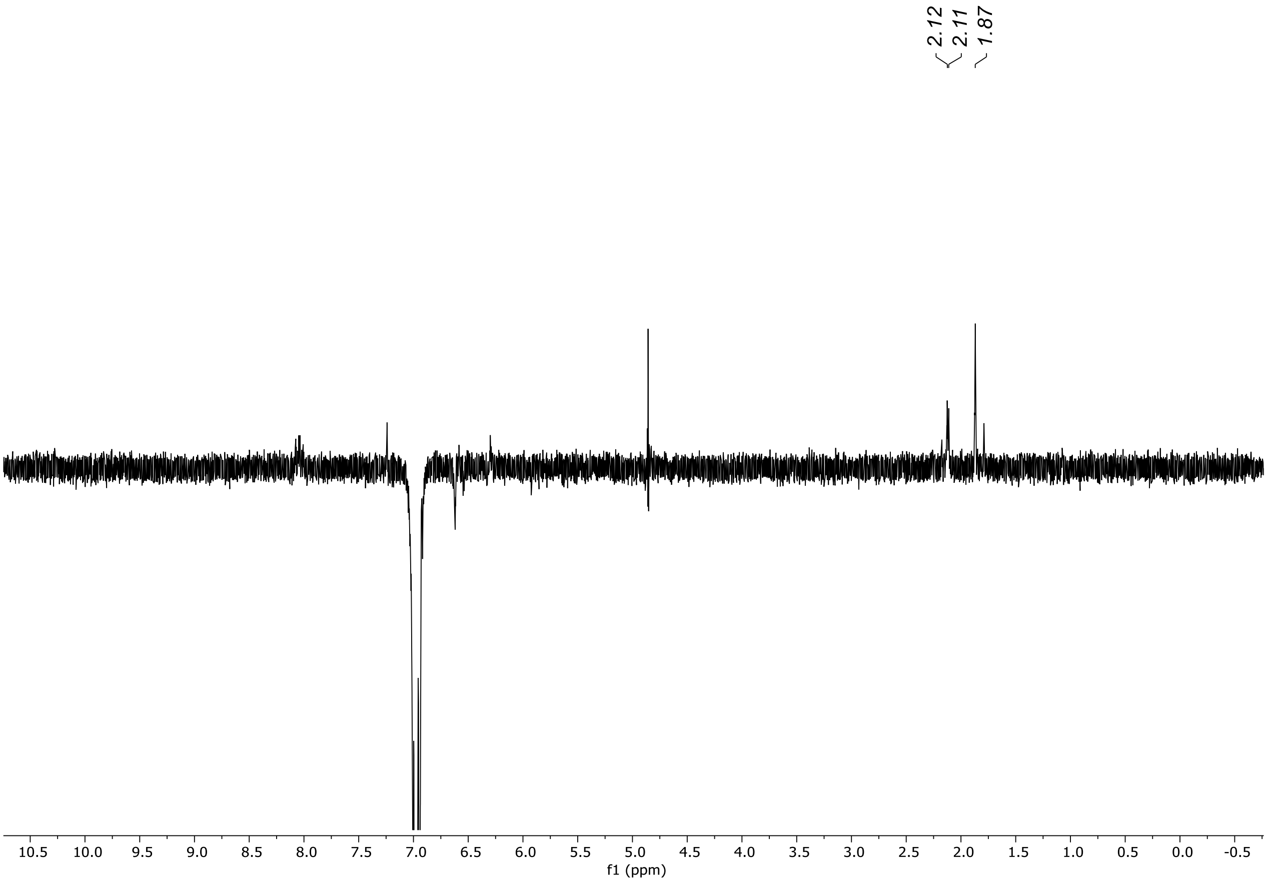

^{1}H-NMR (400.16 MHz, C_6D_6)

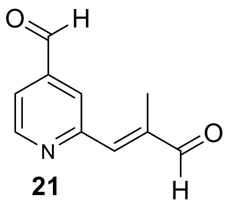

21

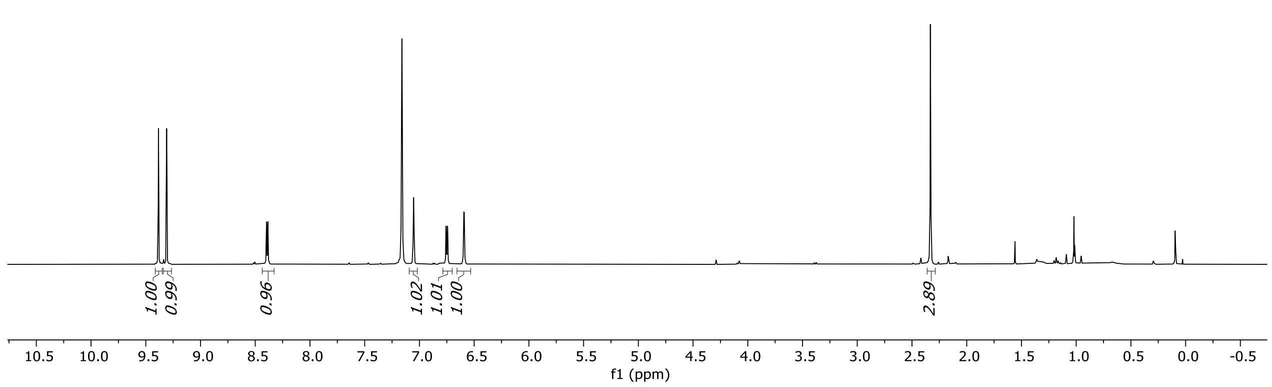

^{13}C{^{1}H}-NMR (100.63 MHz, C_6D_6)

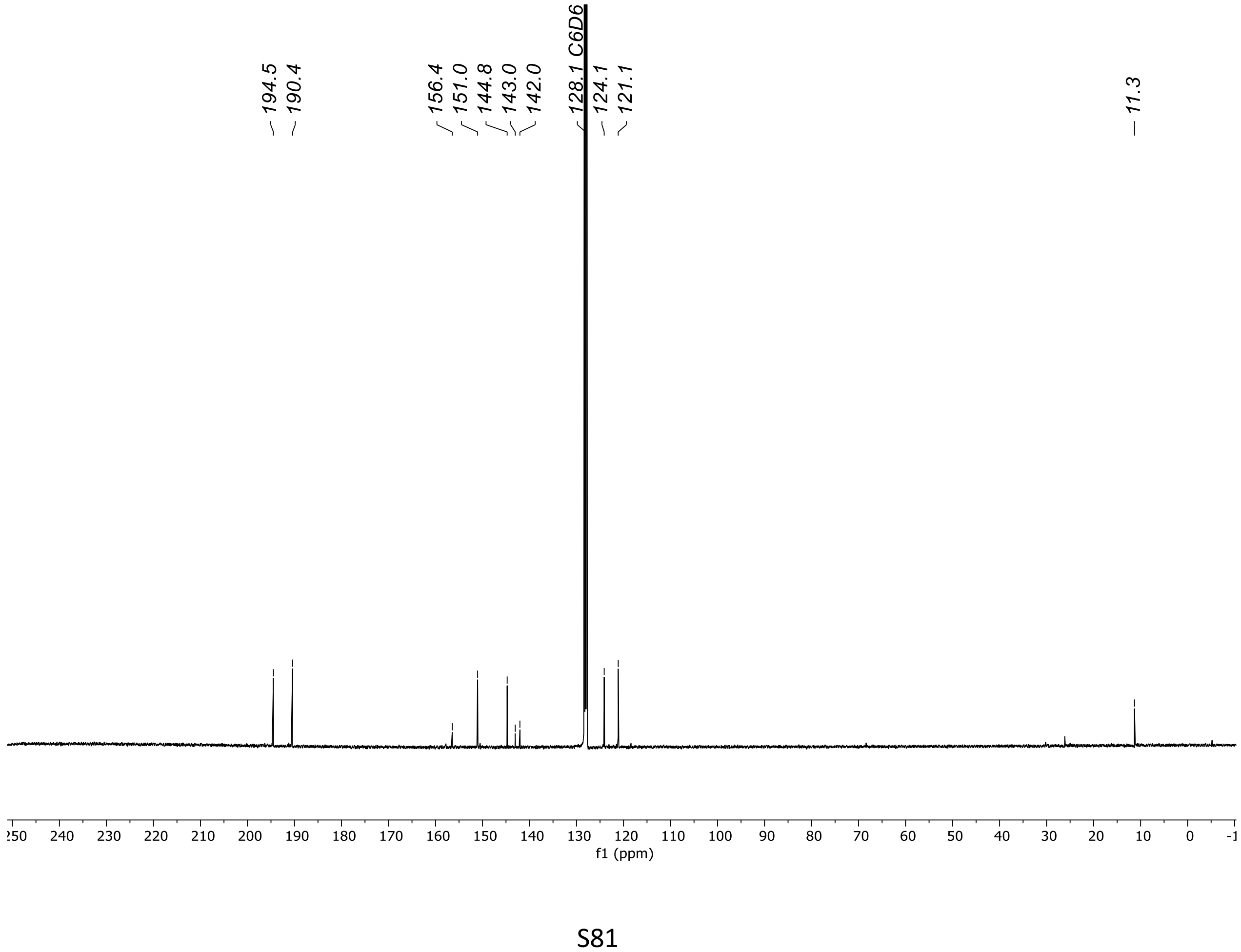

^{1}H-NMR (400.16 MHz, CD_2Cl_2)

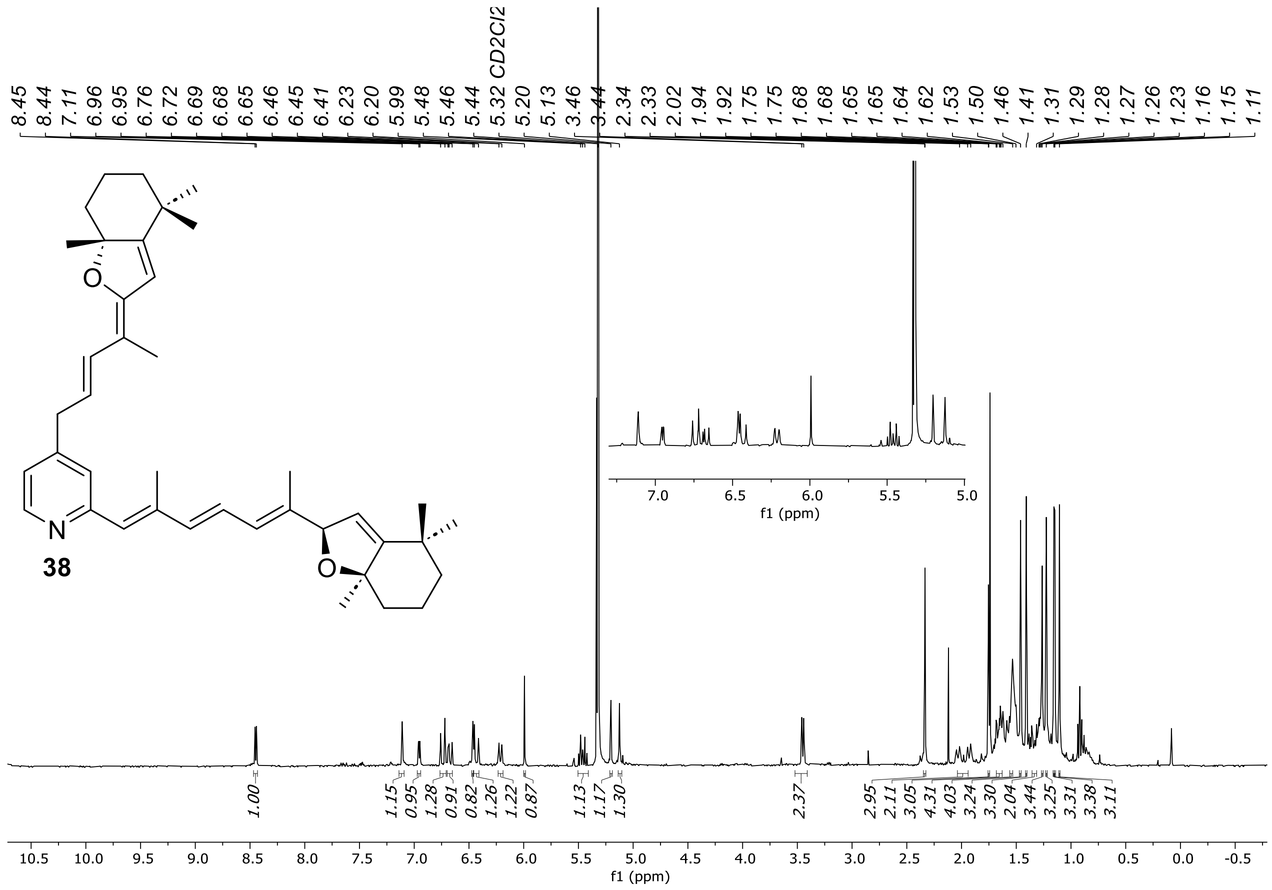

^{13}C{^{1}H}-NMR (100.63 MHz, CD_2Cl_2)

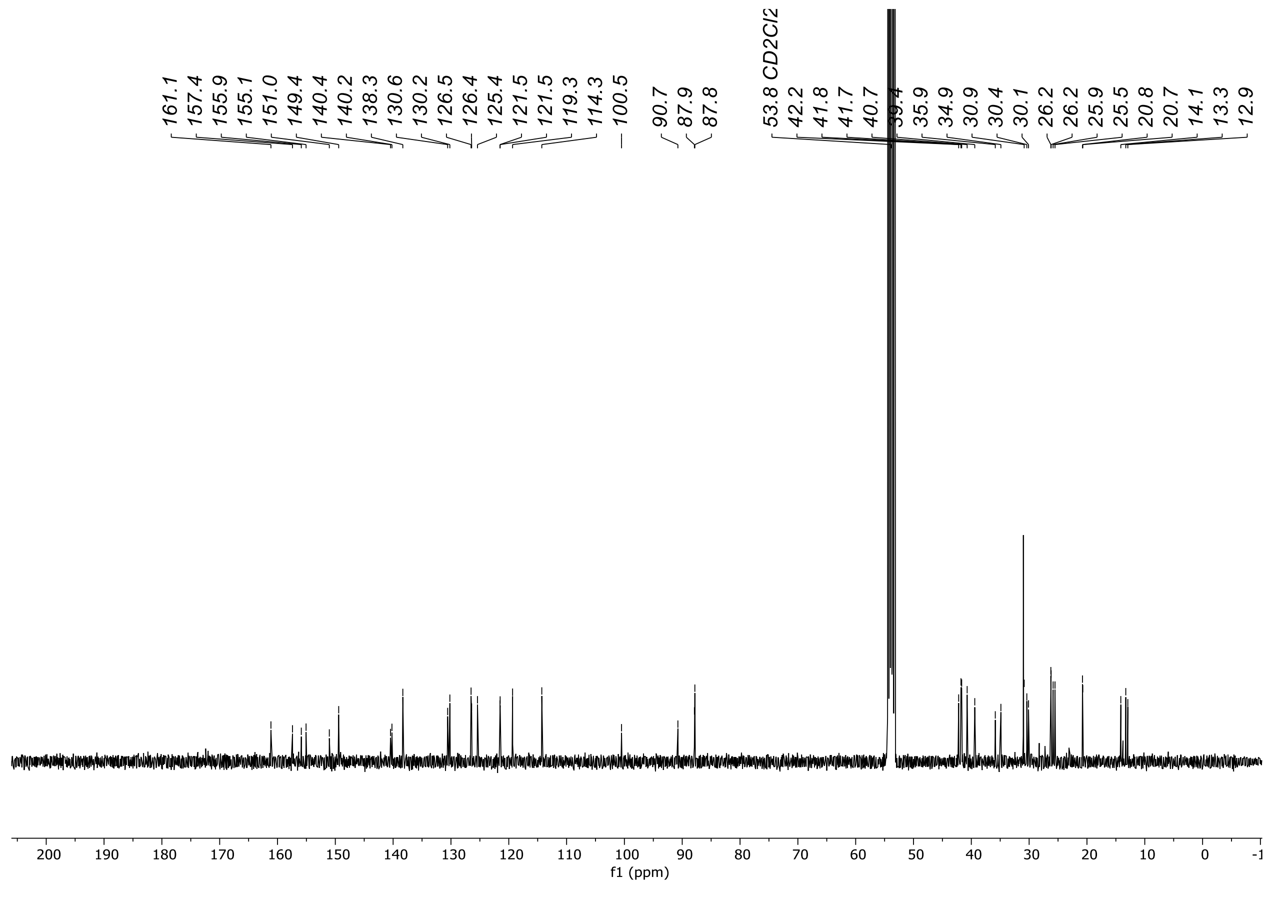

COSY (CD_2Cl_2)

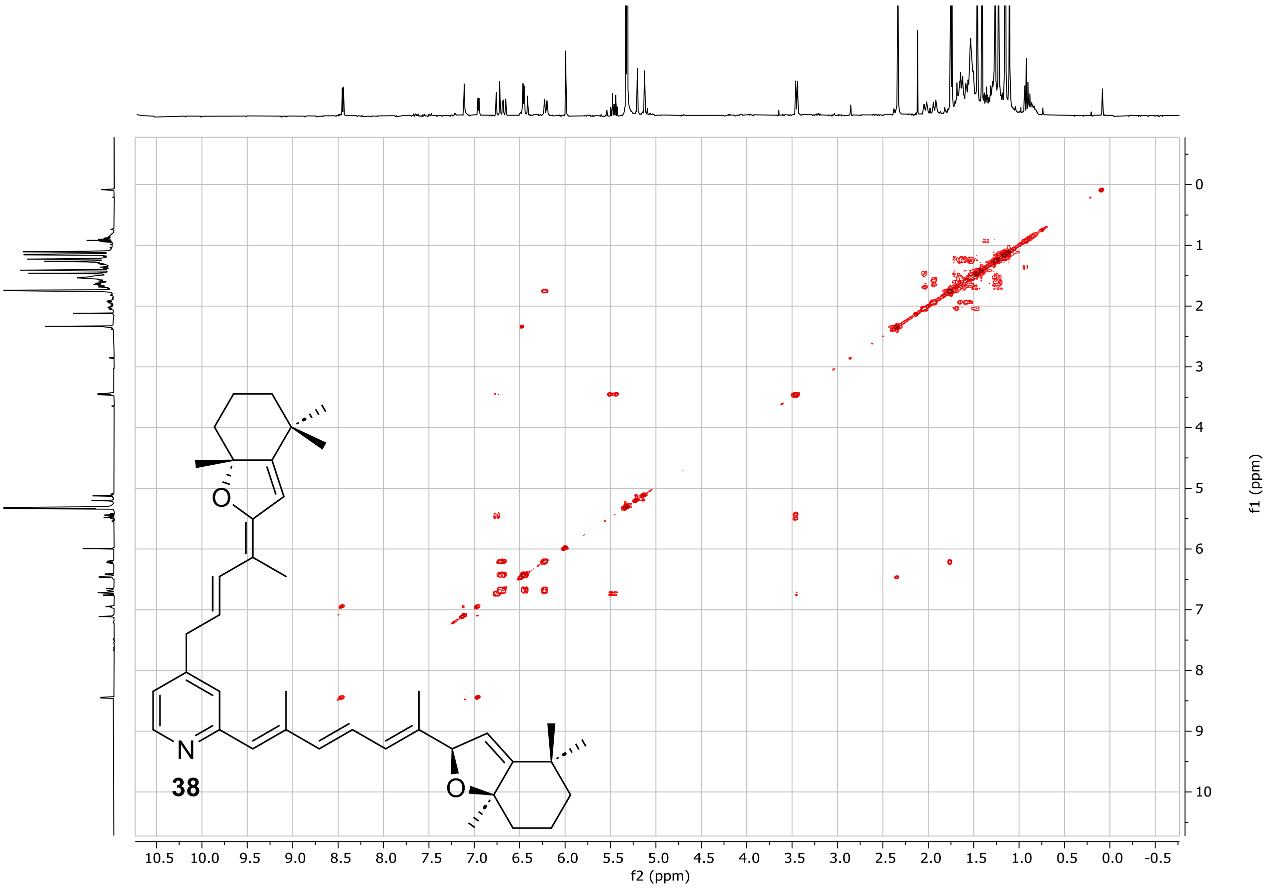

HSQC (CD_2Cl_2)

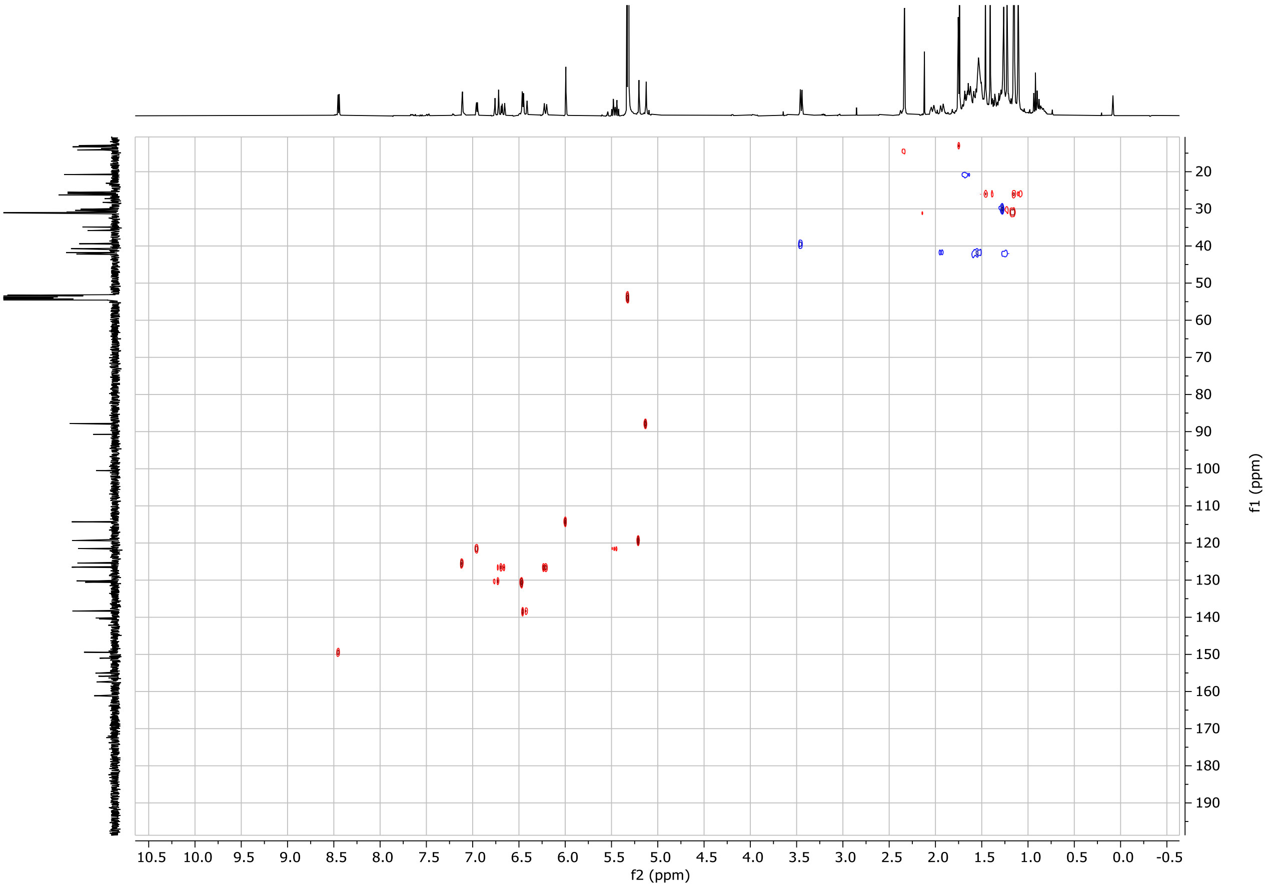

HMBC (CD_2Cl_2)

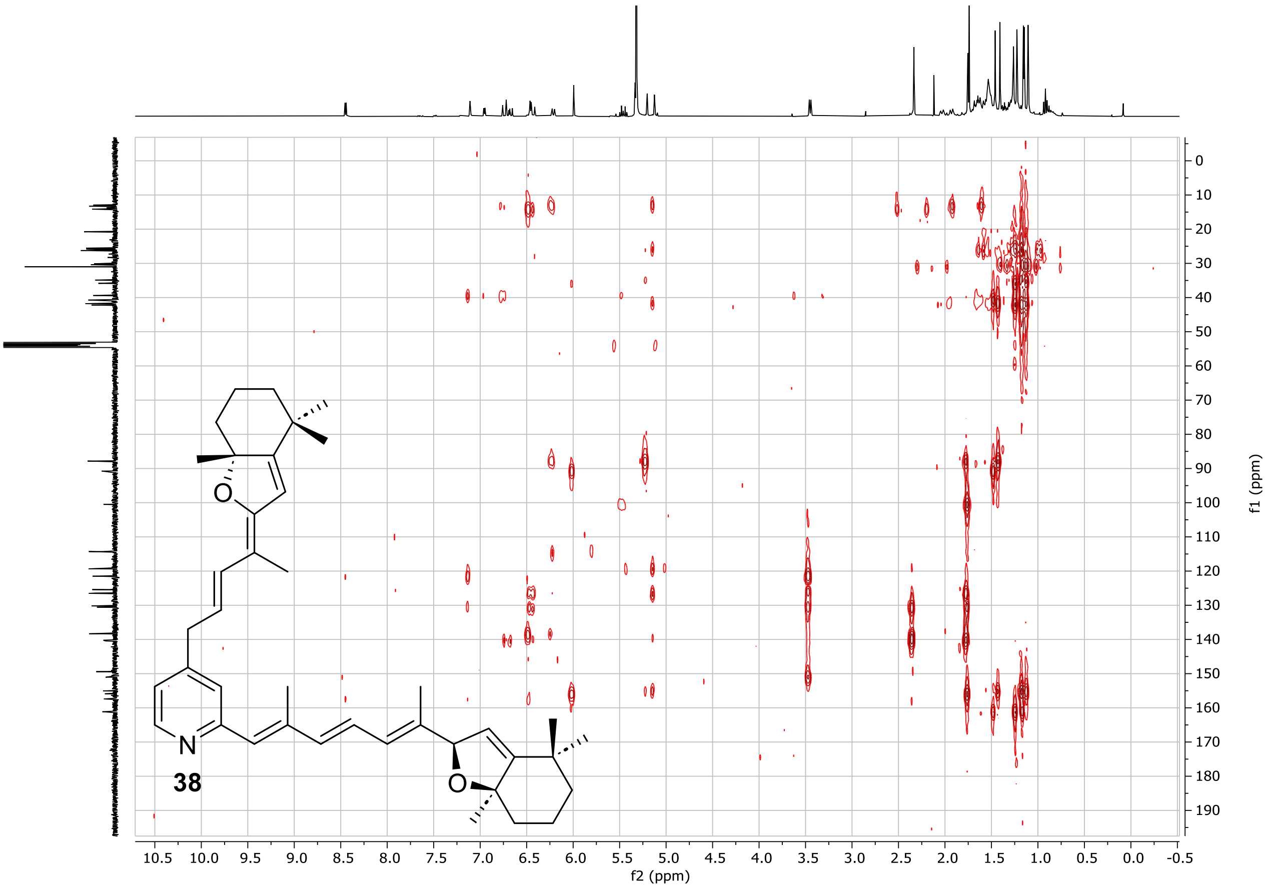

NOE-1D (400.16 MHz, freq. 5.13 ppm, CD_2Cl_2)

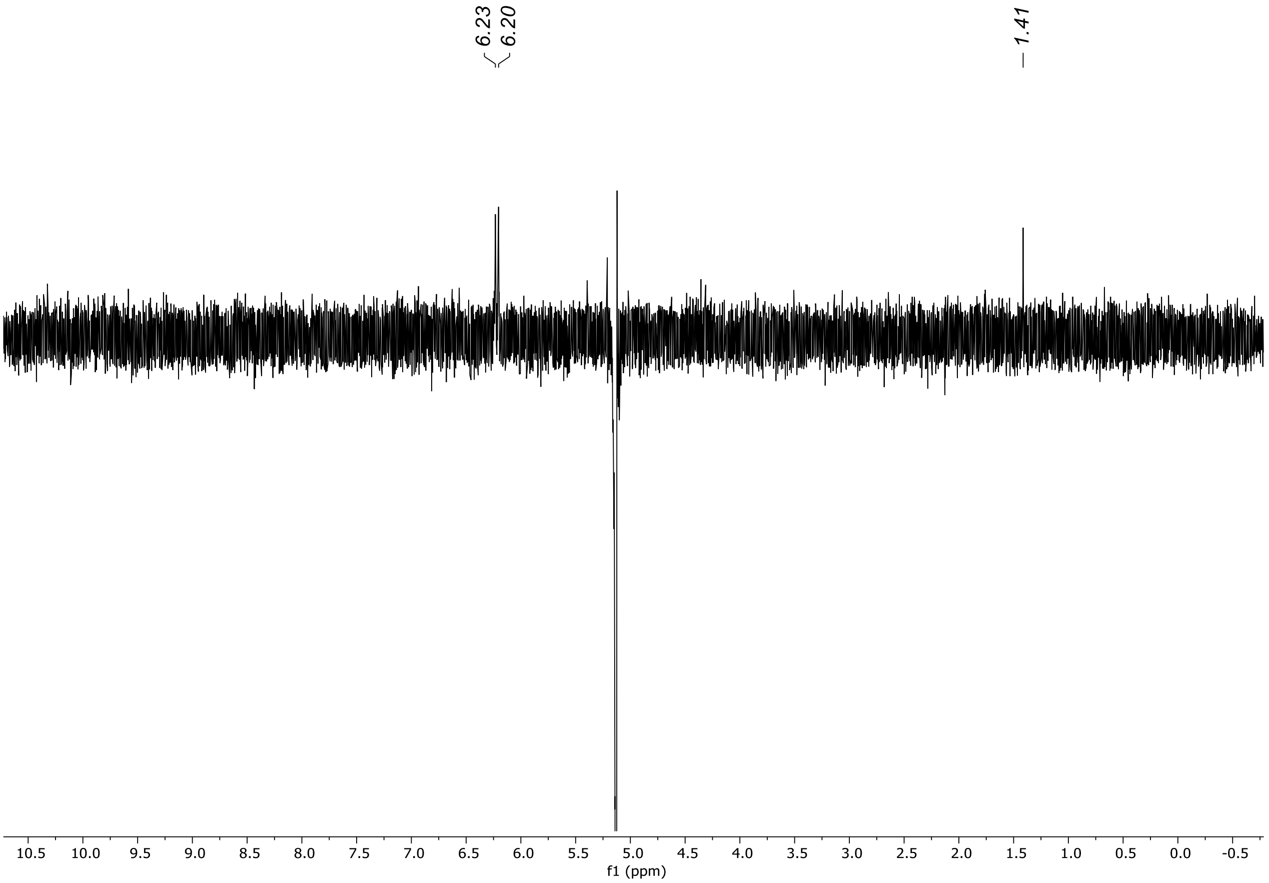

^{1}H-NMR (400.16 MHz, CD_2Cl_2)

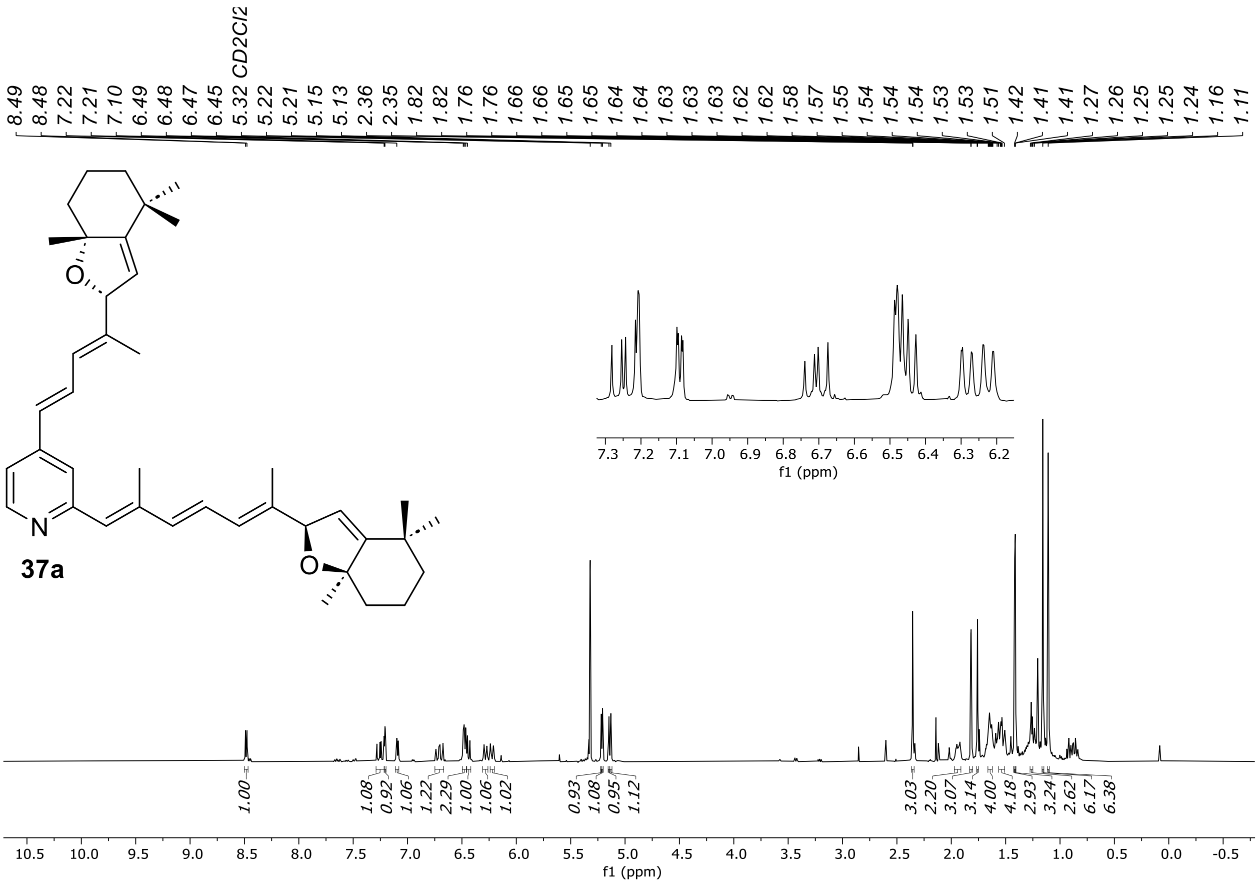

$^{13}C\{^{1}H\}$-NMR (100.63 MHz, CD_2Cl_2)

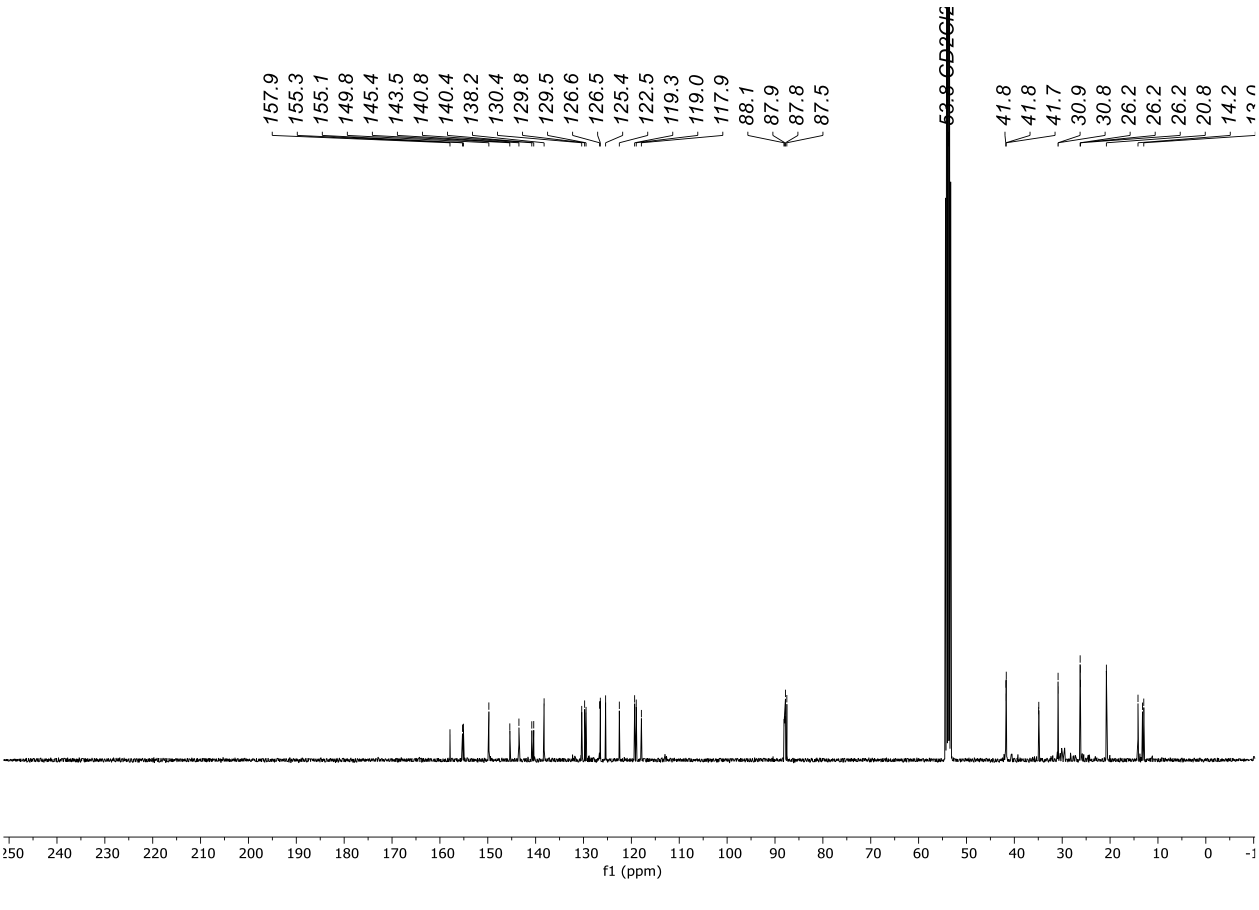

COSY (CD_2Cl_2)

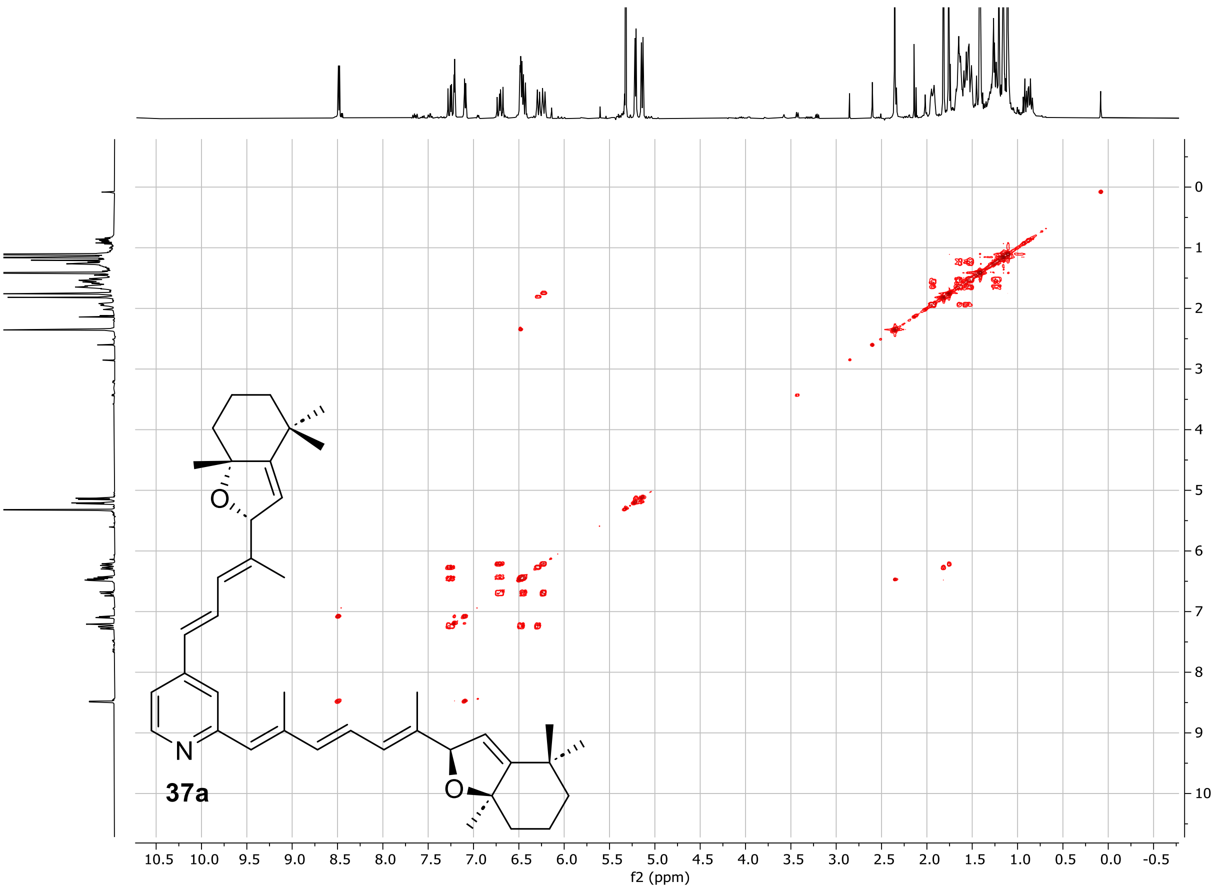

HSQC (CD_2Cl_2)

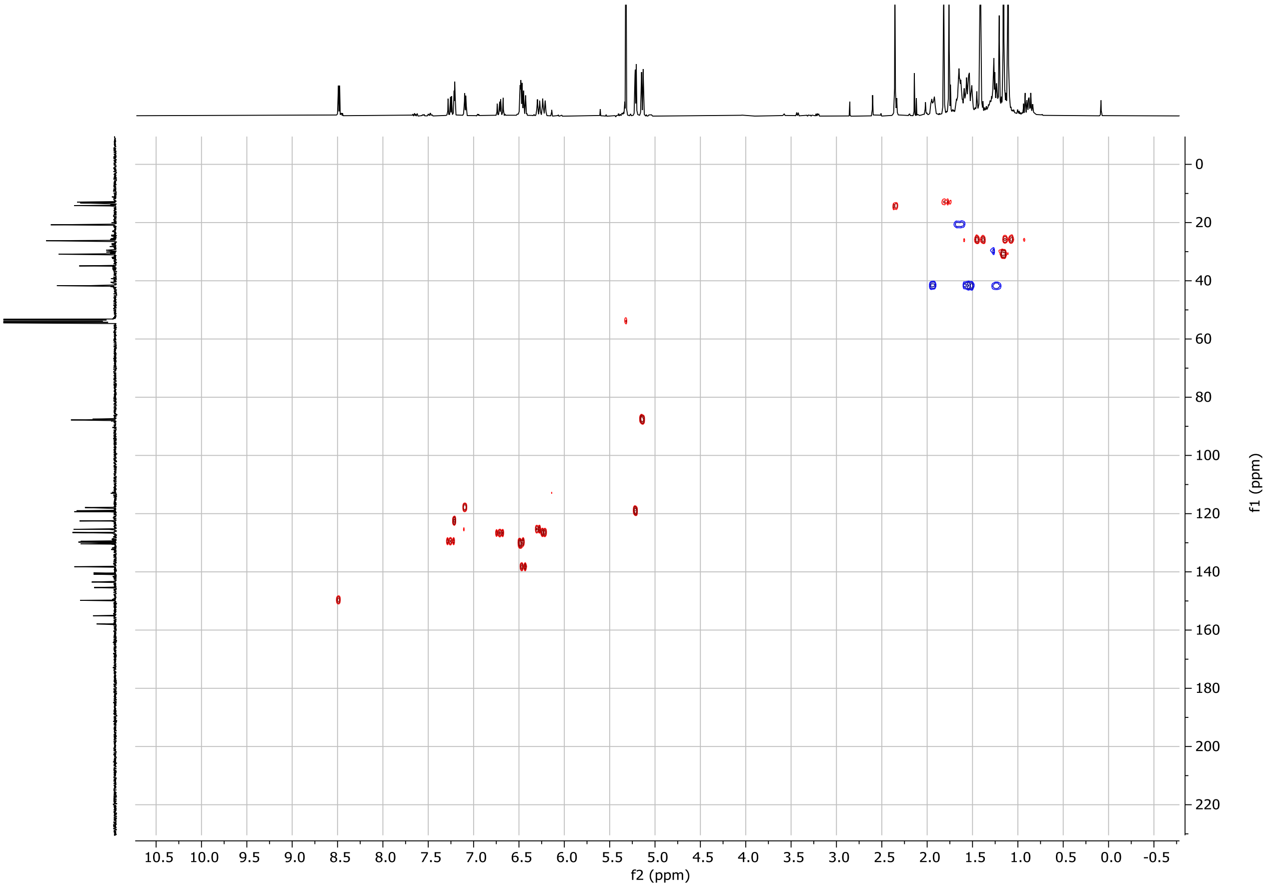

HMBC (CD_2Cl_2)

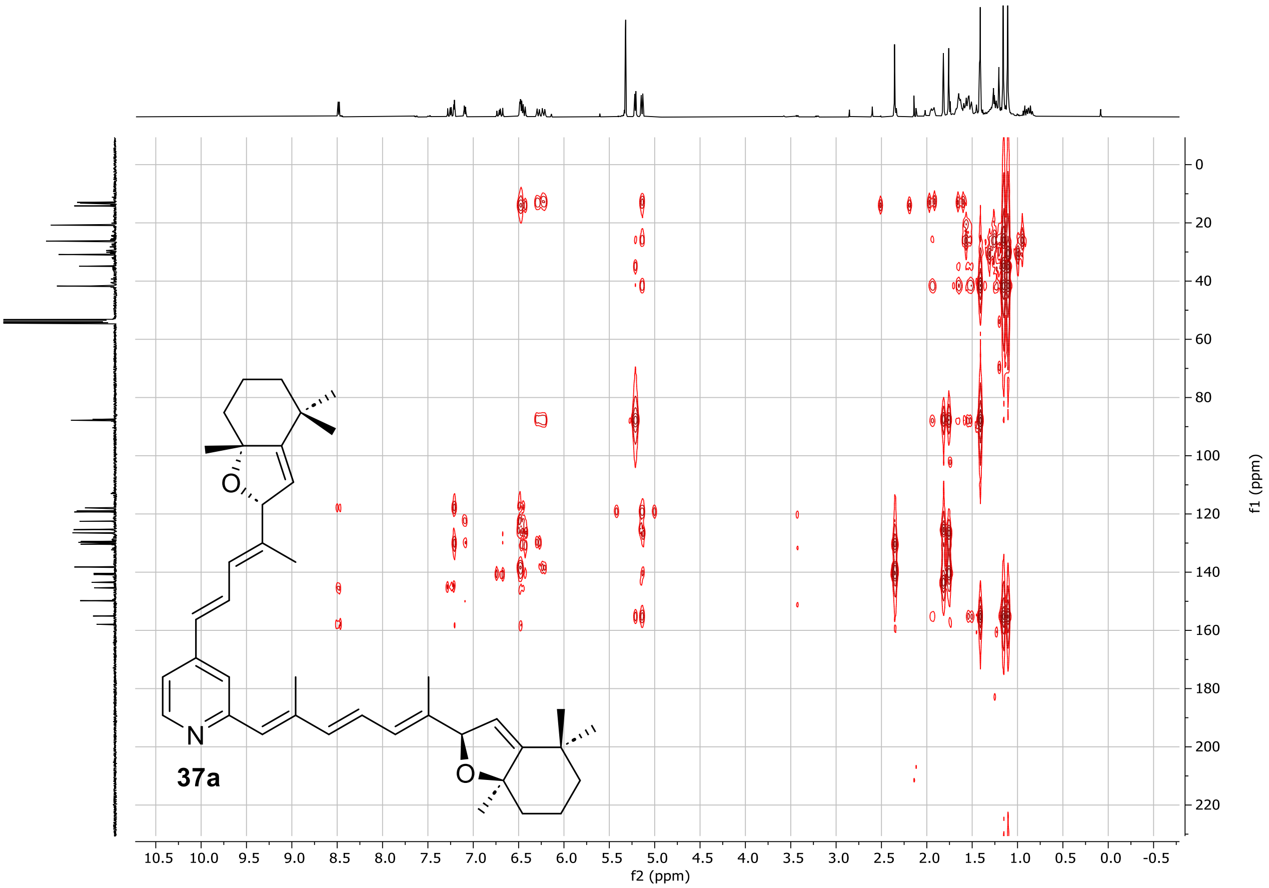

NOE-1D (400.16 MHz, freq. 5.15 ppm, CD_2Cl_2)

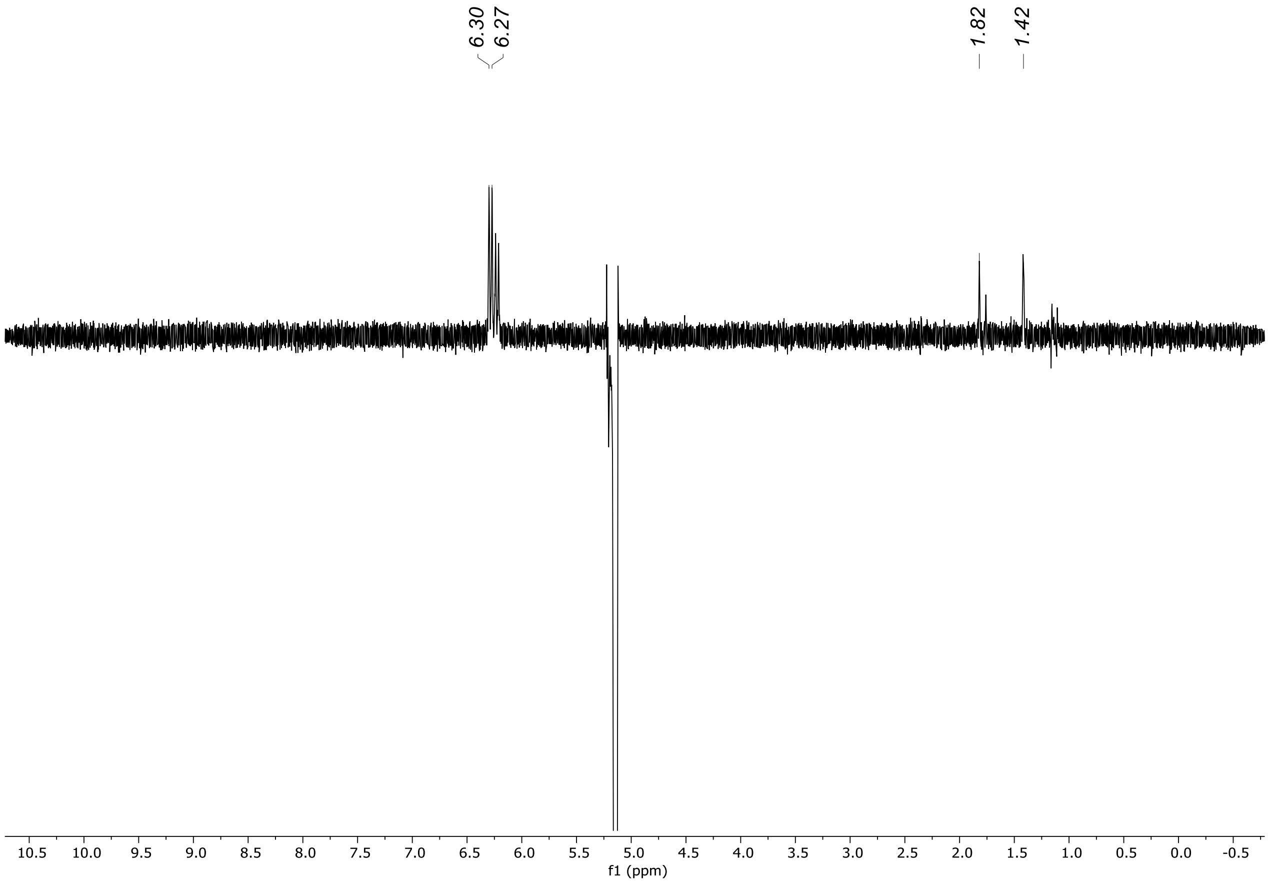

NOE-1D (400.16 MHz, freq. 5.13 ppm, CD_2Cl_2)

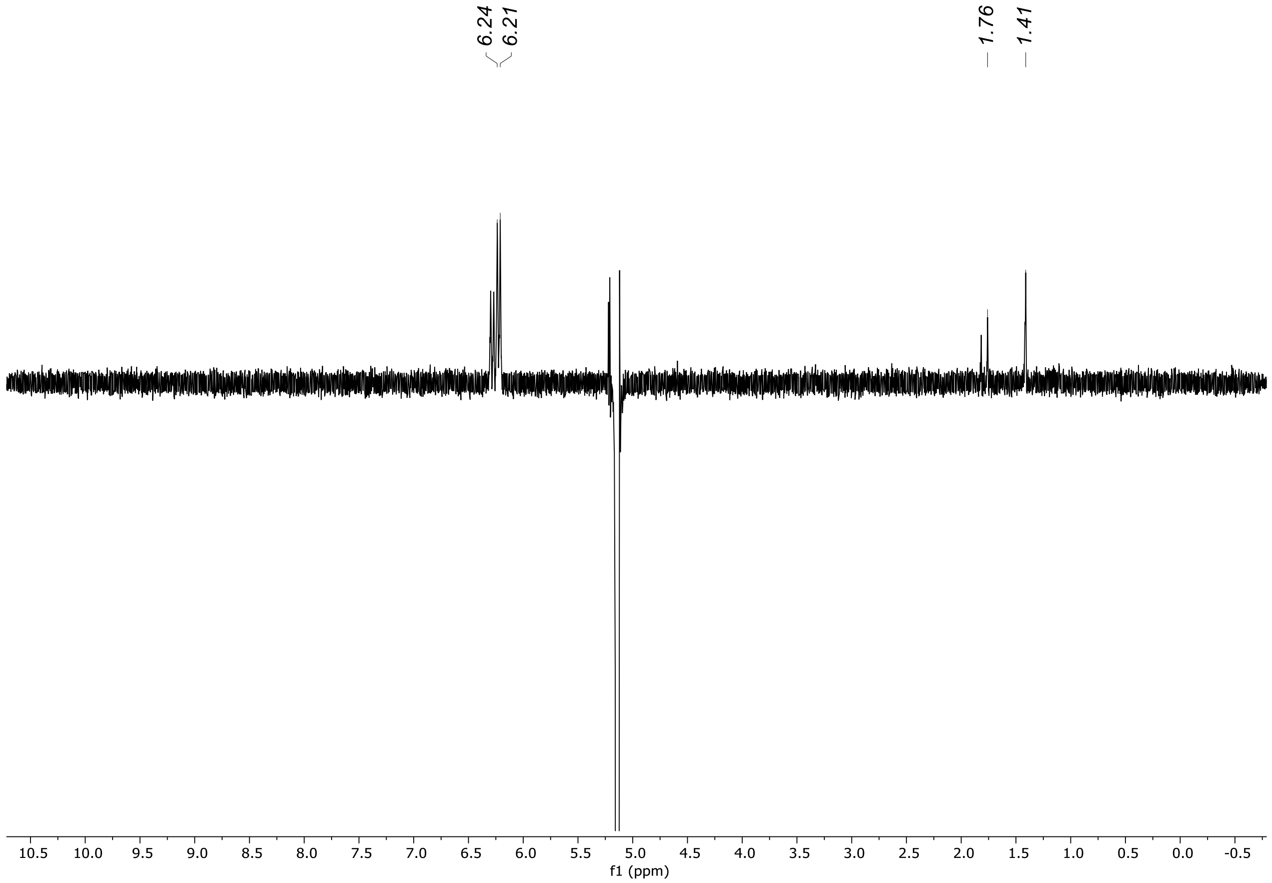

^{1}H-NMR (400.16 MHz, CD_2Cl_2)

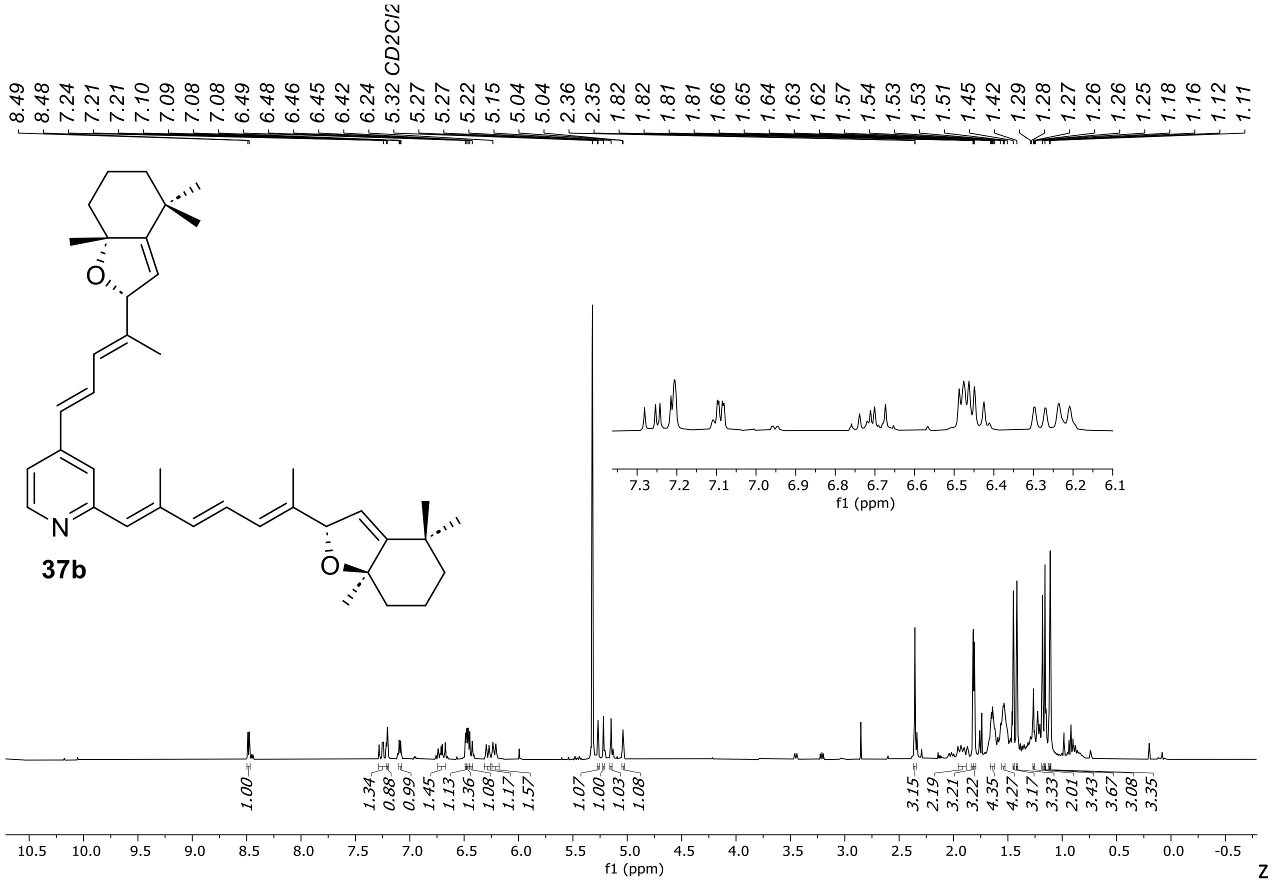

^{13}C{^{1}H}-NMR (100.63 MHz, CD_2Cl_2)

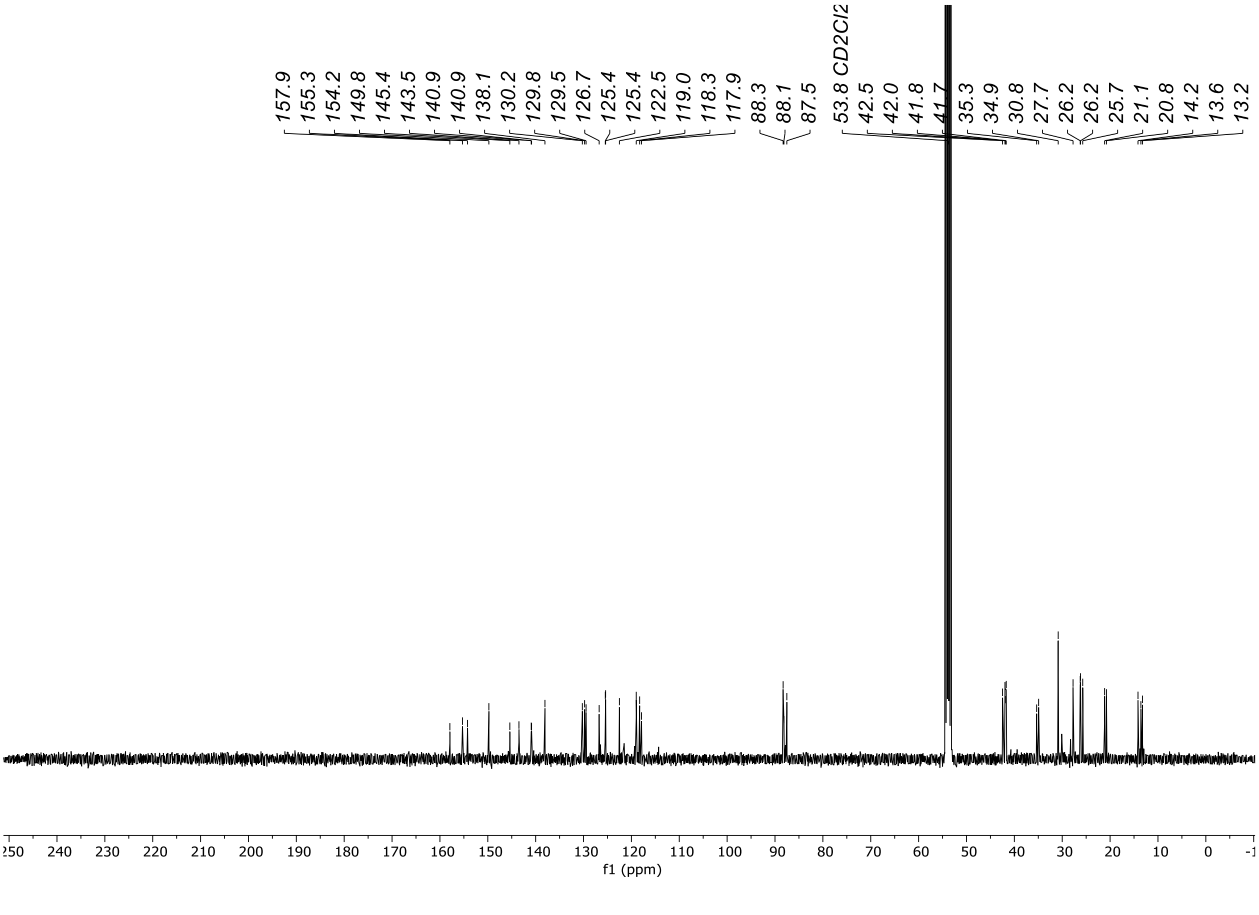

COSY (CD_2Cl_2)

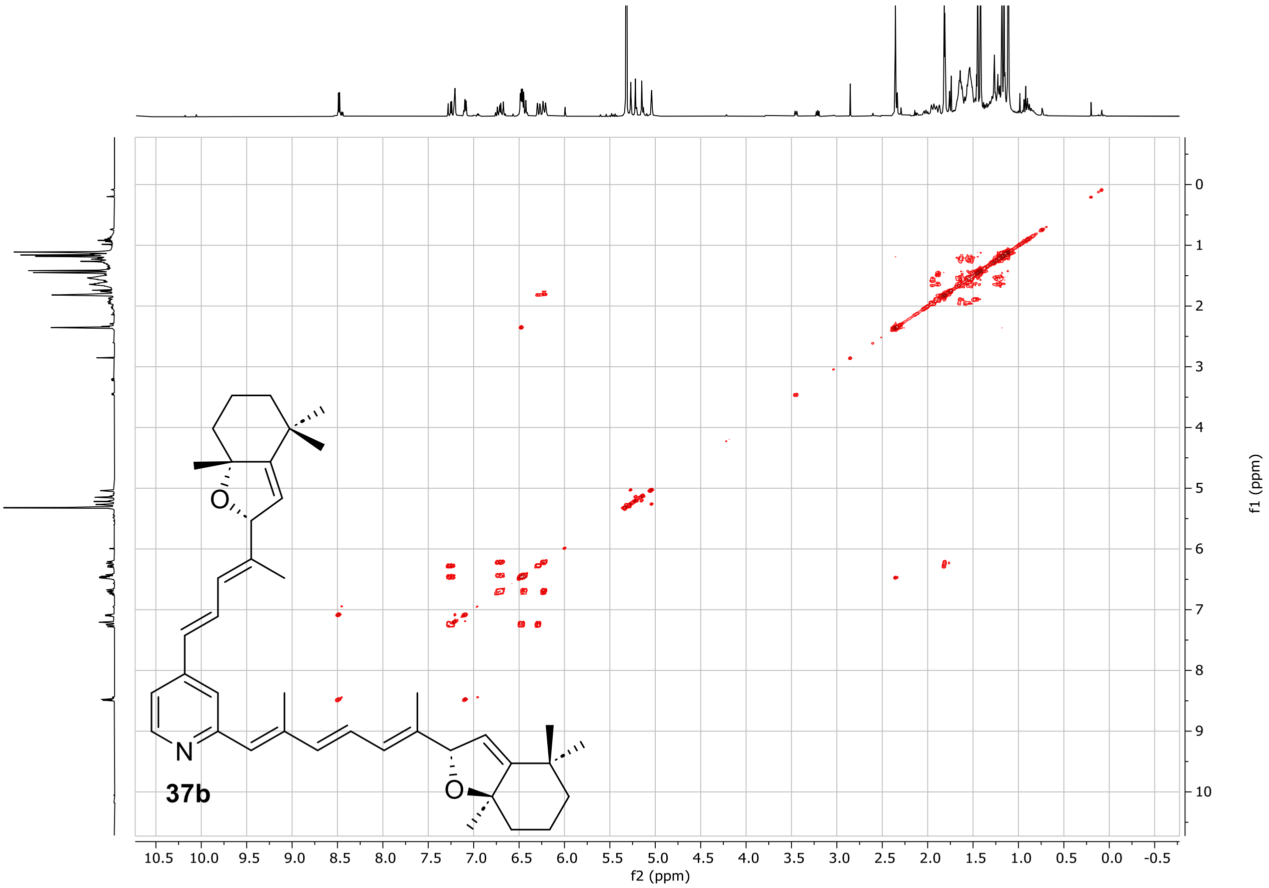

HSQC (CD_2Cl_2)

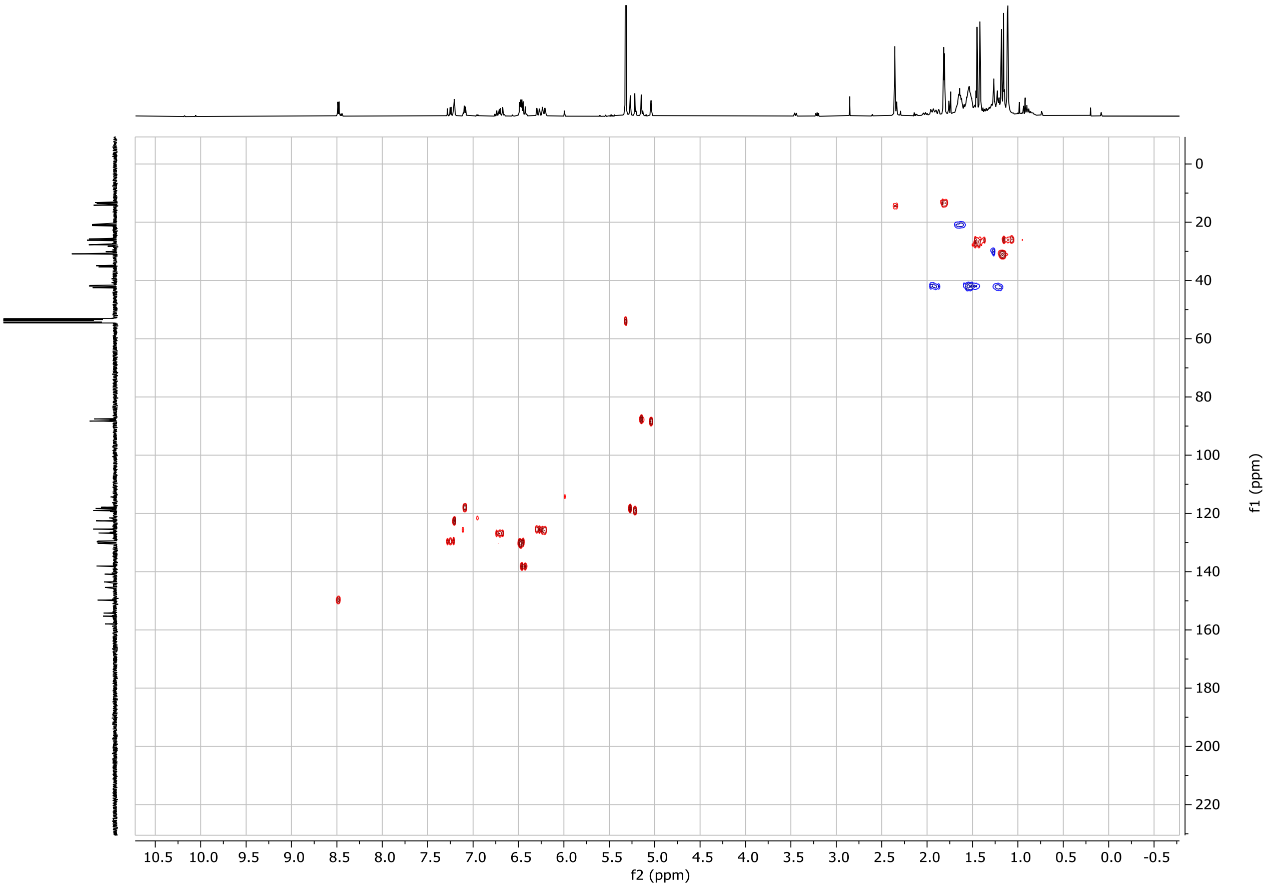

HMBC (CD_2Cl_2)

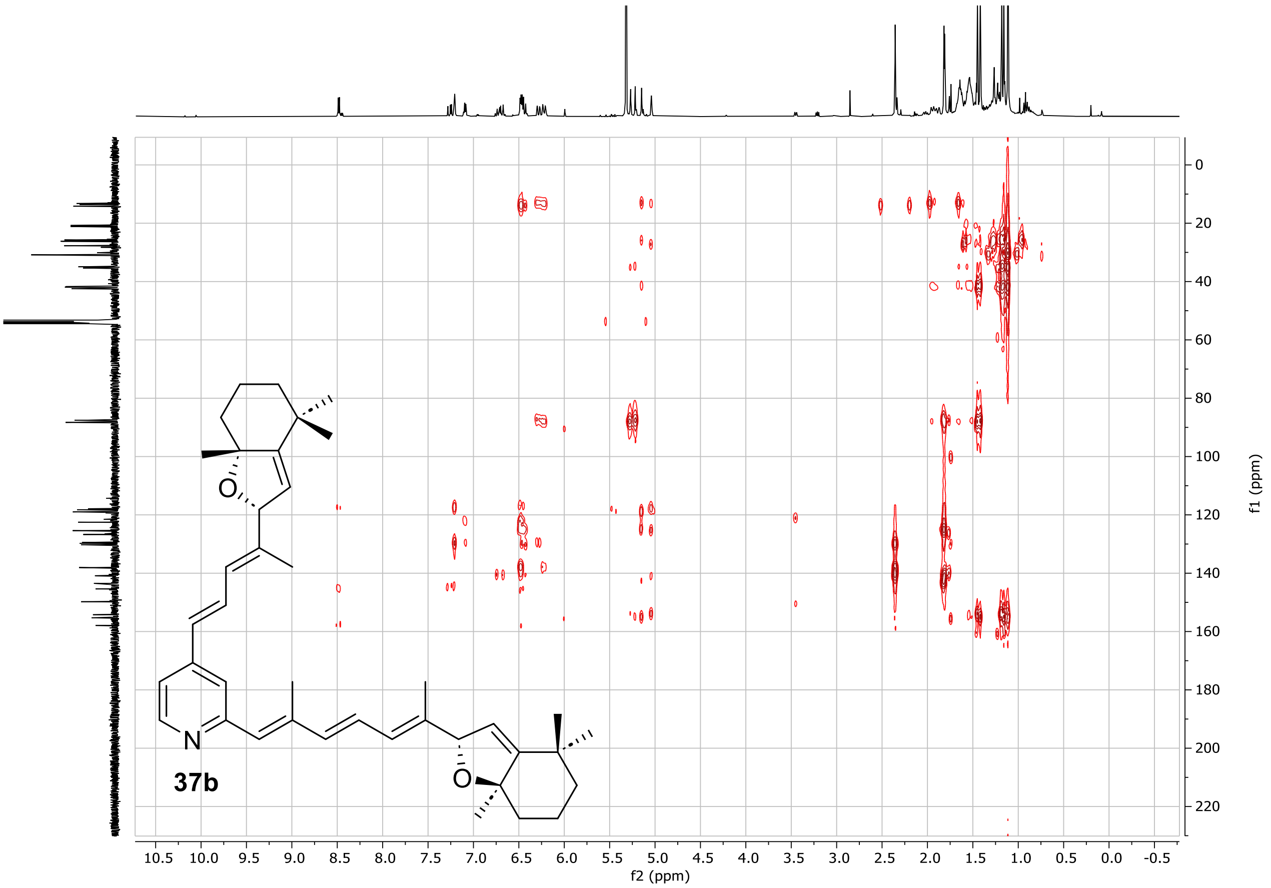

NOE-1D (400.16 MHz, freq. 5.15 ppm, CD_2Cl_2)

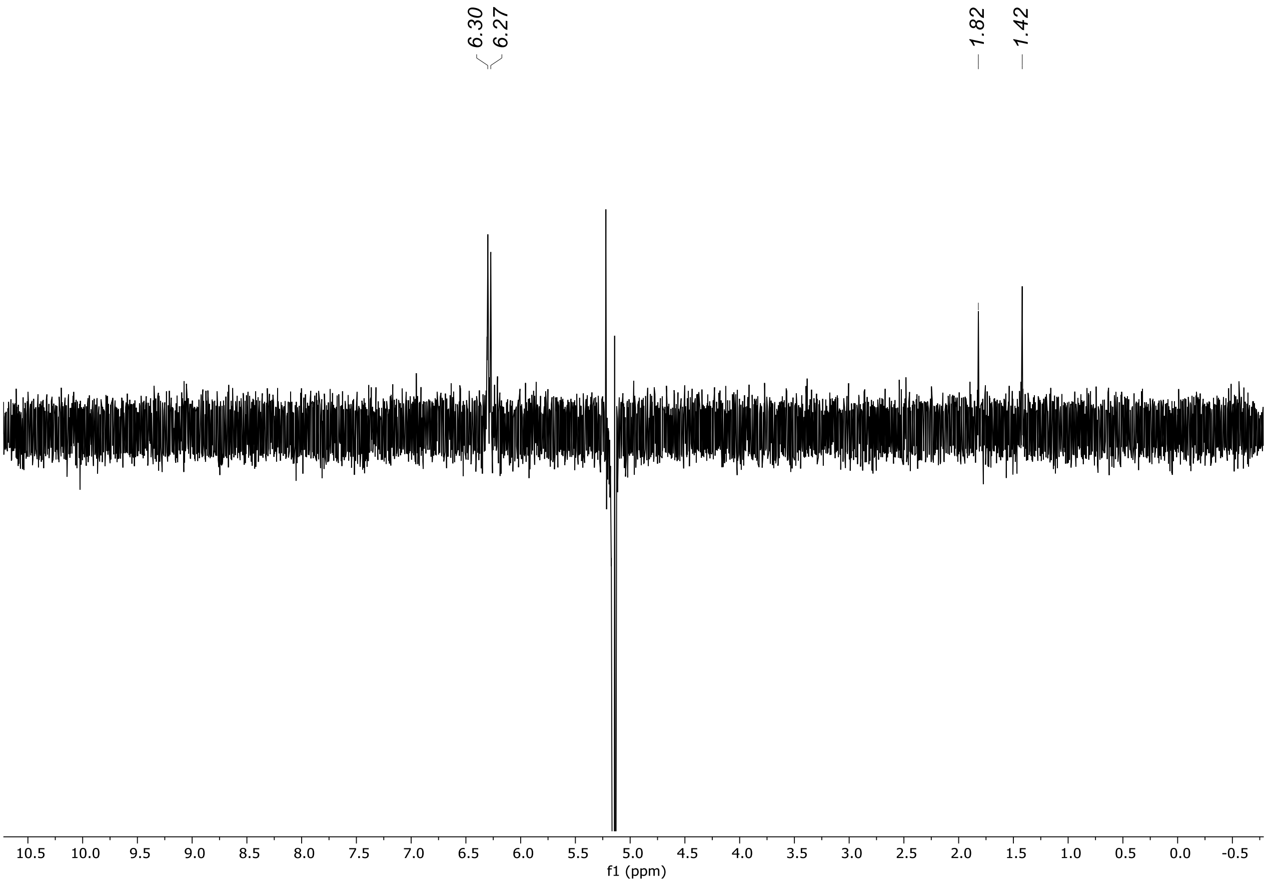

NOE-1D (400.16 MHz, freq. 5.04 ppm, CD_2Cl_2)

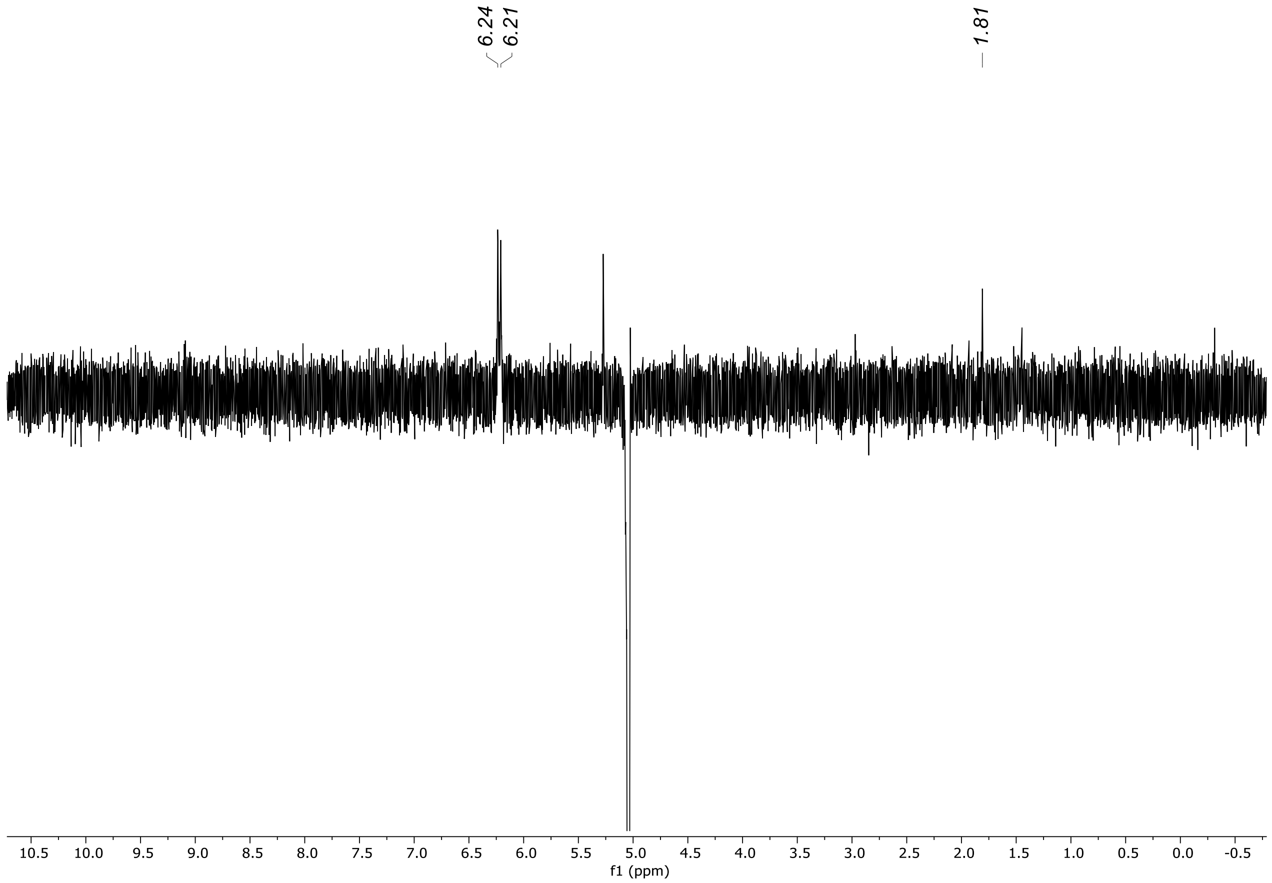

^{1}H-NMR (400.16 MHz, CD_2Cl_2)

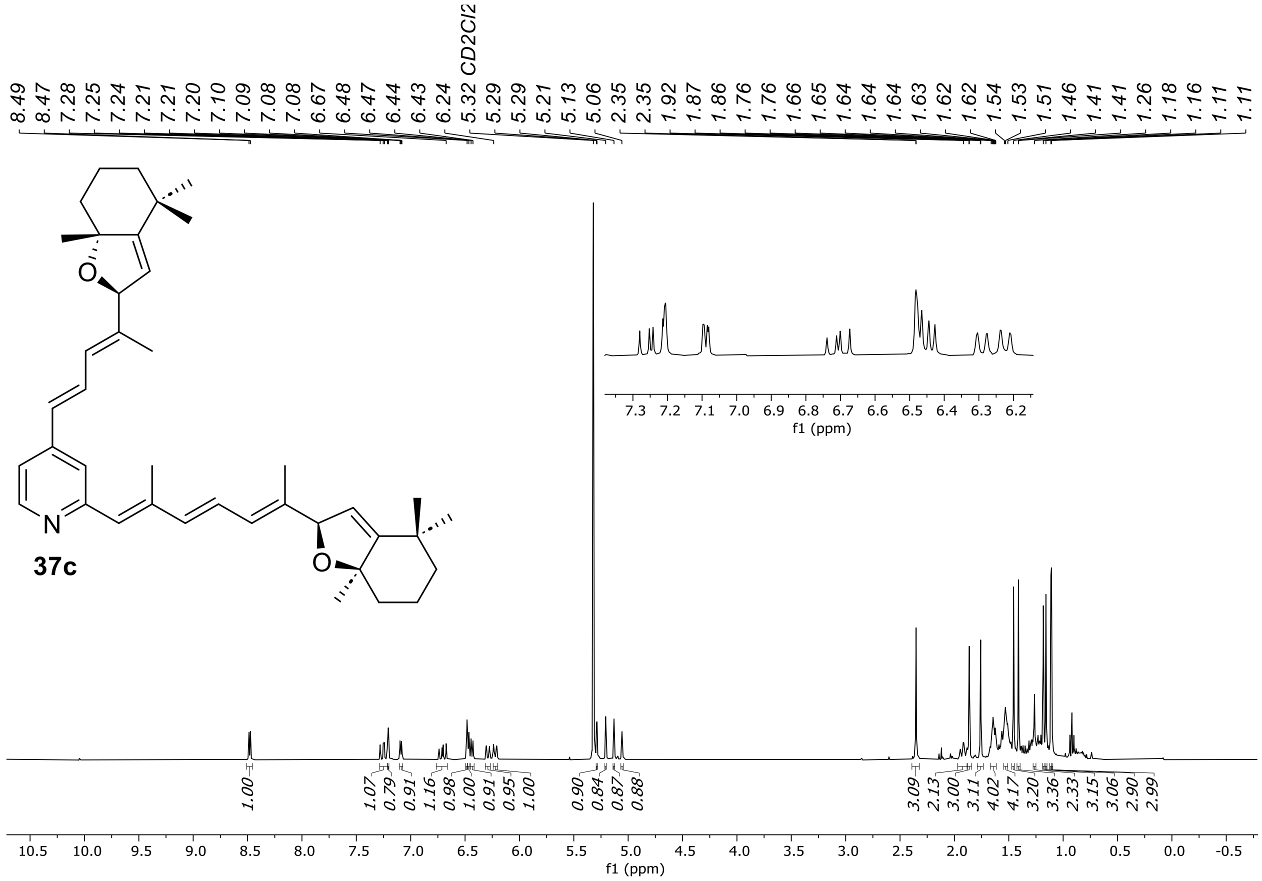

37c

^{13}C{^{1}H}-NMR (100.63 MHz, CD_2Cl_2)

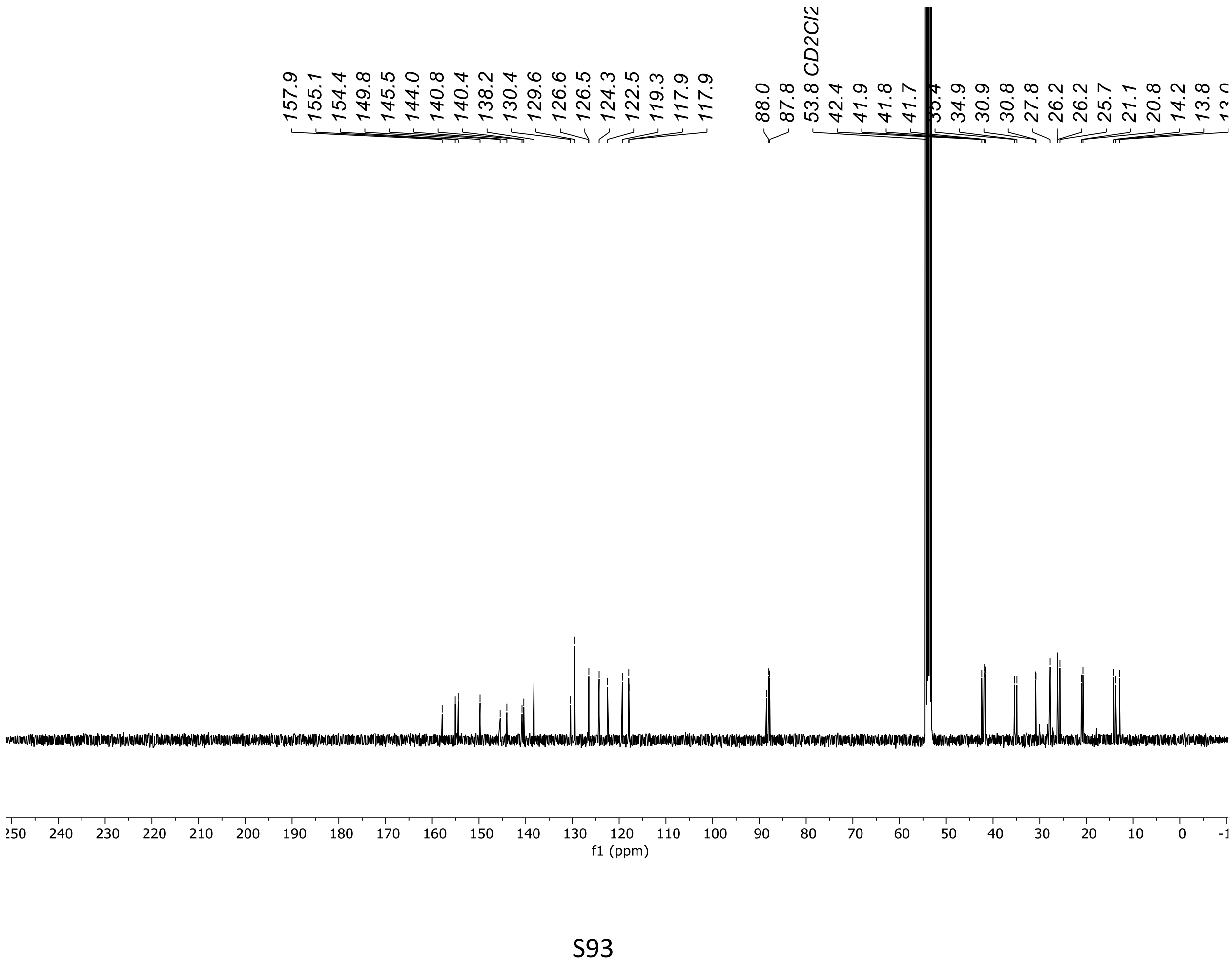

COSY (CD_2Cl_2)

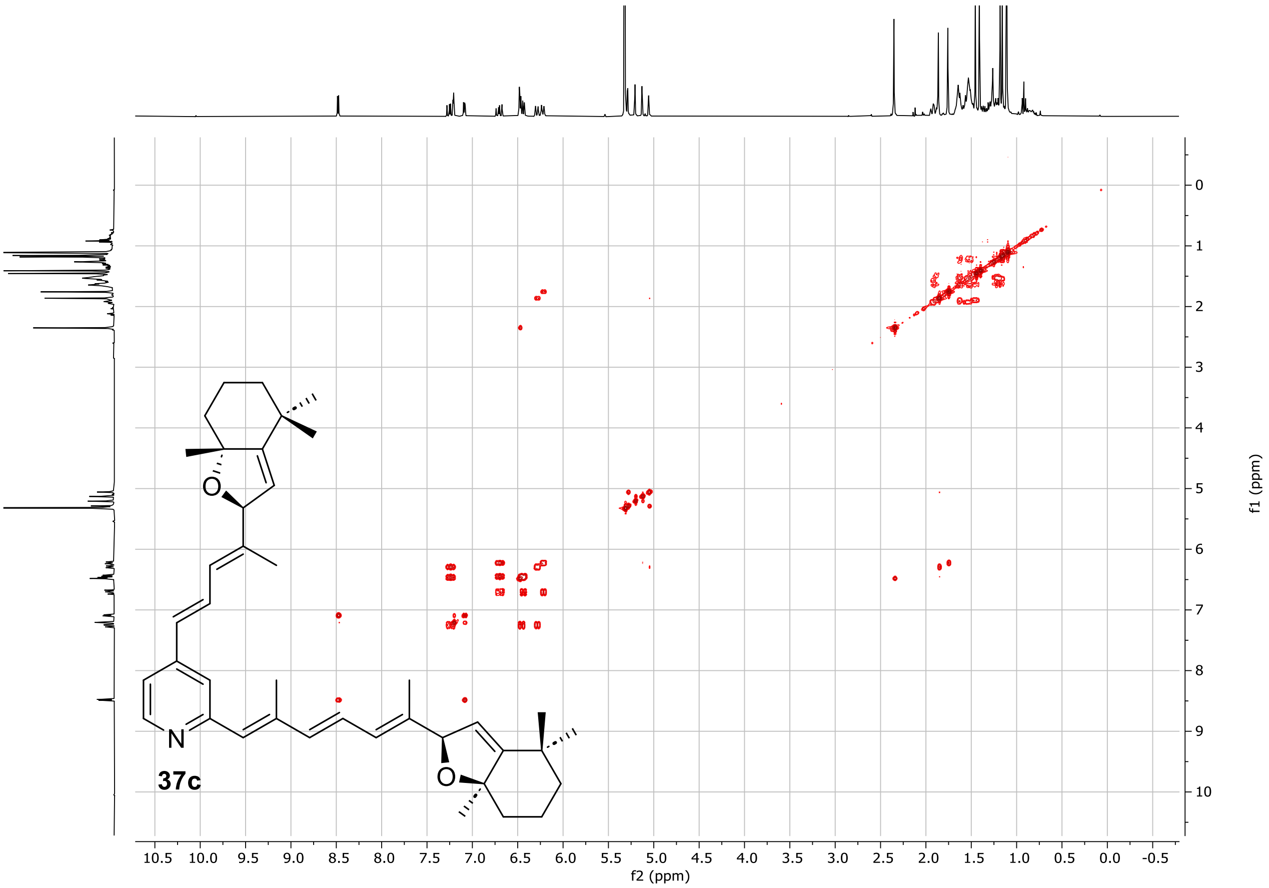

HSQC (CD_2Cl_2)

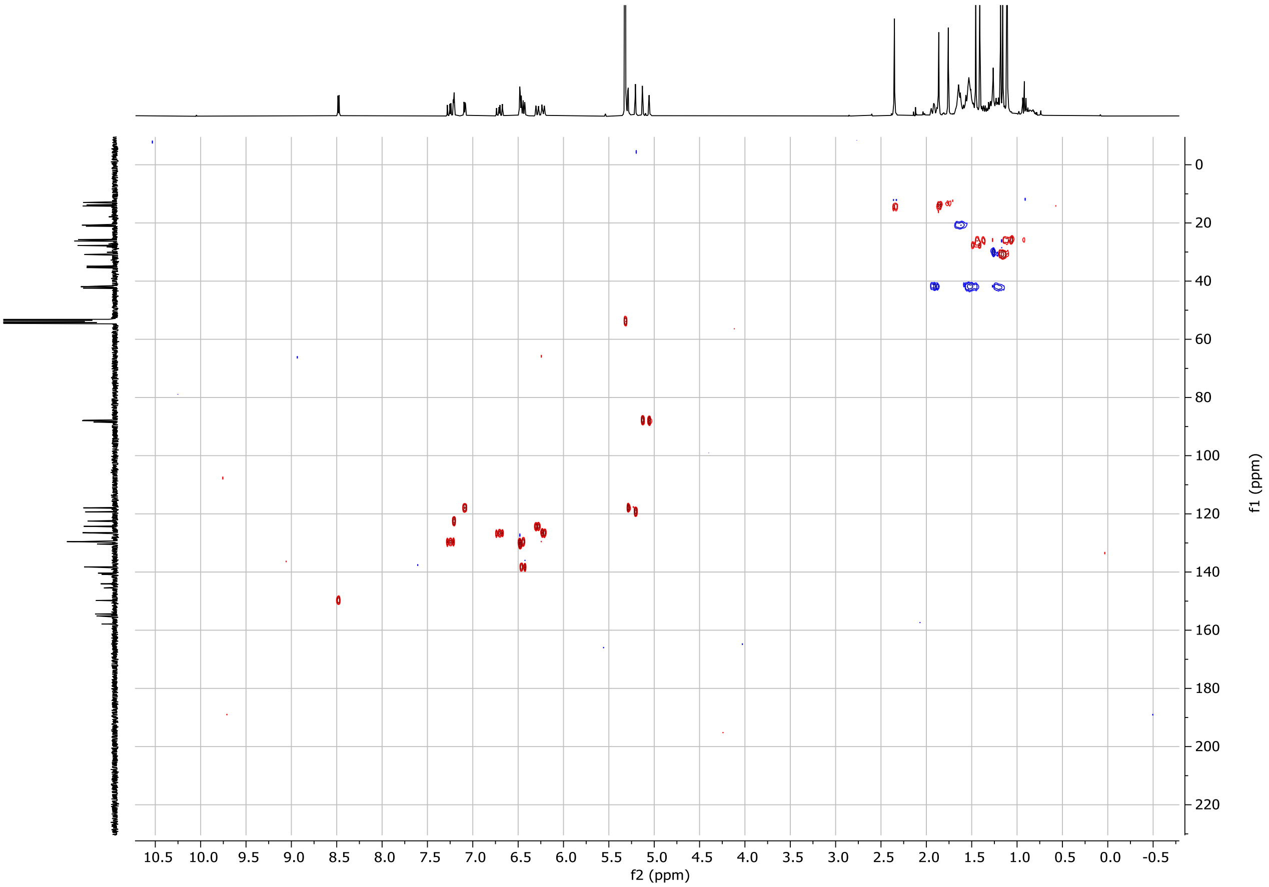

HMBC (CD_2Cl_2)

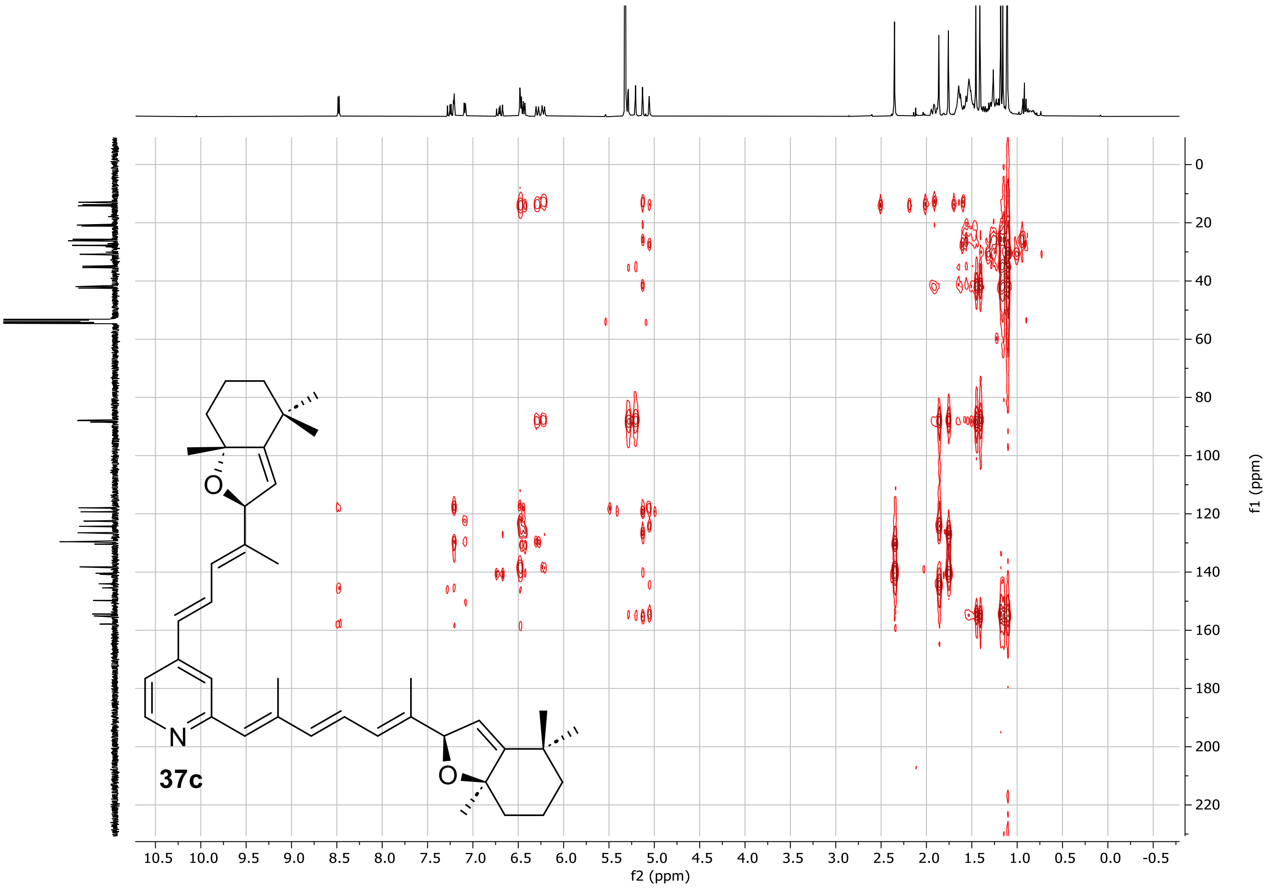

NOE-1D (400.16 MHz, freq. 5.13 ppm, CD_2Cl_2)

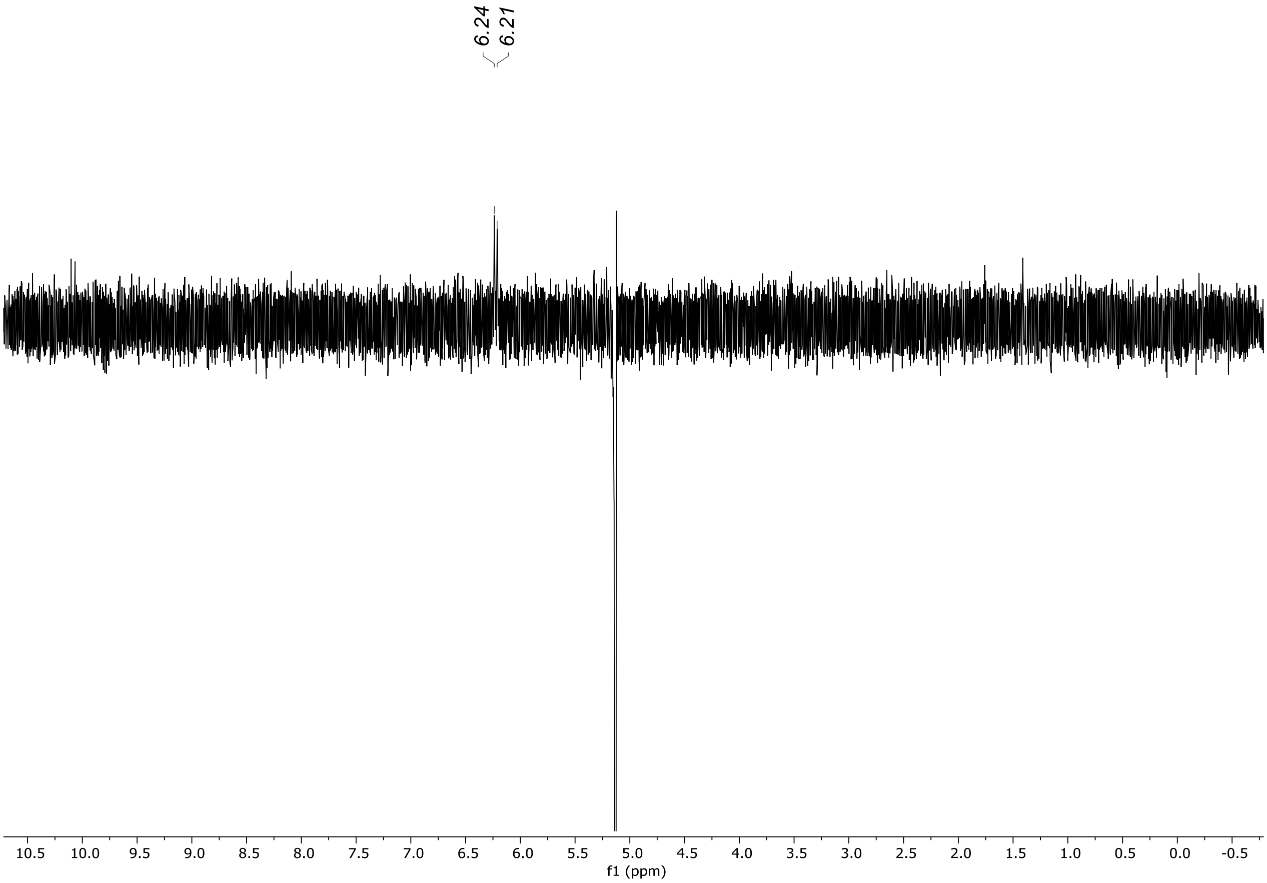

^{1}H-NMR (400.16 MHz, CD_2Cl_2)

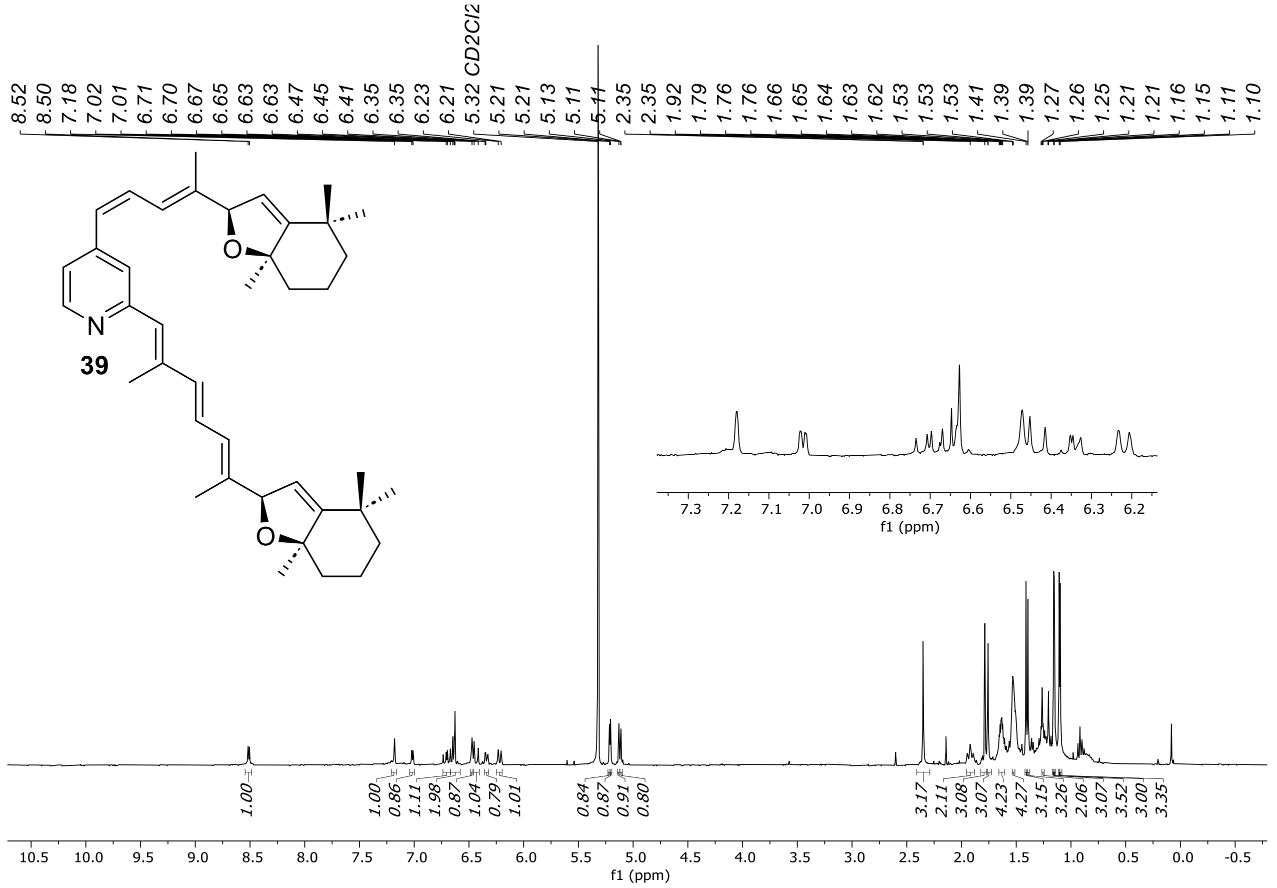

^{13}C{^{1}H}-NMR (100.63 MHz, CD_2Cl_2)

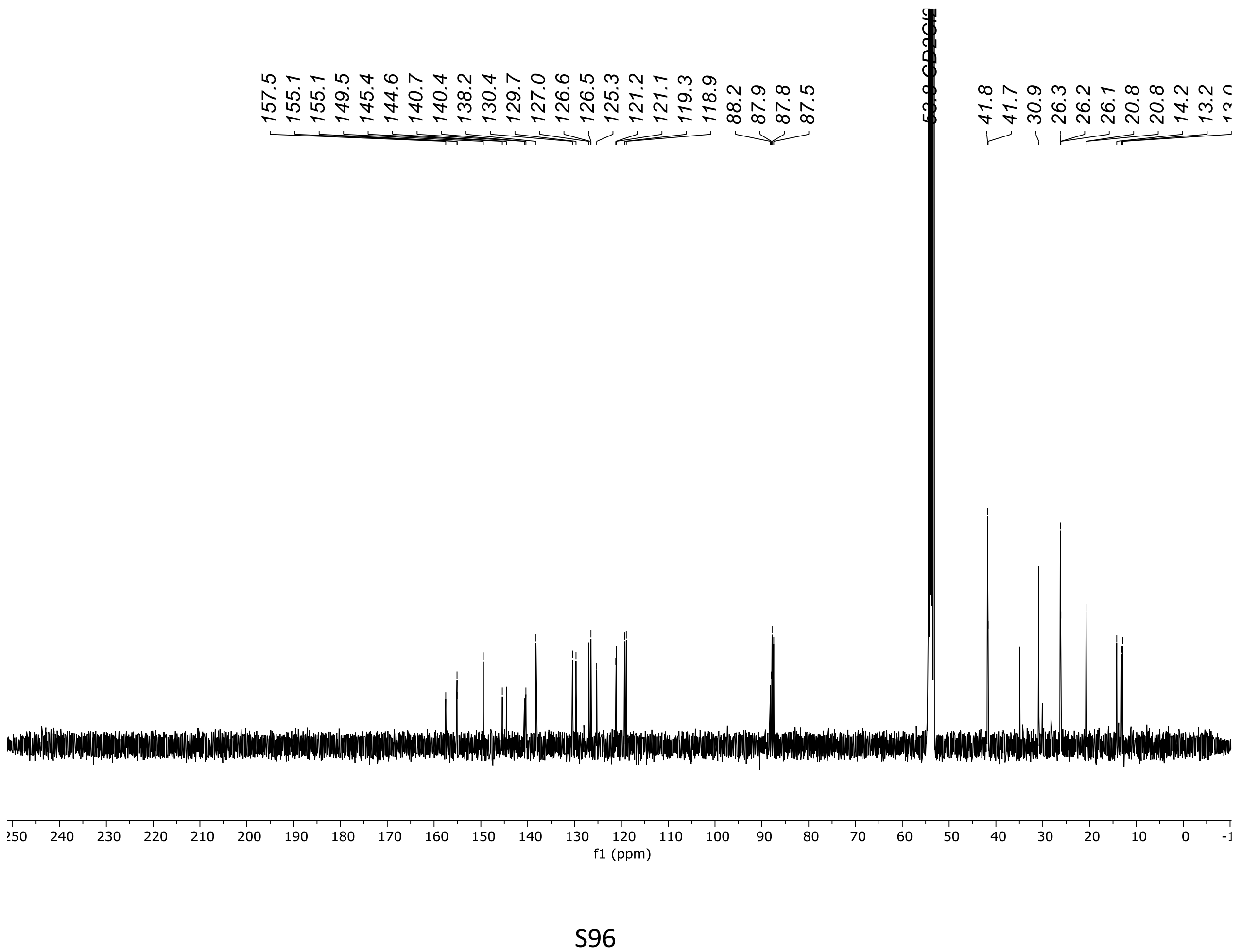

COSY (CD_2Cl_2)

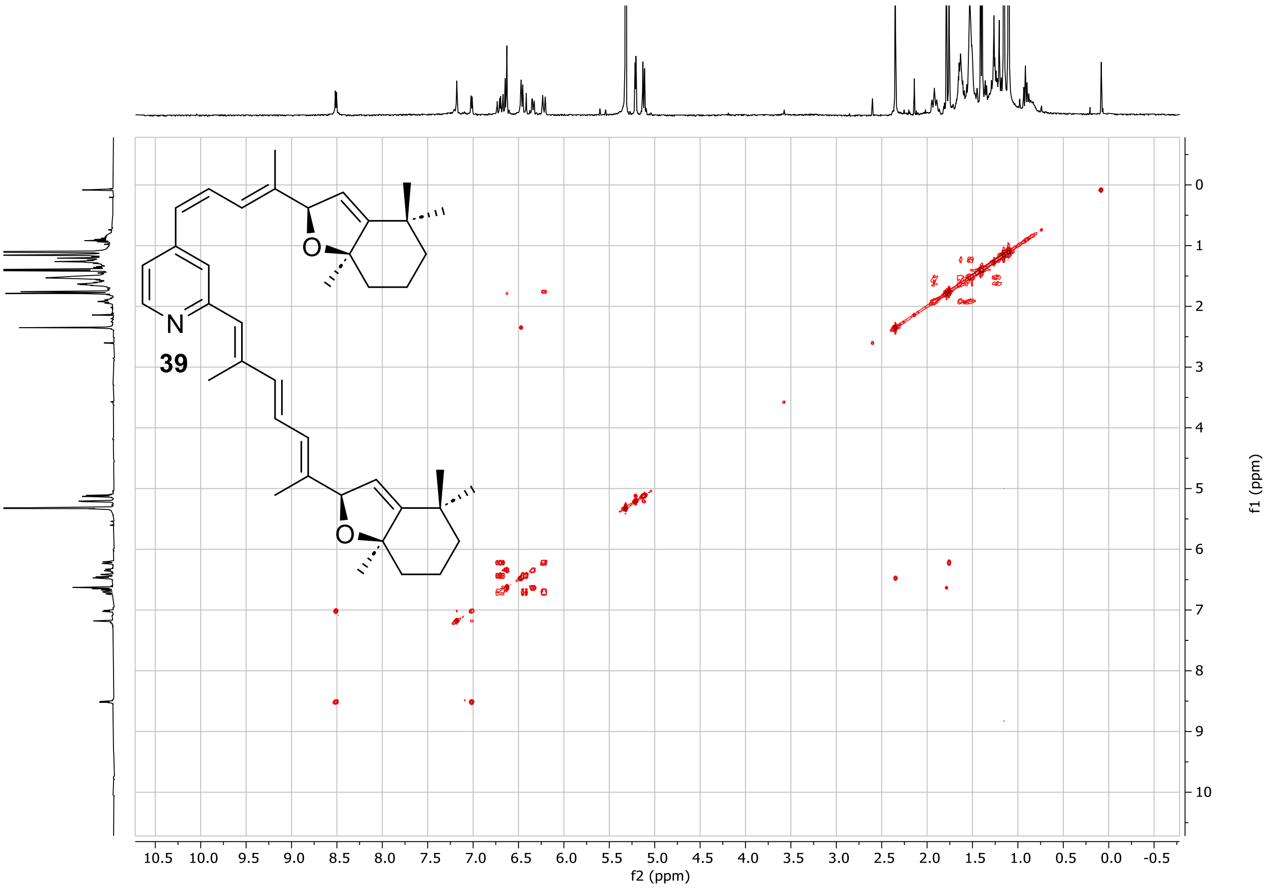

HSQC (CD_2Cl_2)

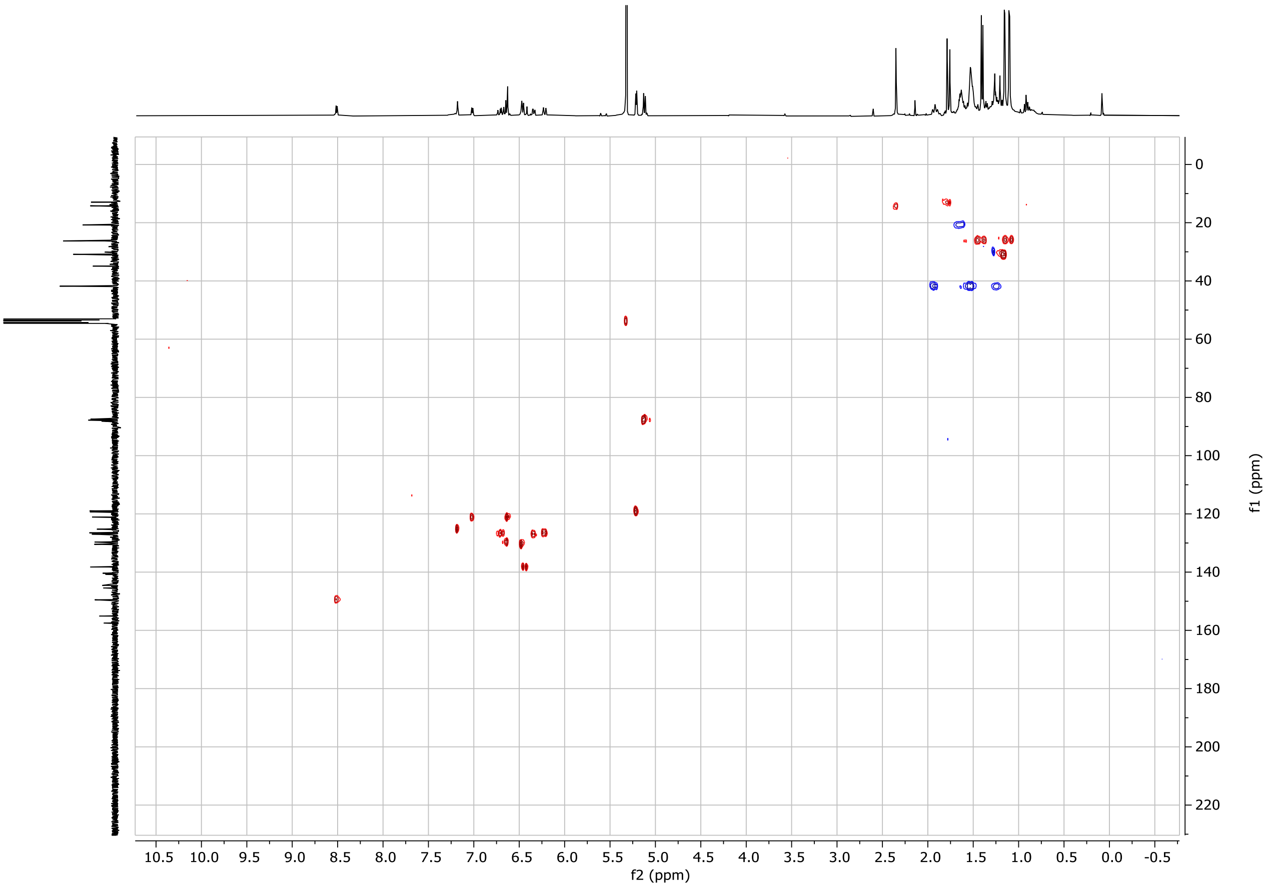

HMBC (CD_2Cl_2)

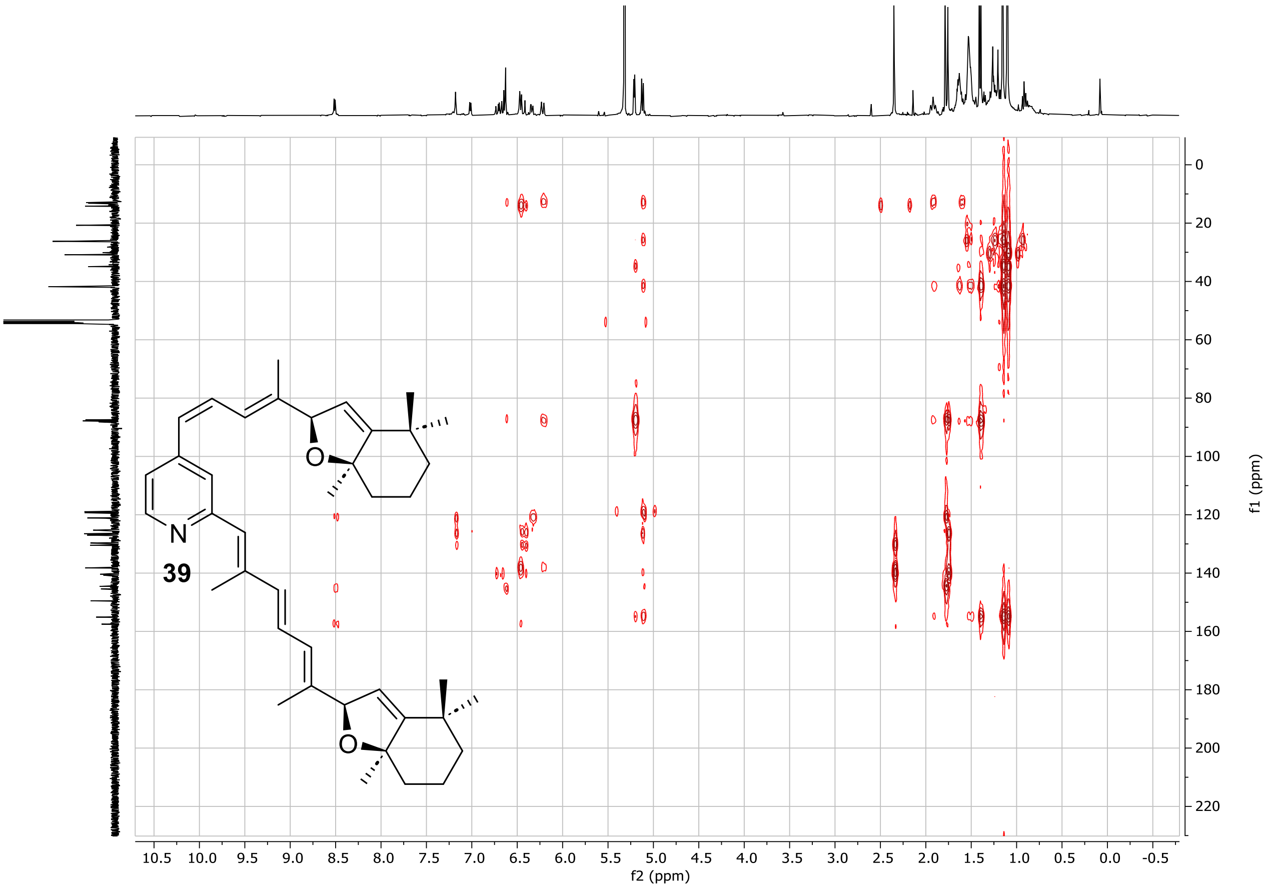

^{1}H-NMR (400.16 MHz, CD_3OD)

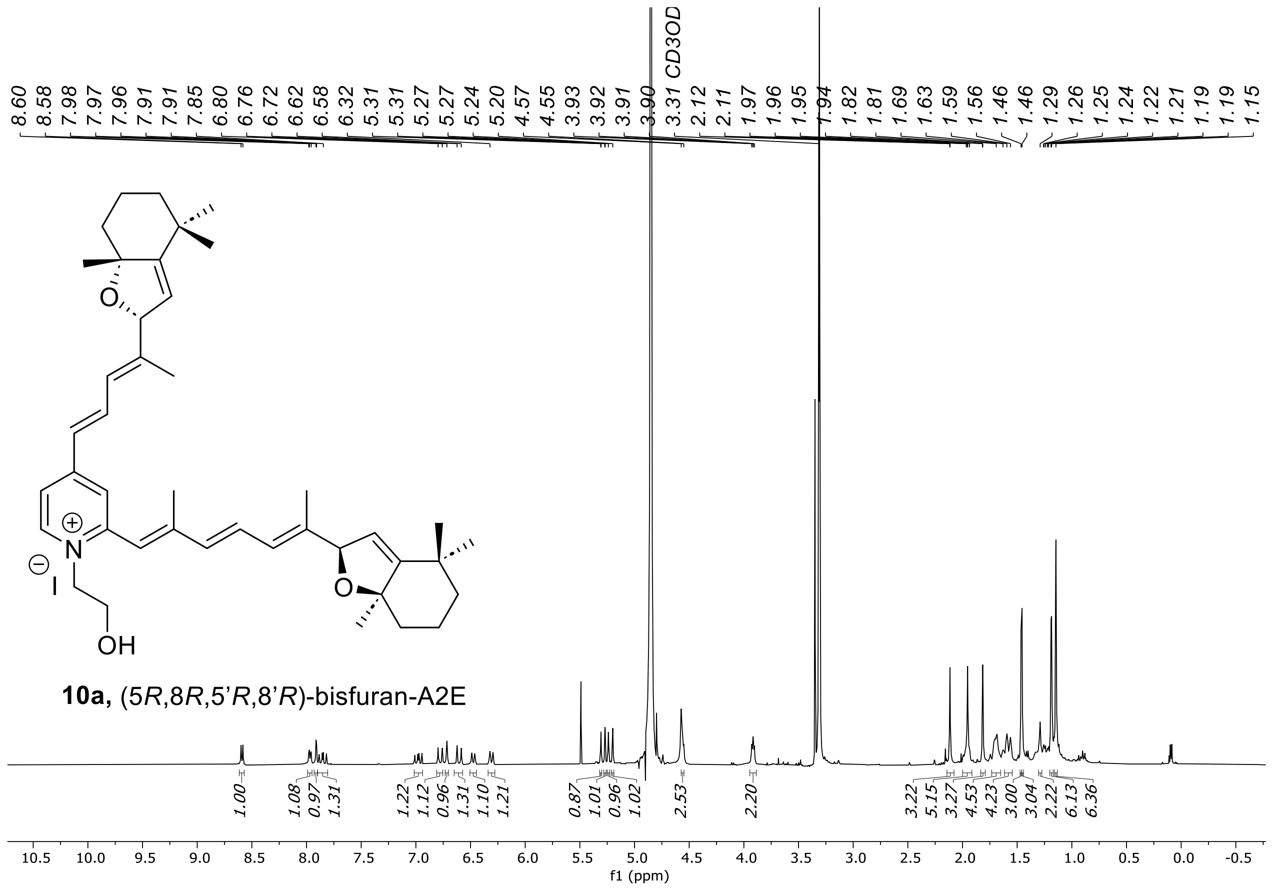

10a, (5*R*,8*R*,5'*R*,8'*R*)-bisfuran-A2E

^{13}C{^{1}H}-NMR (100.63 MHz, C_6D_6)

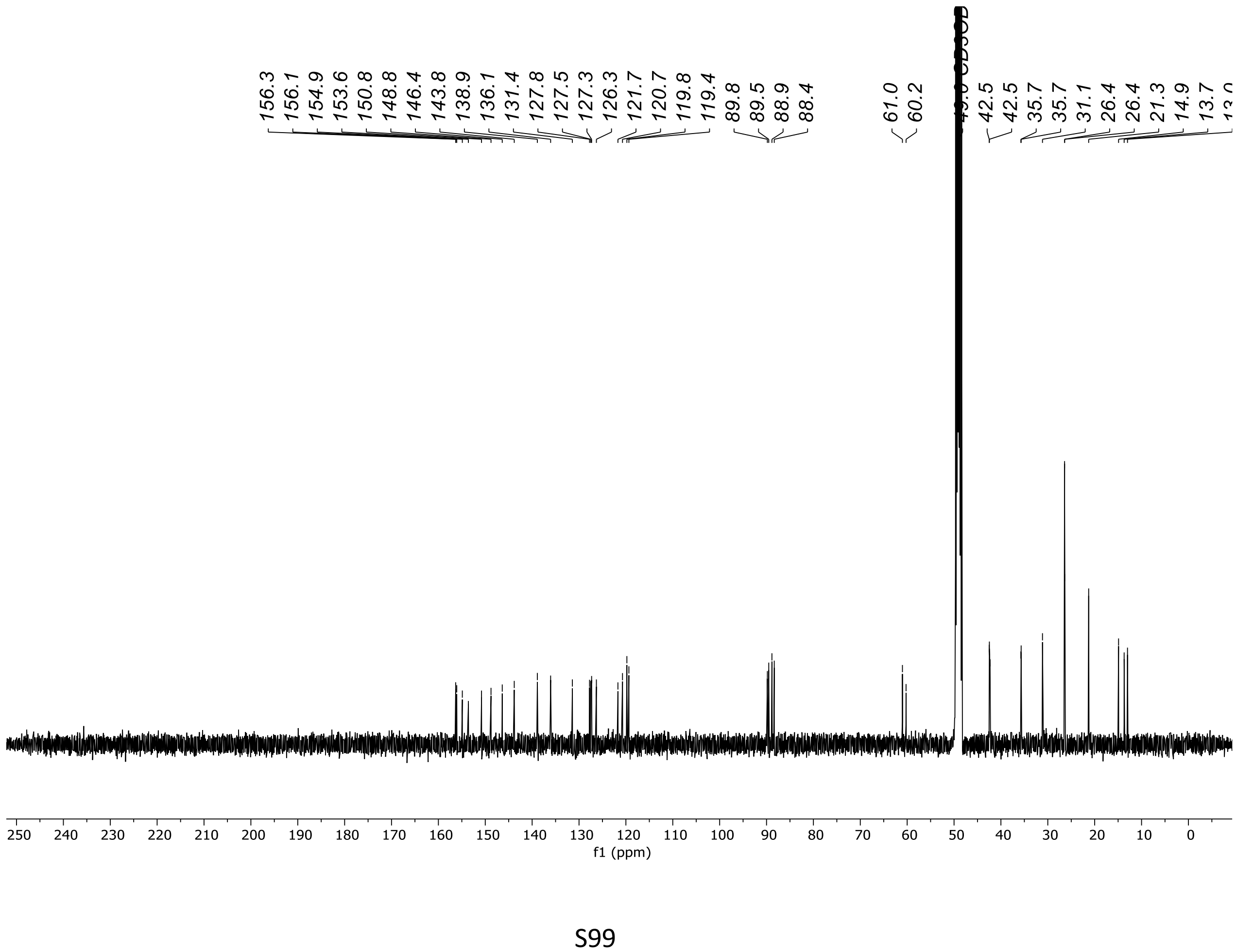

COSY (CD_3OD)

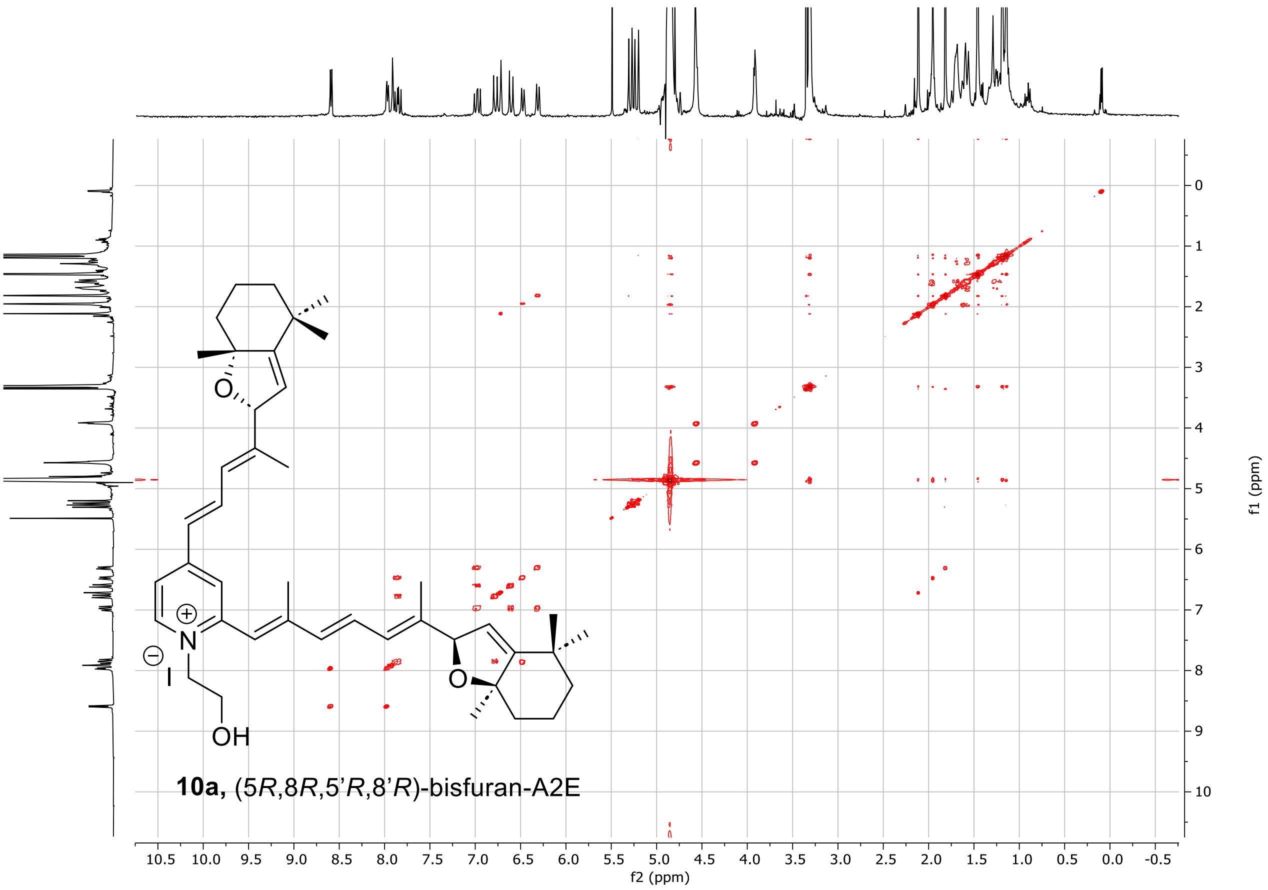

HSQC (CD_3OD)

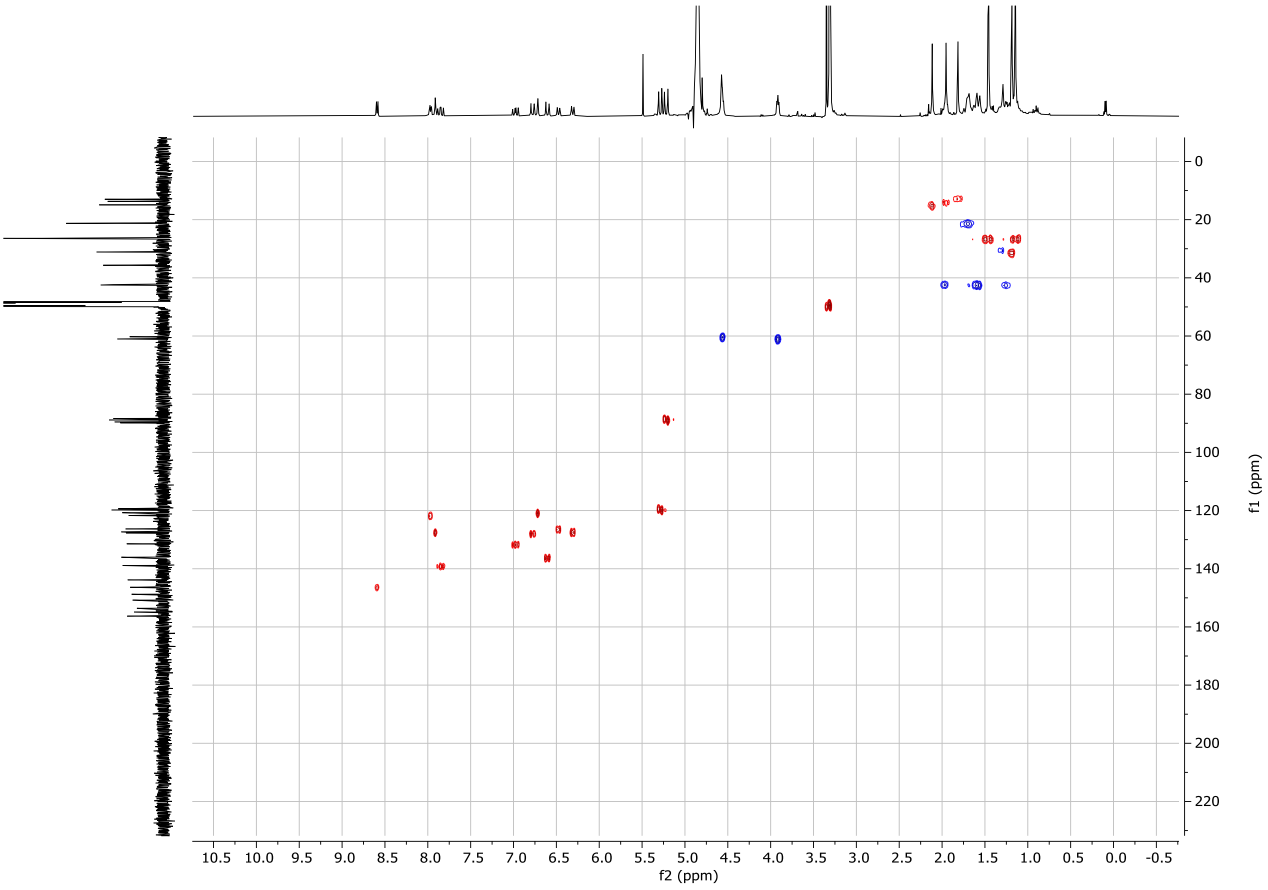

HMBC (CD_3OD)

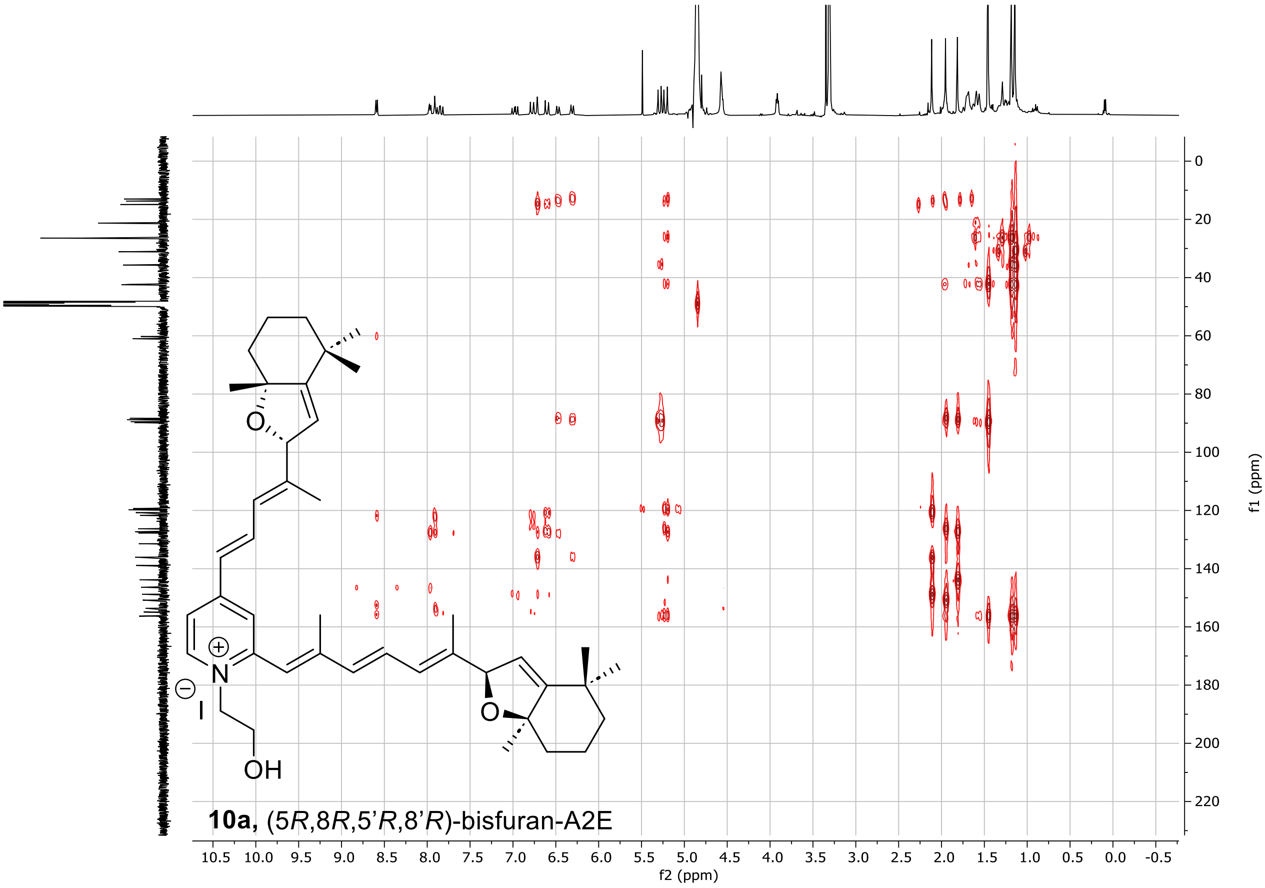

NOE-1D (400.16 MHz, freq. 7.87 ppm, CD_3OD)

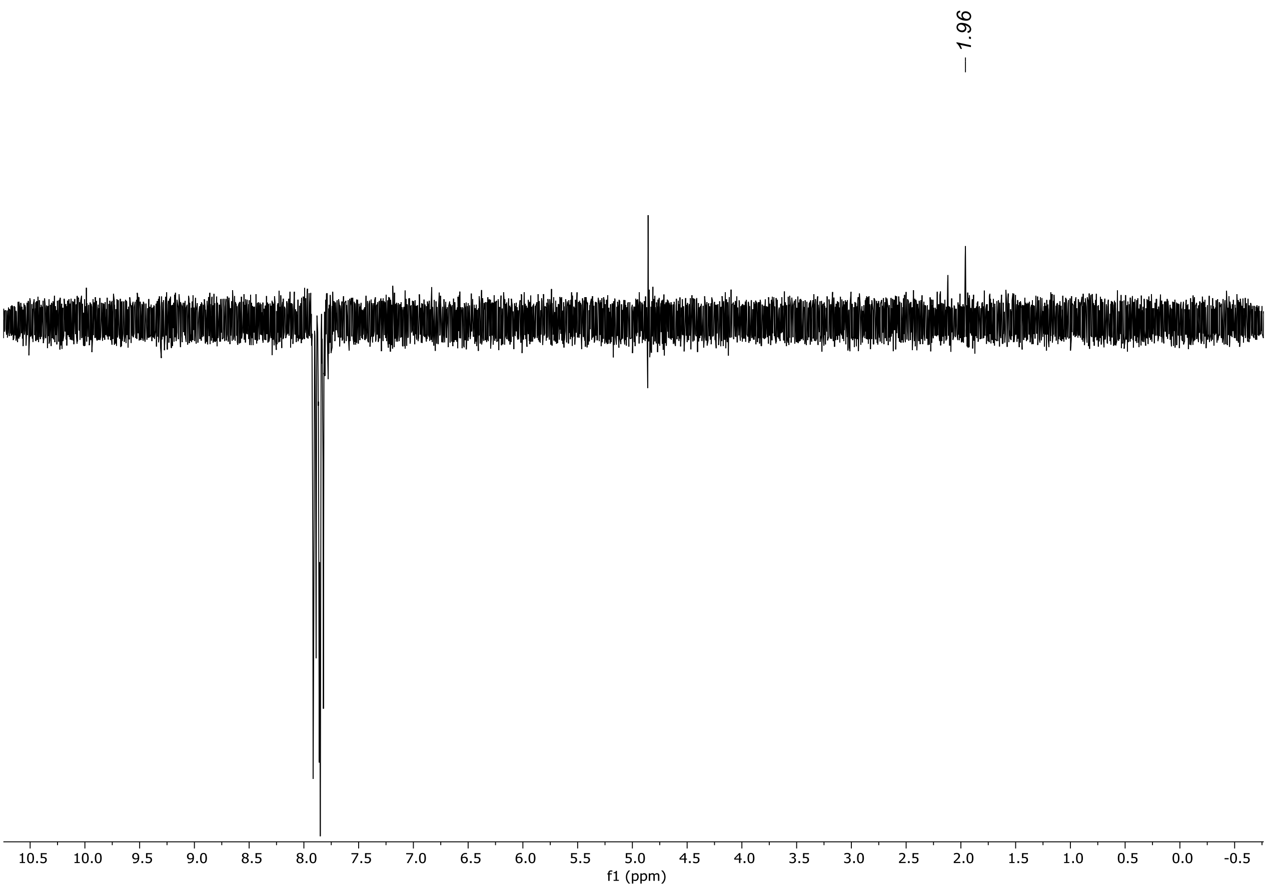

NOE-1D (400.16 MHz, freq. 6.99 ppm, CD_3OD)

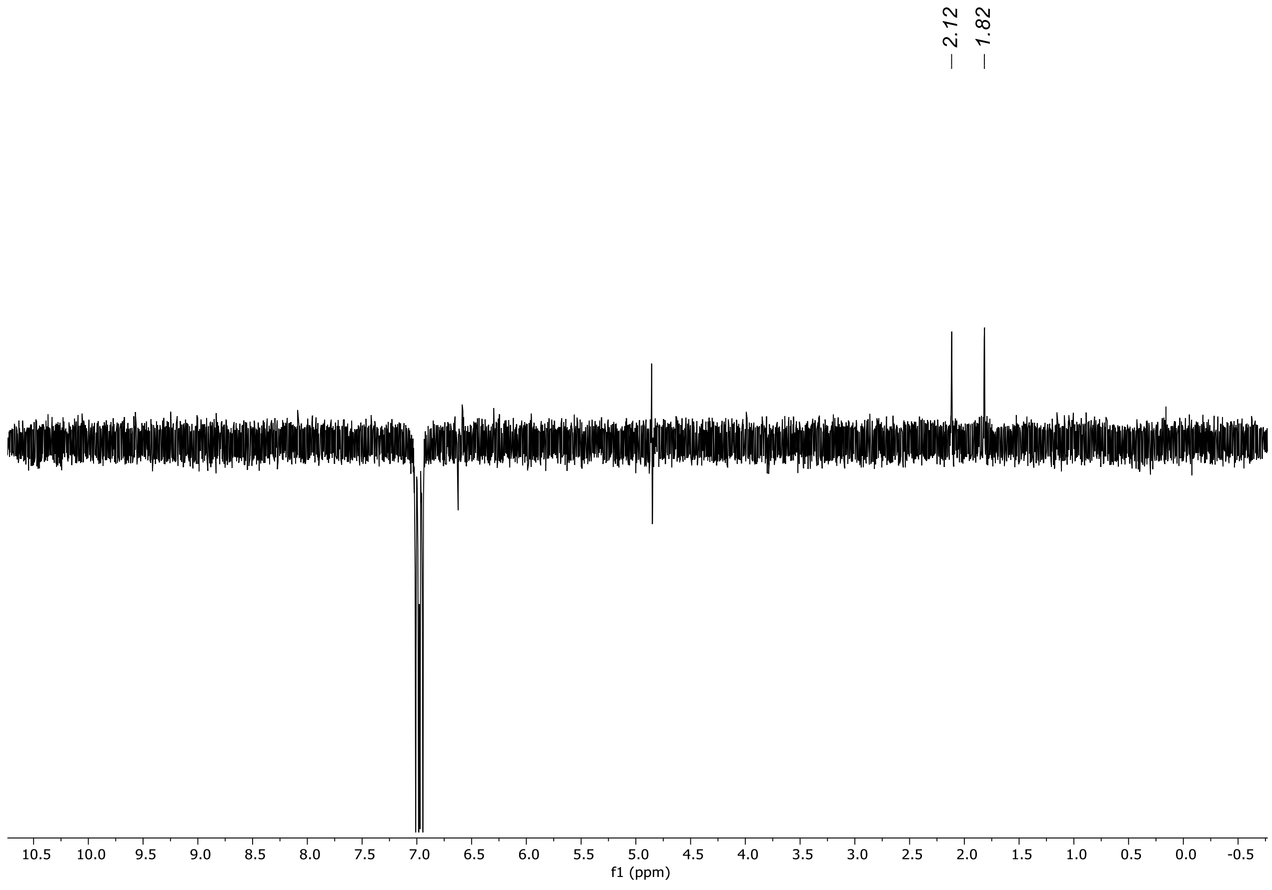

Z-matrix of the pKa simulations

45

Num. Imaginary Frequencies: 0

C	6.022859	1.578585	0.061018
C	5.045260	0.525066	0.008485
C	5.640169	-0.780235	-0.107212
C	7.011450	-0.928885	-0.157951
N	7.921219	0.071477	-0.108033
C	7.371829	1.306362	0.001362
C	3.669500	0.791646	0.067446
C	2.639036	-0.195099	0.023139
C	1.289738	0.038794	0.070384
C	0.269232	-0.997094	0.035166
C	0.703864	-2.437609	-0.050888
C	-1.051656	-0.679732	0.078797
C	-1.715604	0.614968	0.119666
C	-3.047133	0.440925	0.171344
C	-3.347736	-1.043485	0.196221
O	-2.041357	-1.659204	0.050802
C	-4.150066	1.458885	0.034843
C	-5.042659	0.992038	-1.140413
C	-5.453112	-0.480678	-1.061722
C	-4.230220	-1.402222	-1.002250
C	-3.926332	-1.569363	1.510059
C	-4.982377	1.575531	1.322892
C	-3.569991	2.836275	-0.285021
H	-6.096497	-0.650763	-0.187862
H	-5.932939	1.635576	-1.175114
H	-4.484691	1.151547	-2.075246
H	-6.052041	-0.734752	-1.944834
H	-4.530391	-2.456798	-0.940606
H	-3.629522	-1.268326	-1.912800
H	-3.368683	-1.167308	2.364385
H	-4.984098	-1.314615	1.620601
H	-3.839483	-2.662738	1.510908
H	-5.703781	2.395199	1.208457
H	-5.544531	0.664431	1.546330
H	-4.335653	1.803302	2.179701
H	-4.386578	3.552347	-0.442671
H	-2.949303	3.204011	0.542736
H	-2.954791	2.806346	-1.193040
H	-1.190819	1.563791	0.067422
H	0.950664	1.075197	0.143082
H	2.960488	-1.237741	-0.052179
H	3.370531	1.839952	0.150583
H	-0.151841	-3.117195	-0.069596
H	1.302035	-2.610576	-0.956639
H	1.339047	-2.705696	0.805151
H	5.693530	2.614845	0.148786
H	8.079255	2.137912	0.043989
H	7.423458	-1.936994	-0.246407
H	5.022655	-1.675695	-0.157511

44

Num. Imaginary Frequencies: 0

C	6.304483	0.294953	-1.234224
C	5.248549	0.112403	-0.331117
C	5.593806	-0.009458	1.024389
C	6.931142	0.054547	1.392494
N	7.945019	0.228610	0.528819
C	7.612766	0.345050	-0.763461
C	3.871198	0.058922	-0.835880
C	2.764305	-0.125732	-0.088899
C	1.436390	-0.168184	-0.682662
C	0.271956	-0.358988	-0.029456
C	0.125956	-0.596554	1.443386
C	-1.017778	-0.306141	-0.825239
C	-1.906602	0.858539	-0.477775
C	-3.094176	0.437963	-0.041350
C	-3.132791	-1.078193	-0.041459
O	-1.835364	-1.456988	-0.564400
C	-4.343126	1.243734	0.224073
C	-5.437209	0.675787	-0.711318
C	-5.567163	-0.848940	-0.664113
C	-4.239289	-1.538457	-0.996929
C	-3.273354	-1.737654	1.331600
C	-4.783478	1.142826	1.693126
C	-4.112808	2.717906	-0.105910
H	-5.922153	-1.169260	0.324694
H	-0.763886	-0.287726	-1.896020
H	-6.394589	1.149354	-0.452470
H	-5.190083	0.975300	-1.740934
H	-6.329321	-1.166685	-1.386255
H	-4.339307	-2.631430	-0.949121
H	-3.934261	-1.266552	-2.017397
H	-2.665449	-1.224296	2.084650
H	-4.313433	-1.758612	1.669554
H	-2.928812	-2.775676	1.245078
H	-5.636418	1.814030	1.857507
H	-5.092920	0.132183	1.973188
H	-3.968891	1.452841	2.360362
H	-5.045979	3.277509	0.037393
H	-3.348833	3.153713	0.551497
H	-3.788560	2.845796	-1.146218
H	-1.590882	1.887213	-0.632934
H	1.398476	-0.028621	-1.766381
H	2.851992	-0.249574	0.991034
H	3.763920	0.179833	-1.916413
H	-0.598595	0.110653	1.869634
H	-0.268663	-1.605564	1.621942
H	1.070848	-0.496021	1.982293
H	6.107151	0.396884	-2.300508
H	8.435198	0.486254	-1.465061
H	7.205031	-0.039244	2.443406
H	4.839545	-0.152645	1.795294

48

Num. Imaginary Frequencies: 0

C	7.134125	-0.569394	-0.025245
C	6.295988	0.596282	0.001576
N	6.908317	1.835337	0.028298
C	8.238037	1.912919	0.021947
C	9.106838	0.824542	-0.009434
C	8.509193	-0.445740	-0.031133
C	4.875859	0.626336	0.011740
C	3.892436	-0.399169	-0.033925
C	4.290638	-1.853902	-0.127130
C	2.544371	-0.049895	0.005793

C	1.403072	-0.916972	-0.019964
C	0.104301	-0.488986	0.025115
C	-1.057201	-1.362973	0.006514
C	-0.841379	-2.851978	-0.071517
C	-2.314525	-0.848442	0.056159
C	-2.773632	0.532406	0.105848
C	-4.115159	0.560188	0.174019
C	-4.636335	-0.861335	0.194705
O	-3.437850	-1.665869	0.035596
C	-5.052218	1.734121	0.056073
C	-6.013405	1.417187	-1.115144
C	-6.640957	0.022842	-1.043191
C	-5.571288	-1.073694	-0.999176
C	-5.276291	-1.304845	1.509926
C	-5.846477	1.962371	1.353247
C	-4.273299	3.010947	-0.258376
H	-7.297380	-0.054256	-0.165914
H	-6.796302	2.188115	-1.137822
H	-5.443929	1.498444	-2.053081
H	-7.276762	-0.131252	-1.923576
H	-6.027132	-2.071046	-0.941561
H	-4.963429	-1.025499	-1.913516
H	-4.656693	-0.999268	2.361481
H	-6.282391	-0.894293	1.632502
H	-5.355588	-2.398691	1.501474
H	-6.435682	2.883257	1.252716
H	-6.538774	1.145218	1.574762
H	-5.165316	2.080891	2.205394
H	-4.973969	3.842949	-0.403924
H	-3.598353	3.273680	0.566828
H	-3.676516	2.896391	-1.171910
H	-2.112873	1.391751	0.051693
H	-0.076995	0.587165	0.082338
H	1.571503	-1.993993	-0.074978
H	2.322369	1.020500	0.069345
H	-0.246655	-3.204820	0.782809
H	-1.788632	-3.397058	-0.079159
H	-0.281838	-3.117573	-0.979516
H	4.473893	1.640518	0.060391
H	6.702841	-1.562619	-0.036006
H	8.649261	2.925576	0.043918
H	9.126520	-1.344905	-0.050379
H	3.422767	-2.512307	-0.200837
H	4.920433	-2.029980	-1.008648
H	4.868966	-2.159007	0.754886
H	10.185080	0.964809	-0.013116

47

Num. Imaginary Frequencies: 0

C	9.250353	-0.050815	0.180294
C	8.692538	-0.666247	-0.938665
N	7.386500	-0.653754	-1.220726
C	6.555470	-0.022841	-0.364884
C	7.035309	0.650078	0.769054
C	8.398326	0.631694	1.044952
C	5.132090	-0.033883	-0.741431
C	4.054196	-0.101735	0.075465
C	4.119230	-0.270063	1.569274
C	2.731181	-0.035149	-0.550344

C	1.554117	-0.019468	0.111192
C	0.276812	0.067267	-0.583080
C	-0.941190	0.092219	-0.005515
C	-1.198450	0.043704	1.473036
C	-2.170196	0.134836	-0.887565
O	-2.904083	1.348423	-0.642523
C	-4.318444	1.071357	-0.502650
C	-4.371252	-0.437023	-0.339499
C	-3.165507	-0.953044	-0.578481
C	-5.622720	-1.129430	0.145482
C	-6.126884	-0.341990	1.376616
C	-6.217725	1.169034	1.150926
C	-4.858147	1.753809	0.755503
C	-5.008777	1.587914	-1.766123
C	-6.704674	-1.167708	-0.946459
C	-5.310441	-2.567576	0.558502
H	-6.966858	1.395246	0.380089
H	-1.850950	0.109015	-1.941769
H	-7.106315	-0.746088	1.668518
H	-5.432372	-0.530869	2.209426
H	-6.563013	1.651416	2.073539
H	-4.926215	2.837404	0.589833
H	-4.141115	1.577450	1.569025
H	-4.710512	0.993194	-2.638249
H	-6.098933	1.566258	-1.677136
H	-4.702817	2.629227	-1.925792
H	-7.554587	-1.762641	-0.587195
H	-7.078487	-0.173293	-1.206585
H	-6.314251	-1.640022	-1.856862
H	-6.212725	-3.037041	0.970989
H	-4.980796	-3.162644	-0.303100
H	-4.522887	-2.597216	1.322270
H	-2.879523	-2.001202	-0.532919
H	0.327650	0.106462	-1.674668
H	1.545502	-0.071432	1.200140
H	2.719504	0.025919	-1.641980
H	-0.320845	0.325198	2.060966
H	-2.020970	0.721787	1.733504
H	-1.506246	-0.967728	1.774734
H	4.952133	-0.006631	-1.818147
H	6.349668	1.201625	1.407800
H	9.333070	-1.190996	-1.648111
H	8.790037	1.152194	1.917920
H	3.392536	-1.027080	1.887764
H	5.109899	-0.590044	1.901427
H	3.862486	0.666064	2.084289
H	10.323814	-0.094276	0.351951

18-A$^-$

Num. Imaginary Frequencies: 0

C	5.579946	-0.157210	-0.358144
C	5.609713	1.280685	0.134056
C	4.393477	1.998242	-0.428427
C	3.020001	1.416969	-0.019715
C	3.026757	-0.138140	-0.101111
C	4.334639	-0.933808	0.104210
H	5.617500	1.317783	1.232709
C	2.754107	1.780859	1.462877
O	2.050034	2.006132	-0.834138

C	1.882967	-0.858944	-0.223969
C	0.514439	-0.373743	-0.184086
C	-0.602504	-1.139335	-0.327968
C	-0.623844	-2.612253	-0.624888
C	4.478286	-1.264684	1.604233
C	4.338475	-2.269083	-0.657711
C	-1.884247	-0.464649	-0.180536
C	-3.090108	-1.064061	-0.279630
P	-4.596583	-0.152553	-0.096304
O	-4.958264	-0.256125	1.483496
C	-6.234148	0.279231	1.895840
O	-4.238503	1.431633	-0.207019
C	-4.072261	1.964908	-1.536137
O	-5.724393	-0.576481	-1.014165
H	-3.349258	1.367758	-2.105457
H	-6.287723	1.349928	1.668620
H	0.368775	0.685555	0.012513
H	6.466294	-0.714124	-0.023583
H	5.601094	-0.148958	-1.459192
H	6.530656	1.772887	-0.206022
H	4.383482	3.061227	-0.147150
H	4.442626	1.954086	-1.527470
H	1.786077	1.372565	1.780419
H	3.527743	1.410912	2.147287
H	2.716824	2.876083	1.544890
H	5.368406	-1.891831	1.751826
H	4.590043	-0.368961	2.223730
H	3.600762	-1.820581	1.956667
H	5.348352	-2.697473	-0.610285
H	3.653912	-3.006862	-0.224150
H	4.075511	-2.124362	-1.713295
H	1.981553	-1.940641	-0.281765
H	-1.841328	0.608560	0.021966
H	-3.205809	-2.127175	-0.487210
H	-1.190045	-2.804617	-1.546702
H	0.377673	-3.029487	-0.748617
H	-1.130716	-3.159171	0.182511
H	-3.692231	2.981578	-1.412689
H	-5.035849	1.984650	-2.055620
H	-7.047726	-0.257526	1.396415
H	-6.293016	0.125309	2.975456

18

Num. Imaginary Frequencies: 0

C	-3.133591	1.506386	-0.027018
C	-4.547811	2.035996	-0.189504
C	-5.605258	0.961299	-0.440366
C	-5.347141	-0.274978	0.411642
C	-4.020402	-0.968223	0.062494
C	-2.866039	0.044684	0.100757
C	-4.101172	-1.597118	-1.335097
C	-3.751188	-2.062771	1.098702
O	-2.867113	0.892754	1.258775
C	-1.535847	-0.505917	-0.286727
C	-0.464909	-0.511916	0.517978
C	0.845452	-1.074971	0.161628
C	1.838450	-0.987889	1.068021
C	3.236697	-1.504511	0.897032
P	4.350425	-0.263773	0.220541
O	4.193475	0.959021	1.280104
C	4.842012	2.200244	0.934091
H	4.717702	2.862353	1.793449
C	-2.061648	2.459420	-0.488024
C	0.990772	-1.703922	-1.195250
O	5.783068	-0.681195	-0.028736
O	3.612565	0.275515	-1.126288
C	4.421494	0.612551	-2.271826
H	3.727207	0.992602	-3.024858
H	-6.599038	1.370312	-0.221665
H	-5.603173	0.682685	-1.502233
H	-6.152472	-1.009056	0.276879
H	-5.338795	0.005559	1.474326
H	-4.547283	2.766634	-1.007188
H	-4.779477	2.591971	0.730745
H	-5.020127	-2.191794	-1.415372
H	-4.111288	-0.837850	-2.126956
H	-3.253304	-2.267617	-1.517379
H	-2.848446	-2.635017	0.852249
H	-3.629074	-1.630485	2.100657
H	-4.600047	-2.758622	1.127707
H	-2.226910	3.439919	-0.021720
H	-1.061784	2.104752	-0.225164
H	-2.123926	2.589071	-1.575627
H	-1.478456	-0.938895	-1.285212
H	-0.546265	-0.071545	1.514155
H	1.631134	-0.489052	2.015627
H	0.813328	-0.953727	-1.978099
H	1.983538	-2.131658	-1.354325
H	0.241759	-2.495248	-1.330208
H	3.305599	-2.362739	0.219103
H	5.153092	1.386599	-2.016513
H	4.932566	-0.278540	-2.649993
H	5.908600	2.034341	0.744759
H	4.359886	2.639566	0.053128
H	3.673887	-1.796855	1.859561

Z-matrix of the DFT-study on the epoxide-furanoxide rearrangement under basic conditions.

8S-III

Num. Imaginary Frequencies: 0

C	-5.465345	0.204618	-1.277314
C	-5.406060	-1.319761	-1.157984
C	-3.964586	-1.832768	-1.119641
C	-3.165719	-1.191720	0.027696
C	-3.330310	0.320861	-0.063385
C	-4.697410	0.965364	-0.164974
H	-5.950171	-1.651367	-0.257675
C	-3.546248	-1.811167	1.378960
O	-1.773877	-1.404775	-0.155099
C	-2.126502	0.871746	-0.215986
C	-1.035338	-0.166347	-0.230584
C	0.014557	-0.038385	0.835542
C	-0.435410	0.041011	2.270232
C	-5.464214	0.891734	1.168698
C	-4.576913	2.440427	-0.561228
C	1.337566	-0.054661	0.495957
C	2.489671	0.035230	1.331122
P	4.078086	0.005597	0.723221
O	4.360662	-1.442459	-0.009102
C	5.603647	-1.681610	-0.648703
C	5.459355	-1.668705	-2.159230
O	4.150895	0.905887	-0.670086
C	4.029261	2.312123	-0.563091
C	2.775582	2.807763	-1.258528
O	5.194263	0.362911	1.663213
H	-0.519917	-0.139245	-1.211456
H	-6.514005	0.546995	-1.289140
H	-5.028091	0.494148	-2.248844
H	-5.937327	-1.771098	-2.010597
H	-3.937913	-2.931359	-1.032111
H	-3.454697	-1.567462	-2.060519
H	-3.124315	-1.225748	2.207532
H	-4.631558	-1.894547	1.524619
H	-3.119662	-2.824422	1.421018
H	-6.416938	1.438265	1.083691
H	-5.700293	-0.136785	1.469100
H	-4.877254	1.350779	1.978844
H	-5.577103	2.882054	-0.693956
H	-4.053033	3.020069	0.214646
H	-4.024647	2.557995	-1.505851
H	-1.907868	1.932550	-0.345719
H	1.546152	-0.131060	-0.581929
H	2.405024	0.058786	2.420360
H	0.225386	0.693687	2.866028
H	-1.457231	0.446986	2.350686
H	-0.442722	-0.943981	2.774827
H	4.925852	2.764878	-1.020180
H	4.022202	2.616856	0.497799
H	6.348308	-0.937198	-0.320113
H	5.963245	-2.666858	-0.307360
H	2.724541	3.906868	-1.220726
H	2.771134	2.498044	-2.314801
H	1.878094	2.396862	-0.773356
H	6.422257	-1.898381	-2.641210
H	4.722185	-2.418506	-2.484301
H	5.120016	-0.678782	-2.494379

8R-III

Num. Imaginary Frequencies: 0

C	5.387210	1.181454	-0.729547
C	3.945977	1.689302	-0.665392
C	3.152584	1.001401	0.453552
C	3.335214	-0.509402	0.354941
C	4.702521	-1.139629	0.185133
C	5.441473	-0.335986	-0.914929
C	3.506186	1.579956	1.831275
O	1.760113	1.194128	0.252150
C	2.130671	-1.078626	0.328000
C	1.031799	-0.044731	0.349371
C	0.007318	-0.171618	-0.737139
C	-1.322720	-0.190663	-0.434250
C	-2.436854	-0.290193	-1.318206
P	-4.048881	-0.262109	-0.775775
O	-4.212732	0.892384	0.406514
C	-4.042794	2.251827	0.052166
C	-2.887053	2.867146	0.817860
C	5.505070	-1.112924	1.499950
C	4.573734	-2.599087	-0.263605
H	5.934619	1.473504	0.182641
C	0.487899	-0.231375	-2.163133
O	-5.135286	-0.147081	-1.806741
O	-4.309270	-1.544969	0.223928
C	-5.571978	-1.708554	0.848167
C	-5.507548	-1.374546	2.327014
H	0.499468	-0.076962	1.323557
H	6.488637	-0.678923	-0.967371
H	4.979664	-0.583661	-1.886854
H	5.914855	1.669303	-1.564006
H	3.916044	2.783487	-0.535488
H	3.433555	1.460346	-1.613897
H	3.032706	0.988026	2.628699
H	4.587962	1.610541	2.019804
H	3.121787	2.609440	1.886757
H	6.460735	-1.644571	1.366672
H	5.738952	-0.094526	1.835996
H	4.944934	-1.611211	2.305760
H	5.569581	-3.026986	-0.459579
H	4.090914	-3.214653	0.511121
H	3.977250	-2.681788	-1.184770
H	1.913682	-2.145025	0.253388
H	-1.570421	-0.111004	0.634877
H	-2.301521	-0.402426	-2.396619
H	0.048913	0.575746	-2.778671
H	1.582997	-0.130336	-2.221868
H	0.216529	-1.179951	-2.664296
H	-4.982669	2.784806	0.277361
H	-3.868612	2.348284	-1.033698
H	-6.328016	-1.087737	0.338827
H	-5.872627	-2.760352	0.706844

H	-2.800212	3.940137	0.587338
H	-3.039476	2.757945	1.902647
H	-1.941911	2.373360	0.548146
H	-6.481456	-1.558003	2.806653
H	-4.751122	-1.995191	2.830849
H	-5.237380	-0.318192	2.461640

II

Num. Imaginary Frequencies: 0

C	-5.814276	-1.566383	0.087195
C	-4.446018	-2.152823	-0.216248
C	-3.208799	-1.364197	0.311055
C	-3.418730	0.174150	0.000010
C	-4.831331	0.810661	0.017118
C	-5.902612	-0.170639	-0.506944
C	-3.185662	-1.477744	1.874750
O	-2.099293	-1.904351	-0.205098
C	-2.361258	1.001319	-0.210564
C	-0.969966	0.601796	-0.172929
C	0.120467	1.417433	-0.237124
C	1.419406	0.769313	-0.196004
C	2.628875	1.369251	-0.281814
P	4.148879	0.470237	-0.283953
O	3.758766	-1.104937	-0.233481
C	3.891237	-1.942299	-1.384919
C	2.711315	-1.817459	-2.327141
C	-5.188859	1.245287	1.454840
C	-4.924340	2.068795	-0.867029
H	-5.993330	-1.534785	1.175886
C	0.082111	2.918157	-0.354660
O	5.117816	0.791976	-1.372744
O	4.730083	0.724967	1.202433
C	6.040434	0.270481	1.551126
C	5.988372	-1.057531	2.279629
H	-0.866744	-0.492177	-0.088699
H	-6.897573	0.274397	-0.331939
H	-5.789318	-0.255240	-1.602812
H	-6.614537	-2.199294	-0.333101
H	-4.348086	-3.181856	0.168720
H	-4.306671	-2.214379	-1.310137
H	-2.361651	-0.862864	2.270079
H	-4.111883	-1.189476	2.398026
H	-2.966009	-2.531453	2.111211
H	-6.147531	1.789959	1.455156
H	-5.288881	0.396041	2.142638
H	-4.413137	1.913633	1.858787
H	-5.976982	2.384975	-0.943680
H	-4.364126	2.920894	-0.455354
H	-4.552114	1.872661	-1.884127
H	-2.559740	2.060828	-0.392734
H	1.393452	-0.320554	-0.090706
H	2.726388	2.453652	-0.391488
H	0.579101	3.257422	-1.279279
H	-0.941936	3.311484	-0.363788
H	0.615511	3.395358	0.484735
H	3.975645	-2.966266	-0.994671
H	4.829773	-1.693007	-1.903528
H	6.658392	0.204117	0.641287
H	6.475786	1.052010	2.191001

H	2.835411	-2.510696	-3.172289
H	1.766884	-2.059480	-1.818339
H	2.638250	-0.796329	-2.730701
H	7.001506	-1.359837	2.583827
H	5.363167	-0.980242	3.181163
H	5.566648	-1.839700	1.633191

TS_{II-8S_III}

Num. Imaginary Frequencies: 2

C	-5.750944	-0.121116	0.990990
C	-5.697114	1.400586	1.023508
C	-4.265890	1.873887	1.241150
C	-3.224588	1.381061	0.188507
C	-3.437392	-0.154498	0.003564
C	-4.847472	-0.769347	-0.085033
H	-6.102674	1.816943	0.085382
C	-3.515328	2.097354	-1.169722
O	-1.980993	1.671624	0.589551
C	-2.344024	-0.938976	-0.076869
C	-0.945503	-0.528518	0.086072
C	0.072910	-0.882999	-0.752875
C	-0.138407	-1.566768	-2.077526
C	-5.461189	-0.557785	-1.484323
C	-4.850750	-2.284414	0.176220
C	1.419301	-0.547559	-0.352795
C	2.561559	-0.809369	-1.036131
P	4.161926	-0.394011	-0.428289
O	4.469569	1.052058	-1.083100
C	5.734238	1.682089	-0.866108
C	5.631851	2.762448	0.191365
O	3.995322	0.003581	1.140993
C	4.318752	-0.925274	2.175923
C	3.171189	-1.865702	2.486587
O	5.239283	-1.402322	-0.655481
H	-0.707492	0.010568	1.001196
H	-6.786129	-0.474689	0.841423
H	-5.431942	-0.499616	1.978928
H	-6.355533	1.772610	1.826972
H	-4.206728	2.975038	1.275880
H	-3.899328	1.506804	2.216202
H	-2.902719	1.647716	-1.967922
H	-4.564857	2.111141	-1.503864
H	-3.179035	3.137801	-1.036421
H	-6.420235	-1.096465	-1.559248
H	-5.655774	0.497119	-1.710947
H	-4.788231	-0.948168	-2.263559
H	-5.889142	-2.648927	0.233164
H	-4.354430	-2.846674	-0.629320
H	-4.350440	-2.531937	1.124842
H	-2.474981	-2.004156	-0.296095
H	1.509520	-0.037705	0.613244
H	2.546943	-1.315381	-2.006151
H	0.361047	-2.550535	-2.112292
H	-1.205710	-1.718653	-2.287351
H	0.280676	-0.969630	-2.905566
H	4.571652	-0.314415	3.054280
H	5.216859	-1.491252	1.883006
H	6.482984	0.921684	-0.590947
H	6.039119	2.107720	-1.833698

H	3.445451	-2.523538	3.324717
H	2.266567	-1.306387	2.766971
H	2.931977	-2.497324	1.618081
H	6.602067	3.267402	0.310673
H	4.881691	3.513768	-0.095204
H	5.337300	2.329154	1.157227
H	1.886538	-3.258051	-1.671957
H	1.165870	-2.079760	-0.548957
H	2.041926	-1.515735	-1.992656
H	7.159769	-0.512540	2.568262
H	5.691214	0.347944	3.104076
H	5.543601	-1.130950	2.118686

TS$_{II\text{-}8R\_III}$

Num. Imaginary Frequencies: 1

C	-5.425700	-1.520539	-0.627023
C	-3.954494	-1.861956	-0.821407
C	-2.963480	-1.192312	0.178024
C	-3.337259	0.328367	0.278988
C	-4.799608	0.803310	0.302780
C	-5.631404	-0.012970	-0.713275
C	-3.167088	-1.841116	1.588005
O	-1.693024	-1.362592	-0.220263
C	-2.317731	1.205936	0.324509
C	-0.899916	0.831615	0.383950
C	0.117545	1.461209	-0.276188
C	1.438226	0.919451	-0.073263
C	2.578571	1.228918	-0.739408
P	4.049631	0.285566	-0.517674
O	3.614934	-1.072166	0.268312
C	3.346382	-2.286309	-0.437967
C	2.032476	-2.275868	-1.197458
C	-5.394111	0.643784	1.717692
C	-4.941712	2.285693	-0.077242
H	-5.785380	-1.899592	0.345278
C	-0.076146	2.536648	-1.310372
O	4.851678	0.015643	-1.747459
O	4.870493	1.057596	0.643858
C	6.178290	0.614265	1.013055
C	6.139585	-0.221457	2.276728
H	-0.623292	0.050056	1.089412
H	-6.697313	0.245984	-0.587079
H	-5.349205	0.316348	-1.729503
H	-6.046623	-2.018509	-1.391218
H	-3.782380	-2.951797	-0.780927
H	-3.627351	-1.530531	-1.822207
H	-2.616059	-1.265268	2.349141
H	-4.210783	-1.943296	1.926973
H	-2.719793	-2.845885	1.535071
H	-6.411876	1.066585	1.747585
H	-5.460592	-0.403553	2.036460
H	-4.778553	1.179663	2.456588
H	-6.009071	2.547843	-0.155612
H	-4.496799	2.952100	0.677148
H	-4.467293	2.499928	-1.047231
H	-2.520861	2.280833	0.322028
H	1.497434	0.116362	0.669667
H	2.609431	1.999159	-1.515423
H	0.199031	2.174576	-2.316469
H	-1.119610	2.875547	-1.358699
H	0.559853	3.413863	-1.103537
H	3.344900	-3.071269	0.331515
H	4.182961	-2.486967	-1.126335
H	6.626676	0.053563	0.176958
H	6.781976	1.521984	1.161720

Z-matrix of the DFT-study on the 4-(epoxycyclohaxenetrienyl)pyridine rearrangement under acidic conditions.

41

Num. Imaginary Frequencies: 0

C	-4.744173	-1.843019	-0.631966
C	-3.394237	-1.390758	-0.049793
C	-3.225619	0.140164	-0.098988
C	-4.450909	1.041463	0.101180
C	-5.740477	0.413124	-0.472625
C	-5.932856	-1.051136	-0.119885
C	-3.235326	-1.860425	1.404178
O	-2.372986	-2.064113	-0.761317
C	-1.996703	0.752582	-0.202764
C	-0.731902	0.122166	-0.204649
C	0.510817	0.779120	-0.184956
C	0.682080	2.274237	-0.195888
C	-4.613883	1.275792	1.624667
C	-4.279240	2.418321	-0.566866
H	-6.056777	-1.186057	0.966616
C	1.608959	-0.072810	-0.138695
C	2.973743	0.332175	-0.088190
C	3.960268	-0.601489	-0.043555
C	5.398345	-0.347014	0.010689
H	-0.716765	-0.968404	-0.210488
H	-6.592096	1.017646	-0.124791
H	-5.717067	0.510534	-1.571372
H	-6.856826	-1.428864	-0.581616
H	-4.843025	-2.919390	-0.428613
H	-4.694280	-1.729781	-1.729196
H	-2.476817	-1.880428	-1.702778
H	-2.276505	-1.522405	1.820373
H	-4.041550	-1.497125	2.050953
H	-3.255687	-2.959198	1.405425
H	-5.407582	2.021702	1.779455
H	-4.900143	0.367706	2.169661
H	-3.684762	1.664000	2.066507
H	-5.243392	2.945929	-0.532842
H	-3.549543	3.057915	-0.051248
H	-3.982272	2.322306	-1.621704
H	-1.991059	1.840786	-0.215206
H	1.416629	-1.150472	-0.137113
H	3.209278	1.398210	-0.083870
H	3.668900	-1.657227	-0.047901
H	1.335248	2.584504	-1.024319
H	-0.268650	2.805245	-0.311974
H	1.144118	2.619801	0.740974
C	6.282005	-1.434324	0.056669
C	7.653075	-1.191332	0.108348
N	8.178577	0.032683	0.116332
C	7.340276	1.070935	0.072294
C	5.956557	0.940972	0.019146
H	5.907055	-2.459547	0.052240
H	8.354588	-2.030811	0.144780
H	7.791827	2.067925	0.079460
H	5.336199	1.837157	-0.015307

TS_{41-42}

Num. Imaginary Frequencies: 1

C	3.891783	-1.269684	1.613374
C	3.116891	-1.213473	0.293599
C	3.231679	0.175345	-0.345954
C	4.575735	0.893640	-0.390316
C	5.294987	0.706059	0.966084
C	5.311787	-0.732578	1.471831
C	3.527037	-2.327177	-0.672190
O	1.718862	-1.442894	0.544461
C	2.084007	0.696417	-0.802254
C	0.817208	-0.034240	-0.762952
C	-0.363685	0.503934	-0.198895
C	-0.321849	1.721779	0.685737
C	5.435583	0.323076	-1.538696
C	4.397120	2.396062	-0.642304
H	5.900429	-1.377620	0.799755
C	-1.531759	-0.152756	-0.527911
C	-2.848569	0.228957	-0.118247
C	-3.925874	-0.490357	-0.517217
H	0.690595	-0.884697	-1.439328
H	6.321843	1.093132	0.872490
H	4.788984	1.336448	1.717538
H	5.814588	-0.775953	2.448930
H	3.883723	-2.303048	1.992091
H	3.356258	-0.645496	2.351161
H	1.495549	-1.115454	1.427623
H	3.000255	-2.226931	-1.631648
H	4.604170	-2.314296	-0.871452
H	3.269549	-3.297034	-0.225022
H	6.350851	0.925768	-1.638526
H	5.745691	-0.715803	-1.370778
H	4.889938	0.365400	-2.492920
H	5.374536	2.896364	-0.575078
H	3.993587	2.598716	-1.645692
H	3.729563	2.854041	0.103075
H	2.024976	1.718016	-1.187267
H	-1.456617	-1.038350	-1.167672
H	-2.965426	1.111719	0.514093
H	-3.755898	-1.362654	-1.157406
H	-0.965812	1.598494	1.567375
H	0.698758	1.913848	1.041160
H	-0.664063	2.616041	0.142234
C	-5.325276	-0.222510	-0.180779
C	-5.734003	0.835010	0.646160
C	-6.324449	-1.055097	-0.702567
C	-7.091200	1.001622	0.901080
C	-7.656497	-0.798528	-0.383882
N	-8.040450	0.207033	0.400937
H	-5.017330	1.524671	1.093484
H	-6.068529	-1.894952	-1.351379
H	-7.425468	1.821774	1.544382
H	-8.446387	-1.441229	-0.785669

42

Num. Imaginary Frequencies: 0

C	-3.405313	0.585701	1.868720
C	-3.132857	1.094816	0.459455
C	-3.231062	-0.030711	-0.548830

C	-4.485783	-0.882718	-0.587152
C	-4.775893	-1.302692	0.877873
C	-4.748055	-0.154033	1.885798
C	-3.871506	2.375987	0.112096
O	-1.667497	1.455580	0.303514
C	-2.092516	-0.214447	-1.217276
C	-0.970558	0.673505	-0.786943
C	0.245860	-0.007908	-0.230559
C	0.013901	-1.228858	0.613718
C	-5.676971	-0.110222	-1.184545
C	-4.254131	-2.139164	-1.431795
H	-5.571018	0.553083	1.696586
C	1.448566	0.515010	-0.552659
C	2.750405	0.009404	-0.150582
C	3.893845	0.608224	-0.533472
C	5.257485	0.181823	-0.188233
H	-0.697825	1.434644	-1.529711
H	-5.753343	-1.808728	0.911276
H	-4.021716	-2.050635	1.176162
H	-4.916499	-0.547852	2.897930
H	-3.399469	1.418043	2.588199
H	-2.611225	-0.121582	2.157217
H	-1.155215	1.572483	1.123793
H	-3.727533	2.640214	-0.943610
H	-4.944677	2.263077	0.302664
H	-3.503438	3.193095	0.747266
H	-6.534075	-0.791314	-1.292825
H	-6.006964	0.728735	-0.559300
H	-5.425571	0.285791	-2.179511
H	-5.143400	-2.784947	-1.385843
H	-4.079110	-1.886584	-2.488578
H	-3.394000	-2.719039	-1.064968
H	-1.915678	-0.971549	-1.979771
H	1.465660	1.405943	-1.189948
H	2.784013	-0.883504	0.478477
H	3.828790	1.502530	-1.162827
H	0.917564	-1.550538	1.143055
H	-0.765849	-1.048478	1.369082
H	-0.338948	-2.068726	-0.005332
C	6.350531	0.935342	-0.637424
C	7.642925	0.531346	-0.309679
N	7.913438	-0.550868	0.419526
C	6.875222	-1.272908	0.848650
C	5.547846	-0.957448	0.578260
H	6.196764	1.833304	-1.239601
H	8.499918	1.117622	-0.658188
H	7.108848	-2.160763	1.445764
H	4.758268	-1.603179	0.965224

$TS_{41\text{-}43}$

Num. Imaginary Frequencies: 1

C	-5.566857	-1.394759	0.245726
C	-4.202401	-2.059331	0.096510
C	-3.051543	-1.113571	0.467030
C	-3.204505	0.217100	-0.266939
C	-4.558990	0.923939	-0.284040
C	-5.647868	-0.131347	-0.603322
C	-2.906748	-0.928378	1.979417
O	-1.831629	-1.673133	-0.031624
C	-2.110198	0.711436	-0.863258
C	-0.810232	0.045043	-0.939575
C	0.341297	0.496867	-0.274407
C	0.279509	1.541307	0.808701
C	-4.848437	1.604304	1.070338
C	-4.608875	2.008075	-1.368295
H	-5.774915	-1.169329	1.304402
C	1.539875	-0.079849	-0.678842
C	2.828105	0.230326	-0.168273
C	3.935730	-0.393718	-0.660892
H	-0.666748	-0.684743	-1.741545
H	-6.634227	0.345107	-0.491434
H	-5.557058	-0.413662	-1.666206
H	-6.349739	-2.100587	-0.067583
H	-4.129046	-2.972426	0.707897
H	-4.050696	-2.359631	-0.952595
H	-1.374774	-2.147189	0.672446
H	-2.120392	-0.194966	2.211129
H	-3.835886	-0.591692	2.450390
H	-2.646906	-1.894543	2.442835
H	-5.748814	2.229263	0.977712
H	-5.035248	0.893829	1.884759
H	-4.014712	2.256726	1.370753
H	-5.629358	2.411724	-1.437296
H	-3.945163	2.855500	-1.137074
H	-4.338707	1.608455	-2.357094
H	-2.128205	1.704996	-1.325065
H	1.494282	-0.838060	-1.469447
H	2.916618	0.979540	0.621358
H	3.789909	-1.135209	-1.455295
H	-0.755838	1.850475	1.000257
H	0.696886	1.160558	1.753432
H	0.851868	2.439255	0.530339
C	5.316511	-0.188418	-0.249737
C	6.329939	-0.931720	-0.873169
C	5.698145	0.723435	0.748711
C	7.652463	-0.734588	-0.477657
C	7.047630	0.840409	1.064951
N	8.004434	0.129861	0.468437
H	6.094125	-1.654929	-1.656948
H	4.967944	1.337861	1.277238
H	8.459721	-1.303030	-0.949870
H	7.371772	1.543378	1.838571

43

Num. Imaginary Frequencies: 0

C	-5.426241	-1.393354	0.312852
C	-4.016763	-1.989595	0.237857
C	-2.988323	-0.916398	0.570752
C	-3.192239	0.305432	-0.302364
C	-4.563025	0.951202	-0.363725
C	-5.583099	-0.189480	-0.615374
C	-2.815156	-0.668010	2.059811
O	-1.649307	-1.374152	0.037070
C	-2.116174	0.589109	-1.037010
C	-0.968164	-0.346086	-0.843895
C	0.281045	0.176475	-0.194932
C	0.153820	1.150076	0.946747
C	-4.884909	1.697290	0.945379

C	-4.633802	1.954614	-1.518375
H	-5.668479	-1.118072	1.351800
C	1.456555	-0.282424	-0.677912
C	2.775789	0.073996	-0.184303
C	3.900550	-0.439061	-0.717958
C	5.276210	-0.156317	-0.284276
H	-0.727708	-0.904479	-1.757518
H	-6.600154	0.224130	-0.529598
H	-5.466329	-0.530556	-1.657959
H	-6.150832	-2.170926	0.032310
H	-3.908465	-2.846809	0.918593
H	-3.822135	-2.341408	-0.786759
H	-1.067756	-1.839224	0.664704
H	-2.174969	0.199924	2.258540
H	-3.791633	-0.490329	2.523939
H	-2.391429	-1.559529	2.545695
H	-5.834271	2.241023	0.829807
H	-4.997522	1.029562	1.808756
H	-4.097368	2.428252	1.181528
H	-5.653295	2.360779	-1.593128
H	-3.948135	2.800526	-1.358372
H	-4.385576	1.482059	-2.480394
H	-2.031529	1.397448	-1.761509
H	1.435070	-0.990279	-1.513839
H	2.835885	0.772138	0.654589
H	3.808047	-1.138872	-1.555684
H	-0.794577	1.702553	0.894560
H	0.190348	0.636774	1.921305
H	0.965504	1.889276	0.939551
C	6.349434	-0.758048	-0.955382
C	7.652932	-0.490033	-0.542815
N	7.952090	0.319473	0.472682
C	6.932693	0.894618	1.115719
C	5.596726	0.694968	0.784523
H	6.172130	-1.432547	-1.795733
H	8.494623	-0.957904	-1.064584
H	7.189724	1.557116	1.949049
H	4.823508	1.202427	1.363101
